# Global burden and trends of hematologic malignancies based on Global Cancer Observatory 2022 and Global Burden of Disease 2021

**DOI:** 10.1186/s40164-025-00684-x

**Published:** 2025-07-17

**Authors:** Tao Pan, Jiyue Zhang, Xiaomin Wang, Yuqin Song

**Affiliations:** 1 Key Laboratory of Carcinogenesis and Translational Research (Ministry of Education/Beijing), Department of Lymphoma, Peking University Cancer Hospital & Institute, Beijing, 100142 China; 2 Key Laboratory of Carcinogenesis and Translational Research (Ministry of Education), Laboratory of Lymphoma Translational Research, Peking University Cancer Hospital & Institute, Beijing, 100142 China

**Keywords:** Hematologic malignancies, GBD 2021, GLOBOCAN 2022, Prevalence, Incidence, Deaths, DALYs

## Abstract

**Background:**

Hematologic malignancies are one of the most common types of cancer. This study aims to assess the global burden of hematologic malignancies and analyze the global epidemiological trends.

**Methods:**

Through the Global Burden of Disease Study 2021 (GBD 2021) and the Global Cancer Observatory (GLOBOCAN) 2022 project, we comprehensively evaluated the global prevalence, incidence, mortality, and disability-adjusted life-years (DALYs) of seven major hematologic malignancies, as well as their respective age-standardized rates (ASR) per 100,000 population. Regions were classified using the Socio-demographic Index (SDI) to evaluate the correlation between disease burden and economic level. In addition, we analyzed disease-related risk factors and predicted future trends up to 2040.

**Results:**

From 1990 to 2021/2022, the number of global hematologic malignancy cases showed a continuously increasing trend, especially for non-Hodgkin lymphoma. However, the age-standardized death rates (ASDR) and age-standardized DALY rates (ASDALYR) of all types of hematologic malignancies tended to be stable or decline. For acute lymphoblastic leukemia, the number of death cases, ASDR, and ASDALYR decreased significantly. Nevertheless, the trends of hematologic malignancies varied by gender, age, and SDI. The burden of hematologic malignancies was generally higher in the elderly and male populations. Of course, acute lymphoblastic leukemia also imposed a huge burden on children, Hodgkin lymphoma also significantly burdened young people. Moreover, regions with a higher SDI had a higher incidence rate. Deaths related to smoking and high body mass index still played an important role in various regions, especially in regions with a higher SDI. It is predicted that the global age-standardized incidence rates (ASIR) and ASDALYR will show a slow downward trend by 2040.

**Conclusions:**

Hematologic malignancies have remained a major global public health issue, with significant demographic and regional differences. The results of this study will provide a basis for analyzing the trends of the global disease burden of specific hematologic malignancies and offer a reference for health policymakers.

**Supplementary Information:**

The online version contains supplementary material available at 10.1186/s40164-025-00684-x.

## Introduction

Hematologic malignancies, a group of diseases that pose a severe threat to global public health, have drawn increasing attention in recent years [1, 2]. These malignancies originate from the abnormal proliferation and differentiation of hematopoietic cells in the bone marrow, blood, and lymphatic system. They cover a wide range of subtypes, including lymphoma, leukemia, multiple myeloma, and myelodysplastic syndromes [3]. Hodgkin lymphoma (HL) is typified by a tumor microenvironment with scarce malignant cells and abundant immune effector cells. It most commonly affects adolescents and young adults, yet can also afflict the elderly [4]. Non-Hodgkin lymphoma are malignant disorders arising from cells of the immune system. The incidence of Non-Hodgkin lymphoma (NHL) peaks among individuals aged 60 and above, and it exhibits a higher prevalence in men compared to women [5]. Leukemia encompasses several major types. Acute Lymphoblastic Leukemia (ALL) predominantly affects children, peaking between 2 and 5 years old, and also occurs in young adults, with a poorer prognosis in the latter group [6]. Acute Myeloid Leukemia (AML) is more common in older adults and those with genetic disorders like Down syndrome [7]. Chronic Lymphocytic Leukemia (CLL) mainly strikes elderly individuals, with men being more susceptible [8]. Chronic Myeloid Leukemia (CML) typically impacts middle-aged and older adults, and having a family history of hematological disorders may slightly elevate the risk [9]. Multiple myeloma (MM) is the second most common haematological malignancy in high-income countries [10].

Findings from the latest Global Cancer Observatory (GLOBOCAN) 2022 research indicate that, among hematological malignancies, non-Hodgkin lymphoma exhibits the highest incidence, ranking 10th out of 33 cancer categories, with its mortality rate ranking 11th. Leukemia, conversely, has the highest mortality rate among hematological malignancies, ranking 10th overall, and its incidence rate is 13th. Multiple myeloma occupies a middle-ranking position, with an incidence ranking of 21st and a mortality ranking of 17th. Hodgkin lymphoma has relatively lower incidence and mortality rates, ranking 26th in incidence and 28th in mortality [11]. These statistics underscore the distinct epidemiological profiles of various hematological malignancies within the broader spectrum of cancers. The variations in incidence and mortality rankings among these diseases highlight the need for tailored approaches in cancer prevention, diagnosis, and treatment strategies. Understanding these differences is crucial for healthcare providers, researchers, and public health policymakers to allocate resources effectively and develop targeted interventions aimed at reducing the global burden of hematologic malignancies.

Although hematologic malignancies are a major global health concern, evidence on their disease burden remains limited, with only a few studies reporting the burden of individual subtypes [2, 11–14]. The latest updates of the GLOBOCAN 2022 project and the Global Burden of Disease (GBD) study in 2021 have provided an expanded dataset, which has improved the accuracy and coverage of the estimates of the cancer burden [11, 15–18]. The GBD 2021 and GLOBOCAN 2022 are comprehensive and in-depth research initiatives aiming to provide a detailed understanding of the global health landscape. In the context of hematologic malignancies, the GBD 2021 and GLOBOCAN 2022 can provide insights into their global burden trends. For instance, it can show how the incidence and mortality of leukemia, lymphoma, and multiple myeloma have changed over the years across different regions. This information can help in understanding the impact of risk factors such as environmental exposures, lifestyle changes, and genetic predispositions in different populations. This knowledge can provide a solid foundation for precise prevention and control strategies and the optimization of treatment approaches, aiming to alleviate the heavy burden these diseases impose on human society.

## Methods

### Data source

In this study, we present a comprehensive overview of the hematologic malignancies burden estimation procedures for GBD 2021 and GLOBOCAN 2022. GBD 2021 for subtype analysis and long-term trends; GLOBOCAN 2022 for validating post-2021 estimates. Additionally, we highlight the crucial disparities between the two databases. Hematologic malignancies are identified by the International Classification of Diseases (ICD-10) code (Table S1).

GBD 2021 is an extremely comprehensive epidemiological study that provides data on 371 diseases and injuries for 204 countries and regions worldwide. Its data sources are extensive and diverse, covering conventional channels such as vital registration systems, epidemiological surveys, disease monitoring systems, cancer registries, police records, and open-source databases on one hand, and on the other hand, raw data is collected from disease-specific registries, health service contact data, censuses, household surveys, and other sources. In GBD 2021, the Bayesian disease meta-regression algorithm DisMod-MR 2.1 was used to estimate prevalence, generating consistent estimates of prevalence, incidence, remission, and mortality stratified by sex, location, year, and age group. For areas lacking data, it estimated prevalence by cascading down the five-level GBD geographical hierarchy, using higher-level data as priors and incorporating country-level covariates. Uncertainty was calculated with 1000 computational draws, reporting 95% uncertainty intervals. To estimate incidence and mortality across all age groups and sexes, the cause-of-death ensemble model was applied for cause-specific mortality, while DisMod-MR 2.1 was used for incidence estimation via an analytical cascade. Prior to modeling, data were adjusted by disaggregating them by age and sex and applying a meta-regression model to correct biases, with detailed information on bias correction and other modifications for specific disorders provided in the GBD 2021 capstone report. The complete dataset related to this project can be obtained from the Global Health Data Exchange query tool (http://ghdx.healthdata.org/) [16, 17].

The GLOBOCAN 2022 project provides estimates for 36 types of cancers in 185 countries and regions. The data sources include cancer registries at the national and subnational levels, mortality databases, and prediction models. In addition, non-specific or unclear cases have been reallocated to specific cancer categories to enhance accuracy. In GLOBOCAN 2022, when only mortality or incidence data were available, mortality-to-incidence ratios were employed. These ratios were extrapolated using two methods: [1] involved utilizing country-specific mortality-to-incidence ratios; [2] entailed regional modeling of mortality-to-incidence ratios. The regional models were constructed based on incidence and mortality data derived from population-based cancer registries that contributed data to Cancer Incidence in Five Continents Vol. XI. For each combination of sex and cancer site, both mortality-to-incidence and incidence-to-mortality ratios were calculated. The complete dataset related to this project can be obtained from the Cancer Today (https://gco.iarc.who.int/today/en/dataviz/) [11].

This analysis employed data from the GBD 2021 and GLOBOCAN 2022, encompassing the incidence of cases, prevalence of cases, mortality, and disability-adjusted life years (DALYs) for Hodgkin lymphoma (HL), Non − Hodgkin lymphoma (NHL), Acute myeloid leukemia (AML), Chronic myeloid leukemia (CML), Acute lymphoid leukemia (ALL), Chronic lymphoid leukemia (CLL) and Multiple myeloma (MM), along with their respective age-standardized rates per 100,000 persons (ASR). ASR elucidates variations in age distribution and population size, enabling significant comparisons across temporal and regional contexts. It is worth noting that the GLOBOCAN 2022 project aggregates leukemia subtypes (e.g., AML, CML, ALL, CLL) into a single category (‘leukemia’), whereas the GBD 2021 study separately analyzed four leukemia subtypes (AML, CML, ALL, CLL), along with HL, NHL, and MM. To ensure consistency in subtype-specific analyses, this study primarily utilized GBD 2021 data for leukemia subtypes. GLOBOCAN 2022 data were used only for validating global leukemia trends as a whole. The 204 nations and 21 areas in the GBD 2021 research were categorized into five classifications, ranging from low Socio-demographic Index (SDI) to high SDI. This value was computed as the geometric mean of three principal indicators: fertility rate, average years of schooling, and per capita income.

### Risk factors

The GBD 2021 database offers a thorough assessment of the influence of risk factor exposure on particular health outcomes [19]. In this study, we searched for common risk factors to explore their association with hematologic malignancies, including high body-mass index (BMI) and tobacco. However, these risks were not found in HL. While environmental toxins (e.g., benzene, pesticides) and alcohol consumption may contribute to hematologic malignancy risk, their exclusion from our analysis reflects limitations in current global risk factor surveillance systems rather than biological irrelevance. The GBD 2021 framework prioritizes risk factors with robust epidemiological consensus, and insufficient evidence for these exposures at a global scale. Future iterations of cancer burden studies should integrate region-specific environmental and lifestyle data to address this critical gap. The specific risk factors related to hematologic malignancies were summarized as follows: [1] NHL: high BMI; [2] AML: high BMI and tobacco; [3] CML: high BMI and tobacco; [4] ALL: high BMI and tobacco; [5] CLL: high BMI and tobacco; [6] MM: high BMI. In the GBD study, High BMI for adults (ages 20+) is defined as BMI greater than 20 to 23 kg/m^2^. High BMI for children and adolescents (ages 2–19) is defined as being overweight or obese based on International Obesity Task Force standards [20]. The definition of tobacco includes smoking, chewing tobacco, and secondhand smoke (Table S1).

### Statistical analysis

To assess temporal trends, the estimated annual percentage change (EAPC) of ASR was calculated, including age-standardized prevalence rates (ASPR), age-standardized incidence rates (ASIR), mortality rates (ASDR), and DALY rates (ASDALYR) per 100,000 population. The formula y = α + βx + ε was used (where y = ln [ASR], and x = calendar year). The formula for calculating the EAPC value is EAPC = 100 × (exp(β) − 1). Positive EAPC values with 95% confidence intervals (CIs) exceeding zero showed upward trends, whilst negative values denoted declines. Moreover, the Pearson correlation coefficient was selected as an initial exploratory step to identify broad patterns of association across SDI strata, consistent with prior burden studies [20, 21]. The Bayesian age-period-cohort models (BAPC) with integrated nested Laplace approximation (INLA) were applied to forecast the ASIR and ASDALYR of future hematologic till 2040. Statistical analyses were conducted using R (version 4.4.2). A two-tailed P-value of less than 0.05 was deemed statistically significant.

## Results

### Hematologic malignancies burden

**Fig. 1:**
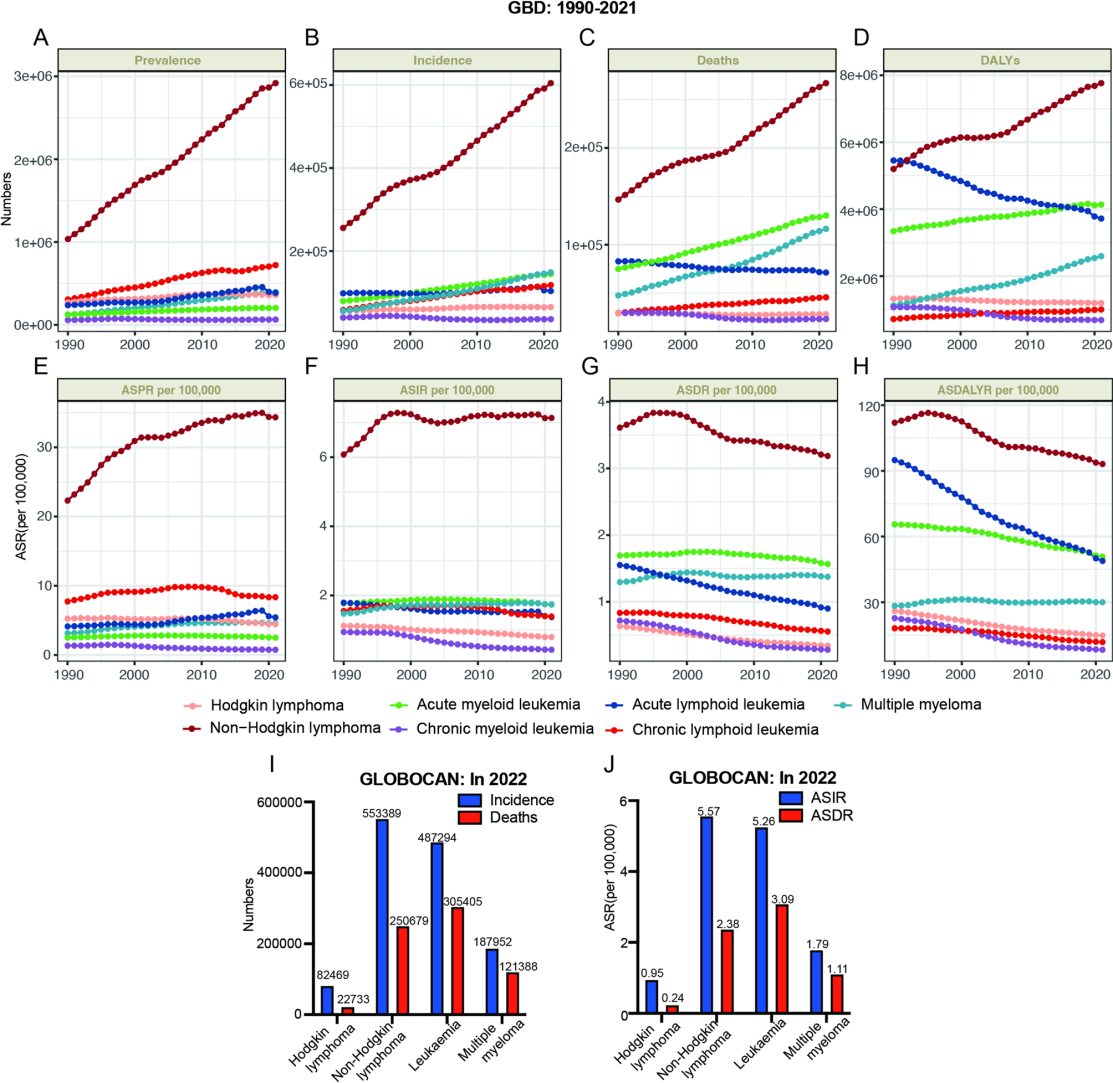
Global trends in prevalence, incidence, deaths, and DALYs. A-D: The absolute numbers for prevalence (**A**), incidence (**B**), deaths (**C**), and DALYs (**D**) were displayed globally in 1990–2021 from GBD. E-H: The ASPR (**E**), ASIR (**F**), ASDRs (**G**), and ASDALYR (**H**) were shown globally in 1990–2021 from GBD. **I**: The absolute numbers for incidence, death from GLOBOCAN 2022. **J**: The ASIR and ASDR from GLOBOCAN 2022. DALYs, disability-adjusted life years; ASPR, age-standardized prevalence rate; ASIR, age-standardized incidence rate; ASDR, age-standardized death rate; ASDALYR, age-standardized DALY rate

**Fig. 2:**
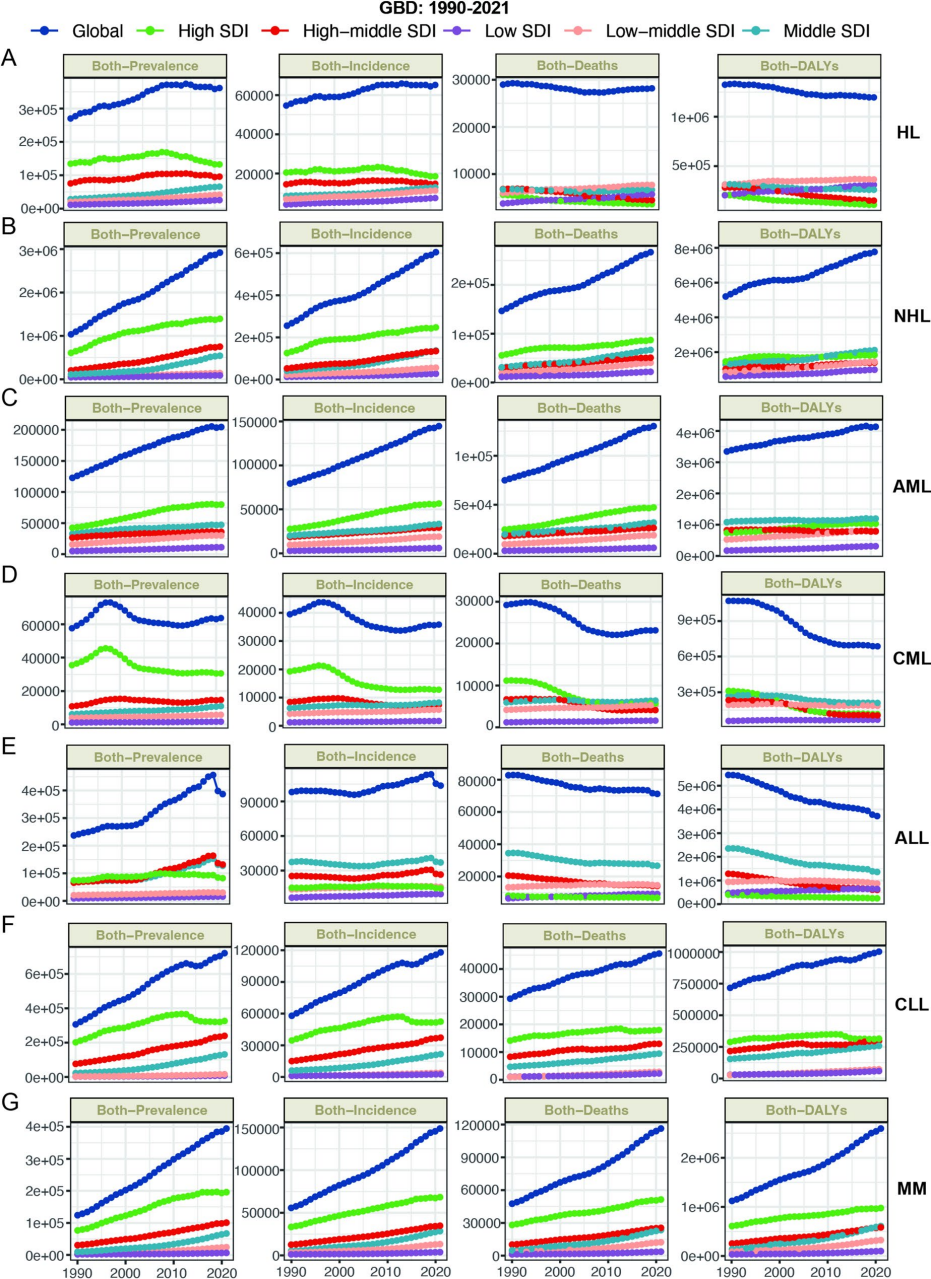
Global and regional trends in prevalence, incidence, deaths, and DALYs in 1990–2021 from GBD. (**A**): HL, (**B**): NHL, (**C**): AML, (**D**): CML, (**E**): ALL, (**F**): CLL, (**G**): MM. HL, Hodgkin lymphoma; NHL, Non − Hodgkin lymphoma; AML, Acute myeloid leukemia; CML, Chronic myeloid leukemia; ALL, Acute lymphoid leukemia; CLL, Chronic lymphoid leukemia; MM, Multiple myeloma; DALYs, disability-adjusted life years; SDI, socio-demographic index

Fig. 1 presents the trend of events for seven major hematologic malignancies on a global scale. In GBD 2021, the NHL demonstrated a notable rise in prevalence, incidence, mortality, and DALY from 1990 to 2021; however, ASDR and ASDALYR exhibited a declining trend. CML exhibited a consistent decline across nearly all eight metrics. In the last 30 years, ALL exhibited a notable decline in mortality, DALY, ASDR, and ASDALYR. Additionally, the prevalence of ALL, along with morbidity, ASPR, and ASIR, demonstrated a significant inflection point in 2019, transitioning from an increasing to a decreasing trend. CLL exhibited a slight rise in prevalence, morbidity, mortality, and DALY, alongside a minor decline in ASPR, ASIR, ASDR, and ASDALYR. AML and MM exhibited an increase in mortality and DALY. However, the ASDALYR for AML demonstrated a downward trend, whereas the ASDALYR for MM remained stable (Fig. 1). Of course, we also analyzed the incidence and mortality data of Hodgkin lymphoma, non-Hodgkin lymphoma, leukemia, and multiple myeloma in 2022 from the GLOBOCAN 2022 database. The overall trends are consistent with those of the GBD 2021 (Fig. 1I and J). We also analyzed the effect of regional SDI levels on the burden of hematologic malignancies at different gender levels, and the overall trends were generally consistent (Fig. 2; Figure S1; Figure S2; Figure S3; Figure S4; Figure S5).

### Hodgkin lymphoma burden

**Fig. 3:**
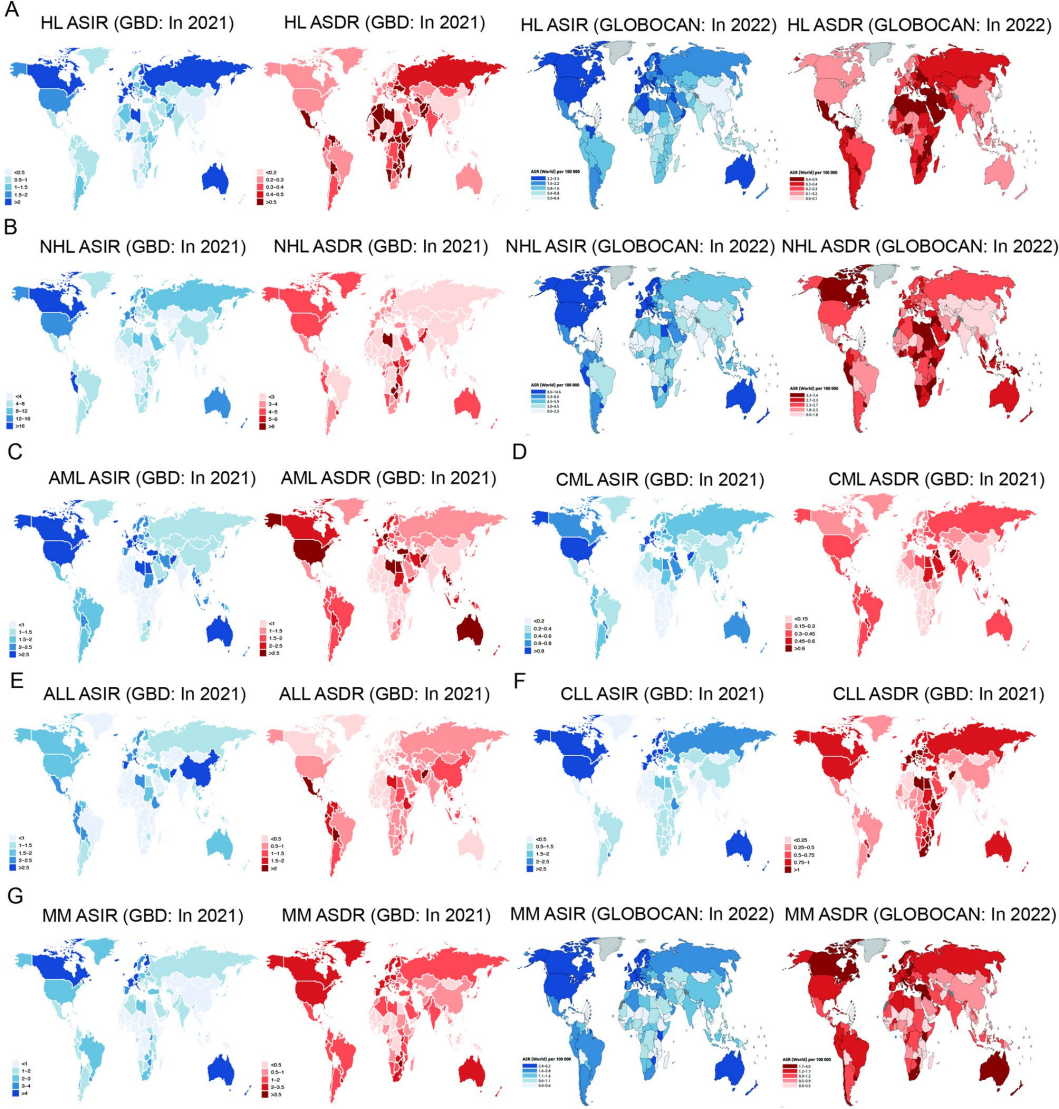
In 2021/2022, global distribution of ASIR and ASDR in GBD 2021 and GLOBOCAN 2022. (**A**): HL, (**B**): NHL, (**C**): AML, (**D**): CML, (**E**): ALL, (**F**): CLL, (**G**): MM. HL, Hodgkin lymphoma; NHL, Non − Hodgkin lymphoma; AML, Acute myeloid leukemia; CML, Chronic myeloid leukemia; ALL, Acute lymphoid leukemia; CLL, Chronic lymphoid leukemia; MM, Multiple myeloma; ASIR, age-standardized incidence rate; ASDR, age-standardized death rate

In GBD 2021, from 1990 to 2010, the prevalent and incident cases of HL continued to increase, while from 2010 to 2021, they remained relatively stable (Fig. 2A). During the period from 1990 to 2021, the global number of cases increased from 0.27 million to 0.36 million, while the ASPR decreased from 5.24 to 4.42 (EAPC = -0.48) (Table S2). The global incident cases of HL increased from 54,671 in 1990 to 65,182 in 2021, while the ASIR decreased from 1.12 to 0.79 (EAPC=-1.10) (Table 1). According to the GLOBOCAN 2022 database, in 2022, the number of incident cases of HL globally was 82,469, and the ASIR was 0.95 (Fig. 1I and J). In 2021, from GBD 2021, the incidence rate decreased sequentially from high SDI countries to low SDI countries; the ASIR in high SDI countries was the highest (1.42) (Fig. 2A; figure S1A; Table 1). In the 21 global regions, Western Europe had the highest incidence rate and ASIR, at 11,998 and 2.37, respectively (Table 1). At the country level, India had the highest number of new cases (7,455), followed by the United States (6,837) and China (4,211); consistent with ASPR, Monaco (9.40), Greece (5.44), and San Marino (4.99) ranked the top three in ASIR (Fig. 3A; figure S6B; Table S5). According to the GLOBOCAN 2022 database, in 2022, Italy (3.48), Malta (3.3), and Portugal (3.19) ranked the top three in ASIR (Fig. 3A).

**Table 1:** The incidence of HL in 1990 and 2021. Annotations: ASIR: Age-standardized incidence rate (per 100,000 population); EAPC: estimated annual percentage change; SDI: Socio-demographic index

Location	1990 incident cases (95% CI)	1990 ASIR (95% CI)	2021 incident cases (95% CI)	2021 ASIR (95% CI)	EAPC (95% CI)
Global	54,671 (45649–59832)	1.12 (0.93–1.23)	65,182 (53167–77143)	0.79 (0.64–0.94)	-1.1 (-1.16 to -1.05)
High SDI	20,496 (19866–21164)	2.11 (2.04–2.17)	18,581 (17509–19686)	1.42 (1.34–1.51)	-1.18 (-1.41 to -0.95)
High-middle SDI	14,472 (12560–15815)	1.33 (1.15–1.45)	14,769 (13072–16109)	0.99 (0.88–1.08)	-0.93 (-1.04 to -0.82)
Middle SDI	8508 (5676–10032)	0.6 (0.4–0.71)	12,968 (9236–15913)	0.51 (0.36–0.62)	-0.59 (-0.74 to -0.43)
Low-middle SDI	6959 (4557–9352)	0.75 (0.49–1.04)	11,301 (8144–16508)	0.62 (0.45–0.91)	-0.69 (-0.75 to -0.63)
Low SDI	4162 (2423–5219)	1.12 (0.65–1.44)	7494 (4361–10262)	0.83 (0.49–1.15)	-1.13 (-1.19 to -1.06)
Andean Latin America	143 (105–182)	0.5 (0.37–0.63)	362 (259–465)	0.56 (0.4–0.72)	0.57 (0.41 to 0.73)
Australasia	367 (321–422)	1.68 (1.46–1.94)	669 (545–816)	1.88 (1.52–2.31)	0.73 (0.29 to 1.17)
Caribbean	420 (362–474)	1.29 (1.11–1.45)	478 (401–567)	0.94 (0.79–1.12)	-1.22 (-1.37 to -1.07)
Central Asia	555 (485–635)	0.88 (0.77–1.01)	732 (627–852)	0.77 (0.66–0.89)	-0.46 (-0.64 to -0.28)
Central Europe	2788 (2582–2968)	2.07 (1.92–2.21)	2046 (1834–2315)	1.55 (1.39–1.76)	-1.15 (-1.31 to -0.98)
Central Latin America	1163 (1115–1210)	0.95 (0.91–0.98)	2084 (1870–2320)	0.8 (0.72–0.9)	-0.52 (-0.72 to -0.31)
Central Sub-Saharan Africa	148 (103–228)	0.43 (0.29–0.68)	359 (229–571)	0.39 (0.26–0.62)	-0.23 (-0.27 to -0.2)
East Asia	4886 (2132–6702)	0.48 (0.21–0.66)	4434 (2748–5810)	0.23 (0.15–0.31)	-2.63 (-2.88 to -2.37)
Eastern Europe	5221 (4927–5609)	2.21 (2.09–2.37)	4771 (4385–5193)	2.09 (1.93–2.28)	-0.36 (-0.54 to -0.18)
Eastern Sub-Saharan Africa	1829 (1179–2409)	1.32 (0.83–1.77)	3439 (2010–4894)	1.02 (0.6–1.41)	-1.02 (-1.08 to -0.97)
High-income Asia Pacific	326 (303–370)	0.17 (0.16–0.19)	664 (586–727)	0.27 (0.24–0.3)	2.34 (2.07 to 2.61)
High-income North America	10,518 (10224–10809)	3.39 (3.3–3.49)	7707 (7369–8064)	1.73 (1.65–1.81)	-2.22 (-2.55 to -1.88)
North Africa and Middle East	1925 (1415–2863)	0.73 (0.54–1.16)	4756 (3067–6397)	0.8 (0.52–1.09)	0.56 (0.45 to 0.66)
Oceania	8 (5–13)	0.17 (0.11–0.26)	16 (10–23)	0.14 (0.08–0.19)	-0.51 (-0.6 to -0.42)
South Asia	7437 (4518–9749)	0.83 (0.5–1.11)	11,940 (8413–17754)	0.66 (0.46–0.98)	-0.86 (-0.94 to -0.78)
Southeast Asia	1508 (1162–2485)	0.39 (0.3–0.68)	2590 (1944–4498)	0.36 (0.27–0.63)	-0.4 (-0.49 to -0.31)
Southern Latin America	534 (453–643)	1.11 (0.94–1.34)	818 (683–973)	1.11 (0.92–1.32)	0.32 (0.02 to 0.63)
Southern Sub-Saharan Africa	174 (121–249)	0.42 (0.29–0.61)	365 (226–472)	0.48 (0.3–0.62)	0.68 (0.43 to 0.92)
Tropical Latin America	869 (828–911)	0.69 (0.66–0.73)	1259 (1166–1349)	0.51 (0.48–0.55)	-0.71 (-0.9 to -0.52)
Western Europe	11,986 (11408–12597)	2.77 (2.63–2.91)	11,998 (11186–12979)	2.37 (2.21–2.56)	-0.17 (-0.48 to 0.15)
Western Sub-Saharan Africa	1864 (706–2779)	1.34 (0.55–1.98)	3694 (1331–5893)	0.99 (0.38–1.52)	-1.15 (-1.24 to -1.06)

In GBD 2021, the global number of HL deaths declined from 28,977 in 1990 to 28,180 in 2021, with the ASDR decreasing by almost half, from 0.63 to 0.34 (EAPC=-2.07) (Table S3). According to the GLOBOCAN 2022 database, in 2022, the number of deaths of HL globally was 22,733, and the ASDR was 0.24 (Fig. 1I and J). In 2021, from GBD 2021, the highest number of deaths was in low − middle SDI countries (7,700), while the highest ASDR was in low SDI countries (0.70) (Fig. 2A; Figure S1A; Table S3). Among the 21 global regions, South Asia had the highest number of deaths (7,968); while Eastern Sub-Saharan Africa (0.85) and Western Sub-Saharan Africa (0.84) ranked first and second in ASDR, respectively (Table S3). At the 204 countries level, India had the highest number of deaths (4,893), followed by China (2,443) and Nigeria (2,383); while Nigeria (1.56) had the highest ASDR, followed by Pakistan (1.23) and Somalia (1.22) (Fig. 3A, S6C; Table S5). According to the GLOBOCAN 2022 database, in 2022, Iraq (0.91), Yemen (0.91), and Gaza Strip and West Bank (0.84) ranked the top three in ASDR (Fig. 3A).

Between 1990 and 2021, from GBD 2021, global HL DALYs decreased from 1.32 million to 1.20 million and ASDALYR decreased from 26.05 to 14.82 (EAPC =-1.88) (Table S4). In 2021, from GBD 2021, DALYs were highest in low-middle SDI countries (0.37 million) and ASDALYR was highest in low SDI countries (30.28) (Fig. 2A; Figure S1A; Table S4). Among the 21 global regions, South Asia DALYs were the highest (0.38 million); while Eastern Sub-Saharan Africa (36.96) and Western Sub-Saharan Africa (34.29) were ranked first and second in ASDALYR, respectively (Table S4). At the country level, India had the highest DALYs (0.22 million), followed by Nigeria (0.13 million) and Pakistan (0.13 million); while Nigeria (63.66) had the highest ASDALYR, followed by Pakistan (54.93) and Somalia (52.24) (Figure S6D, S6F; Table S5).

**Fig. 4:**
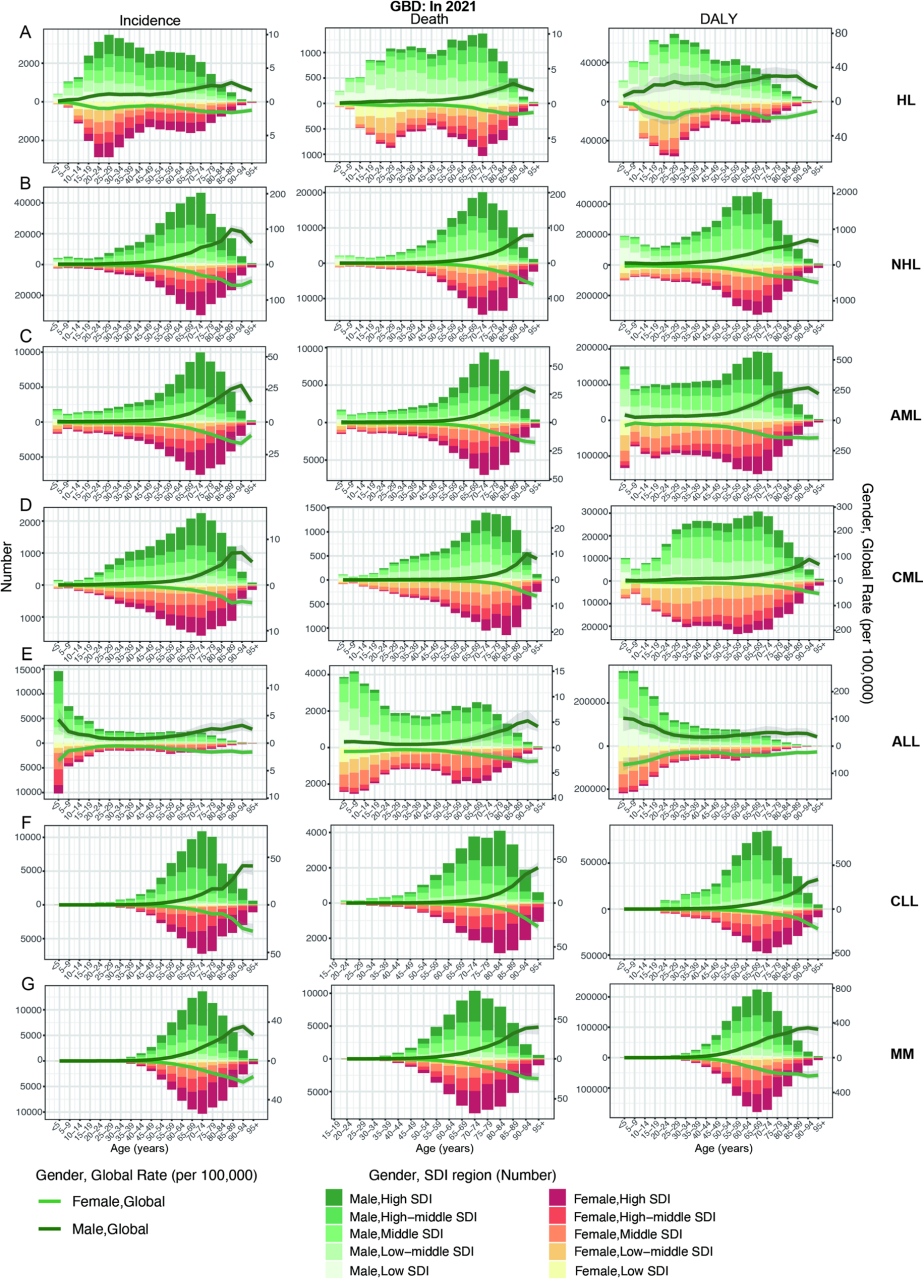
Distribution of incidence, deaths and DALYs by gender, age group, SDI regions in 2021 from GBD. (**A**): HL, (**B**): NHL, (**C**): AML, (**D**): CML, (**E**): ALL, (**F**): CLL, (**G**): MM. HL, Hodgkin lymphoma; NHL, Non − Hodgkin lymphoma; AML, Acute myeloid leukemia; CML, Chronic myeloid leukemia; ALL, Acute lymphoid leukemia; CLL, Chronic lymphoid leukemia; MM, Multiple myeloma; DALYs, disability-adjusted life years; SDI, socio-demographic index

**Fig. 5:**
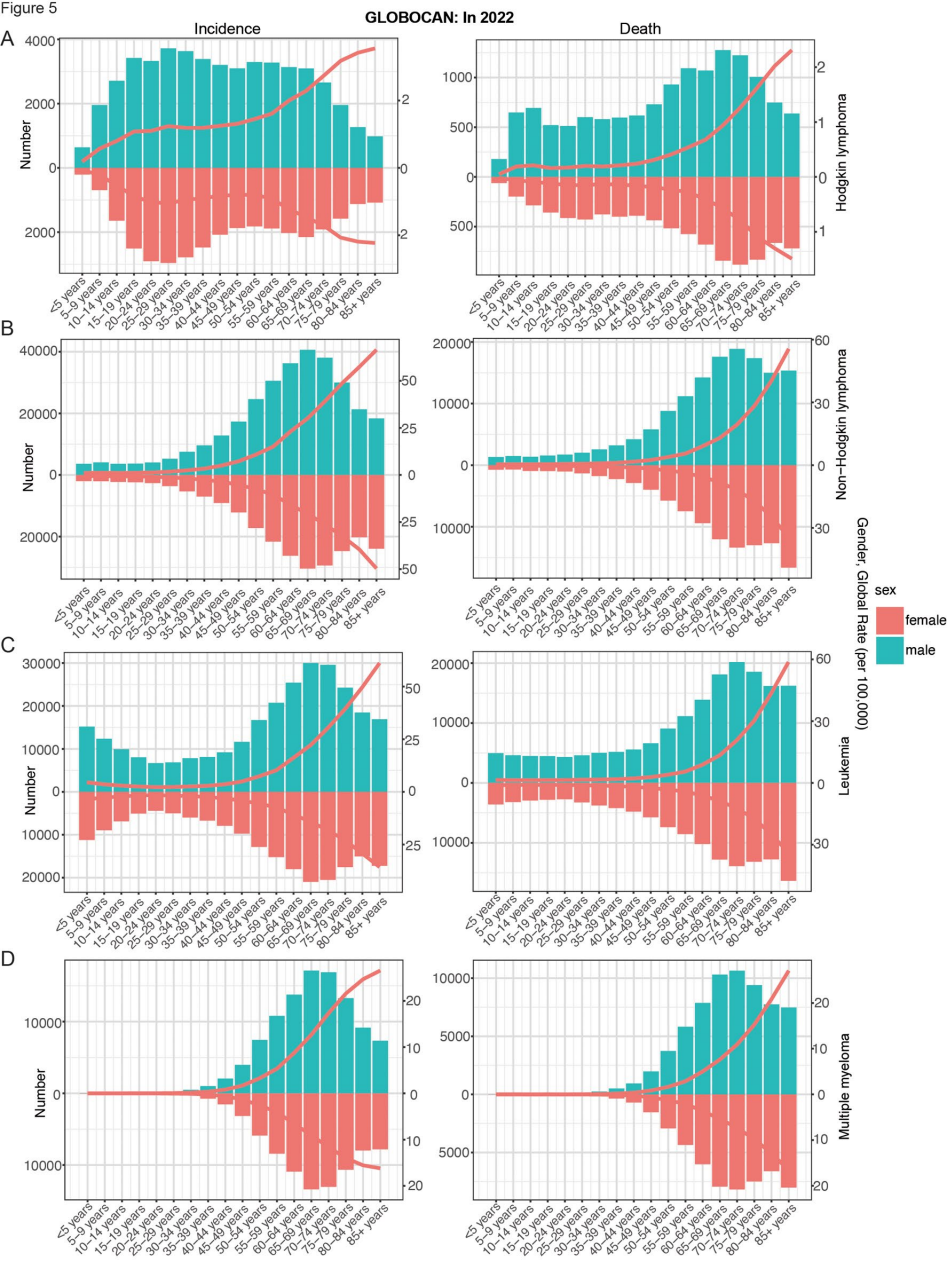
Distribution of incidence and deaths by gender and age group from GLOBOCAN 2022. (**A**): Hodgkin lymphoma, (**B**): Non − Hodgkin lymphoma, (**C**): Leukemia, (**D**): Multiple myeloma

**Fig. 6:**
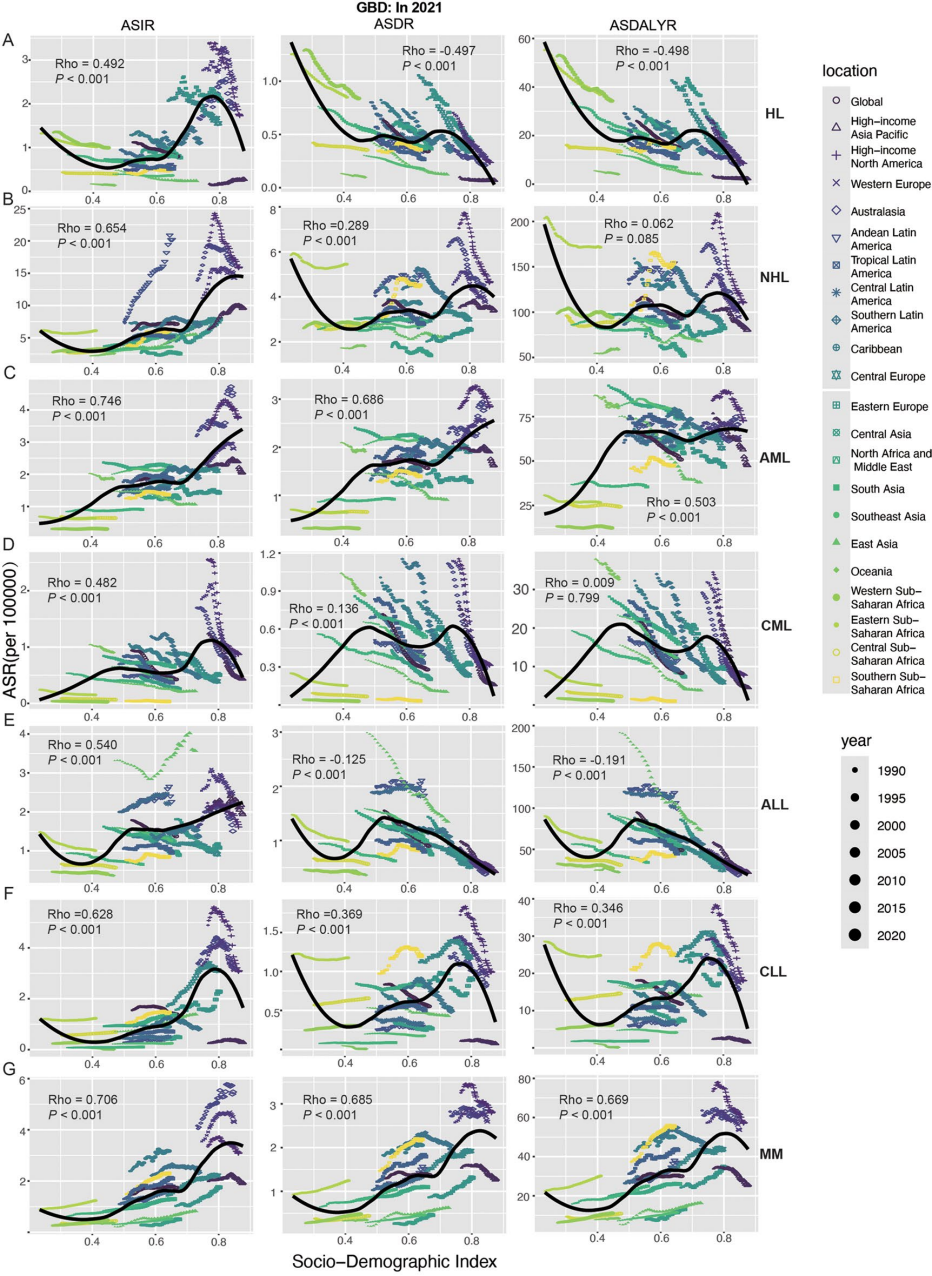
Global trends in ASIR, ASDR, ASDALYR and SDI in 1990–2021 from GBD. The Rho values indicate the strength of the correlation between the ASIR, ASDR, ASDALYR and SDI in HL (**A**), NHL (**B**), AML (**C**), CML (**D**), ALL (**E**), CLL (**F**), and MM (**G**). HL, Hodgkin lymphoma; NHL, Non − Hodgkin lymphoma; AML, Acute myeloid leukemia; CML, Chronic myeloid leukemia; ALL, Acute lymphoid leukemia; CLL, Chronic lymphoid leukemia; MM, Multiple myeloma; DALYs, disability-adjusted life years; ASPR, age-standardized prevalence rate; ASIR, age-standardized incidence rate; ASDR, age-standardized death rate; ASDALYR, age-standardized DALY rate; SDI, socio-demographic index

In both males and females, the prevalence, incidence, mortality, and DALYs of HL show two peaks, the first peak occurs between the ages of 20–34 years, the other peak occurs at the ages of 50–74 years, and the peak of mortality is slightly shifted backward; and the first peak of prevalence, incidence, and DALYs is significantly higher than the second peak, whereas the opposite is true for mortality, with the second peak being higher than the first peak (Figs. 4A and 5A; Figure S13A). This pattern indicates that while the 20–34 year age group exhibits a higher prevalence of HL, the 50–74 year age group faces elevated mortality rates, likely attributable to age-related comorbidities and reduced treatment tolerance. However, the younger age group bears a disproportionately higher burden of DALYs, reflecting the substantial life-years lost due to early-onset disease. It was also found that lower SDI was associated with higher mortality and DALYs (Fig. 4A; Figure S13A). Males consistently show higher incidence, mortality, and DALYs rates than females (Fig. 4A; Figure S13A). Furthermore, SDI showed a statistically significant positive correlation with ASPR and ASIR (ASPR: ρ = 0.655, *p* < 0.001; ASIR: ρ = 0.492, *p* < 0.001); on the contrary, SDI showed a statistically significant negative correlation with ASDR and ASDALYR (ASPR: ρ = -0.497, *p* < 0.001; ASIR: ρ = -0.498, *p* < 0.001) (Fig. 6A; Figure S14A). For future projections, we analyzed ASIR and ASDALYR for HL in 2022–2040, both of which showed a downward trend and were further validated in men and women (Fig. 8A; Figure S15A).

### Non − Hodgkin lymphoma burden

Among hematologic malignancies, NHL has the highest global burden in terms of prevalence, morbidity, mortality, and DALYs (Fig. 1). Between 1990 and 2021, from GBD 2021, global prevalent cases nearly tripled from 1.04 to 2.92 million and the ASPR increased from 22.30 to 34.33 (EAPC = 1.24) (Table S6). Global NHL incident cases increased from 0.26 million in 1990 to 0.60 million in 2021; ASIR rose from 6.08 to 7.14 (EAPC = 0.31) (Table 2). According to the GLOBOCAN 2022 database, in 2022, the number of incident cases of NHL globally was 553,389, and the ASIR was 5.57 (Fig. 1I and J). In 2021, from GBD 2021, high SDI countries had the highest number of new incident cases (0.25 million); and high SDI countries had the highest ASIR (12.96) (Fig. 2B; Fig. S1B; Table 2). Among the 21 global regions, East Asia had the highest incidence (0.11 million) and Andean Latin America ASIR was the highest (20.20) (Table 2). At the 204 countries level, China had the highest number of new cases (0.11 million), followed by the United States (86,980) and India (38,656); Peru (24.00), Slovenia (23.82), and Iceland (23.81) ranked in the top three ASIRs (Figs. 3B, S7B; Table S9). According to the GLOBOCAN 2022 database, in 2022, Malta (14.58), Denmark (14.00), and Israel (13.89) ranked the top three in ASIR (Fig. 3B).

**Table 2:** The incidence of NHL in 1990 and 2021. Annotations: ASIR: Age-standardized incidence rate (per 100,000 population); EAPC: estimated annual percentage change; SDI: Socio-demographic index

Location	1990 incident cases (95% CI)	1990 ASIR (95% CI)	2021 incident cases (95% CI)	2021 ASIR (95% CI)	EAPC (95% CI)
Global	255,668 (242749–272801)	6.08 (5.76–6.48)	604,554 (558229–648746)	7.14 (6.58–7.66)	0.31 (0.17 to 0.44)
High SDI	125,897 (121016–128830)	11.86 (11.43–12.14)	247,508 (225875–263944)	12.96 (12.03–13.68)	-0.07 (-0.32 to 0.18)
High-middle SDI	52,907 (50219–56507)	5.18 (4.91–5.53)	135,074 (121911–148400)	7.39 (6.68–8.09)	1.12 (1.02 to 1.21)
Middle SDI	41,317 (37439–46864)	3.31 (3.04–3.77)	137,684 (121797–154465)	5.12 (4.53–5.73)	1.42 (1.32 to 1.53)
Low-middle SDI	22,203 (19093–26688)	2.81 (2.43–3.39)	56,154 (49729–69679)	3.54 (3.15–4.41)	0.66 (0.61 to 0.71)
Low SDI	13,098 (10295–15849)	3.94 (3.18–4.71)	27,605 (22738–33544)	4 (3.41–4.74)	-0.13 (-0.24 to -0.01)
Andean Latin America	1841 (1626–2155)	7.49 (6.6–8.77)	12,332 (9807–15421)	20.2 (16.13–25.26)	3.32 (3.16 to 3.49)
Australasia	3184 (3005–3363)	13.87 (13.09–14.66)	8214 (7193–9252)	16.63 (14.74–18.52)	0.28 (-0.06 to 0.62)
Caribbean	2119 (1981–2287)	7.3 (6.85–7.83)	3845 (3397–4351)	7.37 (6.49–8.33)	0.14 (-0.02 to 0.3)
Central Asia	1580 (1472–1706)	2.67 (2.51–2.86)	2320 (2045–2624)	2.53 (2.24–2.85)	-0.28 (-0.52 to -0.04)
Central Europe	6056 (5826–6346)	4.23 (4.07–4.43)	14,580 (13385–15838)	7.55 (6.93–8.21)	1.92 (1.65 to 2.19)
Central Latin America	4043 (3938–4158)	3.83 (3.72–3.93)	15,417 (13749–17010)	6.06 (5.41–6.69)	1.26 (1.14 to 1.37)
Central Sub-Saharan Africa	932 (676–1287)	2.95 (2.12–4.1)	2328 (1691–3142)	3.17 (2.3–4.41)	0.2 (0.02 to 0.37)
East Asia	32,517 (28166–39418)	3.33 (2.89–4.03)	114,587 (90510–139098)	5.52 (4.39–6.66)	1.84 (1.57 to 2.11)
Eastern Europe	11,531 (11176–12000)	4.46 (4.33–4.62)	24,488 (22698–26352)	7.89 (7.33–8.46)	1.89 (1.49 to 2.29)
Eastern Sub-Saharan Africa	6947 (5485–8366)	6.06 (4.85–7.17)	14,567 (11598–18628)	6.11 (5.04–7.57)	-0.17 (-0.27 to -0.07)
High-income Asia Pacific	12,447 (11655–13239)	6.3 (5.9–6.69)	41,407 (35512–47168)	9.62 (8.49–10.81)	1.32 (1.06 to 1.57)
High-income North America	64,908 (61881–66927)	19.26 (18.45–19.83)	97,865 (89281–103520)	15.95 (14.69–16.78)	-1.18 (-1.46 to -0.89)
North Africa and Middle East	8156 (6886–10107)	3.69 (3.1–4.55)	27,801 (23912–34337)	5.39 (4.61–6.63)	1.34 (1.28 to 1.39)
Oceania	97 (75–131)	2.2 (1.74–2.94)	272 (201–353)	2.57 (1.97–3.3)	0.56 (0.53 to 0.59)
South Asia	20,483 (17352–24075)	2.77 (2.33–3.26)	54,304 (48059–65017)	3.42 (3.02–4.1)	0.51 (0.4 to 0.62)
Southeast Asia	8862 (7595–11267)	2.8 (2.44–3.58)	24,885 (21291–33269)	3.63 (3.12–4.86)	0.66 (0.58 to 0.74)
Southern Latin America	2758 (2616–2905)	5.85 (5.54–6.16)	5632 (5103–6088)	6.84 (6.18–7.39)	0.49 (0.15 to 0.82)
Southern Sub-Saharan Africa	1345 (1186–1535)	3.79 (3.32–4.34)	4247 (3372–4831)	6.15 (4.89–6.96)	1.81 (1.48 to 2.15)
Tropical Latin America	4577 (4423–4732)	4.21 (4.05–4.36)	13,235 (12415–13940)	5.21 (4.88–5.49)	0.45 (0.22 to 0.68)
Western Europe	57,414 (54924–59484)	11.16 (10.7-11.54)	112,417 (102315–121619)	14.27 (13.29–15.22)	0.53 (0.23 to 0.84)
Western Sub-Saharan Africa	3868 (3049–4664)	2.76 (2.22–3.24)	9810 (6994–12205)	3.26 (2.43–3.87)	0.45 (0.37 to 0.52)

In GBD 2021, the global number of NHL deaths increased from 0.15 million in 1990 to 0.27 million in 2021, while the ASDR decreased from 3.61 to 3.19 (EAPC=-0.59) (Table S7). According to the GLOBOCAN 2022 database, in 2022, the number of deaths of NHL globally was 250,679, and the ASDR was 2.38 (Fig. 1I and J). In 2021, from GBD 2021, high SDI countries had the highest number of deaths and ASDR of 86,990 and 3.97, respectively (Fig. 2B; Figure S1B; Table S7). Among the 21 global regions, East Asia had the highest number of deaths (45,118); while Eastern Sub-Saharan Africa had the highest ASDR (5.45) (Table S7). At the 204 countries level, China had the highest number of deaths (42,857), followed by the United States (28,057) and India (27,651); whereas Malawi (13.00) had the highest ASDR, followed by Grenada (10.00) and Zimbabwe (9.46) (Figs. 3B, S7C; Table S9). According to the GLOBOCAN 2022 database, in 2022, Zimbabwe (7.38), Uganda (5.5), and Egypt (5.16) ranked the top three in ASDR (Fig. 3B).

Between 1990 and 2021, from GBD 2021, global NHL DALYs decreased from 5.20 million to 7.77 million and ASDALYR decreased from 26.05 to 14.82 (EAPC =-0.76) (Table S8). In 2021, from GBD 2021, DALYs were highest in middle-SDI countries (2.11 million), while ASDALYR was highest in low-SDI countries (113.87) (Fig. 2B; Figure S1B; Table S8). Among the 21 global regions, East Asia DALYs were the highest (1.34 million); while Eastern Sub-Saharan Africa had the highest ASDALYR (171.59) (Table S8). At the 204 countries level, China had the highest DALYs (1.28 million), followed by India (0.88 million) and the United States (0.60 million); while Malawi (426.06) had the highest ASDALYR, followed by Grenada (315.66) and Zimbabwe (305.15) (Figures S7D, S7F; Table S9).

**Fig. 7:**
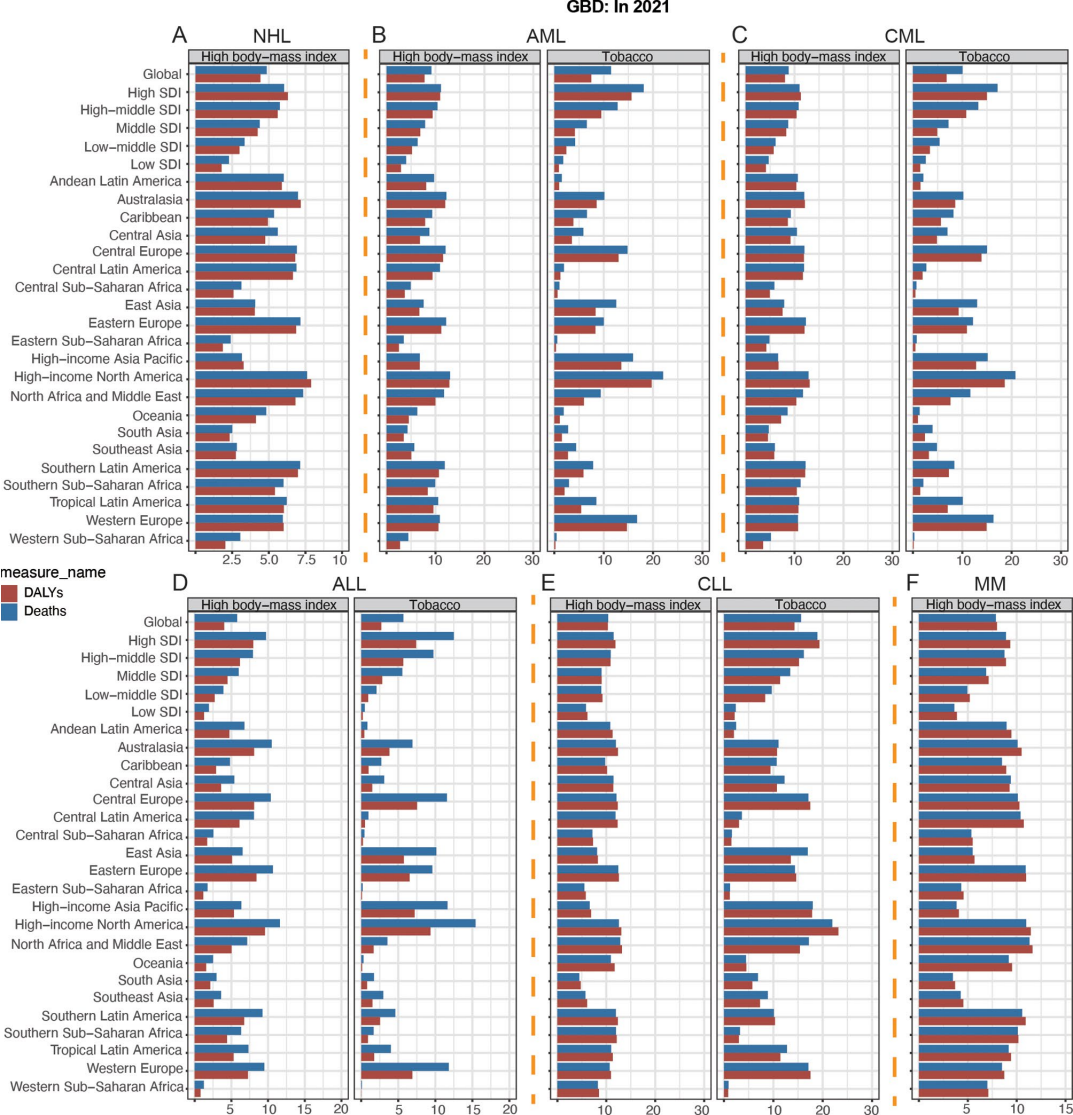
The proportions of risk factors contributing patients with deaths and DALYs vary across the 27 global regions in 2021 from GBD. (**A**): HL, (**B**): NHL, (**C**): AML, (**D**): CML, (**E**): ALL, (**F**): CLL, (**G**): MM. HL, Hodgkin lymphoma; NHL, Non − Hodgkin lymphoma; AML, Acute myeloid leukemia; CML, Chronic myeloid leukemia; ALL, Acute lymphoid leukemia; CLL, Chronic lymphoid leukemia; MM, Multiple myeloma; DALYs, disability-adjusted life years; SDI, socio-demographic index

**Fig. 8:**
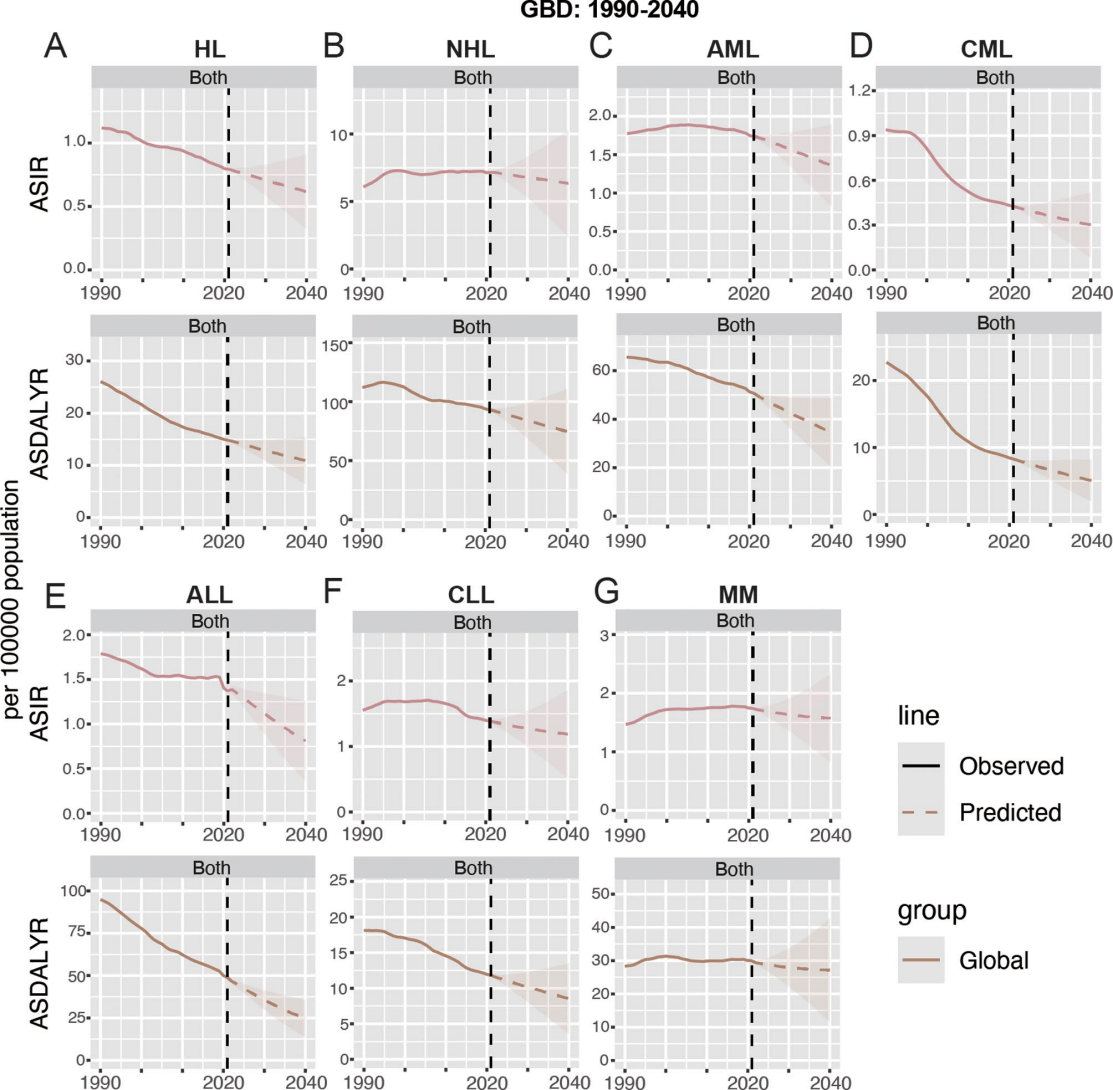
Future forecasts of GBD in ASIR and ASDALYR in 2021–2040. (**A**): HL, (**B**): NHL, (**C**): AML, (**D**): CML, (**E**): ALL, (**F**): CLL, (**G**): MM. HL, Hodgkin lymphoma; NHL, Non − Hodgkin lymphoma; AML, Acute myeloid leukemia; CML, Chronic myeloid leukemia; ALL, Acute lymphoid leukemia; CLL, Chronic lymphoid leukemia; MM, Multiple myeloma; ASIR, age-standardized incidence rate; ASDALYR, age-standardized DALY rate

For both males and females, NHL prevalence, morbidity, mortality, and DALYs peaked at 65–74 years, with additional small peaks at prevalence (30–39 years) and DALYs (0–9 years) (Figs. 4B and 5B; Figure S13B). Meanwhile, higher SDI had higher prevalence and morbidity (Fig. 4B, Figure S13B). In addition, SDI was statistically positively correlated with ASPR, ASIR, and ASDR (ASPR: ρ = 0.729, *p* < 0.001; ASIR: ρ = 0.654, *p* < 0.001; ASDR: ρ = 0.289, *p* < 0.001) (Fig. 6B, Figure S14B). Globally, the contribution of high BMI to NHL mortality and DALYs was greater and progressively increased with increasing levels of SDI (Fig. 7A). For future projections, we analyzed the ASIR and ASDALYR of NHL in 2022–2040, both of which showed a slow downward trend and were further validated in both men and women (Fig. 8B, Figure S15B).

### Acute myeloid leukemia burden

Between 1990 and 2021, from GBD 2021, the global prevalence of AML increased from 0.12 to 0.20 million cases, whereas the ASPR remained essentially flat, decreasing from 2.52 to 2.50 (EAPC =-0.01) (Table S10). While global AML incidence increased from 79,373 in 1990 to 144,646 in 2021; and ASIR decreased from 1.77 to 1.73 (EAPC=-0.03) (Table 3). In 2021, from GBD 2021, high SDI countries had the highest number of new incident cases and ASIR at 56,646 and 2.88, respectively (Fig. 2C; Figure S1C; Table 3). Among the 21 global regions, Western Europe had the highest incidence (24,638) and Australasia had the highest ASIR (4.53) (Table 3). At the 204 countries level, the United States had the highest number of new cases (21,533), followed by China (17,835) and India (11,040); Monaco (6.30), Australia (4.92), and Fiji (4.15) had the top three ASIRs (Fig. 3C, Figure S8B, Table S13).

**Table 3:** The incidence of AML in 1990 and 2021. Annotations: ASIR: Age-standardized incidence rate (per 100,000 population); EAPC: estimated annual percentage change; SDI: Socio-demographic index

Location	1990 incident cases (95% CI)	1990 ASIR (95% CI)	2021 incident cases (95% CI)	2021 ASIR (95% CI)	EAPC (95% CI)
Global	79,373 (62806–99429)	1.77 (1.43–2.16)	144,646 (126237–164851)	1.73 (1.51–1.98)	-0.03 (-0.12 to 0.06)
High SDI	27,831 (26575–28799)	2.65 (2.54–2.75)	56,646 (51611–59717)	2.88 (2.66–3.02)	0.41 (0.25 to 0.56)
High-middle SDI	18,663 (14114–22745)	1.83 (1.39–2.23)	29,327 (24602–33130)	1.69 (1.4–1.92)	-0.31 (-0.38 to -0.23)
Middle SDI	20,168 (13540–29687)	1.41 (0.98–2.01)	33,430 (27392–41622)	1.29 (1.06–1.61)	-0.4 (-0.46 to -0.34)
Low-middle SDI	9631 (6242–15986)	1.06 (0.71–1.62)	19,062 (14795–25945)	1.17 (0.92–1.6)	0.34 (0.31 to 0.38)
Low SDI	2992 (1361–5453)	0.81 (0.41–1.25)	6027 (3673–7907)	0.79 (0.5–1.04)	-0.1 (-0.17 to -0.04)
Andean Latin America	452 (326–649)	1.49 (1.12–2.07)	1067 (744–1343)	1.72 (1.2–2.17)	0.8 (0.67 to 0.92)
Australasia	730 (682–782)	3.19 (2.98–3.41)	2333 (2074–2584)	4.53 (4.06–5.02)	0.95 (0.69 to 1.21)
Caribbean	495 (430–617)	1.59 (1.41–1.91)	841 (713–992)	1.65 (1.39–1.99)	0.31 (0.2 to 0.42)
Central Asia	861 (741–999)	1.33 (1.16–1.54)	1155 (1007–1352)	1.25 (1.09–1.46)	-0.05 (-0.15 to 0.06)
Central Europe	2516 (2348–2712)	1.78 (1.66–1.91)	4235 (3853–4595)	2.11 (1.92–2.29)	0.92 (0.78 to 1.07)
Central Latin America	1779 (1706–1860)	1.32 (1.27–1.37)	3946 (3515–4405)	1.56 (1.39–1.74)	0.4 (0.31 to 0.49)
Central Sub-Saharan Africa	250 (125–432)	0.69 (0.35–1.01)	542 (298–750)	0.64 (0.37–0.91)	-0.23 (-0.32 to -0.13)
East Asia	15,919 (8702–24824)	1.47 (0.82–2.23)	19,157 (13181–26301)	1.07 (0.74–1.48)	-1.39 (-1.57 to -1.2)
Eastern Europe	3825 (3528–4210)	1.54 (1.43–1.69)	4278 (3918–4654)	1.43 (1.32–1.55)	-0.3 (-0.46 to -0.13)
Eastern Sub-Saharan Africa	1011 (439–1819)	0.68 (0.32–1.05)	1882 (1009–2672)	0.63 (0.36–0.89)	-0.33 (-0.39 to -0.26)
High-income Asia Pacific	4401 (4143–4754)	2.28 (2.14–2.46)	8361 (7252–9141)	2.05 (1.81–2.22)	-0.09 (-0.27 to 0.1)
High-income North America	11,533 (10888–11876)	3.38 (3.21–3.48)	23,676 (21534–24730)	3.77 (3.47–3.92)	0.42 (0.19 to 0.65)
North Africa and Middle East	5643 (3744–8176)	2.37 (1.62–3.26)	11,358 (8396–15192)	2.22 (1.66-3)	-0.07 (-0.15 to 0.02)
Oceania	96 (47–134)	2.02 (0.98–2.81)	198 (104–281)	1.85 (0.95–2.63)	-0.28 (-0.35 to -0.21)
South Asia	7186 (4452–11930)	0.84 (0.54–1.29)	14,666 (11306–20277)	0.91 (0.68–1.25)	0.15 (0.08 to 0.23)
Southeast Asia	6958 (4746–10405)	2 (1.35–2.82)	14,235 (10093–17323)	2.13 (1.52–2.59)	0.12 (0.05 to 0.18)
Southern Latin America	873 (809–944)	1.82 (1.69–1.97)	1530 (1419–1665)	1.88 (1.75–2.05)	0.34 (0.12 to 0.56)
Southern Sub-Saharan Africa	440 (280–614)	1.25 (0.81–1.75)	889 (612–1236)	1.37 (0.92–1.88)	0.36 (0.2 to 0.52)
Tropical Latin America	2140 (2069–2218)	1.76 (1.7–1.82)	4631 (4337–4863)	1.86 (1.74–1.96)	0.3 (0.17 to 0.42)
Western Europe	11,791 (11278–12196)	2.25 (2.17–2.32)	24,638 (22110–26159)	2.79 (2.57–2.93)	0.87 (0.74 to 1.01)
Western Sub-Saharan Africa	473 (227–869)	0.3 (0.16–0.45)	1026 (578–1353)	0.3 (0.19–0.38)	0.04 (-0.03 to 0.11)

In GBD 2021, the global number of AML deaths increased from 74,918 in 1990 to 130,189 in 2021, whereas the ASDR decreased from 1.69 to 1.57 (EAPC=-0.21) (Table S11). In 2021, from GBD 2021, High-SDI countries had the highest number of deaths and ASDR, 47,189 and 2.31, respectively (Fig. 2C; Figure S1C; Table S11). Among the 21 global regions, Western Europe had the highest number of deaths (22,485); and High-income North America had the highest ASDR (2.85) (Table S11). At the 204 countries level, the United States had the highest number of deaths (16,648), followed by China (15,311) and India (10,981); whereas Monaco (5.34) had the highest ASDR, followed by Fiji (4.27) and Afghanistan (3.94) (Figs. 3C, Figure S8C; Table S13).

Between 1990 and 2021, from GBD 2021, global AML DALYs increased from 3.34 to 4.14 million, but ASDALYR decreased from 65.55 to 50.79 (EAPC =-0.84) (Table S12). In 2021, from GBD 2021, DALYs were highest in the medium SDI countries (1.19 million), whereas ASDALYR was highest in the high SDI countries (62.30) (Fig. 2C; Figure S1C; Table S12). Among the 21 global regions, South Asia DALYs were the highest (0.62 million); while Oceania had the highest ASDALYR (79.86) (Table S12). At the country level, China had the highest DALYs (0.55 million), followed by India (0.44 million) and the United States (0.37 million); while Tokelau (204.88) had the highest ASDALYR, followed by Niue (169.15) and Fiji (167.10) (Figures S8D, S8F; Table S13).

AML prevalence, incidence, deaths, and DALYs peaked at 65–79 years and all had a small peak at 0–5 years. In addition, the prevalence of AML showed a second peak at 30–34 (Fig. 4C, Figure S13C). We also found that SDI was statistically positively correlated with ASPR, ASIR, ASDR and ASDALYR (ASPR: ρ = 0.736, *p* < 0.001; ASIR: ρ = 0.746, *p* < 0.001; ASDR: ρ = 0.686, *p* < 0.001; ASDALYR: ρ = 0.503, *p* < 0.001) (Fig. 6C, Figure S14C). Globally, smoking contributed more to AML mortality and DALYs than high BMI; and the contribution of high BMI and smoking to AML mortality and DALYs increased progressively with increasing levels of SDI; among the 21 global regions, high BMI and smoking in High-income North America had the had the greatest impact (Fig. 7B). For the 2040 projections, the ASIR and ASDALYR for AML tended to be downwardly adjusted for both males and females (Fig. 8C, Figure S15C).

### Chronic myeloid leukemia burden

Between 1990 and 2021, from GBD 2021, CML global prevalent cases increased from 57,606 to 63,728, while the ASPR decreased from 1.33 to 0.75 (EAPC =-2.43) (Table S14). The global number of CML incidence decreased from 39,482 in 1990 to 35,831 in 2021; and the ASIR declined by about half, from 0.94 to 0.43 (EAPC=-3.03) (Table 4). In 2021, from GBD 2021, High-SDI countries had the highest number of new incident cases and ASIR, at 12,806 and 0.69, respectively (Fig. 2D; Fig. S1D; Table 4). Among the 21 global regions, Western Europe had the highest incidence (6,757) and High-income North America had the highest ASIR (0.87) (Table 4). At the 204 countries level, the United States had the highest number of new cases (4,611), followed by India (4,473) and China (3,850); Iraq (2.88), Principality of Monaco (1.81), and Republic of Nauru (1,80) ranked among the top three ASIRs (Fig. 3D; Figure S9B; Table S17).

**Table 4:** The incidence of CML in 1990 and 2021. Annotations: ASIR: Age-standardized incidence rate (per 100,000 population); EAPC: estimated annual percentage change; SDI: Socio-demographic index

Location	1990 incident cases (95% CI)	1990 ASIR (95% CI)	2021 incident cases (95% CI)	2021 ASIR (95% CI)	EAPC (95% CI)
Global	39,482 (33293–45117)	0.94 (0.81–1.07)	35,831 (27939–42581)	0.43 (0.33–0.51)	-3.03 (-3.23 to -2.84)
High SDI	19,286 (18201–20679)	1.82 (1.72–1.95)	12,806 (11654–13983)	0.69 (0.64–0.75)	-3.77 (-4.07 to -3.47)
High-middle SDI	8439 (6968–9966)	0.84 (0.7–0.99)	7395 (5719–8847)	0.41 (0.31–0.49)	-2.94 (-3.22 to -2.67)
Middle SDI	6283 (4115–7822)	0.49 (0.33–0.61)	8237 (5395–10946)	0.31 (0.21–0.41)	-1.7 (-1.81 to -1.59)
Low-middle SDI	4236 (2666–5885)	0.54 (0.36–0.76)	5679 (3638–8179)	0.37 (0.24–0.53)	-1.27 (-1.31 to -1.22)
Low SDI	1193 (628–1842)	0.38 (0.22–0.64)	1672 (953–2700)	0.26 (0.15–0.44)	-1.31 (-1.4 to -1.22)
Andean Latin America	129 (89–194)	0.5 (0.35–0.71)	278 (174–375)	0.45 (0.28–0.61)	-0.11 (-0.23 to 0.01)
Australasia	390 (361–419)	1.7 (1.57–1.83)	305 (262–353)	0.64 (0.55–0.74)	-3.49 (-3.62 to -3.36)
Caribbean	295 (243–367)	1.05 (0.89–1.25)	386 (322–451)	0.73 (0.61–0.87)	-1.1 (-1.22 to -0.98)
Central Asia	263 (222–320)	0.47 (0.39–0.58)	227 (179–306)	0.25 (0.2–0.34)	-2.09 (-2.19 to -1.98)
Central Europe	1291 (1127–1518)	0.9 (0.78–1.05)	793 (699–908)	0.39 (0.35–0.45)	-3.21 (-3.56 to -2.87)
Central Latin America	737 (697–785)	0.7 (0.66–0.74)	1091 (972–1234)	0.43 (0.38–0.48)	-2.18 (-2.44 to -1.92)
Central Sub-Saharan Africa	32 (13–63)	0.09 (0.04–0.2)	51 (19–101)	0.07 (0.02–0.14)	-1 (-1.06 to -0.95)
East Asia	4070 (1948–5943)	0.38 (0.18–0.55)	4099 (2324–6410)	0.22 (0.12–0.34)	-2.23 (-2.4 to -2.05)
Eastern Europe	1867 (1446–2347)	0.7 (0.54–0.87)	1611 (1409–1866)	0.5 (0.44–0.58)	-1.72 (-2.11 to -1.32)
Eastern Sub-Saharan Africa	264 (131–515)	0.24 (0.12–0.49)	368 (158–826)	0.15 (0.07–0.34)	-1.82 (-1.92 to -1.73)
High-income Asia Pacific	2417 (2203–2636)	1.24 (1.13–1.35)	1501 (1264–1746)	0.43 (0.37–0.51)	-3.85 (-4.11 to -3.59)
High-income North America	8532 (8184–8874)	2.56 (2.45–2.66)	5065 (4709–5349)	0.87 (0.82–0.91)	-4.27 (-4.71 to -3.83)
North Africa and Middle East	1781 (1022–2547)	0.84 (0.52–1.26)	3213 (1606–4553)	0.65 (0.33–0.94)	-0.63 (-0.72 to -0.54)
Oceania	43 (23–68)	1.04 (0.59–1.6)	92 (52–136)	0.9 (0.53–1.32)	-0.49 (-0.51 to -0.46)
South Asia	3868 (2331–5586)	0.54 (0.33–0.77)	5708 (3330–8445)	0.37 (0.22–0.56)	-1.21 (-1.3 to -1.12)
Southeast Asia	2124 (1196–3030)	0.65 (0.35–0.93)	2854 (1578–3946)	0.42 (0.24–0.58)	-1.59 (-1.7 to -1.48)
Southern Latin America	551 (483–663)	1.19 (1.04–1.43)	390 (337–446)	0.46 (0.4–0.53)	-3.44 (-3.82 to -3.06)
Southern Sub-Saharan Africa	16 (6–30)	0.04 (0.02–0.09)	21 (10–38)	0.03 (0.02–0.06)	-0.97 (-1.39 to -0.54)
Tropical Latin America	890 (842–939)	0.84 (0.79–0.89)	929 (846–1028)	0.37 (0.34–0.41)	-3.23 (-3.58 to -2.88)
Western Europe	9862 (9172–10843)	1.85 (1.73–2.02)	6757 (6011–7602)	0.81 (0.72–0.9)	-3.42 (-3.75 to -3.1)
Western Sub-Saharan Africa	62 (24–127)	0.04 (0.02–0.08)	91 (42–177)	0.03 (0.01–0.06)	-1 (-1.07 to -0.94)

In GBD 2021, the global number of CML deaths decreased from 29,220 in 1990 to 23,159 in 2021, while the ASDR decreased from 0.72 to 0.28 (EAPC=-3.43) (Table S15). In 2021, from GBD 2021, the highest number of deaths was observed in countries with medium SDI (6,415), and the highest ASDR was observed in countries with low-medium SDI (0.37) (Fig. 2D; Figure S1D; Table S15). Among the 21 global regions, South Asia had the highest number of deaths (5,360); while Oceania had the highest ASDR (0.86) (Table S15). At the 204 countries level, India had the highest number of deaths (4,185), followed by the United States (1,846) and China (1,841); whereas Iraq (2.38) had the highest ASDR, followed by Republic of Nauru (1.70) and Samoa (1.63) (Fig. 3D; Figure S9C; Table S17).

Between 1990 and 2021, from GBD 2021, global CML DALYs decreased from 1.07 to 0.69 million, but ASDALYR decreased from 22.72 to 8.30 (EAPC = -3.62) (Table S16). In 2021, from GBD 2021, DALYs were highest in mid-SDI countries (0.21 million), while ASDALYR was highest in low-mid-SDI countries (10.93) (Fig. 2D; Figure S1D; Table S16). Among the 21 global regions, South Asia DALYs were the highest (0.18 million); while Oceania had the highest ASDALYR (30.61) (Table S16). At the country level, India had the highest DALYs (0.14 million), followed by China (60,929) and the United States (39,174); while Iraq (66.55) had the highest ASDALYR, followed by Republic of Nauru (59.85) and Samoa (50.13) (Figures S9D, S9F; Table S17).

The peak prevalence and incidence of CML occurred at 65–74 years, while the peak mortality of CML was slightly delayed (70–84 years); notably, the DALYs of CML showed three peaks at 0–5, 35–39, and 55–74 years, and lower SDI had higher DALYs (Fig. 4D, Figure S13D). We also found that SDI was statistically positively correlated with ASPR, ASIR, and ASDR (ASPR: ρ = 0.619, *p* < 0.001; ASIR: ρ = 0.482, *p* < 0.001; ASDR: ρ = 0.136, *p* < 0.001) (Fig. 6D, Figure S14D). Globally, smoking contributed more to CML mortality and DALYs than high BMI; and the contribution of high BMI and smoking to CML mortality and DALYs increased progressively with increasing levels of SDI; among the 21 global regions, high BMI and smoking in High-income North America had the had the greatest impact (Fig. 7C). For the 2040 projections, both ASIR and ASDALYR for CML showed a downward trend, which was validated in both men and women (Fig. 8D, Figure S15D).

### Acute lymphoid leukemia burden

Between 1990 and 2021, from GBD 2021, ALL global prevalent cases increased from 0.24 to 0.39 million and ASPR increased from 4.12 to 5.43 (EAPC = 1.40) (Table S18). The global number of ALL incidence was elevated from 98,149 in 1990 to 103,727 in 2021; whereas the ASIR decreased from 1.79 to 1.37 (EAPC=-0.64) (Table 5). In 2021, from GBD 2021, the highest number of new cases was found in countries with medium SDI (37,032), and the highest ASIR was found in countries with medium and high SDI (2.63) (Fig. 2E; Figure S1E; Table 5). Among the 21 global regions, East Asia had the highest incidence (39,300) and East Asia had the highest ASIR (3.57) (Table 5). At the 204 countries level, China had the highest number of new cases (38,571), followed by India (7,751) and the United States (4,369); and Monaco (8.81), San Marino (5.98), and China (3.64) ranked among the top three in terms of ASIR (Fig. 3E; Figure S10B; Table S21).

**Table 5:** The incidence of ALL in 1990 and 2021. Annotations: ASIR: Age-standardized incidence rate (per 100,000 population); EAPC: estimated annual percentage change; SDI: Socio-demographic index

Location	1990 incident cases (95% CI)	1990 ASIR (95% CI)	2021 incident cases (95% CI)	2021 ASIR (95% CI)	EAPC (95% CI)
Global	98,149 (77896–123508)	1.79 (1.42–2.24)	103,727 (71607–122517)	1.37 (0.95–1.64)	-0.64 (-0.74 to -0.54)
High SDI	15,131 (14589–15930)	1.99 (1.91–2.1)	14,604 (13253–15745)	1.78 (1.58–1.95)	-0.12 (-0.28 to 0.05)
High-middle SDI	25,273 (20189–30016)	2.5 (2-2.99)	26,547 (16506–33499)	2.63 (1.59–3.52)	0.58 (0.38 to 0.79)
Middle SDI	37,443 (27583–47980)	2.15 (1.6–2.72)	37,032 (24452–44677)	1.65 (1.09–2.02)	-0.64 (-0.8 to -0.48)
Low-middle SDI	13,702 (9610–19653)	1.1 (0.78–1.54)	15,862 (10874–19381)	0.85 (0.58–1.06)	-0.75 (-0.81 to -0.7)
Low SDI	6532 (3764–10836)	1.15 (0.74–1.84)	9618 (6036–12254)	0.83 (0.51–1.05)	-1.16 (-1.22 to -1.1)
Andean Latin America	836 (678–1128)	2.04 (1.65–2.73)	1447 (952–1842)	2.26 (1.49–2.88)	0.68 (0.57 to 0.79)
Australasia	300 (276–330)	1.62 (1.48–1.81)	372 (324–420)	1.5 (1.27–1.75)	0.19 (-0.21 to 0.59)
Caribbean	543 (418–749)	1.46 (1.13–1.99)	544 (417–749)	1.22 (0.92–1.7)	-0.37 (-0.46 to -0.28)
Central Asia	1069 (941–1200)	1.44 (1.28–1.61)	840 (709–984)	0.89 (0.75–1.04)	-1.62 (-1.74 to -1.5)
Central Europe	1557 (1457–1698)	1.29 (1.2–1.41)	978 (868–1095)	0.91 (0.79–1.05)	-0.87 (-1.01 to -0.72)
Central Latin America	3625 (3472–3801)	2 (1.92–2.08)	5423 (4848–6184)	2.2 (1.95–2.53)	0.63 (0.5 to 0.76)
Central Sub-Saharan Africa	395 (234–645)	0.66 (0.4–0.96)	745 (404–1015)	0.57 (0.31–0.83)	-0.3 (-0.38 to -0.23)
East Asia	38,593 (27220–51050)	3.32 (2.35–4.4)	39,300 (21672–51648)	3.57 (1.97–4.96)	0.64 (0.35 to 0.93)
Eastern Europe	3856 (3566–4122)	1.81 (1.67–1.94)	2089 (1943–2239)	1.08 (1-1.16)	-1.86 (-2.14 to -1.59)
Eastern Sub-Saharan Africa	3201 (1839–5289)	1.48 (0.93–2.38)	4489 (2861–6096)	1.01 (0.63–1.35)	-1.42 (-1.5 to -1.34)
High-income Asia Pacific	2968 (2693–3352)	2.07 (1.84–2.41)	2339 (2025–2623)	1.93 (1.63–2.21)	-0.04 (-0.26 to 0.18)
High-income North America	5557 (5365–5768)	2.23 (2.15–2.32)	4906 (4629–5183)	1.61 (1.5–1.73)	-0.76 (-0.96 to -0.55)
North Africa and Middle East	5930 (3789–8780)	1.62 (0.99–2.4)	7429 (4026–9319)	1.24 (0.67–1.54)	-0.54 (-0.65 to -0.44)
Oceania	29 (14–50)	0.41 (0.21–0.67)	54 (27–93)	0.36 (0.19–0.6)	-0.43 (-0.6 to -0.25)
South Asia	10,712 (7071–16023)	0.94 (0.65–1.35)	11,450 (7597–15733)	0.65 (0.43–0.9)	-1.26 (-1.33 to -1.19)
Southeast Asia	7343 (5156–10540)	1.6 (1.09–2.23)	8354 (5092–10529)	1.27 (0.79–1.6)	-0.74 (-0.8 to -0.68)
Southern Latin America	713 (659–765)	1.43 (1.32–1.53)	874 (797–958)	1.36 (1.22–1.53)	0.35 (0.18 to 0.52)
Southern Sub-Saharan Africa	378 (264–498)	0.76 (0.51–1.02)	638 (375–798)	0.84 (0.49–1.06)	0.52 (0.29 to 0.76)
Tropical Latin America	1663 (1543–1781)	1.05 (0.99–1.12)	2046 (1895–2216)	0.95 (0.87–1.04)	-0.02 (-0.2 to 0.16)
Western Europe	7668 (7333–8012)	2.54 (2.41–2.7)	6870 (6434–7418)	2.22 (2.05–2.44)	-0.46 (-0.72 to -0.19)
Western Sub-Saharan Africa	1211 (624–1800)	0.46 (0.25–0.66)	2540 (1108–3593)	0.41 (0.19–0.56)	-0.23 (-0.32 to -0.15)

In GBD 2021, the global number of ALL deaths decreased from 82,769 in 1990 to 71,221 in 2021, whereas the ASDR decreased from 1.55 to 0.90 (EAPC = -1.73) (Table S19). In 2021, from GBD 2021, the highest number of deaths (26,640) and the highest ASDR (1.09) were observed in countries with mid-SDI (Fig. 2E; Figure S1E; Table S19). Among the 21 global regions, South Asia had the highest number of deaths (21,208); while Andean Latin America had the highest ASDR (1.83) (Table S19). At the country level, China had the highest number of deaths (20,613), followed by India (7,323) and Indonesia (3,163); while Afghanistan (2.94) had the highest ASDR followed by Bolivia (Plurinational State of) (2.24) and Ecuador (2.16) (Fig. 3E; Figure S10C; Table S21).

Between 1990 and 2021, from GBD 2021, global ALL DALYs decreased from 5.46 to 3.72 million and ASDALYR decreased from 94.89 to 48.86 (EAPC =-2.11) (Table S20). In 2021, from GBD 2021, DALYs were highest in the middle SDI countries (1.36 million) and ASDALYR was highest in the middle SDI countries (58.79) (Fig. 2E; Figure S1E; Table S20). Among the 21 global regions, South Asia had the highest DALYs (0.95 million); and Central Latin America had the highest ASDALYR (101.43) (Table S20). At the country level, China had the highest DALYs (0.92 million), followed by India (0.40 million) and Indonesia (0.18 million); while Afghanistan (148.05) had the highest ASDALYR, followed by Bolivia (Plurinational State of) (122.80) and Haiti (115.62) (Figure S10D, S10F; Table S21).

The peak prevalence and incidence of ALL occurred at 0–5 years of age, while the peak mortality of ALL was slightly delayed (5–9 years of age), and there was also a small peak in ALL mortality at 65–69; and lower SDI had higher DALYs (Fig. 4E, Figure S13E). We also found that SDI was statistically positively associated with ASPR and ASIR (ASPR: ρ = 0.725, *p* < 0.001; ASIR: ρ = 0.540, *p* < 0.001); and statistically negatively associated with ASDR and ASDALYR (ASDR: ρ = -0.125, *p* < 0.001; ASDALYR: ρ = -0.191, *p* < 0.001). (Fig. 6E, Figure S14E). Globally, smoking contributed more to ALL mortality and DALYs than high BMI; and the contribution of high BMI and smoking to ALL mortality and DALYs increased progressively with increasing levels of SDI; among the 21 global regions, high BMI and smoking in High-income North America had the had the greatest impact (Fig. 7D). For the 2040 projections, there was a significant downward trend in both ASIR and ASDALYR for ALL, which was validated in both men and women (Fig. 8E, Figure S15E).

### Chronic lymphoid leukemia burden

Between 1990 and 2021, from GBD 2021, the global prevalence of CLL more than doubled from 0.31 to 0.72 million cases and the ASPR increased from 7.72 to 8.34 (EAPC = 0.15) (Table S22). Global CLL incidence nearly doubled from 57,961 in 1990 to 117,987 in 2021; whereas ASIR decreased from 1.55 to 1.39 (EAPC=-0.47) (Table 6). In 2021, from GBD 2021, high SDI countries had the highest number of new incident cases and ASIR, 52,424 and 2.38, respectively (Fig. 2F; Figure S1F; Table 6). Among the 21 global regions, Western Europe had the highest incidence (31,227) and Australasia had the highest ASIR (3.31) (Table 6). At the 204 countries level, China had the highest number of new cases (28,927), followed by the United States (18,898) and Germany (6,497); Principality of Monaco (6.94), Slovenia (6.54), and Estonia (4.90) had the top three ASIRs (Fig. 3F; Figure S11B; Table S25).

**Table 6:** The incidence of CLL in 1990 and 2021. Annotations: ASIR: Age-standardized incidence rate (per 100,000 population); EAPC: estimated annual percentage change; SDI: Socio-demographic index

Location	1990 incident cases (95% CI)	1990 ASIR (95% CI)	2021 incident cases (95% CI)	2021 ASIR (95% CI)	EAPC (95% CI)
Global	57,961 (53100–61681)	1.55 (1.41–1.64)	117,987 (98330–132718)	1.39 (1.16–1.56)	-0.47 (-0.67 to -0.28)
High SDI	34,575 (32535–35902)	3.08 (2.9–3.2)	52,424 (47119–55939)	2.38 (2.16–2.52)	-0.98 (-1.31 to -0.64)
High-middle SDI	15,012 (13723–16363)	1.53 (1.4–1.67)	37,237 (30038–43675)	1.91 (1.54–2.25)	0.64 (0.5 to 0.78)
Middle SDI	6046 (3631–7498)	0.55 (0.34–0.67)	21,629 (13984–28515)	0.8 (0.52–1.05)	1.32 (1.26 to 1.37)
Low-middle SDI	1127 (627–1421)	0.2 (0.11–0.25)	3949 (2143–4957)	0.28 (0.16–0.35)	1.21 (1.16 to 1.26)
Low SDI	1132 (639–1500)	0.54 (0.31–0.71)	2591 (1326–3644)	0.56 (0.29–0.79)	0.02 (-0.08 to 0.11)
Andean Latin America	56 (42–78)	0.27 (0.21–0.38)	262 (167–351)	0.44 (0.28–0.59)	1.79 (1.58 to 2.01)
Australasia	806 (742–878)	3.39 (3.11–3.69)	1852 (1577–2139)	3.31 (2.81–3.84)	-0.01 (-0.37 to 0.35)
Caribbean	244 (224–267)	0.96 (0.88–1.05)	487 (414–558)	0.9 (0.77–1.03)	-0.09 (-0.29 to 0.11)
Central Asia	275 (226–319)	0.55 (0.45–0.64)	415 (340–515)	0.48 (0.4–0.6)	-0.46 (-0.71 to -0.21)
Central Europe	2577 (2388–2799)	1.72 (1.59–1.86)	7064 (6347–7747)	3.11 (2.8–3.43)	2.24 (1.87 to 2.6)
Central Latin America	348 (329–366)	0.43 (0.41–0.46)	1240 (1101–1395)	0.5 (0.45–0.57)	0.39 (0.21 to 0.57)
Central Sub-Saharan Africa	112 (52–157)	0.55 (0.27–0.82)	350 (132–535)	0.69 (0.27–1.09)	0.75 (0.67 to 0.83)
East Asia	6894 (4069–9291)	0.7 (0.42–0.96)	29,368 (18318–41148)	1.39 (0.87–1.95)	2.53 (2.43 to 2.64)
Eastern Europe	4472 (4027–4906)	1.57 (1.41–1.72)	8035 (7351–8760)	2.28 (2.08–2.49)	0.66 (0.27 to 1.06)
Eastern Sub-Saharan Africa	844 (535–1146)	1.19 (0.78–1.63)	1886 (1017–2757)	1.23 (0.68–1.78)	-0.03 (-0.11 to 0.04)
High-income Asia Pacific	517 (471–572)	0.26 (0.23–0.28)	1358 (1139–1557)	0.29 (0.24–0.34)	0.29 (-0.17 to 0.75)
High-income North America	17,577 (16491–18206)	4.89 (4.6–5.06)	21,609 (19410–22880)	3.1 (2.8–3.27)	-1.76 (-2.19 to -1.33)
North Africa and Middle East	1419 (594–1904)	0.92 (0.4–1.24)	5878 (2720–7511)	1.37 (0.65–1.73)	1.51 (1.37 to 1.64)
Oceania	1 (0–4)	0.05 (0-0.14)	0 (0–1)	0.01 (0-0.01)	-7.53 (-8.41 to -6.64)
South Asia	412 (154–575)	0.08 (0.03–0.11)	1496 (732–2051)	0.11 (0.05–0.15)	0.82 (0.71 to 0.92)
Southeast Asia	549 (283–724)	0.22 (0.11–0.29)	1600 (1005–2369)	0.25 (0.16–0.37)	0.19 (0.09 to 0.29)
Southern Latin America	435 (381–493)	0.96 (0.84–1.09)	726 (630–819)	0.81 (0.7–0.92)	-0.64 (-1.08 to -0.2)
Southern Sub-Saharan Africa	269 (118–364)	1.05 (0.47–1.43)	789 (368–1023)	1.46 (0.7–1.88)	1.21 (1.01 to 1.4)
Tropical Latin America	448 (414–480)	0.53 (0.49–0.57)	1790 (1618–1926)	0.71 (0.64–0.76)	0.88 (0.72 to 1.04)
Western Europe	19,518 (18323–20520)	3.3 (3.1–3.47)	31,227 (27486–34098)	3.12 (2.78–3.39)	-0.27 (-0.64 to 0.11)
Western Sub-Saharan Africa	188 (98–242)	0.24 (0.13–0.31)	556 (250–726)	0.32 (0.15–0.41)	1.06 (1 to 1.11)

In GBD 2021, the global number of CLL deaths increased from 27,278 in 1990 to 45,573 in 2021, whereas the ASDR decreased from 0.83 to 0.55 (EAPC=-1.45) (Table S23). In 2021, from GBD 2021, high SDI countries had the highest mortality rates and ASDRs, 17,961 and 0.73, respectively (Fig. 2F; Figure S1F; Table S23). Among the 21 global regions, Western Europe had the highest number of deaths (11,068); while Southern Sub-Saharan Africa had the highest ASDR (1.20) (Table S23). At the 204 countries level, China had the highest number of deaths (8,636), followed by the United States (6,456) and Germany (2,447); whereas Ethiopia (2.33) had the highest ASDR, followed by Principality of Monaco (1.96) and Latvia (1.75) (Fig. 3F; Figure S11C; Table S25).

Between 1990 and 2021, from GBD 2021, global CLL DALYs increased from 0.72 to 1.00 million and ASDALYR decreased from 18.10 to 11.81 (EAPC =-1.52) (Table S24). In 2021, from GBD 2021, DALYs were highest in the high SDI countries (0.31 million), whereas ASDALYR was highest in the high and middle SDI countries (15.65) (Fig. 2F; Figure S1F; Table S24). Among the 21 global regions, East Asia DALYs were the highest (0.27 million); while Southern Sub-Saharan Africa had the highest ASDALYR (25.14) (Table S24). At the 204 countries level, China had the highest DALYs (0.27 million), followed by the United States (0.11 million) and the Russian Federation (0.05 million); while Ethiopia (49.60) had the highest ASDALYR, followed by Latvia (41.53) and Principality of Monaco (38.64) (Figure S11D, S11F; Table S25).

The peak prevalence, incidence, and DALYs for CLL occurred at 70–74 years of age, while the peak mortality for ALL was slightly delayed (80–84 years) (Fig. 4F, Figure S13F). We also found that SDI was statistically positively correlated with ASPR, ASIR, ASDR, and ASDALYR (ASPR: ρ = 0.671, *p* < 0.001; ASIR: ρ = 0.628, *p* < 0.001; ASDR: ρ = 0.369, *p* < 0.001; and ASDALYR: ρ = 346, *p* < 0.001) (Fig. 6F. Figure S14F). Globally, smoking contributed more to CLL mortality and DALYs than high BMI; and the contribution of high BMI and smoking to CLL mortality and DALYs increased progressively with increasing levels of SDI; among the 21 global regions, high BMI and smoking in High-income North America had the had the greatest impact (Fig. 7E). For the 2040 projections, the ASIR and ASDALYR for CLL tended to be slowly downwardly adjusted for both males and females (Fig. 8F, Figure S15F).

### Multiple myeloma burden

Between 1990 and 2021, from GBD 2021, MM global prevalent cases more than tripled from 0.12 to 0.39 million and the ASPR increased from 3.13 to 4.55 (EAPC = 1.24) (Table S26). The global number of MM incident cases nearly tripled from 55,710 in 1990 to 148,755 in 2021; and the ASIR increased from 1.47 to 1.74 (EAPC = 0.48) (Table 7). According to the GLOBOCAN 2022 database, in 2022, the number of incident cases of MM globally was 187,952, and the ASIR was 1.79 (Fig. 1I and J). In 2021, from GBD2021, there was a gradual decrease in new cases and ASIR from high to low SDI countries (Fig. 2G; Figure S1G; Table 7). Among the 21 global regions, Western Europe had the highest incidence (41,185) and Australasia had the highest ASIR (5.48) (Table 7). At the 204 countries level, the United States had the highest number of new cases (17,693), followed by China (17,250) and India (12,588); Principality of Monaco (6.86), Commonwealth of the Bahamas (6.55), and New Zealand (6.00) had the top three ASIR rankings (Fig. 3G; Figure S12B; Table S29). According to the GLOBOCAN 2022 database, in 2022, France, Martinique (6.22), Barbados (6.13), and France, Guadeloupe (5.31) ranked the top three in ASIR (Fig. 3G).

**Table 7:** The incidence of MM in 1990 and 2021. Annotations: ASIR: Age-standardized incidence rate (per 100,000 population); EAPC: estimated annual percentage change; SDI: Socio-demographic index

Location	1990 incident cases (95% CI)	1990 ASIR (95% CI)	2021 incident cases (95% CI)	2021 ASIR (95% CI)	EAPC (95% CI)
Global	55,710 (52022–59688)	1.47 (1.37–1.57)	148,755 (131780–162049)	1.74 (1.54–1.89)	0.48 (0.37 to 0.6)
High SDI	33,358 (31615–34445)	2.98 (2.83–3.08)	68,288 (61342–72525)	3.16 (2.87–3.34)	0.15 (-0.02 to 0.33)
High-middle SDI	12,562 (11834–13538)	1.27 (1.2–1.37)	34,788 (30245–38625)	1.75 (1.52–1.95)	1.01 (0.89 to 1.13)
Middle SDI	5249 (4561–6753)	0.51 (0.45–0.66)	28,498 (22906–33492)	1.05 (0.84–1.23)	2.15 (1.98 to 2.33)
Low-middle SDI	3222 (2332–4255)	0.54 (0.39–0.71)	13,201 (11293–18483)	0.92 (0.79–1.3)	1.72 (1.64 to 1.8)
Low SDI	1243 (696–1750)	0.56 (0.32–0.79)	3801 (2523–5131)	0.77 (0.51–1.02)	0.95 (0.78 to 1.13)
Andean Latin America	232 (170–295)	1.15 (0.85–1.46)	1061 (825–1379)	1.8 (1.4–2.33)	1.59 (1.4 to 1.78)
Australasia	987 (921–1058)	4.16 (3.88–4.46)	2991 (2605–3386)	5.48 (4.77–6.21)	1.03 (0.9 to 1.17)
Caribbean	615 (568–677)	2.39 (2.2–2.63)	1712 (1464–1961)	3.17 (2.71–3.63)	0.98 (0.86 to 1.1)
Central Asia	137 (119–154)	0.28 (0.24–0.31)	440 (392–493)	0.5 (0.45–0.56)	2.42 (2.03 to 2.81)
Central Europe	2181 (2047–2297)	1.44 (1.35–1.51)	4952 (4506–5371)	2.21 (2.01–2.4)	1.36 (1.16 to 1.56)
Central Latin America	924 (895–950)	1.1 (1.06–1.13)	4144 (3699–4654)	1.63 (1.46–1.83)	1.15 (1.05 to 1.26)
Central Sub-Saharan Africa	81 (54–109)	0.36 (0.25–0.49)	240 (132–345)	0.44 (0.24–0.63)	0.68 (0.4 to 0.96)
East Asia	1918 (1370–3616)	0.22 (0.15–0.41)	18,189 (11882–23583)	0.83 (0.54–1.07)	3.88 (3.25 to 4.51)
Eastern Europe	2944 (2789–3118)	1.03 (0.97–1.09)	6170 (5703–6690)	1.76 (1.63–1.9)	1.9 (1.64 to 2.15)
Eastern Sub-Saharan Africa	645 (351–924)	0.89 (0.5–1.26)	2059 (1253–2823)	1.24 (0.77–1.68)	1.06 (0.94 to 1.17)
High-income Asia Pacific	3969 (3680–4198)	1.99 (1.84–2.11)	9741 (8163–10907)	1.93 (1.66–2.16)	-0.04 (-0.22 to 0.14)
High-income North America	11,850 (11191–12239)	3.34 (3.17–3.45)	20,898 (19024–22011)	3.1 (2.83–3.26)	-0.39 (-0.54 to -0.24)
North Africa and Middle East	1370 (954–1861)	0.83 (0.58–1.13)	5840 (4386–8005)	1.3 (0.97–1.77)	1.6 (1.51 to 1.69)
Oceania	9 (6–13)	0.33 (0.21–0.48)	26 (15–37)	0.36 (0.21–0.5)	0.28 (0.24 to 0.33)
South Asia	3538 (2254–4501)	0.63 (0.4–0.8)	15,905 (12551–21562)	1.09 (0.86–1.47)	1.62 (1.46 to 1.78)
Southeast Asia	756 (601–1226)	0.3 (0.24–0.48)	3508 (2721–5681)	0.53 (0.41–0.86)	1.8 (1.75 to 1.85)
Southern Latin America	1028 (954–1114)	2.21 (2.06–2.4)	2033 (1876–2190)	2.33 (2.15–2.5)	0.25 (0.08 to 0.41)
Southern Sub-Saharan Africa	403 (276–521)	1.49 (1.01–1.94)	1351 (891–1639)	2.3 (1.51–2.77)	1.49 (1.36 to 1.63)
Tropical Latin America	1200 (1149–1245)	1.3 (1.23–1.35)	5412 (5051–5693)	2.1 (1.95–2.21)	1.49 (1.32 to 1.66)
Western Europe	20,706 (19518–21531)	3.54 (3.35–3.68)	41,185 (36907–44033)	4.3 (3.91–4.57)	0.64 (0.43 to 0.86)
Western Sub-Saharan Africa	216 (113–301)	0.26 (0.13–0.36)	896 (366–1316)	0.48 (0.2–0.69)	2.15 (2.01 to 2.29)

In GBD 2021, the global number of MM deaths more than tripled from 47,569 in 1990 to 116,360 in 2021; while the ASDR rose from 1.29 to 1.37 (EAPC = 0.09) (Table S27). According to the GLOBOCAN 2022 database, in 2022, the number of deaths of MM globally was 121,388, and the ASDR was 1.11 (Fig. 1I and J). In 2021, from GBD 2021, high SDI countries had the highest death rates and ASDRs at 51,435 and 2.28, respectively (Fig. 2G; Figure S1G; Table S27). Among the 21 global regions, Western Europe had the highest number of deaths (26,875); while Australasia had the highest ASDR (2.89) (Table S27). At the 204 countries level, the United States had the highest number of deaths (17,370), followed by China (12,984) and India (11,635); while the Commonwealth of the Bahamas (4.71) had the highest ASDR, followed by Principality of Monaco (4.40) and Grenada (3.57) (Fig. 3G; Figure S12C; Table S29). According to the GLOBOCAN 2022 database, in 2022, Barbados (4.00), Bahamas (3.82), and Jamaica (3.23) ranked the top three in ASDR (Fig. 3G).

Between 1990 and 2021, from GBD 2021, global MM DALYs more than tripled from 1.12 to 2.60 million, and the ASDALYR increased from 28.34 to 30.00 (EAPC = 0.06) (Table S28). In 2021, from GBD 2021, the highest MM DALYs and ASDALYRs were found in the high SDI countries, with 0.98 million and 47.33, respectively (Fig. 2G; Figure S1G; Table S28). Among the 21 global regions, Western Europe DALYs were the highest (0.49 million); while Australasia had the highest ASDALYR (60.65) (Table S28). At the 204 countries level, China had the highest DALYs (0.34 million), followed by the United States (0.34 million) and India (0.30 million); while Commonwealth of the Bahamas (117.52) had the highest ASDALYR, followed by Principality of Monaco (93.34) and Zimbabwe (88.68) (Figures S12D, S12F; Table S29).

The peak prevalence, incidence and mortality of MM occurred at 70–74 years, while the peak of DALYs was slightly earlier (65–69 years) (Figs. 4G and 5D; Figure S13G). We also found that SDI was statistically positively correlated with ASPR, ASIR, ASDR, and ASDALYR (ASPR: ρ = 0.647, *p* < 0.001; ASIR: ρ = 0.706, *p* < 0.001; ASDR: ρ = 0.685, *p* < 0.001; and ASDALYR: ρ = 669, *p* < 0.001) (Fig. 6G. Figure S14G). Globally, the contribution of high BMI and smoking to MM mortality and DALYs increased progressively with increasing levels of SDI; among the 21 global regions, high BMI and smoking had the largest effect in North Africa and Middle East (Fig. 7F). For the 2040 projections, the ASIR and ASDALYR for MM showed relatively stable trends for both males and females (Fig. 8G, Figure S15G).

## Discussion

To the best of our knowledge, this study represents the maiden attempt to conduct the most exhaustive and contemporaneous assessment of hematologic malignancies by leveraging the GBD 2021 and GLOBOCAN 2022. It encompasses HL, NHL, AML, CML, ALL, CLL, and MM. Our research has rejuvenated the epidemiological evidence about hematologic malignancies, proffering a detailed synopsis and novel perspectives. Our investigation centered around prevalence, incidence, mortality rate, DALYs, and their respective age-standardized rates per 100,000 individuals. Furthermore, elaborate classificatory analyses were carried out based on age, gender, geographical location, and human socioeconomic status. Notably, we have ascertained an overall downward trajectory in both the ASIR and the ASDALYR of hematologic malignancies projected up to 2040, which mirrors the unflagging efforts dedicated to the prevention, early detection, and treatment of malignant hematologic neoplasms over the years. While the published studies based on GBD 2021 and GLOBOCAN 2022 data focused on individual subtypes (e.g., AML, MM) [13, 14], our work provided a comprehensive evaluation of seven distinct subtypes (HL, NHL, AML, CML, ALL, CLL, MM) using integrated GBD 2021 and GLOBOCAN 2022 data, enabling cross-validation and enhanced granularity. We incorporated age-sex-SDI stratified analyses to reveal unique demographic patterns, such as the bimodal age distribution of HL incidence and mortality, which were not addressed in prior studies. Meanwhile, our study utilizes the latest Global Burden of Disease 2021 and GLOBOCAN 2022 datasets, providing updated epidemiological trends (1990–2022) compared to previous analyses based on GBD 2019 [2]. We also extended the temporal scope by projecting ASIR and ASDALYR trends up to 2040 using Bayesian age-period-cohort modeling, offering actionable insights for long-term policy planning. By harmonizing two major datasets and emphasizing subtype-specific heterogeneity, our work provides a more nuanced understanding of the evolving global burden of hematologic malignancies, addressing critical gaps in prior analyses. While our analysis highlights disparities in hematologic malignancy burdens, it does not directly evaluate healthcare system performance through survival outcomes. Future studies integrating cancer registry data with treatment accessibility metrics (e.g., availability of stem cell transplantation, CAR-T therapy centers) could better assess how healthcare investments translate into survival improvements, particularly in underserved regions.

For nearly 30 years, the global burden of Hodgkin’s lymphoma (HL) has shown varying trends from 1990 to 2021. While incident cases increased, ASIR remained stable or slightly decreased globally. ASDR and ASDALYR have been declining. This is related to the fact that patients with early stages are successfully treated with chemotherapy and, radiotherapy while the use of immunotherapy in patients with relapse and recently also in patients with advanced stages as first-line therapy has significantly improved survival rates [4, 22, 23]. It is noteworthy that a prominent feature of HL is that the DALYs are the highest among young people aged 20–35. These young people require active prevention and early diagnosis. SDI levels influence HL burden, with higher SDI regions showing higher incidence but lower mortality and DALYs. From 1990 to 2021, the incidence rate of NHL has shown a sharp upward trend globally, especially in higher SDI regions and East Asia. However, the ASDR and ASDALYR remained relatively stable or declined, especially in higher SDI regions. The increase in the incidence rate can be attributed to various factors, including improved diagnostic techniques, the HIV epidemic, and immunosuppressive therapies. Meanwhile, advances in molecular subtyping, targeted therapies, and immunotherapies have reduced the mortality rate [24, 25].

Globally, the incidence rate of AML is gradually increasing, and the ASDR of AML still remains at a high level, especially in higher SDI regions and among the elderly population, which is consistent with previous reports [12, 13]. The occurrence of AML is also associated with smoking and high BMI, particularly in higher SDI regions. Owing to the accelerated aging of the population, the global burden of AML has been aggravated [26]; moreover, the prognosis of elderly AML patients is poorer than that of younger patients [27]. Despite these advances, challenges remain in treating older patients unfit for intensive chemotherapy and those with relapsed or refractory disease [7, 28]. Another potential contributor to the rising burden of AML may be the increasing incidence of therapy-related AML (t-AML), which arises as a late complication of cytotoxic chemotherapy or radiation therapy administered for prior malignancies [29, 30]. Over the past decades, advancements in cancer treatment have significantly improved survival rates for patients with solid tumors and hematologic malignancies. However, prolonged exposure to genotoxic therapies (e.g., alkylating agents, topoisomerase II inhibitors) has been associated with a higher risk of secondary AML, particularly in long-term survivors [29, 30]. For example, studies suggest that t-AML accounts for 10–20% of all AML cases, with a rising trend attributed to the growing population of cancer survivors [30]. This phenomenon may partially explain the observed increase in AML incidence, especially in regions with advanced healthcare systems where cancer therapies are widely accessible. Future strategies to mitigate the burden of AML should therefore consider both primary prevention of de novo AML and surveillance for secondary cases in high-risk populations.

From 1990 to 2021, ALL has imposed a substantial global burden on children. The number of newly diagnosed cases and prevalent cases of ALL witnessed an inflection point in 2019, and both the mortality rate and DALYs have been continuously decreasing. Higher SDI areas have higher incidence and lower mortality burden. The development of disease risk stratification and intensified chemotherapy regimens have significantly improved the outcomes for patients with acute lymphoblastic leukemia, leading to a marked decline in the mortality rate among children. However, the prognosis remains poor for the elderly population and patients with relapsed or refractory acute lymphoblastic leukemia [6]. Adult acute lymphoblastic leukemia still presents challenges and has a relatively poor prognosis, especially in elderly patients who cannot tolerate intensive treatment [31].

Over the past three decades or more, the incidence rate of CML has remained essentially stable, while the mortality rate and DALYs have declined significantly, especially in regions with a higher SDI. The remarkable reduction in the mortality rate can be attributed to the introduction of tyrosine kinase inhibitors (TKIs), which has revolutionized the treatment of CML. Imatinib, in particular, has become the first-line treatment due to its efficacy and tolerability, substantially prolonging the lifespan of CML patients [9]. From 1990 to 2021, the global burden of CLL has increased remarkably, with a higher incidence rate among males and the elderly. The ASDR of CLL has dropped sharply in regions with a higher SDI, which may be attributable to the availability of new diagnostic techniques and treatment methods [8]. Nevertheless, the rising mortality rate in Africa is concerning, highlighting potential disparities in the availability of health resources. High body mass index and smoking are major factors contributing to CLL-related mortality and DALYs.

Our analysis highlights MM as a significant contributor to the global burden of hematologic malignancies. From 1990 to 2021, MM exhibited a tripling in global prevalence and incident cases, with ASIR rising by 0.48% annually (EAPC = 0.48). This upward trajectory aligns with global aging populations and improved diagnostic capabilities, particularly in high SDI regions, where ASIR and mortality rates were highest. High BMI emerged as a critical modifiable risk factor, contributing disproportionately to MM-related deaths and DALYs in high-SDI countries, where obesity rates are elevated. Despite therapeutic advancements, such as proteasome inhibitors and immunomodulatory drugs [32], MM mortality and DALYs remain substantial, especially among elderly populations, who often face comorbidities limiting treatment efficacy. Projections to 2040 suggest stable ASIR and ASDALYR trends, underscoring the need for targeted interventions-including weight management programs and equitable access to novel therapies mitigate MM’s growing burden. These findings emphasize the importance of integrating preventive strategies with healthcare system strengthening, particularly in aging societies, and high-SDI regions where MM incidence is projected to persist.

Over the past 30 years, the global burden of hematologic malignancies has shown discrepancies. In terms of regions, in those with a higher SDI, due to advancements in medical technology, improvement in the quality of medical services, breakthroughs in innovative drugs, and other reasons, there has been a relatively high incidence rate. Conversely, owing to the imbalance in socio-economic development, insufficient investment in healthcare and inadequate health awareness have exacerbated the disease burden in lower SDI regions. This situation could be ameliorated through global partnerships. In terms of age, with the intensification of population aging, the global burden of the vast majority of hematologic malignancies has increased. Notably, age-specific burden patterns reflect both biological and systemic healthcare factors. For instance, pediatric ALL outcomes have improved dramatically due to risk-stratified chemotherapy protocols [33], yet survival gaps persist in low-SDI regions where treatment abandonment and toxicity management remain challenges. Conversely, older adults with AML face under-representation in clinical trials and limited access to novel therapies, contributing to stagnant mortality rates despite therapeutic advances [34]. These disparities underscore the need for age-tailored strategies that address biological heterogeneity and healthcare inequities. In terms of gender, men remain the high-risk group for hematologic malignancies. This may be attributed to the fact that smoking remains the major risk factor for hematologic malignancy-related deaths [35], and high BMI is becoming increasingly significant. Certainly, there are also deficiencies in this study. Firstly, there is a lack of epidemiological statistical data for each pathological subtype of cancer. For example, with the development of precision medicine and the progress of molecular subtyping, NHL encompasses numerous subtypes, and there are obvious differences in disease origin, malignancy degree, diagnosis, and treatment among these subtypes, which has led to imprecise analysis [36, 37]. Secondly, the COVID-19 pandemic has caused significant disruptions to cancer services, resulting in substantial delays in diagnosis and treatment [38]. Finally, in this study, instead of integrating the data, we conducted partial and longitudinal analyses separately, using a unified standardized age group and national classification. Therefore, when processing and interpreting the relevant results, the specific limitations of each database must be taken into account. In addition, due to the fact that the GBD 2021 and GLOBOCAN 2022 employ different modeling approaches and data sources, the integration and comparison of the data are rather complex, and it will inevitably lead to some data inconsistencies [39].

Our study has several limitations related to data quality and regional comparability. First, the diagnosis of hematologic malignancies (e.g., lymphoma subtypes, acute leukemias) heavily relies on specialized techniques (e.g., immunohistochemistry, flow cytometry, cytogenetic analysis), which are often unavailable in low-resource settings. This may lead to underdiagnosis or misclassification, particularly in low-SDI regions where cancer registries may lack population coverage or rely on death certificate-only (DCO) data. For instance, the higher incidence of non-Hodgkin lymphoma in high-SDI regions might partially reflect better diagnostic capacity rather than true risk differences. Second, temporal trends (e.g., declining Hodgkin lymphoma mortality) could be confounded by improved case ascertainment over time, especially in regions with historically weak registration systems. While the GBD and GLOBOCAN models attempt to correct for under-reporting through statistical imputation, residual biases may persist. These limitations necessitate cautious interpretation of regional comparisons, particularly for subtypes requiring advanced diagnostics.

The interpretation of long-term trends in hematologic malignancies must account for evolving disease classification systems. A notable example is the reclassification of ALL-L3 (FAB, French-American-British classification), which was designated as synonymous with Burkitt lymphoma/leukemia in the 2001 WHO Classification of Tumors of Hematopoietic and Lymphoid Tissues. Subsequent iterations (2008 and 2016 editions) discontinued this synonymy while maintaining its classification under mature B-cell neoplasms [40–42]. Such diagnostic shifts are particularly impactful for entities straddling traditional categories. Blastic plasmacytoid dendritic cell neoplasm (BPDCN), historically misclassified as AML or lymphoma, gained recognition as a distinct entity in the 2016 WHO classification [40–42]. This reclassification likely creates artificial reductions in historical AML incidence rates while inflating newer diagnostic categories. Persistent confounding is anticipated, particularly in low-resource settings where diagnostic capabilities lag behind classification updates. These limitations emphasize two critical needs: (1) cautious interpretation of apparent epidemiological trends, and (2) urgent implementation of standardized global reporting protocols to ensure diagnostic harmonization across regions and temporal periods.

Our projections utilize BAPC modeling with integrated nested Laplace approximations, a method validated in prior burden studies for its superior precision in capturing demographic transitions [43–46]. While acknowledging potential impacts from healthcare disruptions or therapeutic breakthroughs, these projections provide critical baseline estimates for strategic planning. Our findings guide policymakers to adopt tailored strategies for hematologic malignancies. High-SDI regions should prioritize preventive measures (e.g., tobacco control, obesity campaigns) to address modifiable risks (smoking, high BMI) for AML, CML, and MM. Low/middle-SDI regions require investments in diagnostics, affordable therapies, and specialist training to reduce high mortality. Global partnerships to share technologies, risk-stratified protocols, and surveillance frameworks can harmonize standards and reduce inequities.

Our study equips policymakers with actionable insights to address the heterogeneous burden of hematologic malignancies. The stark regional disparities tied to SDI levels demand tailored interventions: high-SDI regions should intensify prevention by targeting modifiable risks like smoking and obesity via public health campaigns and regulations, while low/middle-SDI regions require urgent investments in healthcare infrastructure, early diagnosis, and affordable therapies to reduce mortality and DALYs. Age-stratified burdens-notably pediatric ALL and young adult HL-necessitate age-specific care pathways, including pediatric screening and specialized oncology programs. Although projected declines in ASIR and ASDALYR by 2040 reflect progress, sustaining these gains requires scaling proven interventions (e.g., precision diagnostics, immunotherapy) while addressing aging populations’ rising vulnerability. The persistent role of smoking and high BMI underscores the need for integrated non-communicable disease strategies. Global collaboration is critical to equitably share innovations in risk reduction (e.g., tobacco control), diagnostics, and treatment protocols, particularly for resource-limited settings. Policymakers should prioritize funding for translational research, workforce training, and equitable access to care, guided by these data-driven trends. By aligning resource allocation with disease patterns and risk factors, nations can mitigate disparities and reduce the societal impact of hematologic malignancies.

## Conclusion

Our research furnishes a thorough and comprehensive synopsis of the global burden of hematologic malignancies. The findings of our study demonstrate that, in tandem with the aging of the global population, the burden of hematologic malignancies across the globe is exceedingly substantial and manifests disparities in domains including region, age, and gender. In order to tackle these challenges, it is imperative to augment prevention and early detection initiatives and to proactively administer treatments, particularly in underdeveloped regions. On an encouraging front, it is anticipated that by 2040, the global ASIR and ASDALYR will exhibit a gradual downward propensity.

## Electronic supplementary material

Below is the link to the electronic supplementary material.

Supplementary Material 1

Supplementary Material 2

Supplementary Material 3

Supplementary Material 4

Supplementary Material 5

## Data Availability

No datasets were generated or analysed during the current study.

## Abbreviations

GBD: Global Burden of Disease
HL: Hodgkin lymphoma
NHL: Non − Hodgkin lymphoma
AML: Acute myeloid leukemia
CML: Chronic myeloid leukemia
ALL: Acute lymphoid leukemia
CLL: Chronic lymphoid leukemia
MM: Multiple myeloma
DALY: Disability-adjusted life year
ASR: Age-standardized rate
ASPR: Age-standardized prevalence rate
ASIR: Age-standardized incidence rate
ASDR: Age-standardized death rate
ASDALYR: Age-standardized disability-adjusted life year rate
SDI: Socio-demographic index
EAPC: Estimated annual percentage change
CAR-T: Chimeric Antigen Receptor-Modified T
TKI: Tyrosine Kinase Inhibitors
DCO: Death Certificate-Only
FAB: French-American-British classification
BPDCN: Blastic plasmacytoid dendritic cell neoplasm
