# Supplementary material for: Global burden and trends of hematologic malignancies based on Global Cancer Observatory 2022 and Global Burden of Disease 2021
Source: Exp Hematol Oncol. 2025 Jul 17;14:98. doi: 10.1186/s40164-025-00684-x (PMC12273037; doi:10.1186/s40164-025-00684-x)

**Supplementary Figures**

**Figure S1:** Global and regional trends in ASPR, ASIR, ASDR, and ASDALYR in 1990-2021 from GBD. (A): HL, (B): NHL, (C): AML, (D): CML, (E): ALL, (F): CLL, (G): MM. HL, Hodgkin lymphoma; NHL, Non−Hodgkin lymphoma; AML, Acute myeloid leukemia; CML, Chronic myeloid leukemia; ALL, Acute lymphoid leukemia; CLL, Chronic lymphoid leukemia; MM, Multiple myeloma; DALYs, disability-adjusted life years; ASPR, age-standardized prevalence rate; ASIR, age-standardized incidence rate; ASDR, age-standardized death rate; ASDALYR, age-standardized DALY rate; SDI, socio-demographic index.

**
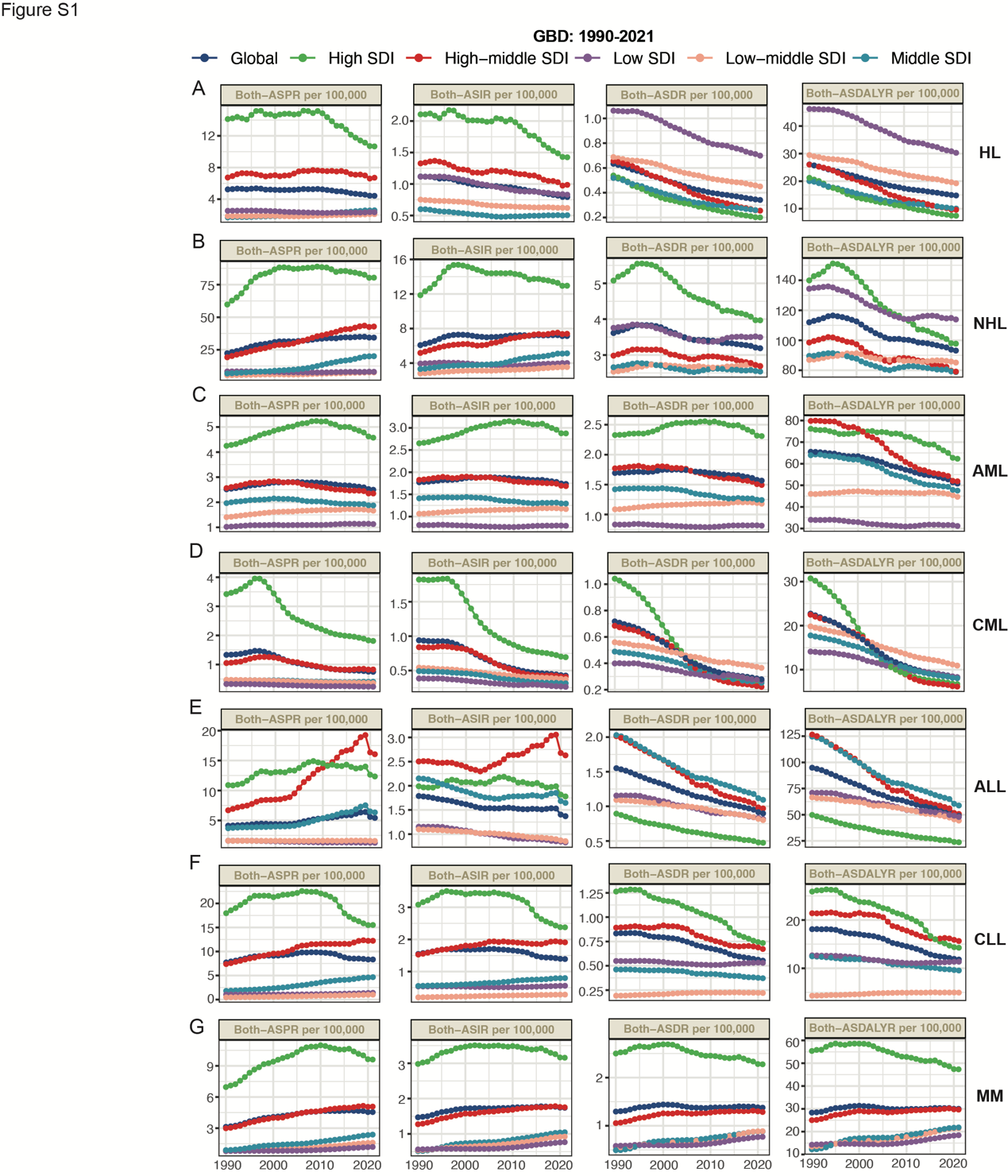
**

**Figure S2**: Global and regional trends in prevalence, incidence, deaths, and DALYs of male in 1990-2021 from GBD. (A): HL, (B): NHL, (C): AML, (D): CML, (E): ALL, (F): CLL, (G): MM. HL, Hodgkin lymphoma; NHL, Non−Hodgkin lymphoma; AML, Acute myeloid leukemia; CML, Chronic myeloid leukemia; ALL, Acute lymphoid leukemia; CLL, Chronic lymphoid leukemia; MM, Multiple myeloma; DALYs, disability-adjusted life years; SDI, socio-demographic index.


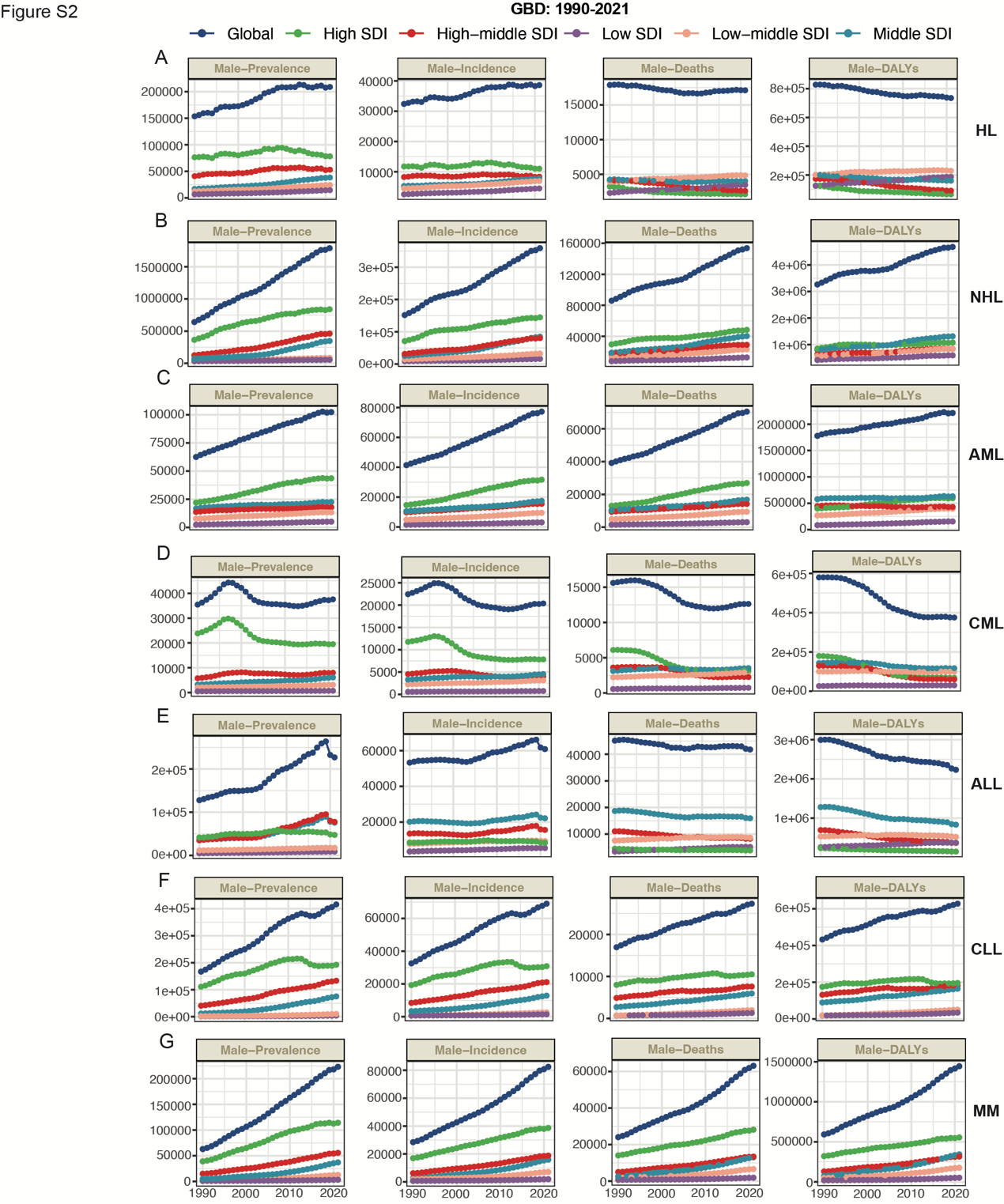


**Figure S3**: Global and regional trends in prevalence, incidence, deaths, and DALYs of female in 1990-2021 from GBD. (A): HL, (B): NHL, (C): AML, (D): CML, (E): ALL, (F): CLL, (G): MM. HL, Hodgkin lymphoma; NHL, Non−Hodgkin lymphoma; AML, Acute myeloid leukemia; CML, Chronic myeloid leukemia; ALL, Acute lymphoid leukemia; CLL, Chronic lymphoid leukemia; MM, Multiple myeloma; DALYs, disability-adjusted life years; SDI, socio-demographic index.


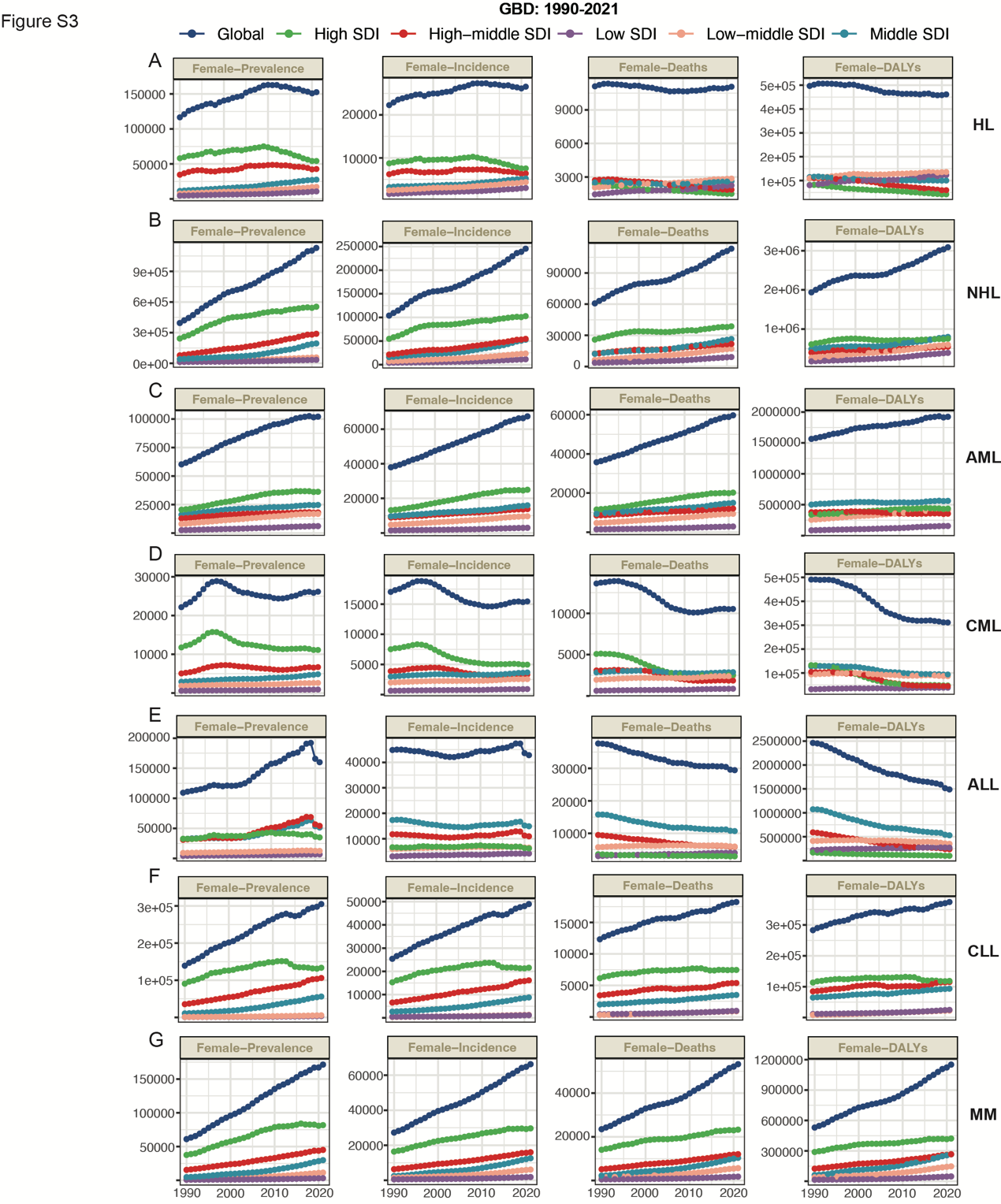


**Figure S4**: Global and regional trends in ASPR, ASIR, ASDR, and ASDALYR of male in 1990-2021 from GBD. (A): HL, (B): NHL, (C): AML, (D): CML, (E): ALL, (F): CLL, (G): MM. HL, Hodgkin lymphoma; NHL, Non−Hodgkin lymphoma; AML, Acute myeloid leukemia; CML, Chronic myeloid leukemia; ALL, Acute lymphoid leukemia; CLL, Chronic lymphoid leukemia; MM, Multiple myeloma; DALYs, disability-adjusted life years; ASPR, age-standardized prevalence rate; ASIR, age-standardized incidence rate; ASDR, age-standardized death rate; ASDALYR, age-standardized DALY rate; SDI, socio-demographic index.


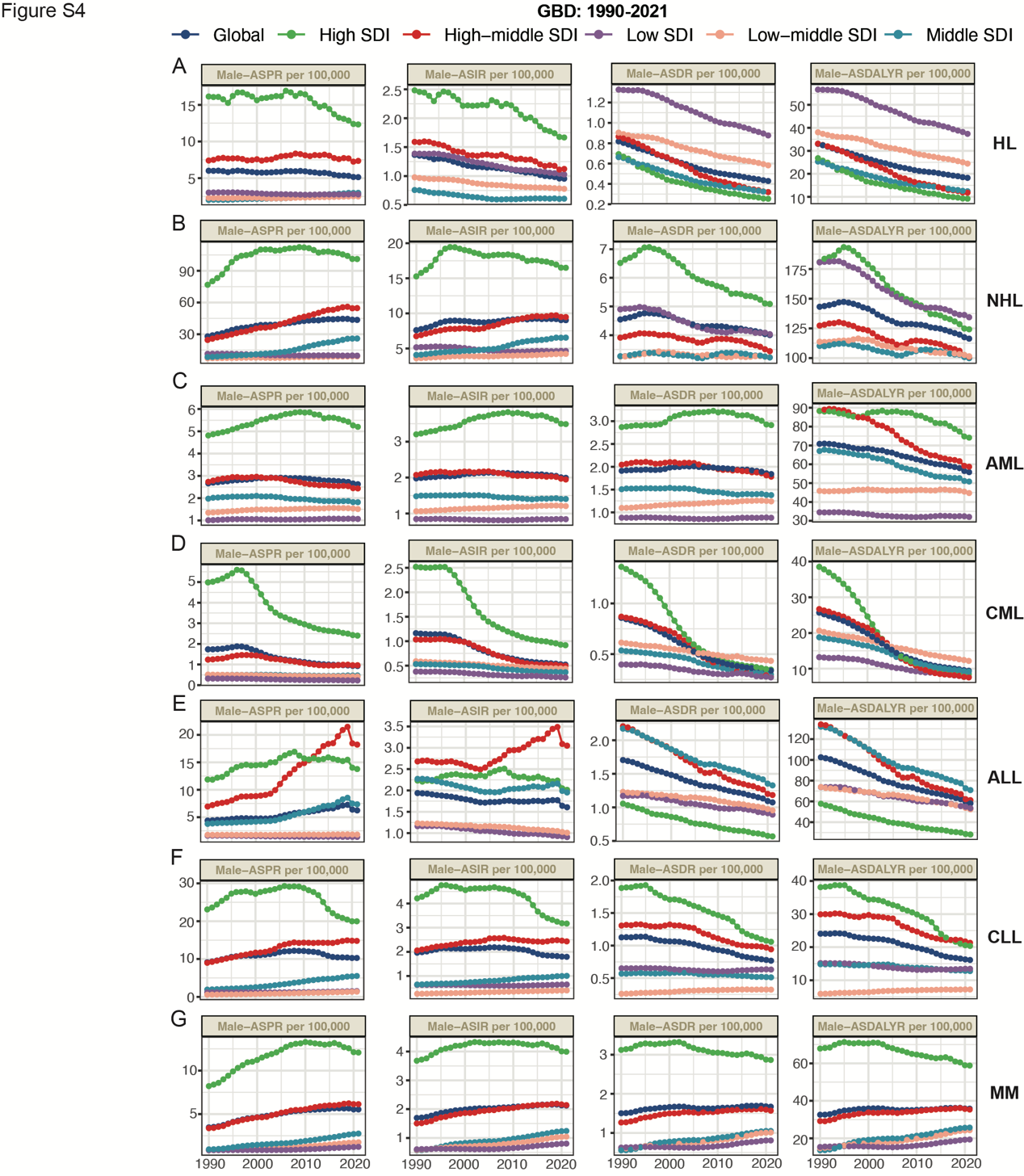


**Figure S5**: Global and regional trends in ASPR, ASIR, ASDR, and ASDALYR of female in 1990-2021 from GBD. (A): HL, (B): NHL, (C): AML, (D): CML, (E): ALL, (F): CLL, (G): MM. HL, Hodgkin lymphoma; NHL, Non−Hodgkin lymphoma; AML, Acute myeloid leukemia; CML, Chronic myeloid leukemia; ALL, Acute lymphoid leukemia; CLL, Chronic lymphoid leukemia; MM, Multiple myeloma; DALYs, disability-adjusted life years; ASPR, age-standardized prevalence rate; ASIR, age-standardized incidence rate; ASDR, age-standardized death rate; ASDALYR, age-standardized DALY rate; SDI, socio-demographic index.


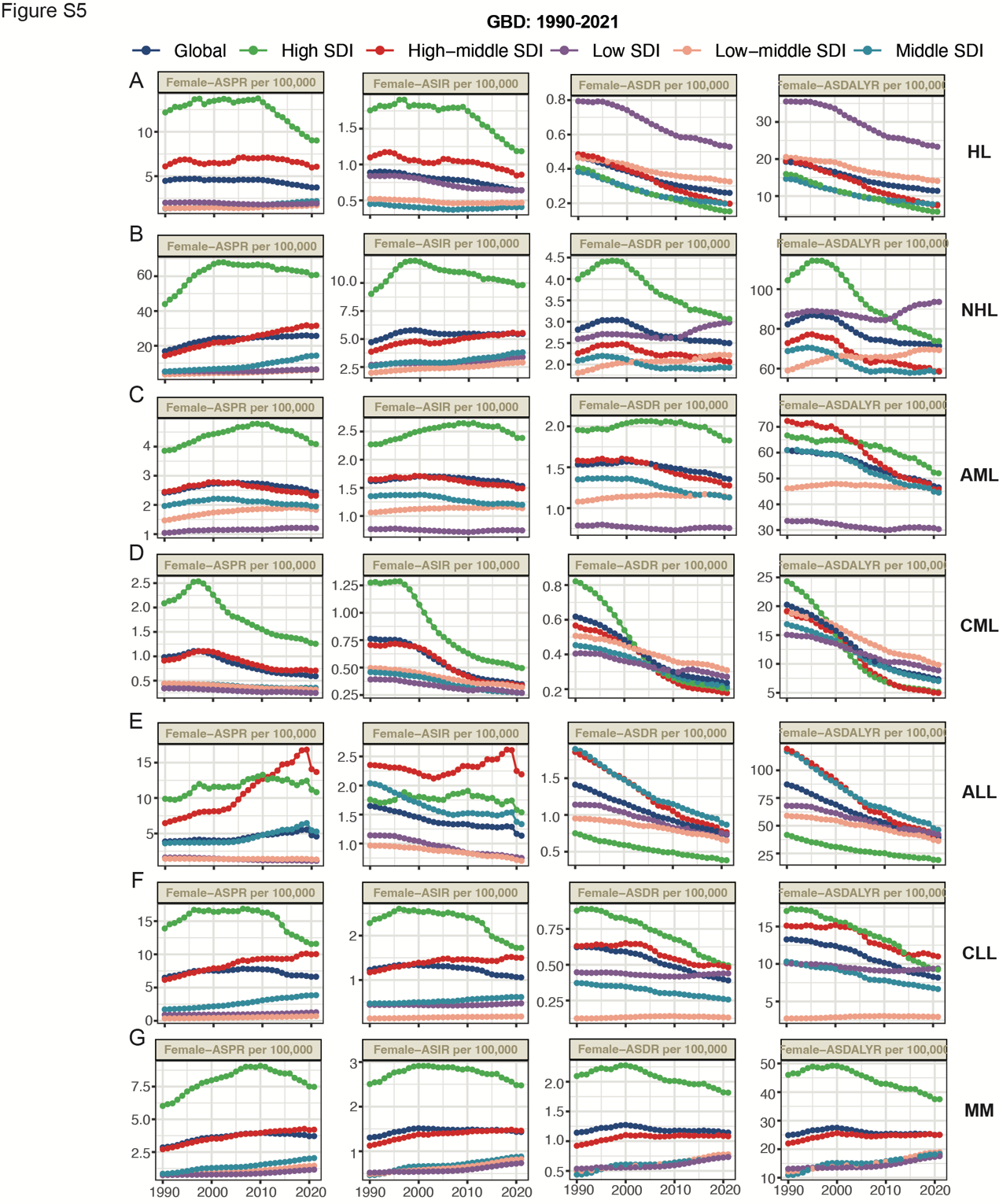


**Figure S6**: Global distribution of HL prevalence (A), incidence (B), deaths (C), DALYs (D), ASPR (E), and ASDALYR (F) in 2021 from GBD. HL, Hodgkin lymphoma; DALYs, disability-adjusted life years; ASPR, age-standardized prevalence rate; ASDALYR, age-standardized DALY rate.


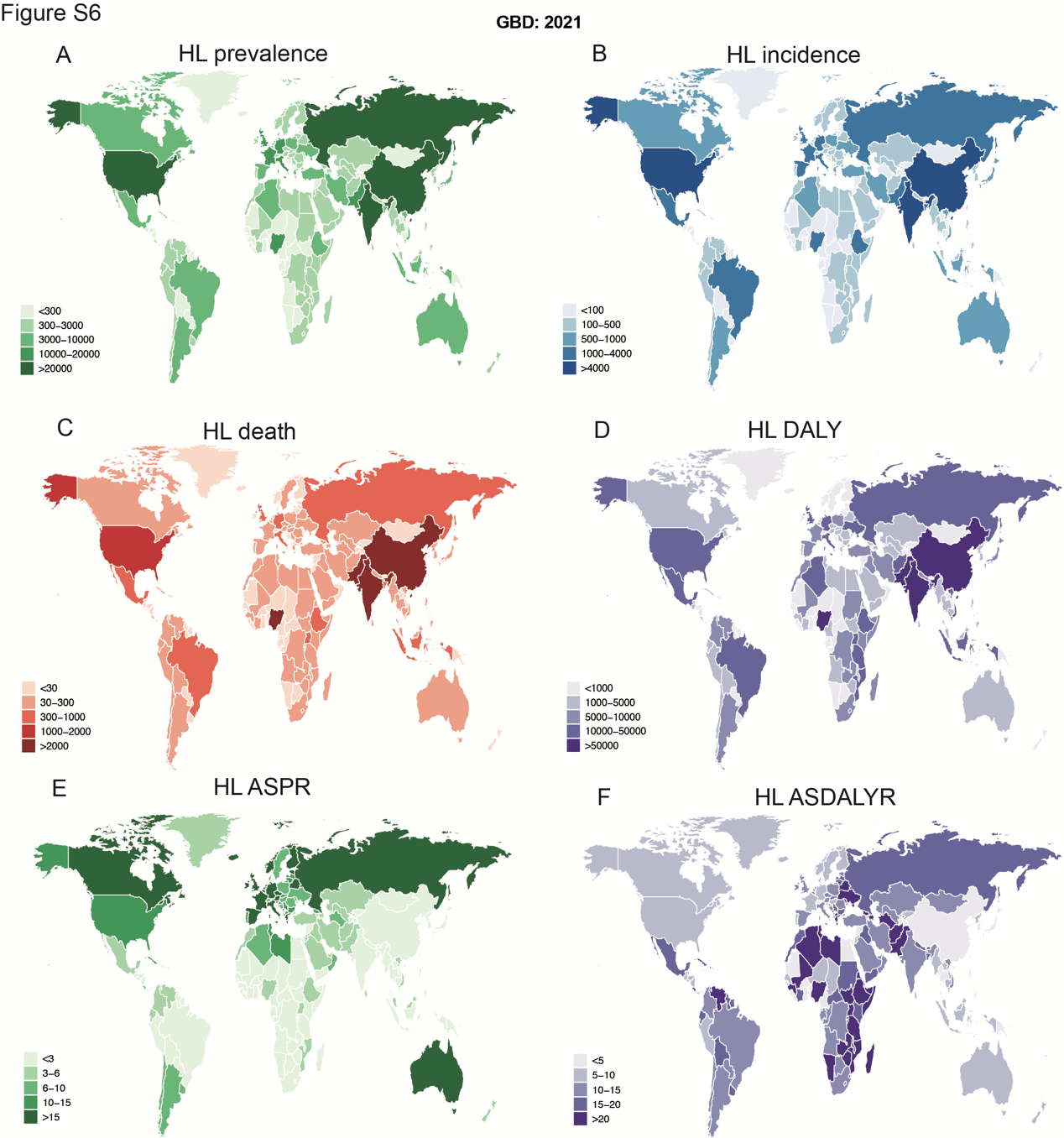


**Figure S7**: Global distribution of NHL prevalence (A), incidence (B), deaths (C), DALYs (D), ASPR (E), and ASDALYR (F) in 2021 from GBD. NHL, Non−Hodgkin lymphoma; DALYs, disability-adjusted life years; ASPR, age-standardized prevalence rate; ASDALYR, age-standardized DALY rate.


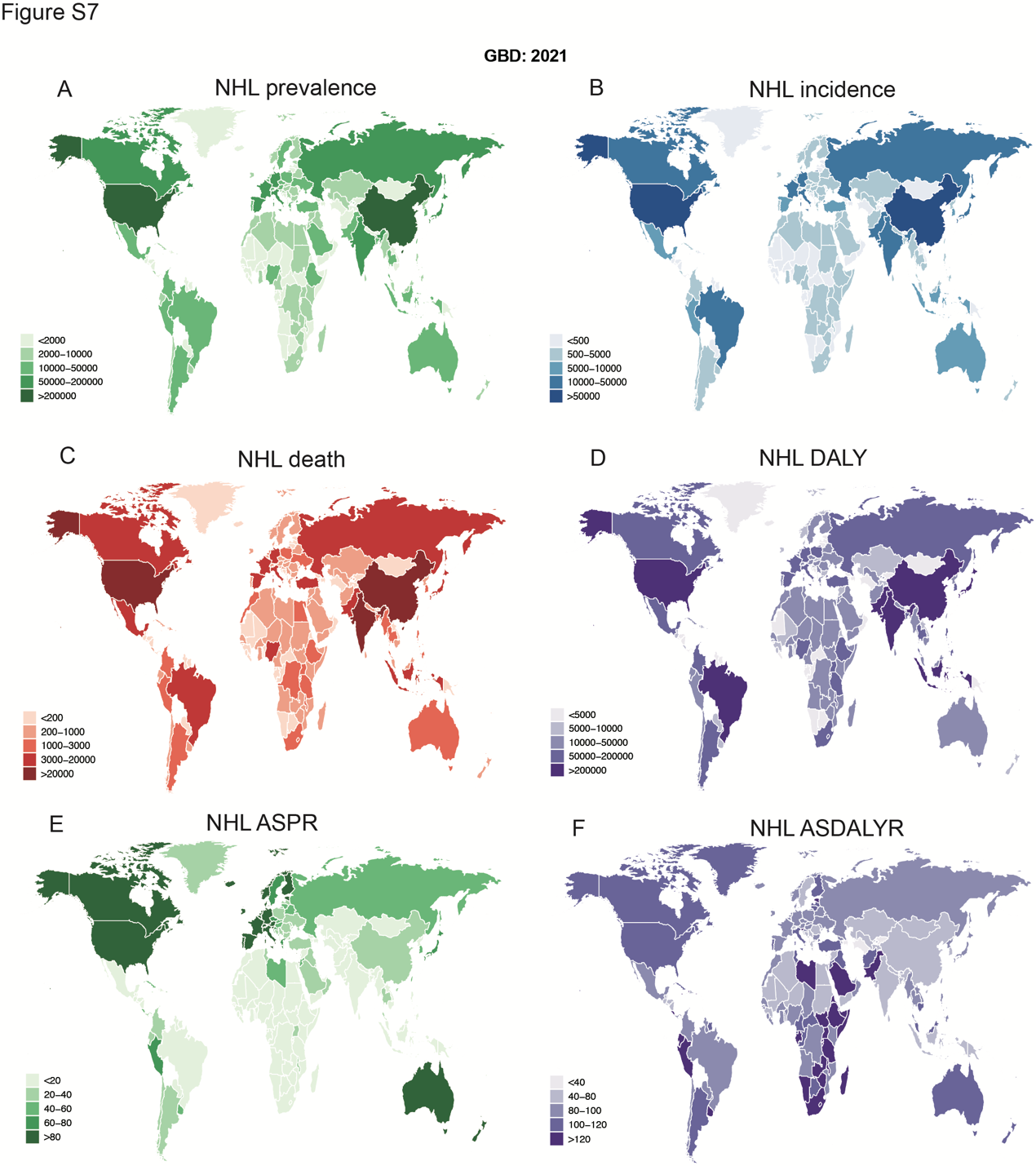


**Figure S8**: Global distribution of AML prevalence (A), incidence (B), deaths (C), DALYs (D), ASPR (E), and ASDALYR (F) in 2021 from GBD. AML, Acute myeloid leukemia; DALYs, disability-adjusted life years; ASPR, age-standardized prevalence rate; ASDALYR, age-standardized DALY rate.


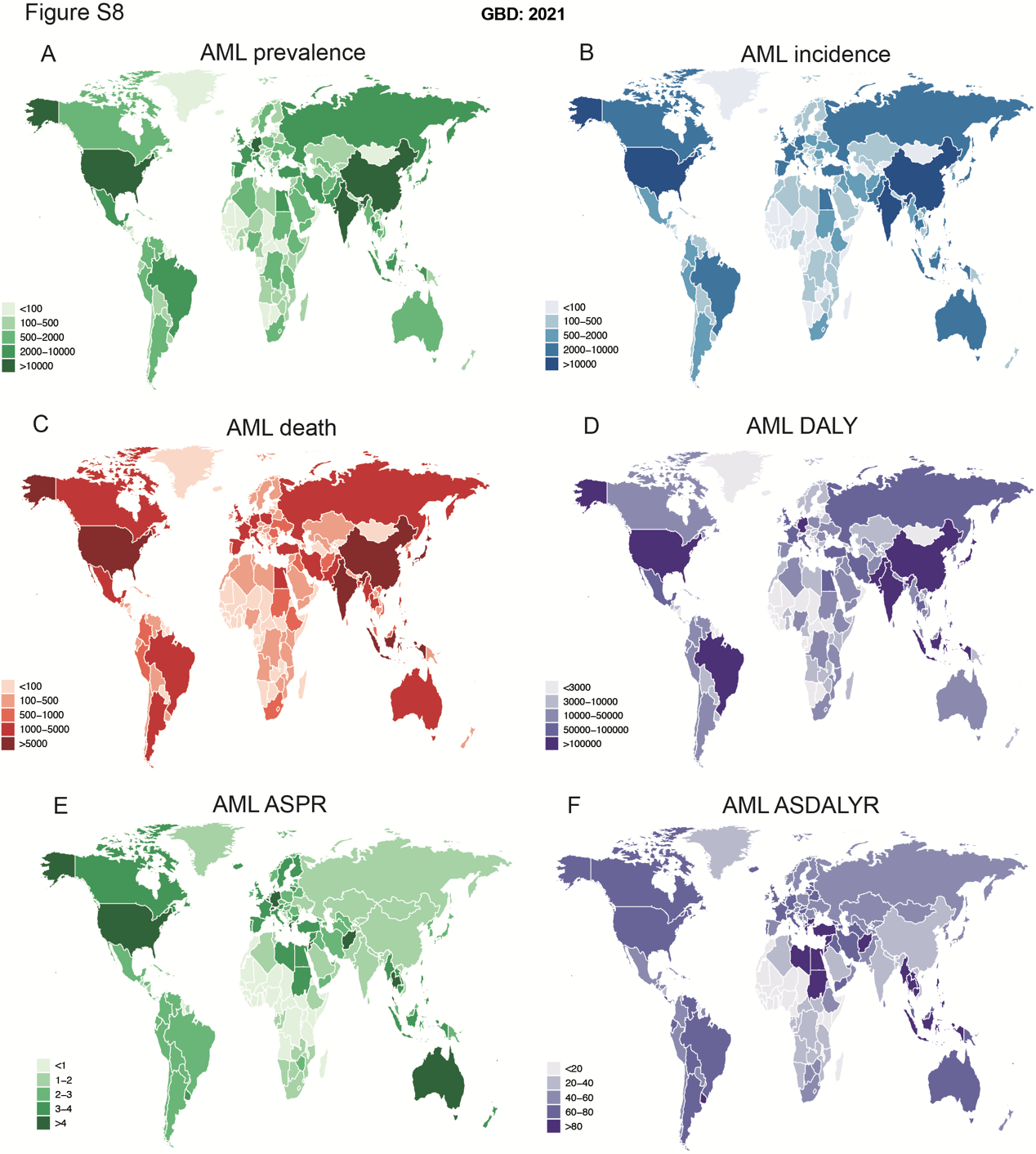


**Figure S9**: Global distribution of CML prevalence (A), incidence (B), deaths (C), DALYs (D), ASPR (E), and ASDALYR (F) in 2021 from GBD. CML, Chronic myeloid leukemia; DALYs, disability-adjusted life years; ASPR, age-standardized prevalence rate; ASDALYR, age-standardized DALY rate.


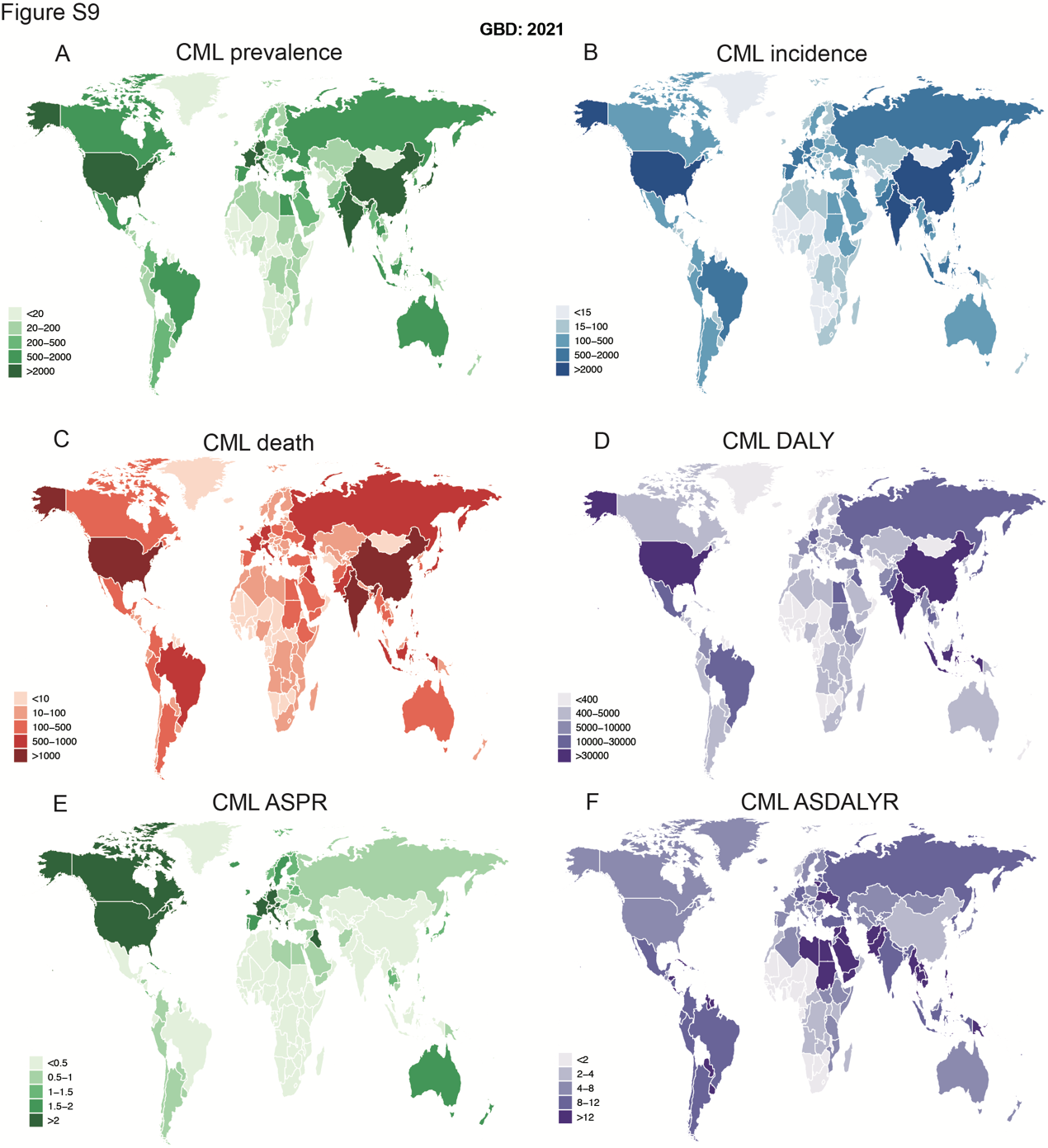


**Figure S10**: Global distribution of ALL prevalence (A), incidence (B), deaths (C), DALYs (D), ASPR (E), and ASDALYR (F) in 2021 from GBD. ALL, Acute lymphoid leukemia; DALYs, disability-adjusted life years; ASPR, age-standardized prevalence rate; ASDALYR, age-standardized DALY rate.


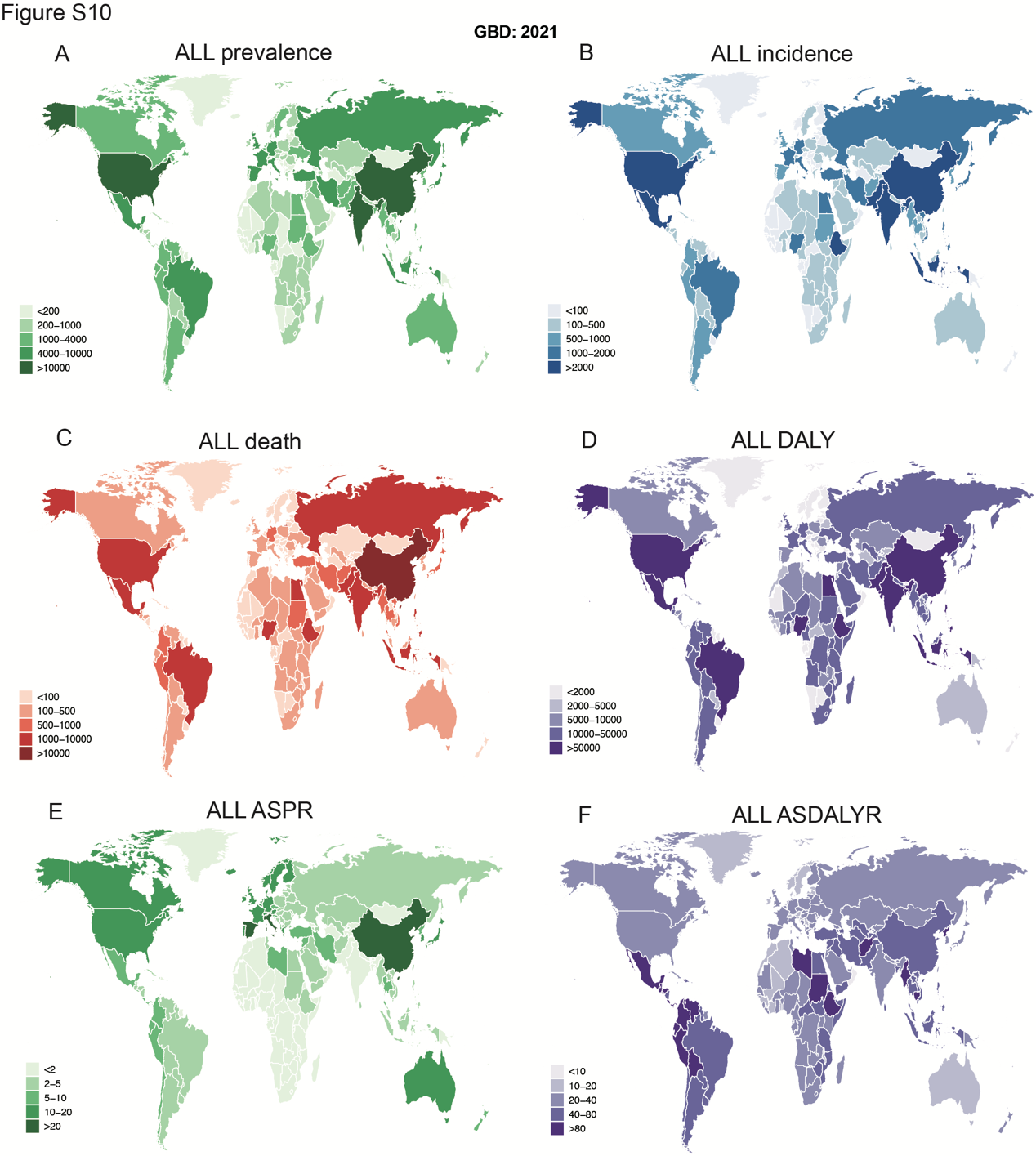


**Figure S11**: Global distribution of CLL prevalence (A), incidence (B), deaths (C), DALYs (D), ASPR (E), and ASDALYR (F) in 2021 from GBD. CLL, Chronic lymphoid leukemia; DALYs, disability-adjusted life years; ASPR, age-standardized prevalence rate; ASDALYR, age-standardized DALY rate.


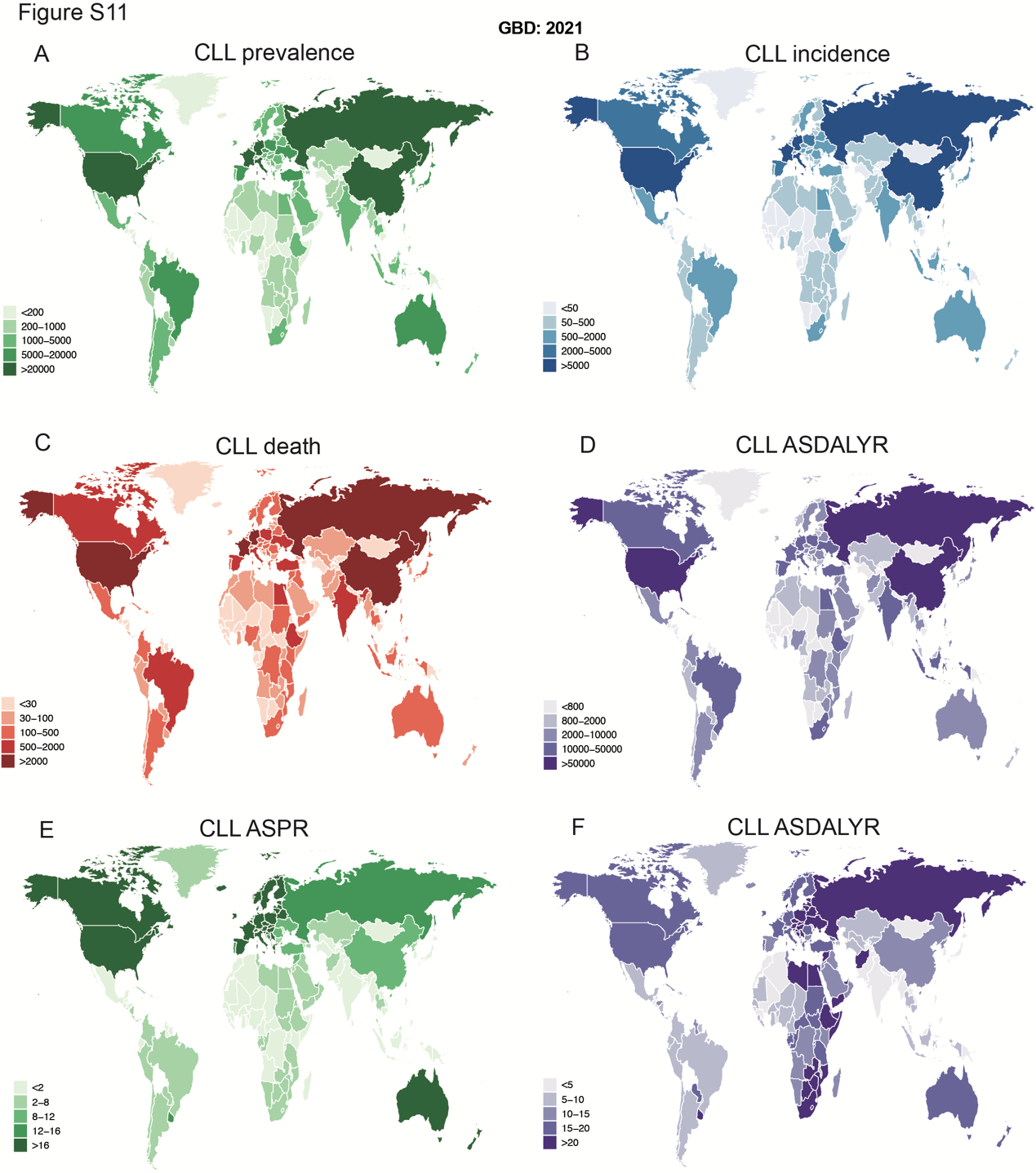


**Figure S12**: Global distribution of MM prevalence (A), incidence (B), deaths (C), DALYs (D), ASPR (E), and ASDALYR (F) in 2021 from GBD. MM, Multiple myeloma; DALYs, disability-adjusted life years; ASPR, age-standardized prevalence rate; ASDALYR, age-standardized DALY rate.


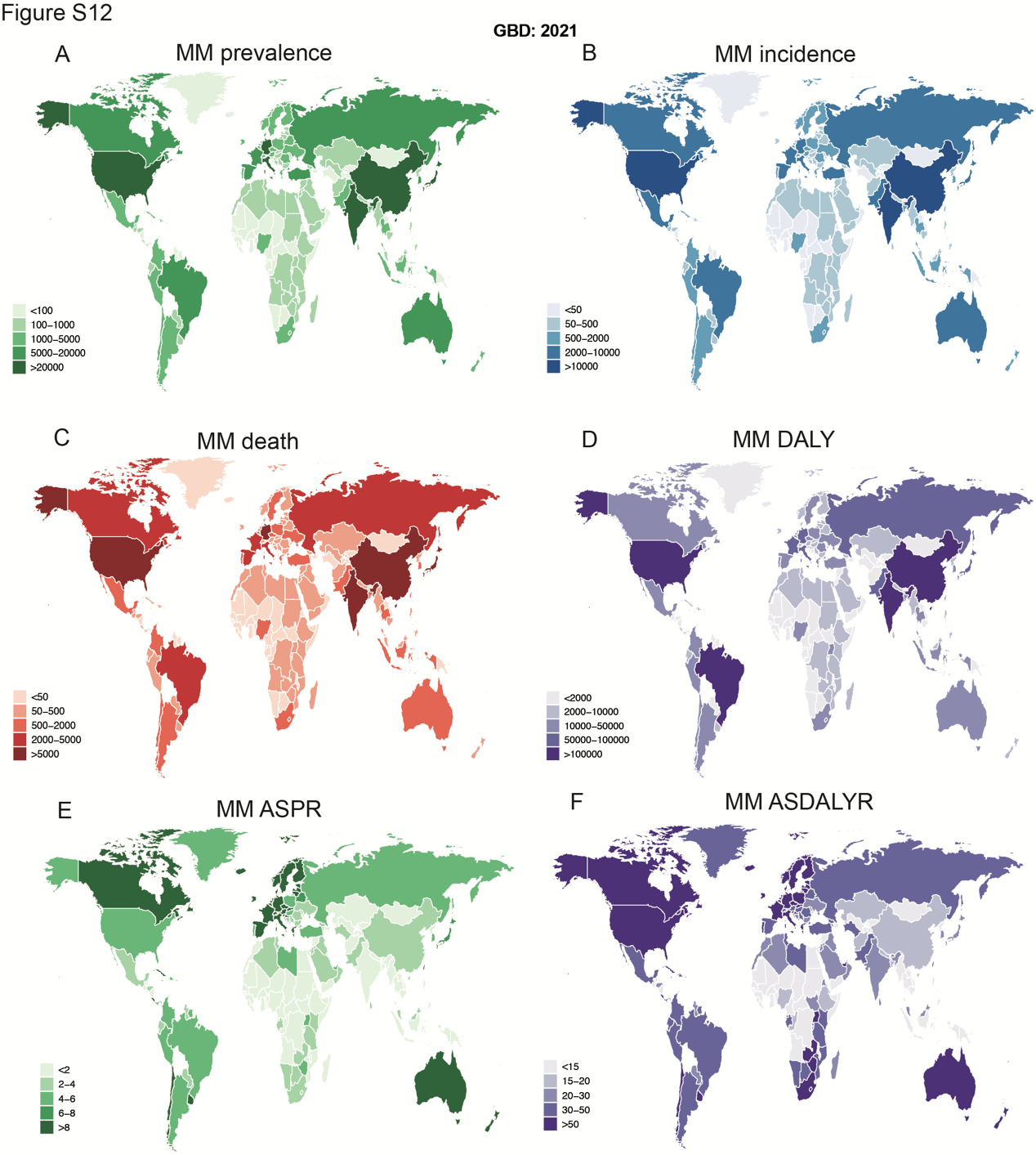


**Figure S13**: Distribution of prevalence by gender, age group, SDI regions in 2021 from GBD. (A): HL, (B): NHL, (C): AML, (D): CML, (E): ALL, (F): CLL, (G): MM. HL, Hodgkin lymphoma; NHL, Non−Hodgkin lymphoma; AML, Acute myeloid leukemia; CML, Chronic myeloid leukemia; ALL, Acute lymphoid leukemia; CLL, Chronic lymphoid leukemia; MM, Multiple myeloma; DALYs, disability-adjusted life years; SDI, socio-demographic index.


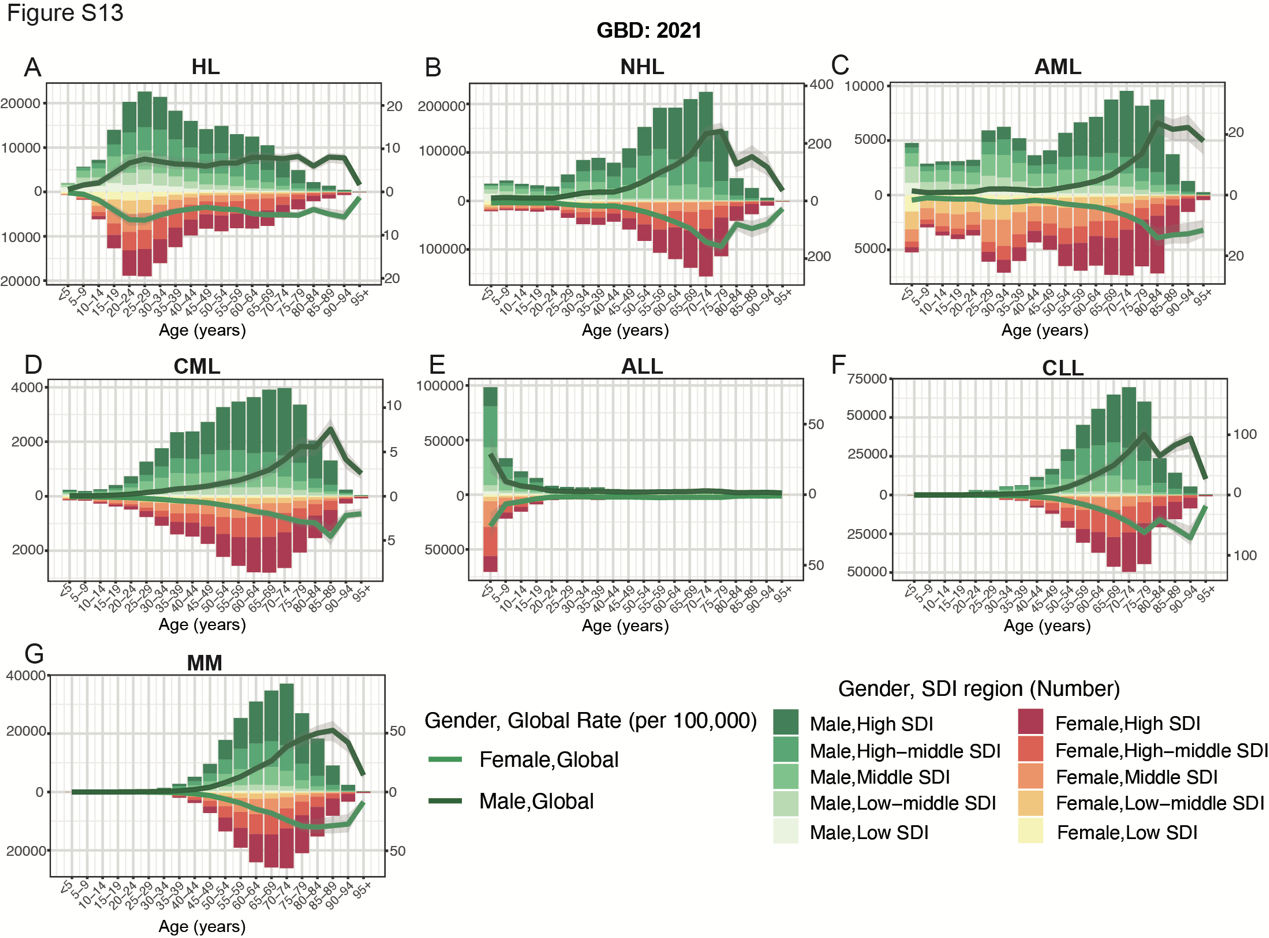


**Figure S14**: Global trends in ASPR in 1990-2021 from GBD. The Rho values indicate the strength of the correlation between the ASPR and SDI in HL (A), NHL (B), AML (C), CML (D), ALL (E), CLL (F), and MM (G). HL, Hodgkin lymphoma; NHL, Non−Hodgkin lymphoma; AML, Acute myeloid leukemia; CML, Chronic myeloid leukemia; ALL, Acute lymphoid leukemia; CLL, Chronic lymphoid leukemia; MM, Multiple myeloma; DALYs, disability-adjusted life years; ASPR, age-standardized prevalence rate; ASIR, age-standardized incidence rate; ASDR, age-standardized death rate; ASDALYR, age-standardized DALY rate; SDI, socio-demographic index.


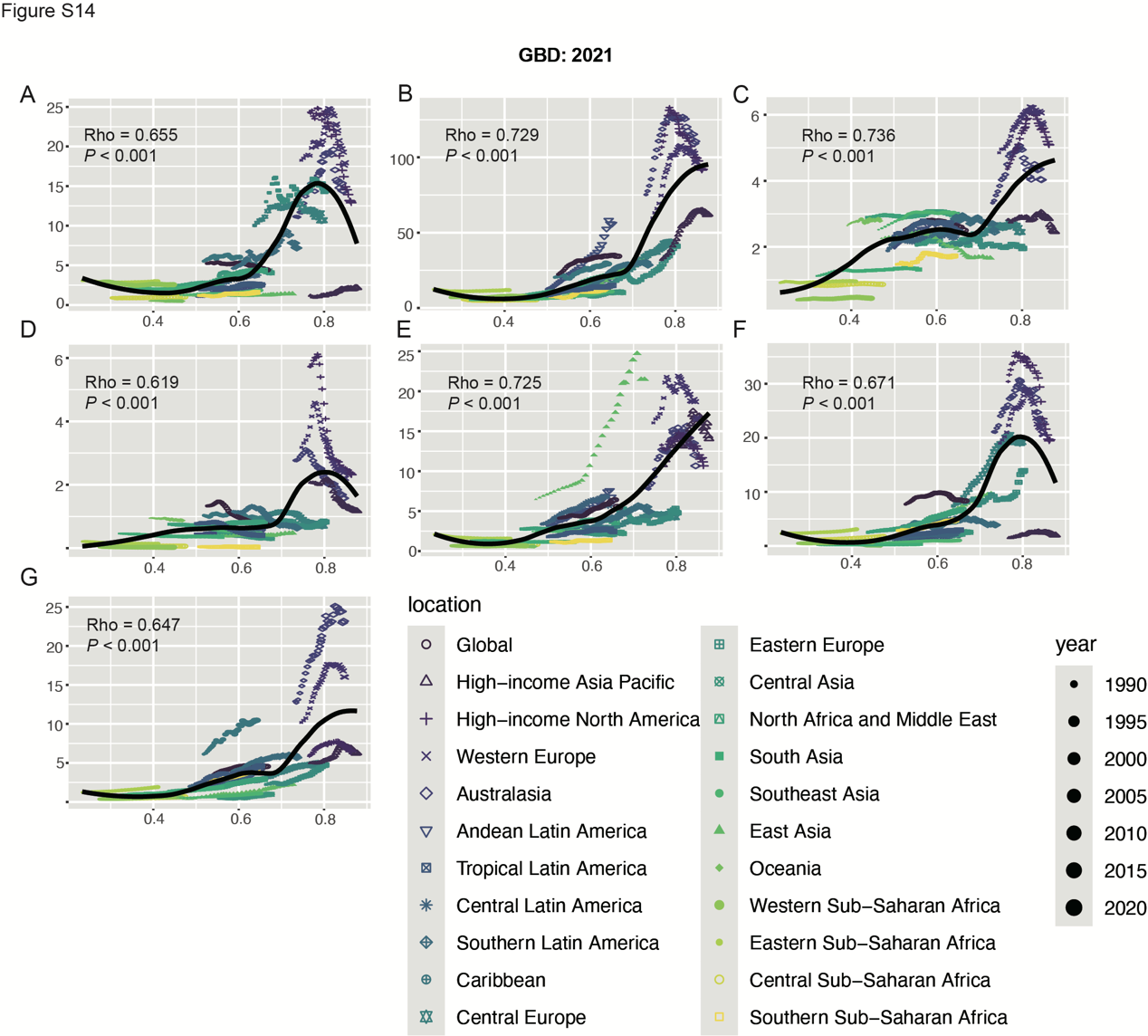


**Figure S15**: Future forecasts of GBD in ASIR and ASDALYR in 2021-2040 by gender. (A): HL, (B): NHL, (C): AML, (D): CML, (E): ALL, (F): CLL, (G): MM. HL, Hodgkin lymphoma; NHL, Non−Hodgkin lymphoma; AML, Acute myeloid leukemia; CML, Chronic myeloid leukemia; ALL, Acute lymphoid leukemia; CLL, Chronic lymphoid leukemia; MM, Multiple myeloma; ASIR, age-standardized incidence rate; ASDALYR, age-standardized DALY rate.


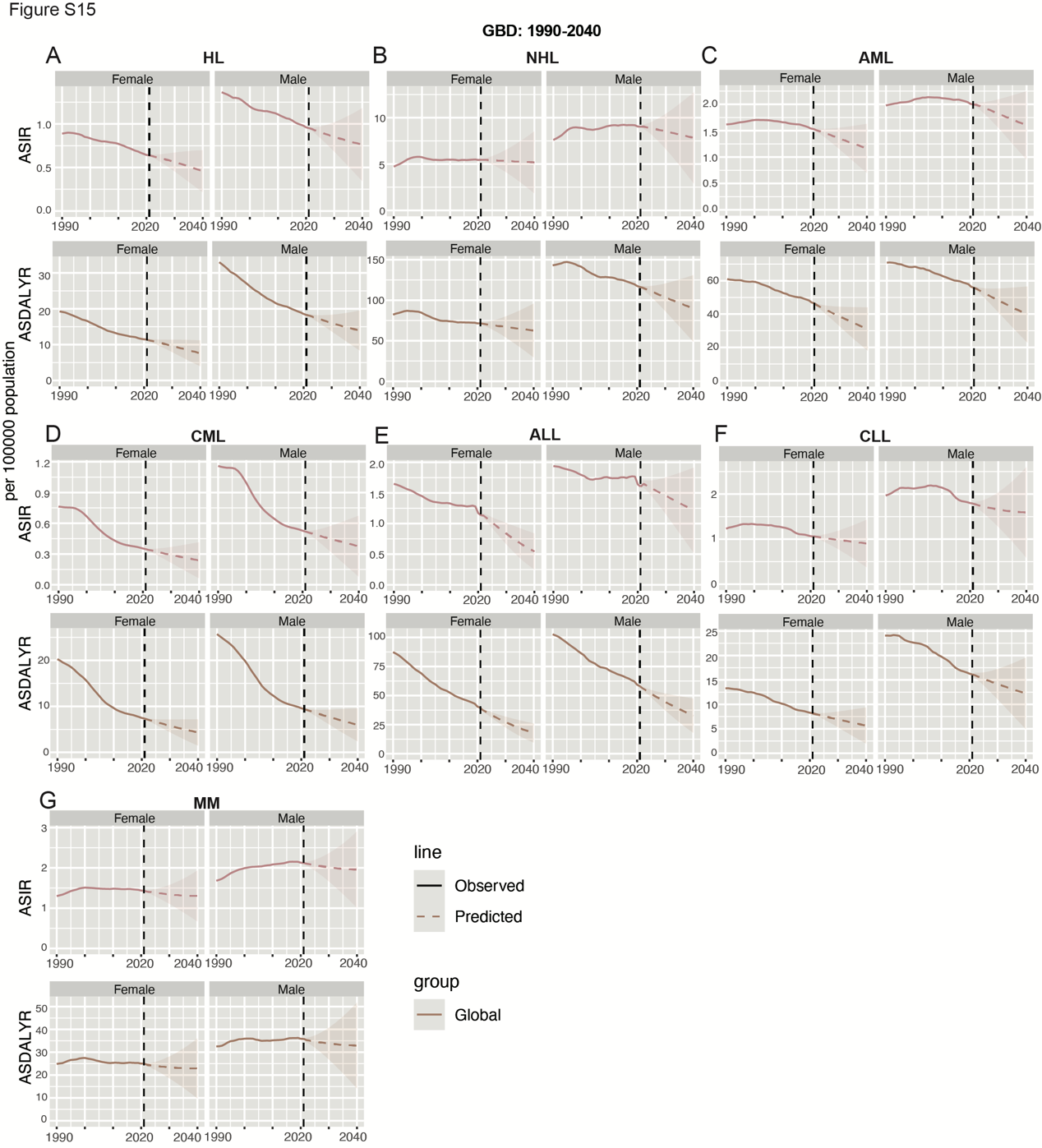

Supplement: Supplementary file 1 — Supplementary Material 1 [file 40164_2025_684_MOESM1_ESM.docx]
