# Supplementary material for: Global burden and trends of hematologic malignancies based on Global Cancer Observatory 2022 and Global Burden of Disease 2021
Source: Exp Hematol Oncol. 2025 Jul 17;14:98. doi: 10.1186/s40164-025-00684-x (PMC12273037; doi:10.1186/s40164-025-00684-x)
Supplement: Supplementary file 2 — Supplementary Material 2 [file 40164_2025_684_MOESM2_ESM.docx]

**Table S1.** Summary of the crucial disparities between GBD 2021 and GLOBOCAN 2022.

|  | **GBD 2021** | **GLOBOCAN 2022** |
| --- | --- | --- |
| **Organization** | Institute for Health Metrics and Evaluation | International Agency for Research on Cancer |
| **Region** | 204 countries and territories | 185 countries |
| **Years** | 1990-2021 | 2022 |
| **Age groups** | 23 age groups from birth to age 95 years and older | 18 age groups from birth to age 85 years and older |
| **Hematologic malignancies coverage (ICD-10 code)** | Hodgkin lymphoma (C81), non-Hodgkin lymphoma (C82-C86, C88), acute myeloid leukemia (C92.0), chronic myeloid leukemia (C92.1), acute lymphoid leukemia (C91.0), chronic lymphoid leukemia (C91.1), and multiple myeloma (C90). | Hodgkin lymphoma(C81), non-Hodgkin lymphoma (C82-86, C88), multiple myeloma (C90), and leukaemia (C91-95). |
| **Methodology** | using two Bayesian statistical models (spatiotemporal Gaussian process regression [ST-GPR] and disease model meta-regression [DisMod-MR 2.1]) to pool heterogeneous data and to control and adjust for bias. | Short-term prediction models and mortality-to-incidence ratios (MIRs); relies on regional and neighboring country data when local data is unavailable. |
| **Data Sources** | Comprehensive sources: cancer registries, vital registration systems, hospital records, clinical data, SEER data, and scientific literature. | Mainly relies on national and subnational cancer registries; proxies used for countries with limited data. |
| **Data Accessibility** | Long-term trends in Prevalence, Incidence, Deaths, DALYs; Risk factors; Socio-demographic Index. | Incidence and mortality for 2022. |
