# Supplementary material for: Global burden and trends of hematologic malignancies based on Global Cancer Observatory 2022 and Global Burden of Disease 2021
Source: Exp Hematol Oncol. 2025 Jul 17;14:98. doi: 10.1186/s40164-025-00684-x (PMC12273037; doi:10.1186/s40164-025-00684-x)
Supplement: Supplementary file 4 — Supplementary Material 4 [file 40164_2025_684_MOESM4_ESM.docx]

setwd("/data")

library(dplyr)

library(purrr)

library(openxlsx)

library(data.table)

library(vroom)

path = "/data"

fileName = dir(path)

fileName

newdata<-data.frame()

for(k in 1:length(fileName)){

data = vroom(file = paste(path,fileName[k],sep = "/"))

newdata=rbind(newdata,data)

}

write.csv(newdata,"total.csv")

#####Global+5 SDI regions+21 regions

HM_region <-subset(newdata,(newdata$location_name == 'High-income Asia Pacific'|

newdata$location_name == 'Central Asia'|

newdata$location_name == 'Southeast Asia'|

newdata$location_name == 'East Asia'|

newdata$location_name == 'Central Europe'|

newdata$location_name == 'Eastern Europe'|

newdata$location_name == 'North Africa and Middle East'|

newdata$location_name == 'Australasia'|

newdata$location_name == 'Western Europe'|

newdata$location_name == 'Andean Latin America'|

newdata$location_name == 'Caribbean'|

newdata$location_name == 'High-income North America'|

newdata$location_name == 'Western Sub-Saharan Africa'|

newdata$location_name == 'South Asia'|

newdata$location_name == 'Oceania'|

newdata$location_name == 'Central Sub-Saharan Africa'|

newdata$location_name == 'Central Latin America'|

newdata$location_name == 'Southern Latin America'|

newdata$location_name == 'Tropical Latin America'|

newdata$location_name == 'Eastern Sub-Saharan Africa'|

newdata$location_name == 'Southern Sub-Saharan Africa'|

newdata$location_name == 'Global'|

newdata$location_name == 'High SDI'|

newdata$location_name == 'High-middle SDI'|

newdata$location_name == 'Middle SDI'|

newdata$location_name == 'Low-middle SDI'|

newdata$location_name == 'Low SDI'

))

unique(HM_region$location_name)###check

write.csv(HM_region,"HM_region.csv")

####204 country

HM_country <-subset(newdata,(newdata$location_name != 'Central Europe, Eastern Europe, and Central Asia'&

newdata$location_name != 'High-income'&

newdata$location_name != 'Latin America and Caribbean'&

newdata$location_name != 'North Africa and Middle East'&

newdata$location_name != 'South Asia'&

newdata$location_name != 'Southeast Asia, East Asia, and Oceania'&

newdata$location_name != 'Sub-Saharan Africa'&

newdata$location_name != 'Global'&

newdata$location_name != 'High-income Asia Pacific'&

newdata$location_name != 'Central Asia'&

newdata$location_name != 'Southeast Asia'&

newdata$location_name != 'East Asia'&

newdata$location_name != 'Central Europe'&

newdata$location_name != 'Eastern Europe'&

newdata$location_name != 'North Africa and Middle East'&

newdata$location_name != 'Australasia'&

newdata$location_name != 'Western Europe'&

newdata$location_name != 'Andean Latin America'&

newdata$location_name != 'Caribbean'&

newdata$location_name != 'High-income North America'&

newdata$location_name != 'Western Sub-Saharan Africa'&

newdata$location_name != 'South Asia'&

newdata$location_name != 'Oceania'&

newdata$location_name != 'Central Sub-Saharan Africa'&

newdata$location_name != 'Central Latin America'&

newdata$location_name != 'Southern Latin America'&

newdata$location_name != 'Tropical Latin America'&

newdata$location_name != 'Eastern Sub-Saharan Africa'&

newdata$location_name != 'Southern Sub-Saharan Africa'))

unique(HM_country$location_name)###check

write.csv(HM_country,"HM_country.csv")

#Figure 1A-H

library(dplyr)

library(ggplot2)

library(ggsci)

library(scales)

region<- vroom::vroom("/data.csv")

IS <- region %>%

dplyr::select(measure_name,location_name,metric_name,cause_name,

sex_name,age_name,year,val,lower,upper) %>%

rename(measure=measure_name,

location=location_name,

metric=metric_name,

sex=sex_name, cause=cause_name

age=age_name)

IS_1 <- IS %>%

filter(age=="All ages" &

metric=="Number") %>%

filter(cause=="Hodgkin lymphoma" |

cause=="Non-Hodgkin lymphoma" |

cause=="Acute myeloid leukemia" |

cause=="Chronic myeloid leukemia" |

cause=="Acute lymphoid leukemia" |

cause=="Chronic lymphoid leukemia" |

cause=="Multiple myeloma" )

IS_1$group <- ifelse(

IS_1$sex=="Both"&IS_1$measure=="Prevalence",'a',ifelse(

IS_1$sex=="Both"&IS_1$measure=="Incidence",'b',ifelse(

IS_1$sex=="Both"&IS_1$measure=="Deaths",'c',ifelse(

IS_1$sex=="Both"&IS_1$measure=="DALYs (Disability-Adjusted Life Years)",'d',ifelse(

)))))

p <- ggplot(data=IS_1,aes(x=year,y=val)) +

geom_line(aes(color=location)) +

geom_point(aes(color=location)) +

scale_color_manual(values=pal_lancet(palette = c("lanonc"), alpha = 1)(7)[c(1,3,2,5,6,4,7)]) +

facet_wrap(~group,scales="free_y",

ncol = 4,

labeller = labeller(group=c(

a="Prevalence",

b="Incidence",

c="Deaths",

d="DALYs"

)))+

theme_bw() +

theme(

strip.text.x = element_text(size = 10,

color = "#ada072",

face = "bold"),

strip.background = element_rect(colour = "black",

fill = "#eeecdf"),

axis.title = element_blank(),

legend.title = element_blank(),

legend.position = "top") +

guides(

color = guide_legend(

nrow= 1,

byrow= T

)

)

p

IS_1 <- IS %>%

filter(age=="Age-standardized" &

metric=="Rate") %>%

filter(cause=="Hodgkin lymphoma" |

cause=="Non-Hodgkin lymphoma" |

cause=="Acute myeloid leukemia" |

cause=="Chronic myeloid leukemia" |

cause=="Acute lymphoid leukemia" |

cause=="Chronic lymphoid leukemia" |

cause=="Multiple myeloma" )

IS_1$group <- ifelse(

IS_1$sex=="Both"&IS_1$measure=="Prevalence",'a',ifelse(

IS_1$sex=="Both"&IS_1$measure=="Incidence",'b',ifelse(

IS_1$sex=="Both"&IS_1$measure=="Deaths",'c',ifelse(

IS_1$sex=="Both"&IS_1$measure=="DALYs (Disability-Adjusted Life Years)",'d',ifelse(

)))))

p <- ggplot(data=IS_1,aes(x=year,y=val)) +

geom_line(aes(color=location)) +

geom_point(aes(color=location)) +

scale_color_manual(values=pal_lancet(palette = c("lanonc"), alpha = 1)(7)[c(1,3,2,5,6,4,7)]) +

facet_wrap(~group,scales="free_y",

ncol = 4,

labeller = labeller(group=c(

a="ASPR per 100,000",

b="ASIR per 100,000",

c="ASDR per 100,000",

d="ASDALYR per 100,000"

)))+

theme_bw() +

theme(

strip.text.x = element_text(size = 10,

color = "#ada072",

face = "bold"),

strip.background = element_rect(colour = "black",

fill = "#eeecdf"),

axis.title = element_blank(),

legend.title = element_blank(),

legend.position = "top") +

guides(

color = guide_legend(

nrow= 1,

byrow= T

)

)

p

#Figure 2; Figure S1-S5

library(dplyr)

library(ggplot2)

library(ggsci)

library(scales)

region<- vroom::vroom("/HL_region.csv")

IS <- region %>%

dplyr::select(measure_name,location_name,metric_name,cause_name,

sex_name,age_name,year,val,lower,upper) %>%

rename(measure=measure_name,

location=location_name,

metric=metric_name,

sex=sex_name, cause=cause_name

age=age_name)

IS_1 <- IS %>%

filter(age=="All ages" &

metric=="Number") %>%

filter(location=="Global" |

location=="High SDI" |

location=="High-middle SDI" |

location=="Middle SDI" |

location=="Low-middle SDI" |

location=="Low SDI")

IS_1$group <- ifelse(

IS_1$sex=="Both"&IS_1$measure=="Prevalence",'a',ifelse(

IS_1$sex=="Both"&IS_1$measure=="Incidence",'b',ifelse(

IS_1$sex=="Both"&IS_1$measure=="Deaths",'c',ifelse(

IS_1$sex=="Both"&IS_1$measure=="DALYs (Disability-Adjusted Life Years)",'d',ifelse(

IS_1$sex=="Male"&IS_1$measure=="Prevalence",'e',ifelse(

IS_1$sex=="Male"&IS_1$measure=="Incidence",'f',ifelse(

IS_1$sex=="Male"&IS_1$measure=="Deaths",'g',ifelse(

IS_1$sex=="Male"&IS_1$measure=="DALYs (Disability-Adjusted Life Years)",'h',ifelse(

IS_1$sex=="Female"&IS_1$measure=="Prevalence",'i',ifelse(

IS_1$sex=="Female"&IS_1$measure=="Incidence",'j',ifelse(

IS_1$sex=="Female"&IS_1$measure=="Deaths",'k','l')))))))))))

p <- ggplot(data=IS_1,aes(x=year,y=val)) +

geom_line(aes(color=location)) +

geom_point(aes(color=location)) +

scale_color_manual(values=pal_lancet(palette = c("lanonc"), alpha = 1)(6)[c(1,3,2,5,6,4)]) +

facet_wrap(~group,scales="free_y",

ncol = 4,

labeller = labeller(group=c(

a="Both-Prevalence",

b="Both-Incidence",

c="Both-Deaths",

d="Both-DALYs",

e="Male-Prevalence",

f="Male-Incidence",

g="Male-Deaths",

h="Male-DALYs",

i="Female-Prevalence",

j="Female-Incidence",

k="Female-Deaths",

l="Female-DALYs"

)))+

theme_bw() +

theme(

strip.text.x = element_text(size = 10,

color = "#ada072",

face = "bold"),

strip.background = element_rect(colour = "black",

fill = "#eeecdf"),

axis.title = element_blank(),

legend.title = element_blank(),

legend.position = "top") +

guides(

color = guide_legend(

nrow= 1,

byrow= T

)

)

p

IS_1 <- IS %>%

filter(age=="Age-standardized" &

metric=="Rate") %>%

filter(location=="Global" |

location=="High SDI" |

location=="High-middle SDI" |

location=="Middle SDI" |

location=="Low-middle SDI" |

location=="Low SDI")

IS_1$group <- ifelse(

IS_1$sex=="Both"&IS_1$measure=="Prevalence",'a',ifelse(

IS_1$sex=="Both"&IS_1$measure=="Incidence",'b',ifelse(

IS_1$sex=="Both"&IS_1$measure=="Deaths",'c',ifelse(

IS_1$sex=="Both"&IS_1$measure=="DALYs (Disability-Adjusted Life Years)",'d',ifelse(

IS_1$sex=="Male"&IS_1$measure=="Prevalence",'e',ifelse(

IS_1$sex=="Male"&IS_1$measure=="Incidence",'f',ifelse(

IS_1$sex=="Male"&IS_1$measure=="Deaths",'g',ifelse(

IS_1$sex=="Male"&IS_1$measure=="DALYs (Disability-Adjusted Life Years)",'h',ifelse(

IS_1$sex=="Female"&IS_1$measure=="Prevalence",'i',ifelse(

IS_1$sex=="Female"&IS_1$measure=="Incidence",'j',ifelse(

IS_1$sex=="Female"&IS_1$measure=="Deaths",'k','l')))))))))))

p <- ggplot(data=IS_1,aes(x=year,y=val)) +

geom_line(aes(color=location)) +

geom_point(aes(color=location)) +

scale_color_manual(values=pal_lancet(palette = c("lanonc"), alpha = 1)(6)[c(1,3,2,5,6,4)]) +

facet_wrap(~group,scales="free_y",

ncol = 4,

labeller = labeller(group=c(

a="Both-ASPR per 100,000",

b="Both-ASIR per 100,000",

c="Both-ASDR per 100,000",

d="Both-ASDALYR per 100,000",

e="Male-ASPR per 100,000",

f="Male-ASIR per 100,000",

g="Male-ASDR per 100,000",

h="Male-ASDALYR per 100,000",

i="Female-ASPR per 100,000",

j="Female-ASIR per 100,000",

k="Female-ASDR per 100,000",

l="Female-ASDALYR per 100,000"

)))+

theme_bw() +

theme(

strip.text.x = element_text(size = 10,

color = "#ada072",

face = "bold"),

strip.background = element_rect(colour = "black",

fill = "#eeecdf"),

axis.title = element_blank(),

legend.title = element_blank(),

legend.position = "top") +

guides(

color = guide_legend(

nrow= 1,

byrow= T

)

)

p

region<- vroom::vroom("/NHL_region.csv")

IS <- region %>%

dplyr::select(measure_name,location_name,metric_name,cause_name,

sex_name,age_name,year,val,lower,upper) %>%

rename(measure=measure_name,

location=location_name,

metric=metric_name,

sex=sex_name, cause=cause_name

age=age_name)

IS_1 <- IS %>%

filter(age=="All ages" &

metric=="Number") %>%

filter(location=="Global" |

location=="High SDI" |

location=="High-middle SDI" |

location=="Middle SDI" |

location=="Low-middle SDI" |

location=="Low SDI")

IS_1$group <- ifelse(

IS_1$sex=="Both"&IS_1$measure=="Prevalence",'a',ifelse(

IS_1$sex=="Both"&IS_1$measure=="Incidence",'b',ifelse(

IS_1$sex=="Both"&IS_1$measure=="Deaths",'c',ifelse(

IS_1$sex=="Both"&IS_1$measure=="DALYs (Disability-Adjusted Life Years)",'d',ifelse(

IS_1$sex=="Male"&IS_1$measure=="Prevalence",'e',ifelse(

IS_1$sex=="Male"&IS_1$measure=="Incidence",'f',ifelse(

IS_1$sex=="Male"&IS_1$measure=="Deaths",'g',ifelse(

IS_1$sex=="Male"&IS_1$measure=="DALYs (Disability-Adjusted Life Years)",'h',ifelse(

IS_1$sex=="Female"&IS_1$measure=="Prevalence",'i',ifelse(

IS_1$sex=="Female"&IS_1$measure=="Incidence",'j',ifelse(

IS_1$sex=="Female"&IS_1$measure=="Deaths",'k','l')))))))))))

p <- ggplot(data=IS_1,aes(x=year,y=val)) +

geom_line(aes(color=location)) +

geom_point(aes(color=location)) +

scale_color_manual(values=pal_lancet(palette = c("lanonc"), alpha = 1)(6)[c(1,3,2,5,6,4)]) +

facet_wrap(~group,scales="free_y",

ncol = 4,

labeller = labeller(group=c(

a="Both-Prevalence",

b="Both-Incidence",

c="Both-Deaths",

d="Both-DALYs",

e="Male-Prevalence",

f="Male-Incidence",

g="Male-Deaths",

h="Male-DALYs",

i="Female-Prevalence",

j="Female-Incidence",

k="Female-Deaths",

l="Female-DALYs"

)))+

theme_bw() +

theme(

strip.text.x = element_text(size = 10,

color = "#ada072",

face = "bold"),

strip.background = element_rect(colour = "black",

fill = "#eeecdf"),

axis.title = element_blank(),

legend.title = element_blank(),

legend.position = "top") +

guides(

color = guide_legend(

nrow= 1,

byrow= T

)

)

p

IS_1 <- IS %>%

filter(age=="Age-standardized" &

metric=="Rate") %>%

filter(location=="Global" |

location=="High SDI" |

location=="High-middle SDI" |

location=="Middle SDI" |

location=="Low-middle SDI" |

location=="Low SDI")

IS_1$group <- ifelse(

IS_1$sex=="Both"&IS_1$measure=="Prevalence",'a',ifelse(

IS_1$sex=="Both"&IS_1$measure=="Incidence",'b',ifelse(

IS_1$sex=="Both"&IS_1$measure=="Deaths",'c',ifelse(

IS_1$sex=="Both"&IS_1$measure=="DALYs (Disability-Adjusted Life Years)",'d',ifelse(

IS_1$sex=="Male"&IS_1$measure=="Prevalence",'e',ifelse(

IS_1$sex=="Male"&IS_1$measure=="Incidence",'f',ifelse(

IS_1$sex=="Male"&IS_1$measure=="Deaths",'g',ifelse(

IS_1$sex=="Male"&IS_1$measure=="DALYs (Disability-Adjusted Life Years)",'h',ifelse(

IS_1$sex=="Female"&IS_1$measure=="Prevalence",'i',ifelse(

IS_1$sex=="Female"&IS_1$measure=="Incidence",'j',ifelse(

IS_1$sex=="Female"&IS_1$measure=="Deaths",'k','l')))))))))))

p <- ggplot(data=IS_1,aes(x=year,y=val)) +

geom_line(aes(color=location)) +

geom_point(aes(color=location)) +

scale_color_manual(values=pal_lancet(palette = c("lanonc"), alpha = 1)(6)[c(1,3,2,5,6,4)]) +

facet_wrap(~group,scales="free_y",

ncol = 4,

labeller = labeller(group=c(

a="Both-ASPR per 100,000",

b="Both-ASIR per 100,000",

c="Both-ASDR per 100,000",

d="Both-ASDALYR per 100,000",

e="Male-ASPR per 100,000",

f="Male-ASIR per 100,000",

g="Male-ASDR per 100,000",

h="Male-ASDALYR per 100,000",

i="Female-ASPR per 100,000",

j="Female-ASIR per 100,000",

k="Female-ASDR per 100,000",

l="Female-ASDALYR per 100,000"

)))+

theme_bw() +

theme(

strip.text.x = element_text(size = 10,

color = "#ada072",

face = "bold"),

strip.background = element_rect(colour = "black",

fill = "#eeecdf"),

axis.title = element_blank(),

legend.title = element_blank(),

legend.position = "top") +

guides(

color = guide_legend(

nrow= 1,

byrow= T

)

)

p

region<- vroom::vroom("/AML_region.csv")

IS <- region %>%

dplyr::select(measure_name,location_name,metric_name,cause_name,

sex_name,age_name,year,val,lower,upper) %>%

rename(measure=measure_name,

location=location_name,

metric=metric_name,

sex=sex_name, cause=cause_name

age=age_name)

IS_1 <- IS %>%

filter(age=="All ages" &

metric=="Number") %>%

filter(location=="Global" |

location=="High SDI" |

location=="High-middle SDI" |

location=="Middle SDI" |

location=="Low-middle SDI" |

location=="Low SDI")

IS_1$group <- ifelse(

IS_1$sex=="Both"&IS_1$measure=="Prevalence",'a',ifelse(

IS_1$sex=="Both"&IS_1$measure=="Incidence",'b',ifelse(

IS_1$sex=="Both"&IS_1$measure=="Deaths",'c',ifelse(

IS_1$sex=="Both"&IS_1$measure=="DALYs (Disability-Adjusted Life Years)",'d',ifelse(

IS_1$sex=="Male"&IS_1$measure=="Prevalence",'e',ifelse(

IS_1$sex=="Male"&IS_1$measure=="Incidence",'f',ifelse(

IS_1$sex=="Male"&IS_1$measure=="Deaths",'g',ifelse(

IS_1$sex=="Male"&IS_1$measure=="DALYs (Disability-Adjusted Life Years)",'h',ifelse(

IS_1$sex=="Female"&IS_1$measure=="Prevalence",'i',ifelse(

IS_1$sex=="Female"&IS_1$measure=="Incidence",'j',ifelse(

IS_1$sex=="Female"&IS_1$measure=="Deaths",'k','l')))))))))))

p <- ggplot(data=IS_1,aes(x=year,y=val)) +

geom_line(aes(color=location)) +

geom_point(aes(color=location)) +

scale_color_manual(values=pal_lancet(palette = c("lanonc"), alpha = 1)(6)[c(1,3,2,5,6,4)]) +

facet_wrap(~group,scales="free_y",

ncol = 4,

labeller = labeller(group=c(

a="Both-Prevalence",

b="Both-Incidence",

c="Both-Deaths",

d="Both-DALYs",

e="Male-Prevalence",

f="Male-Incidence",

g="Male-Deaths",

h="Male-DALYs",

i="Female-Prevalence",

j="Female-Incidence",

k="Female-Deaths",

l="Female-DALYs"

)))+

theme_bw() +

theme(

strip.text.x = element_text(size = 10,

color = "#ada072",

face = "bold"),

strip.background = element_rect(colour = "black",

fill = "#eeecdf"),

axis.title = element_blank(),

legend.title = element_blank(),

legend.position = "top") +

guides(

color = guide_legend(

nrow= 1,

byrow= T

)

)

p

IS_1 <- IS %>%

filter(age=="Age-standardized" &

metric=="Rate") %>%

filter(location=="Global" |

location=="High SDI" |

location=="High-middle SDI" |

location=="Middle SDI" |

location=="Low-middle SDI" |

location=="Low SDI")

IS_1$group <- ifelse(

IS_1$sex=="Both"&IS_1$measure=="Prevalence",'a',ifelse(

IS_1$sex=="Both"&IS_1$measure=="Incidence",'b',ifelse(

IS_1$sex=="Both"&IS_1$measure=="Deaths",'c',ifelse(

IS_1$sex=="Both"&IS_1$measure=="DALYs (Disability-Adjusted Life Years)",'d',ifelse(

IS_1$sex=="Male"&IS_1$measure=="Prevalence",'e',ifelse(

IS_1$sex=="Male"&IS_1$measure=="Incidence",'f',ifelse(

IS_1$sex=="Male"&IS_1$measure=="Deaths",'g',ifelse(

IS_1$sex=="Male"&IS_1$measure=="DALYs (Disability-Adjusted Life Years)",'h',ifelse(

IS_1$sex=="Female"&IS_1$measure=="Prevalence",'i',ifelse(

IS_1$sex=="Female"&IS_1$measure=="Incidence",'j',ifelse(

IS_1$sex=="Female"&IS_1$measure=="Deaths",'k','l')))))))))))

p <- ggplot(data=IS_1,aes(x=year,y=val)) +

geom_line(aes(color=location)) +

geom_point(aes(color=location)) +

scale_color_manual(values=pal_lancet(palette = c("lanonc"), alpha = 1)(6)[c(1,3,2,5,6,4)]) +

facet_wrap(~group,scales="free_y",

ncol = 4,

labeller = labeller(group=c(

a="Both-ASPR per 100,000",

b="Both-ASIR per 100,000",

c="Both-ASDR per 100,000",

d="Both-ASDALYR per 100,000",

e="Male-ASPR per 100,000",

f="Male-ASIR per 100,000",

g="Male-ASDR per 100,000",

h="Male-ASDALYR per 100,000",

i="Female-ASPR per 100,000",

j="Female-ASIR per 100,000",

k="Female-ASDR per 100,000",

l="Female-ASDALYR per 100,000"

)))+

theme_bw() +

theme(

strip.text.x = element_text(size = 10,

color = "#ada072",

face = "bold"),

strip.background = element_rect(colour = "black",

fill = "#eeecdf"),

axis.title = element_blank(),

legend.title = element_blank(),

legend.position = "top") +

guides(

color = guide_legend(

nrow= 1,

byrow= T

)

)

p

region<- vroom::vroom("/CML_region.csv")

IS <- region %>%

dplyr::select(measure_name,location_name,metric_name,cause_name,

sex_name,age_name,year,val,lower,upper) %>%

rename(measure=measure_name,

location=location_name,

metric=metric_name,

sex=sex_name, cause=cause_name

age=age_name)

IS_1 <- IS %>%

filter(age=="All ages" &

metric=="Number") %>%

filter(location=="Global" |

location=="High SDI" |

location=="High-middle SDI" |

location=="Middle SDI" |

location=="Low-middle SDI" |

location=="Low SDI")

IS_1$group <- ifelse(

IS_1$sex=="Both"&IS_1$measure=="Prevalence",'a',ifelse(

IS_1$sex=="Both"&IS_1$measure=="Incidence",'b',ifelse(

IS_1$sex=="Both"&IS_1$measure=="Deaths",'c',ifelse(

IS_1$sex=="Both"&IS_1$measure=="DALYs (Disability-Adjusted Life Years)",'d',ifelse(

IS_1$sex=="Male"&IS_1$measure=="Prevalence",'e',ifelse(

IS_1$sex=="Male"&IS_1$measure=="Incidence",'f',ifelse(

IS_1$sex=="Male"&IS_1$measure=="Deaths",'g',ifelse(

IS_1$sex=="Male"&IS_1$measure=="DALYs (Disability-Adjusted Life Years)",'h',ifelse(

IS_1$sex=="Female"&IS_1$measure=="Prevalence",'i',ifelse(

IS_1$sex=="Female"&IS_1$measure=="Incidence",'j',ifelse(

IS_1$sex=="Female"&IS_1$measure=="Deaths",'k','l')))))))))))

p <- ggplot(data=IS_1,aes(x=year,y=val)) +

geom_line(aes(color=location)) +

geom_point(aes(color=location)) +

scale_color_manual(values=pal_lancet(palette = c("lanonc"), alpha = 1)(6)[c(1,3,2,5,6,4)]) +

facet_wrap(~group,scales="free_y",

ncol = 4,

labeller = labeller(group=c(

a="Both-Prevalence",

b="Both-Incidence",

c="Both-Deaths",

d="Both-DALYs",

e="Male-Prevalence",

f="Male-Incidence",

g="Male-Deaths",

h="Male-DALYs",

i="Female-Prevalence",

j="Female-Incidence",

k="Female-Deaths",

l="Female-DALYs"

)))+

theme_bw() +

theme(

strip.text.x = element_text(size = 10,

color = "#ada072",

face = "bold"),

strip.background = element_rect(colour = "black",

fill = "#eeecdf"),

axis.title = element_blank(),

legend.title = element_blank(),

legend.position = "top") +

guides(

color = guide_legend(

nrow= 1,

byrow= T

)

)

p

IS_1 <- IS %>%

filter(age=="Age-standardized" &

metric=="Rate") %>%

filter(location=="Global" |

location=="High SDI" |

location=="High-middle SDI" |

location=="Middle SDI" |

location=="Low-middle SDI" |

location=="Low SDI")

IS_1$group <- ifelse(

IS_1$sex=="Both"&IS_1$measure=="Prevalence",'a',ifelse(

IS_1$sex=="Both"&IS_1$measure=="Incidence",'b',ifelse(

IS_1$sex=="Both"&IS_1$measure=="Deaths",'c',ifelse(

IS_1$sex=="Both"&IS_1$measure=="DALYs (Disability-Adjusted Life Years)",'d',ifelse(

IS_1$sex=="Male"&IS_1$measure=="Prevalence",'e',ifelse(

IS_1$sex=="Male"&IS_1$measure=="Incidence",'f',ifelse(

IS_1$sex=="Male"&IS_1$measure=="Deaths",'g',ifelse(

IS_1$sex=="Male"&IS_1$measure=="DALYs (Disability-Adjusted Life Years)",'h',ifelse(

IS_1$sex=="Female"&IS_1$measure=="Prevalence",'i',ifelse(

IS_1$sex=="Female"&IS_1$measure=="Incidence",'j',ifelse(

IS_1$sex=="Female"&IS_1$measure=="Deaths",'k','l')))))))))))

p <- ggplot(data=IS_1,aes(x=year,y=val)) +

geom_line(aes(color=location)) +

geom_point(aes(color=location)) +

scale_color_manual(values=pal_lancet(palette = c("lanonc"), alpha = 1)(6)[c(1,3,2,5,6,4)]) +

facet_wrap(~group,scales="free_y",

ncol = 4,

labeller = labeller(group=c(

a="Both-ASPR per 100,000",

b="Both-ASIR per 100,000",

c="Both-ASDR per 100,000",

d="Both-ASDALYR per 100,000",

e="Male-ASPR per 100,000",

f="Male-ASIR per 100,000",

g="Male-ASDR per 100,000",

h="Male-ASDALYR per 100,000",

i="Female-ASPR per 100,000",

j="Female-ASIR per 100,000",

k="Female-ASDR per 100,000",

l="Female-ASDALYR per 100,000"

)))+

theme_bw() +

theme(

strip.text.x = element_text(size = 10,

color = "#ada072",

face = "bold"),

strip.background = element_rect(colour = "black",

fill = "#eeecdf"),

axis.title = element_blank(),

legend.title = element_blank(),

legend.position = "top") +

guides(

color = guide_legend(

nrow= 1,

byrow= T

)

)

p

region<- vroom::vroom("/ALL_region.csv")

IS <- region %>%

dplyr::select(measure_name,location_name,metric_name,cause_name,

sex_name,age_name,year,val,lower,upper) %>%

rename(measure=measure_name,

location=location_name,

metric=metric_name,

sex=sex_name, cause=cause_name

age=age_name)

IS_1 <- IS %>%

filter(age=="All ages" &

metric=="Number") %>%

filter(location=="Global" |

location=="High SDI" |

location=="High-middle SDI" |

location=="Middle SDI" |

location=="Low-middle SDI" |

location=="Low SDI")

IS_1$group <- ifelse(

IS_1$sex=="Both"&IS_1$measure=="Prevalence",'a',ifelse(

IS_1$sex=="Both"&IS_1$measure=="Incidence",'b',ifelse(

IS_1$sex=="Both"&IS_1$measure=="Deaths",'c',ifelse(

IS_1$sex=="Both"&IS_1$measure=="DALYs (Disability-Adjusted Life Years)",'d',ifelse(

IS_1$sex=="Male"&IS_1$measure=="Prevalence",'e',ifelse(

IS_1$sex=="Male"&IS_1$measure=="Incidence",'f',ifelse(

IS_1$sex=="Male"&IS_1$measure=="Deaths",'g',ifelse(

IS_1$sex=="Male"&IS_1$measure=="DALYs (Disability-Adjusted Life Years)",'h',ifelse(

IS_1$sex=="Female"&IS_1$measure=="Prevalence",'i',ifelse(

IS_1$sex=="Female"&IS_1$measure=="Incidence",'j',ifelse(

IS_1$sex=="Female"&IS_1$measure=="Deaths",'k','l')))))))))))

p <- ggplot(data=IS_1,aes(x=year,y=val)) +

geom_line(aes(color=location)) +

geom_point(aes(color=location)) +

scale_color_manual(values=pal_lancet(palette = c("lanonc"), alpha = 1)(6)[c(1,3,2,5,6,4)]) +

facet_wrap(~group,scales="free_y",

ncol = 4,

labeller = labeller(group=c(

a="Both-Prevalence",

b="Both-Incidence",

c="Both-Deaths",

d="Both-DALYs",

e="Male-Prevalence",

f="Male-Incidence",

g="Male-Deaths",

h="Male-DALYs",

i="Female-Prevalence",

j="Female-Incidence",

k="Female-Deaths",

l="Female-DALYs"

)))+

theme_bw() +

theme(

strip.text.x = element_text(size = 10,

color = "#ada072",

face = "bold"),

strip.background = element_rect(colour = "black",

fill = "#eeecdf"),

axis.title = element_blank(),

legend.title = element_blank(),

legend.position = "top") +

guides(

color = guide_legend(

nrow= 1,

byrow= T

)

)

p

IS_1 <- IS %>%

filter(age=="Age-standardized" &

metric=="Rate") %>%

filter(location=="Global" |

location=="High SDI" |

location=="High-middle SDI" |

location=="Middle SDI" |

location=="Low-middle SDI" |

location=="Low SDI")

IS_1$group <- ifelse(

IS_1$sex=="Both"&IS_1$measure=="Prevalence",'a',ifelse(

IS_1$sex=="Both"&IS_1$measure=="Incidence",'b',ifelse(

IS_1$sex=="Both"&IS_1$measure=="Deaths",'c',ifelse(

IS_1$sex=="Both"&IS_1$measure=="DALYs (Disability-Adjusted Life Years)",'d',ifelse(

IS_1$sex=="Male"&IS_1$measure=="Prevalence",'e',ifelse(

IS_1$sex=="Male"&IS_1$measure=="Incidence",'f',ifelse(

IS_1$sex=="Male"&IS_1$measure=="Deaths",'g',ifelse(

IS_1$sex=="Male"&IS_1$measure=="DALYs (Disability-Adjusted Life Years)",'h',ifelse(

IS_1$sex=="Female"&IS_1$measure=="Prevalence",'i',ifelse(

IS_1$sex=="Female"&IS_1$measure=="Incidence",'j',ifelse(

IS_1$sex=="Female"&IS_1$measure=="Deaths",'k','l')))))))))))

p <- ggplot(data=IS_1,aes(x=year,y=val)) +

geom_line(aes(color=location)) +

geom_point(aes(color=location)) +

scale_color_manual(values=pal_lancet(palette = c("lanonc"), alpha = 1)(6)[c(1,3,2,5,6,4)]) +

facet_wrap(~group,scales="free_y",

ncol = 4,

labeller = labeller(group=c(

a="Both-ASPR per 100,000",

b="Both-ASIR per 100,000",

c="Both-ASDR per 100,000",

d="Both-ASDALYR per 100,000",

e="Male-ASPR per 100,000",

f="Male-ASIR per 100,000",

g="Male-ASDR per 100,000",

h="Male-ASDALYR per 100,000",

i="Female-ASPR per 100,000",

j="Female-ASIR per 100,000",

k="Female-ASDR per 100,000",

l="Female-ASDALYR per 100,000"

)))+

theme_bw() +

theme(

strip.text.x = element_text(size = 10,

color = "#ada072",

face = "bold"),

strip.background = element_rect(colour = "black",

fill = "#eeecdf"),

axis.title = element_blank(),

legend.title = element_blank(),

legend.position = "top") +

guides(

color = guide_legend(

nrow= 1,

byrow= T

)

)

p

region<- vroom::vroom("/CLL_region.csv")

IS <- region %>%

dplyr::select(measure_name,location_name,metric_name,cause_name,

sex_name,age_name,year,val,lower,upper) %>%

rename(measure=measure_name,

location=location_name,

metric=metric_name,

sex=sex_name, cause=cause_name

age=age_name)

IS_1 <- IS %>%

filter(age=="All ages" &

metric=="Number") %>%

filter(location=="Global" |

location=="High SDI" |

location=="High-middle SDI" |

location=="Middle SDI" |

location=="Low-middle SDI" |

location=="Low SDI")

IS_1$group <- ifelse(

IS_1$sex=="Both"&IS_1$measure=="Prevalence",'a',ifelse(

IS_1$sex=="Both"&IS_1$measure=="Incidence",'b',ifelse(

IS_1$sex=="Both"&IS_1$measure=="Deaths",'c',ifelse(

IS_1$sex=="Both"&IS_1$measure=="DALYs (Disability-Adjusted Life Years)",'d',ifelse(

IS_1$sex=="Male"&IS_1$measure=="Prevalence",'e',ifelse(

IS_1$sex=="Male"&IS_1$measure=="Incidence",'f',ifelse(

IS_1$sex=="Male"&IS_1$measure=="Deaths",'g',ifelse(

IS_1$sex=="Male"&IS_1$measure=="DALYs (Disability-Adjusted Life Years)",'h',ifelse(

IS_1$sex=="Female"&IS_1$measure=="Prevalence",'i',ifelse(

IS_1$sex=="Female"&IS_1$measure=="Incidence",'j',ifelse(

IS_1$sex=="Female"&IS_1$measure=="Deaths",'k','l')))))))))))

p <- ggplot(data=IS_1,aes(x=year,y=val)) +

geom_line(aes(color=location)) +

geom_point(aes(color=location)) +

scale_color_manual(values=pal_lancet(palette = c("lanonc"), alpha = 1)(6)[c(1,3,2,5,6,4)]) +

facet_wrap(~group,scales="free_y",

ncol = 4,

labeller = labeller(group=c(

a="Both-Prevalence",

b="Both-Incidence",

c="Both-Deaths",

d="Both-DALYs",

e="Male-Prevalence",

f="Male-Incidence",

g="Male-Deaths",

h="Male-DALYs",

i="Female-Prevalence",

j="Female-Incidence",

k="Female-Deaths",

l="Female-DALYs"

)))+

theme_bw() +

theme(

strip.text.x = element_text(size = 10,

color = "#ada072",

face = "bold"),

strip.background = element_rect(colour = "black",

fill = "#eeecdf"),

axis.title = element_blank(),

legend.title = element_blank(),

legend.position = "top") +

guides(

color = guide_legend(

nrow= 1,

byrow= T

)

)

p

IS_1 <- IS %>%

filter(age=="Age-standardized" &

metric=="Rate") %>%

filter(location=="Global" |

location=="High SDI" |

location=="High-middle SDI" |

location=="Middle SDI" |

location=="Low-middle SDI" |

location=="Low SDI")

IS_1$group <- ifelse(

IS_1$sex=="Both"&IS_1$measure=="Prevalence",'a',ifelse(

IS_1$sex=="Both"&IS_1$measure=="Incidence",'b',ifelse(

IS_1$sex=="Both"&IS_1$measure=="Deaths",'c',ifelse(

IS_1$sex=="Both"&IS_1$measure=="DALYs (Disability-Adjusted Life Years)",'d',ifelse(

IS_1$sex=="Male"&IS_1$measure=="Prevalence",'e',ifelse(

IS_1$sex=="Male"&IS_1$measure=="Incidence",'f',ifelse(

IS_1$sex=="Male"&IS_1$measure=="Deaths",'g',ifelse(

IS_1$sex=="Male"&IS_1$measure=="DALYs (Disability-Adjusted Life Years)",'h',ifelse(

IS_1$sex=="Female"&IS_1$measure=="Prevalence",'i',ifelse(

IS_1$sex=="Female"&IS_1$measure=="Incidence",'j',ifelse(

IS_1$sex=="Female"&IS_1$measure=="Deaths",'k','l')))))))))))

p <- ggplot(data=IS_1,aes(x=year,y=val)) +

geom_line(aes(color=location)) +

geom_point(aes(color=location)) +

scale_color_manual(values=pal_lancet(palette = c("lanonc"), alpha = 1)(6)[c(1,3,2,5,6,4)]) +

facet_wrap(~group,scales="free_y",

ncol = 4,

labeller = labeller(group=c(

a="Both-ASPR per 100,000",

b="Both-ASIR per 100,000",

c="Both-ASDR per 100,000",

d="Both-ASDALYR per 100,000",

e="Male-ASPR per 100,000",

f="Male-ASIR per 100,000",

g="Male-ASDR per 100,000",

h="Male-ASDALYR per 100,000",

i="Female-ASPR per 100,000",

j="Female-ASIR per 100,000",

k="Female-ASDR per 100,000",

l="Female-ASDALYR per 100,000"

)))+

theme_bw() +

theme(

strip.text.x = element_text(size = 10,

color = "#ada072",

face = "bold"),

strip.background = element_rect(colour = "black",

fill = "#eeecdf"),

axis.title = element_blank(),

legend.title = element_blank(),

legend.position = "top") +

guides(

color = guide_legend(

nrow= 1,

byrow= T

)

)

p

region<- vroom::vroom("/MM_region.csv")

IS <- region %>%

dplyr::select(measure_name,location_name,metric_name,cause_name,

sex_name,age_name,year,val,lower,upper) %>%

rename(measure=measure_name,

location=location_name,

metric=metric_name,

sex=sex_name, cause=cause_name

age=age_name)

IS_1 <- IS %>%

filter(age=="All ages" &

metric=="Number") %>%

filter(location=="Global" |

location=="High SDI" |

location=="High-middle SDI" |

location=="Middle SDI" |

location=="Low-middle SDI" |

location=="Low SDI")

IS_1$group <- ifelse(

IS_1$sex=="Both"&IS_1$measure=="Prevalence",'a',ifelse(

IS_1$sex=="Both"&IS_1$measure=="Incidence",'b',ifelse(

IS_1$sex=="Both"&IS_1$measure=="Deaths",'c',ifelse(

IS_1$sex=="Both"&IS_1$measure=="DALYs (Disability-Adjusted Life Years)",'d',ifelse(

IS_1$sex=="Male"&IS_1$measure=="Prevalence",'e',ifelse(

IS_1$sex=="Male"&IS_1$measure=="Incidence",'f',ifelse(

IS_1$sex=="Male"&IS_1$measure=="Deaths",'g',ifelse(

IS_1$sex=="Male"&IS_1$measure=="DALYs (Disability-Adjusted Life Years)",'h',ifelse(

IS_1$sex=="Female"&IS_1$measure=="Prevalence",'i',ifelse(

IS_1$sex=="Female"&IS_1$measure=="Incidence",'j',ifelse(

IS_1$sex=="Female"&IS_1$measure=="Deaths",'k','l')))))))))))

p <- ggplot(data=IS_1,aes(x=year,y=val)) +

geom_line(aes(color=location)) +

geom_point(aes(color=location)) +

scale_color_manual(values=pal_lancet(palette = c("lanonc"), alpha = 1)(6)[c(1,3,2,5,6,4)]) +

facet_wrap(~group,scales="free_y",

ncol = 4,

labeller = labeller(group=c(

a="Both-Prevalence",

b="Both-Incidence",

c="Both-Deaths",

d="Both-DALYs",

e="Male-Prevalence",

f="Male-Incidence",

g="Male-Deaths",

h="Male-DALYs",

i="Female-Prevalence",

j="Female-Incidence",

k="Female-Deaths",

l="Female-DALYs"

)))+

theme_bw() +

theme(

strip.text.x = element_text(size = 10,

color = "#ada072",

face = "bold"),

strip.background = element_rect(colour = "black",

fill = "#eeecdf"),

axis.title = element_blank(),

legend.title = element_blank(),

legend.position = "top") +

guides(

color = guide_legend(

nrow= 1,

byrow= T

)

)

p

IS_1 <- IS %>%

filter(age=="Age-standardized" &

metric=="Rate") %>%

filter(location=="Global" |

location=="High SDI" |

location=="High-middle SDI" |

location=="Middle SDI" |

location=="Low-middle SDI" |

location=="Low SDI")

IS_1$group <- ifelse(

IS_1$sex=="Both"&IS_1$measure=="Prevalence",'a',ifelse(

IS_1$sex=="Both"&IS_1$measure=="Incidence",'b',ifelse(

IS_1$sex=="Both"&IS_1$measure=="Deaths",'c',ifelse(

IS_1$sex=="Both"&IS_1$measure=="DALYs (Disability-Adjusted Life Years)",'d',ifelse(

IS_1$sex=="Male"&IS_1$measure=="Prevalence",'e',ifelse(

IS_1$sex=="Male"&IS_1$measure=="Incidence",'f',ifelse(

IS_1$sex=="Male"&IS_1$measure=="Deaths",'g',ifelse(

IS_1$sex=="Male"&IS_1$measure=="DALYs (Disability-Adjusted Life Years)",'h',ifelse(

IS_1$sex=="Female"&IS_1$measure=="Prevalence",'i',ifelse(

IS_1$sex=="Female"&IS_1$measure=="Incidence",'j',ifelse(

IS_1$sex=="Female"&IS_1$measure=="Deaths",'k','l')))))))))))

p <- ggplot(data=IS_1,aes(x=year,y=val)) +

geom_line(aes(color=location)) +

geom_point(aes(color=location)) +

scale_color_manual(values=pal_lancet(palette = c("lanonc"), alpha = 1)(6)[c(1,3,2,5,6,4)]) +

facet_wrap(~group,scales="free_y",

ncol = 4,

labeller = labeller(group=c(

a="Both-ASPR per 100,000",

b="Both-ASIR per 100,000",

c="Both-ASDR per 100,000",

d="Both-ASDALYR per 100,000",

e="Male-ASPR per 100,000",

f="Male-ASIR per 100,000",

g="Male-ASDR per 100,000",

h="Male-ASDALYR per 100,000",

i="Female-ASPR per 100,000",

j="Female-ASIR per 100,000",

k="Female-ASDR per 100,000",

l="Female-ASDALYR per 100,000"

)))+

theme_bw() +

theme(

strip.text.x = element_text(size = 10,

color = "#ada072",

face = "bold"),

strip.background = element_rect(colour = "black",

fill = "#eeecdf"),

axis.title = element_blank(),

legend.title = element_blank(),

legend.position = "top") +

guides(

color = guide_legend(

nrow= 1,

byrow= T

)

)

p

#Figure 3; Figure S6-S12

library(sf)

library(maps)

library(patchwork)

library(dplyr)

library(ggplot2)

library(RColorBrewer)

world <- map_data('world')

HL_country<- vroom::vroom("HL_country.csv")

IS <- HL_country %>%

dplyr::select(measure_name,location_name,metric_name,

sex_name,age_name,year,val,lower,upper) %>%

rename(measure=measure_name,

location=location_name,

metric=metric_name,

sex=sex_name,

age=age_name)

IS1 <- IS %>%

filter(measure == "Incidence") %>%

filter(year == 2021) %>%

filter(sex == "Both") %>%

filter(age == "Age-standardized") %>%

filter(metric == "Rate")

summary(IS1$val)

quantile(IS1$val,seq(0.1,1,0.1))

map <- IS1%>% mutate(val2 = cut(val, breaks = c(0,1,2,3,4,10000000),

labels = c("<1","1-2","2-3","3-4",">4"),

include.lowest = T,right = T))

map$location[map$location == 'United States of America'] = 'USA'

map$location[map$location == 'Russian Federation'] = 'Russia'

map$location[map$location == 'United Kingdom'] = 'UK'

map$location[map$location == 'Congo'] = 'Republic of Congo'

map$location[map$location == "Iran (Islamic Republic of)"] = 'Iran'

map$location[map$location == "Democratic People's Republic of Korea"] = 'North Korea'

map$location[map$location == "Taiwan (Province of China)"] = 'Taiwan'

map$location[map$location == "Republic of Korea"] = 'South Korea'

map$location[map$location == "United Republic of Tanzania"] = 'Tanzania'

map$location[map$location == "Bolivia (Plurinational State of)"] = 'Bolivia'

map$location[map$location == "Venezuela (Bolivarian Republic of)"] = 'Venezuela'

map$location[map$location == "Czechia"] = 'Czech Republic'

map$location[map$location == "Republic of Moldova"] = 'Moldova'

map$location[map$location == "Viet Nam"] = 'Vietnam'

map$location[map$location == "Lao People's Democratic Republic"] = 'Laos'

map$location[map$location == "Syrian Arab Republic"] = 'Syria'

map$location[map$location == "North Macedonia"] = 'Macedonia'

map$location[map$location == "Micronesia (Federated States of)"] = 'Micronesia'

map$location[map$location == "Macedonia"] = 'North Macedonia'

map$location[map$location == "Trinidad and Tobago"] = 'Trinidad'

a <- map[map$location == "Trinidad",]

a$location <- 'Tobago'

map <- rbind(map,a)

map$location[map$location == "Cabo Verde"] = 'Cape Verde'

map$location[map$location == "United States Virgin Islands"] = 'Virgin Islands'

map$location[map$location == "Antigua and Barbuda"] = 'Antigu'

a <- map[map$location == "Antigu",]

a$location <- 'Barbuda'

map <- rbind(map,a)

map$location[map$location == "Saint Kitts and Nevis"] = 'Saint Kitts'

a <- map[map$location == "Saint Kitts",]

a$location <- 'Nevis'

map <- rbind(map,a)

map$location[map$location == "Côte d'Ivoire"] = 'Ivory Coast'

map$location[map$location == "Saint Vincent and the Grenadines"] = 'Saint Vincent'

a <- map[map$location == "Saint Vincent",]

a$location <- 'Grenadines'

map <- rbind(map,a)

map$location[map$location == "Eswatini"] = 'Swaziland'

map$location[map$location == "Brunei Darussalam"] = 'Brunei'

map <- full_join(world,map,by = c('region'='location')) %>%

filter(val != "NA")

mycolor2<-brewer.pal(5, "Blues")

fig <- map %>%

ggplot()+

geom_polygon(aes(x = long, y = lat,group = group,fill=val2),colour="white",

size=0.2) +

theme_void()+

scale_fill_manual(values=mycolor2) +

theme(legend.position = c(0.1,0.2),

legend.title = element_blank(),

legend.text = element_text(color="black",

size = 12,

),

plot.title = element_blank(),

panel.grid=element_blank(),

axis.title.x = element_blank(),

axis.text.x = element_blank(),

axis.ticks.x = element_blank(),

axis.title.y = element_blank(),

axis.text.y = element_blank(),

axis.ticks.y = element_blank(),

)

fig

IS1 <- IS %>%

filter(measure == "Prevalence") %>%

filter(year == 2021) %>%

filter(sex == "Both") %>%

filter(age == "Age-standardized") %>%

filter(metric == "Rate")

summary(IS1$val)

quantile(IS1$val,seq(0.1,1,0.1))

map <- IS1%>% mutate(val2 = cut(val, breaks = c(0,1,2,3,4,10000000),

labels = c("<1","1-2","2-3","3-4",">4"),

include.lowest = T,right = T))

map$location[map$location == 'United States of America'] = 'USA'

map$location[map$location == 'Russian Federation'] = 'Russia'

map$location[map$location == 'United Kingdom'] = 'UK'

map$location[map$location == 'Congo'] = 'Republic of Congo'

map$location[map$location == "Iran (Islamic Republic of)"] = 'Iran'

map$location[map$location == "Democratic People's Republic of Korea"] = 'North Korea'

map$location[map$location == "Taiwan (Province of China)"] = 'Taiwan'

map$location[map$location == "Republic of Korea"] = 'South Korea'

map$location[map$location == "United Republic of Tanzania"] = 'Tanzania'

map$location[map$location == "Bolivia (Plurinational State of)"] = 'Bolivia'

map$location[map$location == "Venezuela (Bolivarian Republic of)"] = 'Venezuela'

map$location[map$location == "Czechia"] = 'Czech Republic'

map$location[map$location == "Republic of Moldova"] = 'Moldova'

map$location[map$location == "Viet Nam"] = 'Vietnam'

map$location[map$location == "Lao People's Democratic Republic"] = 'Laos'

map$location[map$location == "Syrian Arab Republic"] = 'Syria'

map$location[map$location == "North Macedonia"] = 'Macedonia'

map$location[map$location == "Micronesia (Federated States of)"] = 'Micronesia'

map$location[map$location == "Macedonia"] = 'North Macedonia'

map$location[map$location == "Trinidad and Tobago"] = 'Trinidad'

a <- map[map$location == "Trinidad",]

a$location <- 'Tobago'

map <- rbind(map,a)

map$location[map$location == "Cabo Verde"] = 'Cape Verde'

map$location[map$location == "United States Virgin Islands"] = 'Virgin Islands'

map$location[map$location == "Antigua and Barbuda"] = 'Antigu'

a <- map[map$location == "Antigu",]

a$location <- 'Barbuda'

map <- rbind(map,a)

map$location[map$location == "Saint Kitts and Nevis"] = 'Saint Kitts'

a <- map[map$location == "Saint Kitts",]

a$location <- 'Nevis'

map <- rbind(map,a)

map$location[map$location == "Côte d'Ivoire"] = 'Ivory Coast'

map$location[map$location == "Saint Vincent and the Grenadines"] = 'Saint Vincent'

a <- map[map$location == "Saint Vincent",]

a$location <- 'Grenadines'

map <- rbind(map,a)

map$location[map$location == "Eswatini"] = 'Swaziland'

map$location[map$location == "Brunei Darussalam"] = 'Brunei'

map <- full_join(world,map,by = c('region'='location')) %>%

filter(val != "NA")

mycolor2<-brewer.pal(5, "Blues")

fig <- map %>%

ggplot()+

geom_polygon(aes(x = long, y = lat,group = group,fill=val2),colour="white",

size=0.2) +

theme_void()+

scale_fill_manual(values=mycolor2) +

theme(legend.position = c(0.1,0.2),

legend.title = element_blank(),

legend.text = element_text(color="black",

size = 12,

),

plot.title = element_blank(),

panel.grid=element_blank(),

axis.title.x = element_blank(),

axis.text.x = element_blank(),

axis.ticks.x = element_blank(),

axis.title.y = element_blank(),

axis.text.y = element_blank(),

axis.ticks.y = element_blank(),

)

fig

IS1 <- IS %>%

filter(measure == "Deaths") %>%

filter(year == 2021) %>%

filter(sex == "Both") %>%

filter(age == "Age-standardized") %>%

filter(metric == "Rate")

summary(IS1$val)

quantile(IS1$val,seq(0.1,1,0.1))

map <- IS1%>% mutate(val2 = cut(val, breaks = c(0,1,2,3,4,10000000),

labels = c("<1","1-2","2-3","3-4",">4"),

include.lowest = T,right = T))

map$location[map$location == 'United States of America'] = 'USA'

map$location[map$location == 'Russian Federation'] = 'Russia'

map$location[map$location == 'United Kingdom'] = 'UK'

map$location[map$location == 'Congo'] = 'Republic of Congo'

map$location[map$location == "Iran (Islamic Republic of)"] = 'Iran'

map$location[map$location == "Democratic People's Republic of Korea"] = 'North Korea'

map$location[map$location == "Taiwan (Province of China)"] = 'Taiwan'

map$location[map$location == "Republic of Korea"] = 'South Korea'

map$location[map$location == "United Republic of Tanzania"] = 'Tanzania'

map$location[map$location == "Bolivia (Plurinational State of)"] = 'Bolivia'

map$location[map$location == "Venezuela (Bolivarian Republic of)"] = 'Venezuela'

map$location[map$location == "Czechia"] = 'Czech Republic'

map$location[map$location == "Republic of Moldova"] = 'Moldova'

map$location[map$location == "Viet Nam"] = 'Vietnam'

map$location[map$location == "Lao People's Democratic Republic"] = 'Laos'

map$location[map$location == "Syrian Arab Republic"] = 'Syria'

map$location[map$location == "North Macedonia"] = 'Macedonia'

map$location[map$location == "Micronesia (Federated States of)"] = 'Micronesia'

map$location[map$location == "Macedonia"] = 'North Macedonia'

map$location[map$location == "Trinidad and Tobago"] = 'Trinidad'

a <- map[map$location == "Trinidad",]

a$location <- 'Tobago'

map <- rbind(map,a)

map$location[map$location == "Cabo Verde"] = 'Cape Verde'

map$location[map$location == "United States Virgin Islands"] = 'Virgin Islands'

map$location[map$location == "Antigua and Barbuda"] = 'Antigu'

a <- map[map$location == "Antigu",]

a$location <- 'Barbuda'

map <- rbind(map,a)

map$location[map$location == "Saint Kitts and Nevis"] = 'Saint Kitts'

a <- map[map$location == "Saint Kitts",]

a$location <- 'Nevis'

map <- rbind(map,a)

map$location[map$location == "Côte d'Ivoire"] = 'Ivory Coast'

map$location[map$location == "Saint Vincent and the Grenadines"] = 'Saint Vincent'

a <- map[map$location == "Saint Vincent",]

a$location <- 'Grenadines'

map <- rbind(map,a)

map$location[map$location == "Eswatini"] = 'Swaziland'

map$location[map$location == "Brunei Darussalam"] = 'Brunei'

map <- full_join(world,map,by = c('region'='location')) %>%

filter(val != "NA")

mycolor2<-brewer.pal(5, "Blues")

fig <- map %>%

ggplot()+

geom_polygon(aes(x = long, y = lat,group = group,fill=val2),colour="white",

size=0.2) +

theme_void()+

scale_fill_manual(values=mycolor2) +

theme(legend.position = c(0.1,0.2),

legend.title = element_blank(),

legend.text = element_text(color="black",

size = 12,

),

plot.title = element_blank(),

panel.grid=element_blank(),

axis.title.x = element_blank(),

axis.text.x = element_blank(),

axis.ticks.x = element_blank(),

axis.title.y = element_blank(),

axis.text.y = element_blank(),

axis.ticks.y = element_blank(),

)

fig

IS1 <- IS %>%

filter(measure == "DALYs") %>%

filter(year == 2021) %>%

filter(sex == "Both") %>%

filter(age == "Age-standardized") %>%

filter(metric == "Rate")

summary(IS1$val)

quantile(IS1$val,seq(0.1,1,0.1))

map <- IS1%>% mutate(val2 = cut(val, breaks = c(0,1,2,3,4,10000000),

labels = c("<1","1-2","2-3","3-4",">4"),

include.lowest = T,right = T))

map$location[map$location == 'United States of America'] = 'USA'

map$location[map$location == 'Russian Federation'] = 'Russia'

map$location[map$location == 'United Kingdom'] = 'UK'

map$location[map$location == 'Congo'] = 'Republic of Congo'

map$location[map$location == "Iran (Islamic Republic of)"] = 'Iran'

map$location[map$location == "Democratic People's Republic of Korea"] = 'North Korea'

map$location[map$location == "Taiwan (Province of China)"] = 'Taiwan'

map$location[map$location == "Republic of Korea"] = 'South Korea'

map$location[map$location == "United Republic of Tanzania"] = 'Tanzania'

map$location[map$location == "Bolivia (Plurinational State of)"] = 'Bolivia'

map$location[map$location == "Venezuela (Bolivarian Republic of)"] = 'Venezuela'

map$location[map$location == "Czechia"] = 'Czech Republic'

map$location[map$location == "Republic of Moldova"] = 'Moldova'

map$location[map$location == "Viet Nam"] = 'Vietnam'

map$location[map$location == "Lao People's Democratic Republic"] = 'Laos'

map$location[map$location == "Syrian Arab Republic"] = 'Syria'

map$location[map$location == "North Macedonia"] = 'Macedonia'

map$location[map$location == "Micronesia (Federated States of)"] = 'Micronesia'

map$location[map$location == "Macedonia"] = 'North Macedonia'

map$location[map$location == "Trinidad and Tobago"] = 'Trinidad'

a <- map[map$location == "Trinidad",]

a$location <- 'Tobago'

map <- rbind(map,a)

map$location[map$location == "Cabo Verde"] = 'Cape Verde'

map$location[map$location == "United States Virgin Islands"] = 'Virgin Islands'

map$location[map$location == "Antigua and Barbuda"] = 'Antigu'

a <- map[map$location == "Antigu",]

a$location <- 'Barbuda'

map <- rbind(map,a)

map$location[map$location == "Saint Kitts and Nevis"] = 'Saint Kitts'

a <- map[map$location == "Saint Kitts",]

a$location <- 'Nevis'

map <- rbind(map,a)

map$location[map$location == "Côte d'Ivoire"] = 'Ivory Coast'

map$location[map$location == "Saint Vincent and the Grenadines"] = 'Saint Vincent'

a <- map[map$location == "Saint Vincent",]

a$location <- 'Grenadines'

map <- rbind(map,a)

map$location[map$location == "Eswatini"] = 'Swaziland'

map$location[map$location == "Brunei Darussalam"] = 'Brunei'

map <- full_join(world,map,by = c('region'='location')) %>%

filter(val != "NA")

mycolor2<-brewer.pal(5, "Blues")

fig <- map %>%

ggplot()+

geom_polygon(aes(x = long, y = lat,group = group,fill=val2),colour="white",

size=0.2) +

theme_void()+

scale_fill_manual(values=mycolor2) +

theme(legend.position = c(0.1,0.2),

legend.title = element_blank(),

legend.text = element_text(color="black",

size = 12,

),

plot.title = element_blank(),

panel.grid=element_blank(),

axis.title.x = element_blank(),

axis.text.x = element_blank(),

axis.ticks.x = element_blank(),

axis.title.y = element_blank(),

axis.text.y = element_blank(),

axis.ticks.y = element_blank(),

)

fig

IS1 <- IS %>%

filter(measure == "Incidence") %>%

filter(year == 2021) %>%

filter(sex == "Both") %>%

filter(age == "All ages") %>%

filter(metric == "Number")

summary(IS1$val)

quantile(IS1$val,seq(0.1,1,0.1))

map <- IS1%>% mutate(val2 = cut(val, breaks = c(0,1,2,3,4,10000000),

labels = c("<1","1-2","2-3","3-4",">4"),

include.lowest = T,right = T))

map$location[map$location == 'United States of America'] = 'USA'

map$location[map$location == 'Russian Federation'] = 'Russia'

map$location[map$location == 'United Kingdom'] = 'UK'

map$location[map$location == 'Congo'] = 'Republic of Congo'

map$location[map$location == "Iran (Islamic Republic of)"] = 'Iran'

map$location[map$location == "Democratic People's Republic of Korea"] = 'North Korea'

map$location[map$location == "Taiwan (Province of China)"] = 'Taiwan'

map$location[map$location == "Republic of Korea"] = 'South Korea'

map$location[map$location == "United Republic of Tanzania"] = 'Tanzania'

map$location[map$location == "Bolivia (Plurinational State of)"] = 'Bolivia'

map$location[map$location == "Venezuela (Bolivarian Republic of)"] = 'Venezuela'

map$location[map$location == "Czechia"] = 'Czech Republic'

map$location[map$location == "Republic of Moldova"] = 'Moldova'

map$location[map$location == "Viet Nam"] = 'Vietnam'

map$location[map$location == "Lao People's Democratic Republic"] = 'Laos'

map$location[map$location == "Syrian Arab Republic"] = 'Syria'

map$location[map$location == "North Macedonia"] = 'Macedonia'

map$location[map$location == "Micronesia (Federated States of)"] = 'Micronesia'

map$location[map$location == "Macedonia"] = 'North Macedonia'

map$location[map$location == "Trinidad and Tobago"] = 'Trinidad'

a <- map[map$location == "Trinidad",]

a$location <- 'Tobago'

map <- rbind(map,a)

map$location[map$location == "Cabo Verde"] = 'Cape Verde'

map$location[map$location == "United States Virgin Islands"] = 'Virgin Islands'

map$location[map$location == "Antigua and Barbuda"] = 'Antigu'

a <- map[map$location == "Antigu",]

a$location <- 'Barbuda'

map <- rbind(map,a)

map$location[map$location == "Saint Kitts and Nevis"] = 'Saint Kitts'

a <- map[map$location == "Saint Kitts",]

a$location <- 'Nevis'

map <- rbind(map,a)

map$location[map$location == "Côte d'Ivoire"] = 'Ivory Coast'

map$location[map$location == "Saint Vincent and the Grenadines"] = 'Saint Vincent'

a <- map[map$location == "Saint Vincent",]

a$location <- 'Grenadines'

map <- rbind(map,a)

map$location[map$location == "Eswatini"] = 'Swaziland'

map$location[map$location == "Brunei Darussalam"] = 'Brunei'

map <- full_join(world,map,by = c('region'='location')) %>%

filter(val != "NA")

mycolor2<-brewer.pal(5, "Blues")

fig <- map %>%

ggplot()+

geom_polygon(aes(x = long, y = lat,group = group,fill=val2),colour="white",

size=0.2) +

theme_void()+

scale_fill_manual(values=mycolor2) +

theme(legend.position = c(0.1,0.2),

legend.title = element_blank(),

legend.text = element_text(color="black",

size = 12,

),

plot.title = element_blank(),

panel.grid=element_blank(),

axis.title.x = element_blank(),

axis.text.x = element_blank(),

axis.ticks.x = element_blank(),

axis.title.y = element_blank(),

axis.text.y = element_blank(),

axis.ticks.y = element_blank(),

)

fig

IS1 <- IS %>%

filter(measure == "Prevalence") %>%

filter(year == 2021) %>%

filter(sex == "Both") %>%

filter(age == "All ages") %>%

filter(metric == "Number")

summary(IS1$val)

quantile(IS1$val,seq(0.1,1,0.1))

map <- IS1%>% mutate(val2 = cut(val, breaks = c(0,1,2,3,4,10000000),

labels = c("<1","1-2","2-3","3-4",">4"),

include.lowest = T,right = T))

map$location[map$location == 'United States of America'] = 'USA'

map$location[map$location == 'Russian Federation'] = 'Russia'

map$location[map$location == 'United Kingdom'] = 'UK'

map$location[map$location == 'Congo'] = 'Republic of Congo'

map$location[map$location == "Iran (Islamic Republic of)"] = 'Iran'

map$location[map$location == "Democratic People's Republic of Korea"] = 'North Korea'

map$location[map$location == "Taiwan (Province of China)"] = 'Taiwan'

map$location[map$location == "Republic of Korea"] = 'South Korea'

map$location[map$location == "United Republic of Tanzania"] = 'Tanzania'

map$location[map$location == "Bolivia (Plurinational State of)"] = 'Bolivia'

map$location[map$location == "Venezuela (Bolivarian Republic of)"] = 'Venezuela'

map$location[map$location == "Czechia"] = 'Czech Republic'

map$location[map$location == "Republic of Moldova"] = 'Moldova'

map$location[map$location == "Viet Nam"] = 'Vietnam'

map$location[map$location == "Lao People's Democratic Republic"] = 'Laos'

map$location[map$location == "Syrian Arab Republic"] = 'Syria'

map$location[map$location == "North Macedonia"] = 'Macedonia'

map$location[map$location == "Micronesia (Federated States of)"] = 'Micronesia'

map$location[map$location == "Macedonia"] = 'North Macedonia'

map$location[map$location == "Trinidad and Tobago"] = 'Trinidad'

a <- map[map$location == "Trinidad",]

a$location <- 'Tobago'

map <- rbind(map,a)

map$location[map$location == "Cabo Verde"] = 'Cape Verde'

map$location[map$location == "United States Virgin Islands"] = 'Virgin Islands'

map$location[map$location == "Antigua and Barbuda"] = 'Antigu'

a <- map[map$location == "Antigu",]

a$location <- 'Barbuda'

map <- rbind(map,a)

map$location[map$location == "Saint Kitts and Nevis"] = 'Saint Kitts'

a <- map[map$location == "Saint Kitts",]

a$location <- 'Nevis'

map <- rbind(map,a)

map$location[map$location == "Côte d'Ivoire"] = 'Ivory Coast'

map$location[map$location == "Saint Vincent and the Grenadines"] = 'Saint Vincent'

a <- map[map$location == "Saint Vincent",]

a$location <- 'Grenadines'

map <- rbind(map,a)

map$location[map$location == "Eswatini"] = 'Swaziland'

map$location[map$location == "Brunei Darussalam"] = 'Brunei'

map <- full_join(world,map,by = c('region'='location')) %>%

filter(val != "NA")

mycolor2<-brewer.pal(5, "Blues")

fig <- map %>%

ggplot()+

geom_polygon(aes(x = long, y = lat,group = group,fill=val2),colour="white",

size=0.2) +

theme_void()+

scale_fill_manual(values=mycolor2) +

theme(legend.position = c(0.1,0.2),

legend.title = element_blank(),

legend.text = element_text(color="black",

size = 12,

),

plot.title = element_blank(),

panel.grid=element_blank(),

axis.title.x = element_blank(),

axis.text.x = element_blank(),

axis.ticks.x = element_blank(),

axis.title.y = element_blank(),

axis.text.y = element_blank(),

axis.ticks.y = element_blank(),

)

fig

IS1 <- IS %>%

filter(measure == "Deaths") %>%

filter(year == 2021) %>%

filter(sex == "Both") %>%

filter(age == "All ages") %>%

filter(metric == "Number")

summary(IS1$val)

quantile(IS1$val,seq(0.1,1,0.1))

map <- IS1%>% mutate(val2 = cut(val, breaks = c(0,1,2,3,4,10000000),

labels = c("<1","1-2","2-3","3-4",">4"),

include.lowest = T,right = T))

map$location[map$location == 'United States of America'] = 'USA'

map$location[map$location == 'Russian Federation'] = 'Russia'

map$location[map$location == 'United Kingdom'] = 'UK'

map$location[map$location == 'Congo'] = 'Republic of Congo'

map$location[map$location == "Iran (Islamic Republic of)"] = 'Iran'

map$location[map$location == "Democratic People's Republic of Korea"] = 'North Korea'

map$location[map$location == "Taiwan (Province of China)"] = 'Taiwan'

map$location[map$location == "Republic of Korea"] = 'South Korea'

map$location[map$location == "United Republic of Tanzania"] = 'Tanzania'

map$location[map$location == "Bolivia (Plurinational State of)"] = 'Bolivia'

map$location[map$location == "Venezuela (Bolivarian Republic of)"] = 'Venezuela'

map$location[map$location == "Czechia"] = 'Czech Republic'

map$location[map$location == "Republic of Moldova"] = 'Moldova'

map$location[map$location == "Viet Nam"] = 'Vietnam'

map$location[map$location == "Lao People's Democratic Republic"] = 'Laos'

map$location[map$location == "Syrian Arab Republic"] = 'Syria'

map$location[map$location == "North Macedonia"] = 'Macedonia'

map$location[map$location == "Micronesia (Federated States of)"] = 'Micronesia'

map$location[map$location == "Macedonia"] = 'North Macedonia'

map$location[map$location == "Trinidad and Tobago"] = 'Trinidad'

a <- map[map$location == "Trinidad",]

a$location <- 'Tobago'

map <- rbind(map,a)

map$location[map$location == "Cabo Verde"] = 'Cape Verde'

map$location[map$location == "United States Virgin Islands"] = 'Virgin Islands'

map$location[map$location == "Antigua and Barbuda"] = 'Antigu'

a <- map[map$location == "Antigu",]

a$location <- 'Barbuda'

map <- rbind(map,a)

map$location[map$location == "Saint Kitts and Nevis"] = 'Saint Kitts'

a <- map[map$location == "Saint Kitts",]

a$location <- 'Nevis'

map <- rbind(map,a)

map$location[map$location == "Côte d'Ivoire"] = 'Ivory Coast'

map$location[map$location == "Saint Vincent and the Grenadines"] = 'Saint Vincent'

a <- map[map$location == "Saint Vincent",]

a$location <- 'Grenadines'

map <- rbind(map,a)

map$location[map$location == "Eswatini"] = 'Swaziland'

map$location[map$location == "Brunei Darussalam"] = 'Brunei'

map <- full_join(world,map,by = c('region'='location')) %>%

filter(val != "NA")

mycolor2<-brewer.pal(5, "Blues")

fig <- map %>%

ggplot()+

geom_polygon(aes(x = long, y = lat,group = group,fill=val2),colour="white",

size=0.2) +

theme_void()+

scale_fill_manual(values=mycolor2) +

theme(legend.position = c(0.1,0.2),

legend.title = element_blank(),

legend.text = element_text(color="black",

size = 12,

),

plot.title = element_blank(),

panel.grid=element_blank(),

axis.title.x = element_blank(),

axis.text.x = element_blank(),

axis.ticks.x = element_blank(),

axis.title.y = element_blank(),

axis.text.y = element_blank(),

axis.ticks.y = element_blank(),

)

fig

IS1 <- IS %>%

filter(measure == "DALYs") %>%

filter(year == 2021) %>%

filter(sex == "Both") %>%

filter(age == "All ages") %>%

filter(metric == "Number")

summary(IS1$val)

quantile(IS1$val,seq(0.1,1,0.1))

map <- IS1%>% mutate(val2 = cut(val, breaks = c(0,1,2,3,4,10000000),

labels = c("<1","1-2","2-3","3-4",">4"),

include.lowest = T,right = T))

map$location[map$location == 'United States of America'] = 'USA'

map$location[map$location == 'Russian Federation'] = 'Russia'

map$location[map$location == 'United Kingdom'] = 'UK'

map$location[map$location == 'Congo'] = 'Republic of Congo'

map$location[map$location == "Iran (Islamic Republic of)"] = 'Iran'

map$location[map$location == "Democratic People's Republic of Korea"] = 'North Korea'

map$location[map$location == "Taiwan (Province of China)"] = 'Taiwan'

map$location[map$location == "Republic of Korea"] = 'South Korea'

map$location[map$location == "United Republic of Tanzania"] = 'Tanzania'

map$location[map$location == "Bolivia (Plurinational State of)"] = 'Bolivia'

map$location[map$location == "Venezuela (Bolivarian Republic of)"] = 'Venezuela'

map$location[map$location == "Czechia"] = 'Czech Republic'

map$location[map$location == "Republic of Moldova"] = 'Moldova'

map$location[map$location == "Viet Nam"] = 'Vietnam'

map$location[map$location == "Lao People's Democratic Republic"] = 'Laos'

map$location[map$location == "Syrian Arab Republic"] = 'Syria'

map$location[map$location == "North Macedonia"] = 'Macedonia'

map$location[map$location == "Micronesia (Federated States of)"] = 'Micronesia'

map$location[map$location == "Macedonia"] = 'North Macedonia'

map$location[map$location == "Trinidad and Tobago"] = 'Trinidad'

a <- map[map$location == "Trinidad",]

a$location <- 'Tobago'

map <- rbind(map,a)

map$location[map$location == "Cabo Verde"] = 'Cape Verde'

map$location[map$location == "United States Virgin Islands"] = 'Virgin Islands'

map$location[map$location == "Antigua and Barbuda"] = 'Antigu'

a <- map[map$location == "Antigu",]

a$location <- 'Barbuda'

map <- rbind(map,a)

map$location[map$location == "Saint Kitts and Nevis"] = 'Saint Kitts'

a <- map[map$location == "Saint Kitts",]

a$location <- 'Nevis'

map <- rbind(map,a)

map$location[map$location == "Côte d'Ivoire"] = 'Ivory Coast'

map$location[map$location == "Saint Vincent and the Grenadines"] = 'Saint Vincent'

a <- map[map$location == "Saint Vincent",]

a$location <- 'Grenadines'

map <- rbind(map,a)

map$location[map$location == "Eswatini"] = 'Swaziland'

map$location[map$location == "Brunei Darussalam"] = 'Brunei'

map <- full_join(world,map,by = c('region'='location')) %>%

filter(val != "NA")

mycolor2<-brewer.pal(5, "Blues")

fig <- map %>%

ggplot()+

geom_polygon(aes(x = long, y = lat,group = group,fill=val2),colour="white",

size=0.2) +

theme_void()+

scale_fill_manual(values=mycolor2) +

theme(legend.position = c(0.1,0.2),

legend.title = element_blank(),

legend.text = element_text(color="black",

size = 12,

),

plot.title = element_blank(),

panel.grid=element_blank(),

axis.title.x = element_blank(),

axis.text.x = element_blank(),

axis.ticks.x = element_blank(),

axis.title.y = element_blank(),

axis.text.y = element_blank(),

axis.ticks.y = element_blank(),

)

fig

world <- map_data('world')

NHL_country<- vroom::vroom("NHL_country.csv")

IS <- NHL_country %>%

dplyr::select(measure_name,location_name,metric_name,

sex_name,age_name,year,val,lower,upper) %>%

rename(measure=measure_name,

location=location_name,

metric=metric_name,

sex=sex_name,

age=age_name)

IS1 <- IS %>%

filter(measure == "Incidence") %>%

filter(year == 2021) %>%

filter(sex == "Both") %>%

filter(age == "Age-standardized") %>%

filter(metric == "Rate")

summary(IS1$val)

quantile(IS1$val,seq(0.1,1,0.1))

map <- IS1%>% mutate(val2 = cut(val, breaks = c(0,1,2,3,4,10000000),

labels = c("<1","1-2","2-3","3-4",">4"),

include.lowest = T,right = T))

map$location[map$location == 'United States of America'] = 'USA'

map$location[map$location == 'Russian Federation'] = 'Russia'

map$location[map$location == 'United Kingdom'] = 'UK'

map$location[map$location == 'Congo'] = 'Republic of Congo'

map$location[map$location == "Iran (Islamic Republic of)"] = 'Iran'

map$location[map$location == "Democratic People's Republic of Korea"] = 'North Korea'

map$location[map$location == "Taiwan (Province of China)"] = 'Taiwan'

map$location[map$location == "Republic of Korea"] = 'South Korea'

map$location[map$location == "United Republic of Tanzania"] = 'Tanzania'

map$location[map$location == "Bolivia (Plurinational State of)"] = 'Bolivia'

map$location[map$location == "Venezuela (Bolivarian Republic of)"] = 'Venezuela'

map$location[map$location == "Czechia"] = 'Czech Republic'

map$location[map$location == "Republic of Moldova"] = 'Moldova'

map$location[map$location == "Viet Nam"] = 'Vietnam'

map$location[map$location == "Lao People's Democratic Republic"] = 'Laos'

map$location[map$location == "Syrian Arab Republic"] = 'Syria'

map$location[map$location == "North Macedonia"] = 'Macedonia'

map$location[map$location == "Micronesia (Federated States of)"] = 'Micronesia'

map$location[map$location == "Macedonia"] = 'North Macedonia'

map$location[map$location == "Trinidad and Tobago"] = 'Trinidad'

a <- map[map$location == "Trinidad",]

a$location <- 'Tobago'

map <- rbind(map,a)

map$location[map$location == "Cabo Verde"] = 'Cape Verde'

map$location[map$location == "United States Virgin Islands"] = 'Virgin Islands'

map$location[map$location == "Antigua and Barbuda"] = 'Antigu'

a <- map[map$location == "Antigu",]

a$location <- 'Barbuda'

map <- rbind(map,a)

map$location[map$location == "Saint Kitts and Nevis"] = 'Saint Kitts'

a <- map[map$location == "Saint Kitts",]

a$location <- 'Nevis'

map <- rbind(map,a)

map$location[map$location == "Côte d'Ivoire"] = 'Ivory Coast'

map$location[map$location == "Saint Vincent and the Grenadines"] = 'Saint Vincent'

a <- map[map$location == "Saint Vincent",]

a$location <- 'Grenadines'

map <- rbind(map,a)

map$location[map$location == "Eswatini"] = 'Swaziland'

map$location[map$location == "Brunei Darussalam"] = 'Brunei'

map <- full_join(world,map,by = c('region'='location')) %>%

filter(val != "NA")

mycolor2<-brewer.pal(5, "Blues")

fig <- map %>%

ggplot()+

geom_polygon(aes(x = long, y = lat,group = group,fill=val2),colour="white",

size=0.2) +

theme_void()+

scale_fill_manual(values=mycolor2) +

theme(legend.position = c(0.1,0.2),

legend.title = element_blank(),

legend.text = element_text(color="black",

size = 12,

),

plot.title = element_blank(),

panel.grid=element_blank(),

axis.title.x = element_blank(),

axis.text.x = element_blank(),

axis.ticks.x = element_blank(),

axis.title.y = element_blank(),

axis.text.y = element_blank(),

axis.ticks.y = element_blank(),

)

fig

IS1 <- IS %>%

filter(measure == "Prevalence") %>%

filter(year == 2021) %>%

filter(sex == "Both") %>%

filter(age == "Age-standardized") %>%

filter(metric == "Rate")

summary(IS1$val)

quantile(IS1$val,seq(0.1,1,0.1))

map <- IS1%>% mutate(val2 = cut(val, breaks = c(0,1,2,3,4,10000000),

labels = c("<1","1-2","2-3","3-4",">4"),

include.lowest = T,right = T))

map$location[map$location == 'United States of America'] = 'USA'

map$location[map$location == 'Russian Federation'] = 'Russia'

map$location[map$location == 'United Kingdom'] = 'UK'

map$location[map$location == 'Congo'] = 'Republic of Congo'

map$location[map$location == "Iran (Islamic Republic of)"] = 'Iran'

map$location[map$location == "Democratic People's Republic of Korea"] = 'North Korea'

map$location[map$location == "Taiwan (Province of China)"] = 'Taiwan'

map$location[map$location == "Republic of Korea"] = 'South Korea'

map$location[map$location == "United Republic of Tanzania"] = 'Tanzania'

map$location[map$location == "Bolivia (Plurinational State of)"] = 'Bolivia'

map$location[map$location == "Venezuela (Bolivarian Republic of)"] = 'Venezuela'

map$location[map$location == "Czechia"] = 'Czech Republic'

map$location[map$location == "Republic of Moldova"] = 'Moldova'

map$location[map$location == "Viet Nam"] = 'Vietnam'

map$location[map$location == "Lao People's Democratic Republic"] = 'Laos'

map$location[map$location == "Syrian Arab Republic"] = 'Syria'

map$location[map$location == "North Macedonia"] = 'Macedonia'

map$location[map$location == "Micronesia (Federated States of)"] = 'Micronesia'

map$location[map$location == "Macedonia"] = 'North Macedonia'

map$location[map$location == "Trinidad and Tobago"] = 'Trinidad'

a <- map[map$location == "Trinidad",]

a$location <- 'Tobago'

map <- rbind(map,a)

map$location[map$location == "Cabo Verde"] = 'Cape Verde'

map$location[map$location == "United States Virgin Islands"] = 'Virgin Islands'

map$location[map$location == "Antigua and Barbuda"] = 'Antigu'

a <- map[map$location == "Antigu",]

a$location <- 'Barbuda'

map <- rbind(map,a)

map$location[map$location == "Saint Kitts and Nevis"] = 'Saint Kitts'

a <- map[map$location == "Saint Kitts",]

a$location <- 'Nevis'

map <- rbind(map,a)

map$location[map$location == "Côte d'Ivoire"] = 'Ivory Coast'

map$location[map$location == "Saint Vincent and the Grenadines"] = 'Saint Vincent'

a <- map[map$location == "Saint Vincent",]

a$location <- 'Grenadines'

map <- rbind(map,a)

map$location[map$location == "Eswatini"] = 'Swaziland'

map$location[map$location == "Brunei Darussalam"] = 'Brunei'

map <- full_join(world,map,by = c('region'='location')) %>%

filter(val != "NA")

mycolor2<-brewer.pal(5, "Blues")

fig <- map %>%

ggplot()+

geom_polygon(aes(x = long, y = lat,group = group,fill=val2),colour="white",

size=0.2) +

theme_void()+

scale_fill_manual(values=mycolor2) +

theme(legend.position = c(0.1,0.2),

legend.title = element_blank(),

legend.text = element_text(color="black",

size = 12,

),

plot.title = element_blank(),

panel.grid=element_blank(),

axis.title.x = element_blank(),

axis.text.x = element_blank(),

axis.ticks.x = element_blank(),

axis.title.y = element_blank(),

axis.text.y = element_blank(),

axis.ticks.y = element_blank(),

)

fig

IS1 <- IS %>%

filter(measure == "Deaths") %>%

filter(year == 2021) %>%

filter(sex == "Both") %>%

filter(age == "Age-standardized") %>%

filter(metric == "Rate")

summary(IS1$val)

quantile(IS1$val,seq(0.1,1,0.1))

map <- IS1%>% mutate(val2 = cut(val, breaks = c(0,1,2,3,4,10000000),

labels = c("<1","1-2","2-3","3-4",">4"),

include.lowest = T,right = T))

map$location[map$location == 'United States of America'] = 'USA'

map$location[map$location == 'Russian Federation'] = 'Russia'

map$location[map$location == 'United Kingdom'] = 'UK'

map$location[map$location == 'Congo'] = 'Republic of Congo'

map$location[map$location == "Iran (Islamic Republic of)"] = 'Iran'

map$location[map$location == "Democratic People's Republic of Korea"] = 'North Korea'

map$location[map$location == "Taiwan (Province of China)"] = 'Taiwan'

map$location[map$location == "Republic of Korea"] = 'South Korea'

map$location[map$location == "United Republic of Tanzania"] = 'Tanzania'

map$location[map$location == "Bolivia (Plurinational State of)"] = 'Bolivia'

map$location[map$location == "Venezuela (Bolivarian Republic of)"] = 'Venezuela'

map$location[map$location == "Czechia"] = 'Czech Republic'

map$location[map$location == "Republic of Moldova"] = 'Moldova'

map$location[map$location == "Viet Nam"] = 'Vietnam'

map$location[map$location == "Lao People's Democratic Republic"] = 'Laos'

map$location[map$location == "Syrian Arab Republic"] = 'Syria'

map$location[map$location == "North Macedonia"] = 'Macedonia'

map$location[map$location == "Micronesia (Federated States of)"] = 'Micronesia'

map$location[map$location == "Macedonia"] = 'North Macedonia'

map$location[map$location == "Trinidad and Tobago"] = 'Trinidad'

a <- map[map$location == "Trinidad",]

a$location <- 'Tobago'

map <- rbind(map,a)

map$location[map$location == "Cabo Verde"] = 'Cape Verde'

map$location[map$location == "United States Virgin Islands"] = 'Virgin Islands'

map$location[map$location == "Antigua and Barbuda"] = 'Antigu'

a <- map[map$location == "Antigu",]

a$location <- 'Barbuda'

map <- rbind(map,a)

map$location[map$location == "Saint Kitts and Nevis"] = 'Saint Kitts'

a <- map[map$location == "Saint Kitts",]

a$location <- 'Nevis'

map <- rbind(map,a)

map$location[map$location == "Côte d'Ivoire"] = 'Ivory Coast'

map$location[map$location == "Saint Vincent and the Grenadines"] = 'Saint Vincent'

a <- map[map$location == "Saint Vincent",]

a$location <- 'Grenadines'

map <- rbind(map,a)

map$location[map$location == "Eswatini"] = 'Swaziland'

map$location[map$location == "Brunei Darussalam"] = 'Brunei'

map <- full_join(world,map,by = c('region'='location')) %>%

filter(val != "NA")

mycolor2<-brewer.pal(5, "Blues")

fig <- map %>%

ggplot()+

geom_polygon(aes(x = long, y = lat,group = group,fill=val2),colour="white",

size=0.2) +

theme_void()+

scale_fill_manual(values=mycolor2) +

theme(legend.position = c(0.1,0.2),

legend.title = element_blank(),

legend.text = element_text(color="black",

size = 12,

),

plot.title = element_blank(),

panel.grid=element_blank(),

axis.title.x = element_blank(),

axis.text.x = element_blank(),

axis.ticks.x = element_blank(),

axis.title.y = element_blank(),

axis.text.y = element_blank(),

axis.ticks.y = element_blank(),

)

fig

IS1 <- IS %>%

filter(measure == "DALYs") %>%

filter(year == 2021) %>%

filter(sex == "Both") %>%

filter(age == "Age-standardized") %>%

filter(metric == "Rate")

summary(IS1$val)

quantile(IS1$val,seq(0.1,1,0.1))

map <- IS1%>% mutate(val2 = cut(val, breaks = c(0,1,2,3,4,10000000),

labels = c("<1","1-2","2-3","3-4",">4"),

include.lowest = T,right = T))

map$location[map$location == 'United States of America'] = 'USA'

map$location[map$location == 'Russian Federation'] = 'Russia'

map$location[map$location == 'United Kingdom'] = 'UK'

map$location[map$location == 'Congo'] = 'Republic of Congo'

map$location[map$location == "Iran (Islamic Republic of)"] = 'Iran'

map$location[map$location == "Democratic People's Republic of Korea"] = 'North Korea'

map$location[map$location == "Taiwan (Province of China)"] = 'Taiwan'

map$location[map$location == "Republic of Korea"] = 'South Korea'

map$location[map$location == "United Republic of Tanzania"] = 'Tanzania'

map$location[map$location == "Bolivia (Plurinational State of)"] = 'Bolivia'

map$location[map$location == "Venezuela (Bolivarian Republic of)"] = 'Venezuela'

map$location[map$location == "Czechia"] = 'Czech Republic'

map$location[map$location == "Republic of Moldova"] = 'Moldova'

map$location[map$location == "Viet Nam"] = 'Vietnam'

map$location[map$location == "Lao People's Democratic Republic"] = 'Laos'

map$location[map$location == "Syrian Arab Republic"] = 'Syria'

map$location[map$location == "North Macedonia"] = 'Macedonia'

map$location[map$location == "Micronesia (Federated States of)"] = 'Micronesia'

map$location[map$location == "Macedonia"] = 'North Macedonia'

map$location[map$location == "Trinidad and Tobago"] = 'Trinidad'

a <- map[map$location == "Trinidad",]

a$location <- 'Tobago'

map <- rbind(map,a)

map$location[map$location == "Cabo Verde"] = 'Cape Verde'

map$location[map$location == "United States Virgin Islands"] = 'Virgin Islands'

map$location[map$location == "Antigua and Barbuda"] = 'Antigu'

a <- map[map$location == "Antigu",]

a$location <- 'Barbuda'

map <- rbind(map,a)

map$location[map$location == "Saint Kitts and Nevis"] = 'Saint Kitts'

a <- map[map$location == "Saint Kitts",]

a$location <- 'Nevis'

map <- rbind(map,a)

map$location[map$location == "Côte d'Ivoire"] = 'Ivory Coast'

map$location[map$location == "Saint Vincent and the Grenadines"] = 'Saint Vincent'

a <- map[map$location == "Saint Vincent",]

a$location <- 'Grenadines'

map <- rbind(map,a)

map$location[map$location == "Eswatini"] = 'Swaziland'

map$location[map$location == "Brunei Darussalam"] = 'Brunei'

map <- full_join(world,map,by = c('region'='location')) %>%

filter(val != "NA")

mycolor2<-brewer.pal(5, "Blues")

fig <- map %>%

ggplot()+

geom_polygon(aes(x = long, y = lat,group = group,fill=val2),colour="white",

size=0.2) +

theme_void()+

scale_fill_manual(values=mycolor2) +

theme(legend.position = c(0.1,0.2),

legend.title = element_blank(),

legend.text = element_text(color="black",

size = 12,

),

plot.title = element_blank(),

panel.grid=element_blank(),

axis.title.x = element_blank(),

axis.text.x = element_blank(),

axis.ticks.x = element_blank(),

axis.title.y = element_blank(),

axis.text.y = element_blank(),

axis.ticks.y = element_blank(),

)

fig

IS1 <- IS %>%

filter(measure == "Incidence") %>%

filter(year == 2021) %>%

filter(sex == "Both") %>%

filter(age == "All ages") %>%

filter(metric == "Number")

summary(IS1$val)

quantile(IS1$val,seq(0.1,1,0.1))

map <- IS1%>% mutate(val2 = cut(val, breaks = c(0,1,2,3,4,10000000),

labels = c("<1","1-2","2-3","3-4",">4"),

include.lowest = T,right = T))

map$location[map$location == 'United States of America'] = 'USA'

map$location[map$location == 'Russian Federation'] = 'Russia'

map$location[map$location == 'United Kingdom'] = 'UK'

map$location[map$location == 'Congo'] = 'Republic of Congo'

map$location[map$location == "Iran (Islamic Republic of)"] = 'Iran'

map$location[map$location == "Democratic People's Republic of Korea"] = 'North Korea'

map$location[map$location == "Taiwan (Province of China)"] = 'Taiwan'

map$location[map$location == "Republic of Korea"] = 'South Korea'

map$location[map$location == "United Republic of Tanzania"] = 'Tanzania'

map$location[map$location == "Bolivia (Plurinational State of)"] = 'Bolivia'

map$location[map$location == "Venezuela (Bolivarian Republic of)"] = 'Venezuela'

map$location[map$location == "Czechia"] = 'Czech Republic'

map$location[map$location == "Republic of Moldova"] = 'Moldova'

map$location[map$location == "Viet Nam"] = 'Vietnam'

map$location[map$location == "Lao People's Democratic Republic"] = 'Laos'

map$location[map$location == "Syrian Arab Republic"] = 'Syria'

map$location[map$location == "North Macedonia"] = 'Macedonia'

map$location[map$location == "Micronesia (Federated States of)"] = 'Micronesia'

map$location[map$location == "Macedonia"] = 'North Macedonia'

map$location[map$location == "Trinidad and Tobago"] = 'Trinidad'

a <- map[map$location == "Trinidad",]

a$location <- 'Tobago'

map <- rbind(map,a)

map$location[map$location == "Cabo Verde"] = 'Cape Verde'

map$location[map$location == "United States Virgin Islands"] = 'Virgin Islands'

map$location[map$location == "Antigua and Barbuda"] = 'Antigu'

a <- map[map$location == "Antigu",]

a$location <- 'Barbuda'

map <- rbind(map,a)

map$location[map$location == "Saint Kitts and Nevis"] = 'Saint Kitts'

a <- map[map$location == "Saint Kitts",]

a$location <- 'Nevis'

map <- rbind(map,a)

map$location[map$location == "Côte d'Ivoire"] = 'Ivory Coast'

map$location[map$location == "Saint Vincent and the Grenadines"] = 'Saint Vincent'

a <- map[map$location == "Saint Vincent",]

a$location <- 'Grenadines'

map <- rbind(map,a)

map$location[map$location == "Eswatini"] = 'Swaziland'

map$location[map$location == "Brunei Darussalam"] = 'Brunei'

map <- full_join(world,map,by = c('region'='location')) %>%

filter(val != "NA")

mycolor2<-brewer.pal(5, "Blues")

fig <- map %>%

ggplot()+

geom_polygon(aes(x = long, y = lat,group = group,fill=val2),colour="white",

size=0.2) +

theme_void()+

scale_fill_manual(values=mycolor2) +

theme(legend.position = c(0.1,0.2),

legend.title = element_blank(),

legend.text = element_text(color="black",

size = 12,

),

plot.title = element_blank(),

panel.grid=element_blank(),

axis.title.x = element_blank(),

axis.text.x = element_blank(),

axis.ticks.x = element_blank(),

axis.title.y = element_blank(),

axis.text.y = element_blank(),

axis.ticks.y = element_blank(),

)

fig

IS1 <- IS %>%

filter(measure == "Prevalence") %>%

filter(year == 2021) %>%

filter(sex == "Both") %>%

filter(age == "All ages") %>%

filter(metric == "Number")

summary(IS1$val)

quantile(IS1$val,seq(0.1,1,0.1))

map <- IS1%>% mutate(val2 = cut(val, breaks = c(0,1,2,3,4,10000000),

labels = c("<1","1-2","2-3","3-4",">4"),

include.lowest = T,right = T))

map$location[map$location == 'United States of America'] = 'USA'

map$location[map$location == 'Russian Federation'] = 'Russia'

map$location[map$location == 'United Kingdom'] = 'UK'

map$location[map$location == 'Congo'] = 'Republic of Congo'

map$location[map$location == "Iran (Islamic Republic of)"] = 'Iran'

map$location[map$location == "Democratic People's Republic of Korea"] = 'North Korea'

map$location[map$location == "Taiwan (Province of China)"] = 'Taiwan'

map$location[map$location == "Republic of Korea"] = 'South Korea'

map$location[map$location == "United Republic of Tanzania"] = 'Tanzania'

map$location[map$location == "Bolivia (Plurinational State of)"] = 'Bolivia'

map$location[map$location == "Venezuela (Bolivarian Republic of)"] = 'Venezuela'

map$location[map$location == "Czechia"] = 'Czech Republic'

map$location[map$location == "Republic of Moldova"] = 'Moldova'

map$location[map$location == "Viet Nam"] = 'Vietnam'

map$location[map$location == "Lao People's Democratic Republic"] = 'Laos'

map$location[map$location == "Syrian Arab Republic"] = 'Syria'

map$location[map$location == "North Macedonia"] = 'Macedonia'

map$location[map$location == "Micronesia (Federated States of)"] = 'Micronesia'

map$location[map$location == "Macedonia"] = 'North Macedonia'

map$location[map$location == "Trinidad and Tobago"] = 'Trinidad'

a <- map[map$location == "Trinidad",]

a$location <- 'Tobago'

map <- rbind(map,a)

map$location[map$location == "Cabo Verde"] = 'Cape Verde'

map$location[map$location == "United States Virgin Islands"] = 'Virgin Islands'

map$location[map$location == "Antigua and Barbuda"] = 'Antigu'

a <- map[map$location == "Antigu",]

a$location <- 'Barbuda'

map <- rbind(map,a)

map$location[map$location == "Saint Kitts and Nevis"] = 'Saint Kitts'

a <- map[map$location == "Saint Kitts",]

a$location <- 'Nevis'

map <- rbind(map,a)

map$location[map$location == "Côte d'Ivoire"] = 'Ivory Coast'

map$location[map$location == "Saint Vincent and the Grenadines"] = 'Saint Vincent'

a <- map[map$location == "Saint Vincent",]

a$location <- 'Grenadines'

map <- rbind(map,a)

map$location[map$location == "Eswatini"] = 'Swaziland'

map$location[map$location == "Brunei Darussalam"] = 'Brunei'

map <- full_join(world,map,by = c('region'='location')) %>%

filter(val != "NA")

mycolor2<-brewer.pal(5, "Blues")

fig <- map %>%

ggplot()+

geom_polygon(aes(x = long, y = lat,group = group,fill=val2),colour="white",

size=0.2) +

theme_void()+

scale_fill_manual(values=mycolor2) +

theme(legend.position = c(0.1,0.2),

legend.title = element_blank(),

legend.text = element_text(color="black",

size = 12,

),

plot.title = element_blank(),

panel.grid=element_blank(),

axis.title.x = element_blank(),

axis.text.x = element_blank(),

axis.ticks.x = element_blank(),

axis.title.y = element_blank(),

axis.text.y = element_blank(),

axis.ticks.y = element_blank(),

)

fig

IS1 <- IS %>%

filter(measure == "Deaths") %>%

filter(year == 2021) %>%

filter(sex == "Both") %>%

filter(age == "All ages") %>%

filter(metric == "Number")

summary(IS1$val)

quantile(IS1$val,seq(0.1,1,0.1))

map <- IS1%>% mutate(val2 = cut(val, breaks = c(0,1,2,3,4,10000000),

labels = c("<1","1-2","2-3","3-4",">4"),

include.lowest = T,right = T))

map$location[map$location == 'United States of America'] = 'USA'

map$location[map$location == 'Russian Federation'] = 'Russia'

map$location[map$location == 'United Kingdom'] = 'UK'

map$location[map$location == 'Congo'] = 'Republic of Congo'

map$location[map$location == "Iran (Islamic Republic of)"] = 'Iran'

map$location[map$location == "Democratic People's Republic of Korea"] = 'North Korea'

map$location[map$location == "Taiwan (Province of China)"] = 'Taiwan'

map$location[map$location == "Republic of Korea"] = 'South Korea'

map$location[map$location == "United Republic of Tanzania"] = 'Tanzania'

map$location[map$location == "Bolivia (Plurinational State of)"] = 'Bolivia'

map$location[map$location == "Venezuela (Bolivarian Republic of)"] = 'Venezuela'

map$location[map$location == "Czechia"] = 'Czech Republic'

map$location[map$location == "Republic of Moldova"] = 'Moldova'

map$location[map$location == "Viet Nam"] = 'Vietnam'

map$location[map$location == "Lao People's Democratic Republic"] = 'Laos'

map$location[map$location == "Syrian Arab Republic"] = 'Syria'

map$location[map$location == "North Macedonia"] = 'Macedonia'

map$location[map$location == "Micronesia (Federated States of)"] = 'Micronesia'

map$location[map$location == "Macedonia"] = 'North Macedonia'

map$location[map$location == "Trinidad and Tobago"] = 'Trinidad'

a <- map[map$location == "Trinidad",]

a$location <- 'Tobago'

map <- rbind(map,a)

map$location[map$location == "Cabo Verde"] = 'Cape Verde'

map$location[map$location == "United States Virgin Islands"] = 'Virgin Islands'

map$location[map$location == "Antigua and Barbuda"] = 'Antigu'

a <- map[map$location == "Antigu",]

a$location <- 'Barbuda'

map <- rbind(map,a)

map$location[map$location == "Saint Kitts and Nevis"] = 'Saint Kitts'

a <- map[map$location == "Saint Kitts",]

a$location <- 'Nevis'

map <- rbind(map,a)

map$location[map$location == "Côte d'Ivoire"] = 'Ivory Coast'

map$location[map$location == "Saint Vincent and the Grenadines"] = 'Saint Vincent'

a <- map[map$location == "Saint Vincent",]

a$location <- 'Grenadines'

map <- rbind(map,a)

map$location[map$location == "Eswatini"] = 'Swaziland'

map$location[map$location == "Brunei Darussalam"] = 'Brunei'

map <- full_join(world,map,by = c('region'='location')) %>%

filter(val != "NA")

mycolor2<-brewer.pal(5, "Blues")

fig <- map %>%

ggplot()+

geom_polygon(aes(x = long, y = lat,group = group,fill=val2),colour="white",

size=0.2) +

theme_void()+

scale_fill_manual(values=mycolor2) +

theme(legend.position = c(0.1,0.2),

legend.title = element_blank(),

legend.text = element_text(color="black",

size = 12,

),

plot.title = element_blank(),

panel.grid=element_blank(),

axis.title.x = element_blank(),

axis.text.x = element_blank(),

axis.ticks.x = element_blank(),

axis.title.y = element_blank(),

axis.text.y = element_blank(),

axis.ticks.y = element_blank(),

)

fig

IS1 <- IS %>%

filter(measure == "DALYs") %>%

filter(year == 2021) %>%

filter(sex == "Both") %>%

filter(age == "All ages") %>%

filter(metric == "Number")

summary(IS1$val)

quantile(IS1$val,seq(0.1,1,0.1))

map <- IS1%>% mutate(val2 = cut(val, breaks = c(0,1,2,3,4,10000000),

labels = c("<1","1-2","2-3","3-4",">4"),

include.lowest = T,right = T))

map$location[map$location == 'United States of America'] = 'USA'

map$location[map$location == 'Russian Federation'] = 'Russia'

map$location[map$location == 'United Kingdom'] = 'UK'

map$location[map$location == 'Congo'] = 'Republic of Congo'

map$location[map$location == "Iran (Islamic Republic of)"] = 'Iran'

map$location[map$location == "Democratic People's Republic of Korea"] = 'North Korea'

map$location[map$location == "Taiwan (Province of China)"] = 'Taiwan'

map$location[map$location == "Republic of Korea"] = 'South Korea'

map$location[map$location == "United Republic of Tanzania"] = 'Tanzania'

map$location[map$location == "Bolivia (Plurinational State of)"] = 'Bolivia'

map$location[map$location == "Venezuela (Bolivarian Republic of)"] = 'Venezuela'

map$location[map$location == "Czechia"] = 'Czech Republic'

map$location[map$location == "Republic of Moldova"] = 'Moldova'

map$location[map$location == "Viet Nam"] = 'Vietnam'

map$location[map$location == "Lao People's Democratic Republic"] = 'Laos'

map$location[map$location == "Syrian Arab Republic"] = 'Syria'

map$location[map$location == "North Macedonia"] = 'Macedonia'

map$location[map$location == "Micronesia (Federated States of)"] = 'Micronesia'

map$location[map$location == "Macedonia"] = 'North Macedonia'

map$location[map$location == "Trinidad and Tobago"] = 'Trinidad'

a <- map[map$location == "Trinidad",]

a$location <- 'Tobago'

map <- rbind(map,a)

map$location[map$location == "Cabo Verde"] = 'Cape Verde'

map$location[map$location == "United States Virgin Islands"] = 'Virgin Islands'

map$location[map$location == "Antigua and Barbuda"] = 'Antigu'

a <- map[map$location == "Antigu",]

a$location <- 'Barbuda'

map <- rbind(map,a)

map$location[map$location == "Saint Kitts and Nevis"] = 'Saint Kitts'

a <- map[map$location == "Saint Kitts",]

a$location <- 'Nevis'

map <- rbind(map,a)

map$location[map$location == "Côte d'Ivoire"] = 'Ivory Coast'

map$location[map$location == "Saint Vincent and the Grenadines"] = 'Saint Vincent'

a <- map[map$location == "Saint Vincent",]

a$location <- 'Grenadines'

map <- rbind(map,a)

map$location[map$location == "Eswatini"] = 'Swaziland'

map$location[map$location == "Brunei Darussalam"] = 'Brunei'

map <- full_join(world,map,by = c('region'='location')) %>%

filter(val != "NA")

mycolor2<-brewer.pal(5, "Blues")

fig <- map %>%

ggplot()+

geom_polygon(aes(x = long, y = lat,group = group,fill=val2),colour="white",

size=0.2) +

theme_void()+

scale_fill_manual(values=mycolor2) +

theme(legend.position = c(0.1,0.2),

legend.title = element_blank(),

legend.text = element_text(color="black",

size = 12,

),

plot.title = element_blank(),

panel.grid=element_blank(),

axis.title.x = element_blank(),

axis.text.x = element_blank(),

axis.ticks.x = element_blank(),

axis.title.y = element_blank(),

axis.text.y = element_blank(),

axis.ticks.y = element_blank(),

)

fig

world <- map_data('world')

AML_country<- vroom::vroom("AML_country.csv")

IS <- AML_country %>%

dplyr::select(measure_name,location_name,metric_name,

sex_name,age_name,year,val,lower,upper) %>%

rename(measure=measure_name,

location=location_name,

metric=metric_name,

sex=sex_name,

age=age_name)

IS1 <- IS %>%

filter(measure == "Incidence") %>%

filter(year == 2021) %>%

filter(sex == "Both") %>%

filter(age == "Age-standardized") %>%

filter(metric == "Rate")

summary(IS1$val)

quantile(IS1$val,seq(0.1,1,0.1))

map <- IS1%>% mutate(val2 = cut(val, breaks = c(0,1,2,3,4,10000000),

labels = c("<1","1-2","2-3","3-4",">4"),

include.lowest = T,right = T))

map$location[map$location == 'United States of America'] = 'USA'

map$location[map$location == 'Russian Federation'] = 'Russia'

map$location[map$location == 'United Kingdom'] = 'UK'

map$location[map$location == 'Congo'] = 'Republic of Congo'

map$location[map$location == "Iran (Islamic Republic of)"] = 'Iran'

map$location[map$location == "Democratic People's Republic of Korea"] = 'North Korea'

map$location[map$location == "Taiwan (Province of China)"] = 'Taiwan'

map$location[map$location == "Republic of Korea"] = 'South Korea'

map$location[map$location == "United Republic of Tanzania"] = 'Tanzania'

map$location[map$location == "Bolivia (Plurinational State of)"] = 'Bolivia'

map$location[map$location == "Venezuela (Bolivarian Republic of)"] = 'Venezuela'

map$location[map$location == "Czechia"] = 'Czech Republic'

map$location[map$location == "Republic of Moldova"] = 'Moldova'

map$location[map$location == "Viet Nam"] = 'Vietnam'

map$location[map$location == "Lao People's Democratic Republic"] = 'Laos'

map$location[map$location == "Syrian Arab Republic"] = 'Syria'

map$location[map$location == "North Macedonia"] = 'Macedonia'

map$location[map$location == "Micronesia (Federated States of)"] = 'Micronesia'

map$location[map$location == "Macedonia"] = 'North Macedonia'

map$location[map$location == "Trinidad and Tobago"] = 'Trinidad'

a <- map[map$location == "Trinidad",]

a$location <- 'Tobago'

map <- rbind(map,a)

map$location[map$location == "Cabo Verde"] = 'Cape Verde'

map$location[map$location == "United States Virgin Islands"] = 'Virgin Islands'

map$location[map$location == "Antigua and Barbuda"] = 'Antigu'

a <- map[map$location == "Antigu",]

a$location <- 'Barbuda'

map <- rbind(map,a)

map$location[map$location == "Saint Kitts and Nevis"] = 'Saint Kitts'

a <- map[map$location == "Saint Kitts",]

a$location <- 'Nevis'

map <- rbind(map,a)

map$location[map$location == "Côte d'Ivoire"] = 'Ivory Coast'

map$location[map$location == "Saint Vincent and the Grenadines"] = 'Saint Vincent'

a <- map[map$location == "Saint Vincent",]

a$location <- 'Grenadines'

map <- rbind(map,a)

map$location[map$location == "Eswatini"] = 'Swaziland'

map$location[map$location == "Brunei Darussalam"] = 'Brunei'

map <- full_join(world,map,by = c('region'='location')) %>%

filter(val != "NA")

mycolor2<-brewer.pal(5, "Blues")

fig <- map %>%

ggplot()+

geom_polygon(aes(x = long, y = lat,group = group,fill=val2),colour="white",

size=0.2) +

theme_void()+

scale_fill_manual(values=mycolor2) +

theme(legend.position = c(0.1,0.2),

legend.title = element_blank(),

legend.text = element_text(color="black",

size = 12,

),

plot.title = element_blank(),

panel.grid=element_blank(),

axis.title.x = element_blank(),

axis.text.x = element_blank(),

axis.ticks.x = element_blank(),

axis.title.y = element_blank(),

axis.text.y = element_blank(),

axis.ticks.y = element_blank(),

)

fig

IS1 <- IS %>%

filter(measure == "Prevalence") %>%

filter(year == 2021) %>%

filter(sex == "Both") %>%

filter(age == "Age-standardized") %>%

filter(metric == "Rate")

summary(IS1$val)

quantile(IS1$val,seq(0.1,1,0.1))

map <- IS1%>% mutate(val2 = cut(val, breaks = c(0,1,2,3,4,10000000),

labels = c("<1","1-2","2-3","3-4",">4"),

include.lowest = T,right = T))

map$location[map$location == 'United States of America'] = 'USA'

map$location[map$location == 'Russian Federation'] = 'Russia'

map$location[map$location == 'United Kingdom'] = 'UK'

map$location[map$location == 'Congo'] = 'Republic of Congo'

map$location[map$location == "Iran (Islamic Republic of)"] = 'Iran'

map$location[map$location == "Democratic People's Republic of Korea"] = 'North Korea'

map$location[map$location == "Taiwan (Province of China)"] = 'Taiwan'

map$location[map$location == "Republic of Korea"] = 'South Korea'

map$location[map$location == "United Republic of Tanzania"] = 'Tanzania'

map$location[map$location == "Bolivia (Plurinational State of)"] = 'Bolivia'

map$location[map$location == "Venezuela (Bolivarian Republic of)"] = 'Venezuela'

map$location[map$location == "Czechia"] = 'Czech Republic'

map$location[map$location == "Republic of Moldova"] = 'Moldova'

map$location[map$location == "Viet Nam"] = 'Vietnam'

map$location[map$location == "Lao People's Democratic Republic"] = 'Laos'

map$location[map$location == "Syrian Arab Republic"] = 'Syria'

map$location[map$location == "North Macedonia"] = 'Macedonia'

map$location[map$location == "Micronesia (Federated States of)"] = 'Micronesia'

map$location[map$location == "Macedonia"] = 'North Macedonia'

map$location[map$location == "Trinidad and Tobago"] = 'Trinidad'

a <- map[map$location == "Trinidad",]

a$location <- 'Tobago'

map <- rbind(map,a)

map$location[map$location == "Cabo Verde"] = 'Cape Verde'

map$location[map$location == "United States Virgin Islands"] = 'Virgin Islands'

map$location[map$location == "Antigua and Barbuda"] = 'Antigu'

a <- map[map$location == "Antigu",]

a$location <- 'Barbuda'

map <- rbind(map,a)

map$location[map$location == "Saint Kitts and Nevis"] = 'Saint Kitts'

a <- map[map$location == "Saint Kitts",]

a$location <- 'Nevis'

map <- rbind(map,a)

map$location[map$location == "Côte d'Ivoire"] = 'Ivory Coast'

map$location[map$location == "Saint Vincent and the Grenadines"] = 'Saint Vincent'

a <- map[map$location == "Saint Vincent",]

a$location <- 'Grenadines'

map <- rbind(map,a)

map$location[map$location == "Eswatini"] = 'Swaziland'

map$location[map$location == "Brunei Darussalam"] = 'Brunei'

map <- full_join(world,map,by = c('region'='location')) %>%

filter(val != "NA")

mycolor2<-brewer.pal(5, "Blues")

fig <- map %>%

ggplot()+

geom_polygon(aes(x = long, y = lat,group = group,fill=val2),colour="white",

size=0.2) +

theme_void()+

scale_fill_manual(values=mycolor2) +

theme(legend.position = c(0.1,0.2),

legend.title = element_blank(),

legend.text = element_text(color="black",

size = 12,

),

plot.title = element_blank(),

panel.grid=element_blank(),

axis.title.x = element_blank(),

axis.text.x = element_blank(),

axis.ticks.x = element_blank(),

axis.title.y = element_blank(),

axis.text.y = element_blank(),

axis.ticks.y = element_blank(),

)

fig

IS1 <- IS %>%

filter(measure == "Deaths") %>%

filter(year == 2021) %>%

filter(sex == "Both") %>%

filter(age == "Age-standardized") %>%

filter(metric == "Rate")

summary(IS1$val)

quantile(IS1$val,seq(0.1,1,0.1))

map <- IS1%>% mutate(val2 = cut(val, breaks = c(0,1,2,3,4,10000000),

labels = c("<1","1-2","2-3","3-4",">4"),

include.lowest = T,right = T))

map$location[map$location == 'United States of America'] = 'USA'

map$location[map$location == 'Russian Federation'] = 'Russia'

map$location[map$location == 'United Kingdom'] = 'UK'

map$location[map$location == 'Congo'] = 'Republic of Congo'

map$location[map$location == "Iran (Islamic Republic of)"] = 'Iran'

map$location[map$location == "Democratic People's Republic of Korea"] = 'North Korea'

map$location[map$location == "Taiwan (Province of China)"] = 'Taiwan'

map$location[map$location == "Republic of Korea"] = 'South Korea'

map$location[map$location == "United Republic of Tanzania"] = 'Tanzania'

map$location[map$location == "Bolivia (Plurinational State of)"] = 'Bolivia'

map$location[map$location == "Venezuela (Bolivarian Republic of)"] = 'Venezuela'

map$location[map$location == "Czechia"] = 'Czech Republic'

map$location[map$location == "Republic of Moldova"] = 'Moldova'

map$location[map$location == "Viet Nam"] = 'Vietnam'

map$location[map$location == "Lao People's Democratic Republic"] = 'Laos'

map$location[map$location == "Syrian Arab Republic"] = 'Syria'

map$location[map$location == "North Macedonia"] = 'Macedonia'

map$location[map$location == "Micronesia (Federated States of)"] = 'Micronesia'

map$location[map$location == "Macedonia"] = 'North Macedonia'

map$location[map$location == "Trinidad and Tobago"] = 'Trinidad'

a <- map[map$location == "Trinidad",]

a$location <- 'Tobago'

map <- rbind(map,a)

map$location[map$location == "Cabo Verde"] = 'Cape Verde'

map$location[map$location == "United States Virgin Islands"] = 'Virgin Islands'

map$location[map$location == "Antigua and Barbuda"] = 'Antigu'

a <- map[map$location == "Antigu",]

a$location <- 'Barbuda'

map <- rbind(map,a)

map$location[map$location == "Saint Kitts and Nevis"] = 'Saint Kitts'

a <- map[map$location == "Saint Kitts",]

a$location <- 'Nevis'

map <- rbind(map,a)

map$location[map$location == "Côte d'Ivoire"] = 'Ivory Coast'

map$location[map$location == "Saint Vincent and the Grenadines"] = 'Saint Vincent'

a <- map[map$location == "Saint Vincent",]

a$location <- 'Grenadines'

map <- rbind(map,a)

map$location[map$location == "Eswatini"] = 'Swaziland'

map$location[map$location == "Brunei Darussalam"] = 'Brunei'

map <- full_join(world,map,by = c('region'='location')) %>%

filter(val != "NA")

mycolor2<-brewer.pal(5, "Blues")

fig <- map %>%

ggplot()+

geom_polygon(aes(x = long, y = lat,group = group,fill=val2),colour="white",

size=0.2) +

theme_void()+

scale_fill_manual(values=mycolor2) +

theme(legend.position = c(0.1,0.2),

legend.title = element_blank(),

legend.text = element_text(color="black",

size = 12,

),

plot.title = element_blank(),

panel.grid=element_blank(),

axis.title.x = element_blank(),

axis.text.x = element_blank(),

axis.ticks.x = element_blank(),

axis.title.y = element_blank(),

axis.text.y = element_blank(),

axis.ticks.y = element_blank(),

)

fig

IS1 <- IS %>%

filter(measure == "DALYs") %>%

filter(year == 2021) %>%

filter(sex == "Both") %>%

filter(age == "Age-standardized") %>%

filter(metric == "Rate")

summary(IS1$val)

quantile(IS1$val,seq(0.1,1,0.1))

map <- IS1%>% mutate(val2 = cut(val, breaks = c(0,1,2,3,4,10000000),

labels = c("<1","1-2","2-3","3-4",">4"),

include.lowest = T,right = T))

map$location[map$location == 'United States of America'] = 'USA'

map$location[map$location == 'Russian Federation'] = 'Russia'

map$location[map$location == 'United Kingdom'] = 'UK'

map$location[map$location == 'Congo'] = 'Republic of Congo'

map$location[map$location == "Iran (Islamic Republic of)"] = 'Iran'

map$location[map$location == "Democratic People's Republic of Korea"] = 'North Korea'

map$location[map$location == "Taiwan (Province of China)"] = 'Taiwan'

map$location[map$location == "Republic of Korea"] = 'South Korea'

map$location[map$location == "United Republic of Tanzania"] = 'Tanzania'

map$location[map$location == "Bolivia (Plurinational State of)"] = 'Bolivia'

map$location[map$location == "Venezuela (Bolivarian Republic of)"] = 'Venezuela'

map$location[map$location == "Czechia"] = 'Czech Republic'

map$location[map$location == "Republic of Moldova"] = 'Moldova'

map$location[map$location == "Viet Nam"] = 'Vietnam'

map$location[map$location == "Lao People's Democratic Republic"] = 'Laos'

map$location[map$location == "Syrian Arab Republic"] = 'Syria'

map$location[map$location == "North Macedonia"] = 'Macedonia'

map$location[map$location == "Micronesia (Federated States of)"] = 'Micronesia'

map$location[map$location == "Macedonia"] = 'North Macedonia'

map$location[map$location == "Trinidad and Tobago"] = 'Trinidad'

a <- map[map$location == "Trinidad",]

a$location <- 'Tobago'

map <- rbind(map,a)

map$location[map$location == "Cabo Verde"] = 'Cape Verde'

map$location[map$location == "United States Virgin Islands"] = 'Virgin Islands'

map$location[map$location == "Antigua and Barbuda"] = 'Antigu'

a <- map[map$location == "Antigu",]

a$location <- 'Barbuda'

map <- rbind(map,a)

map$location[map$location == "Saint Kitts and Nevis"] = 'Saint Kitts'

a <- map[map$location == "Saint Kitts",]

a$location <- 'Nevis'

map <- rbind(map,a)

map$location[map$location == "Côte d'Ivoire"] = 'Ivory Coast'

map$location[map$location == "Saint Vincent and the Grenadines"] = 'Saint Vincent'

a <- map[map$location == "Saint Vincent",]

a$location <- 'Grenadines'

map <- rbind(map,a)

map$location[map$location == "Eswatini"] = 'Swaziland'

map$location[map$location == "Brunei Darussalam"] = 'Brunei'

map <- full_join(world,map,by = c('region'='location')) %>%

filter(val != "NA")

mycolor2<-brewer.pal(5, "Blues")

fig <- map %>%

ggplot()+

geom_polygon(aes(x = long, y = lat,group = group,fill=val2),colour="white",

size=0.2) +

theme_void()+

scale_fill_manual(values=mycolor2) +

theme(legend.position = c(0.1,0.2),

legend.title = element_blank(),

legend.text = element_text(color="black",

size = 12,

),

plot.title = element_blank(),

panel.grid=element_blank(),

axis.title.x = element_blank(),

axis.text.x = element_blank(),

axis.ticks.x = element_blank(),

axis.title.y = element_blank(),

axis.text.y = element_blank(),

axis.ticks.y = element_blank(),

)

fig

IS1 <- IS %>%

filter(measure == "Incidence") %>%

filter(year == 2021) %>%

filter(sex == "Both") %>%

filter(age == "All ages") %>%

filter(metric == "Number")

summary(IS1$val)

quantile(IS1$val,seq(0.1,1,0.1))

map <- IS1%>% mutate(val2 = cut(val, breaks = c(0,1,2,3,4,10000000),

labels = c("<1","1-2","2-3","3-4",">4"),

include.lowest = T,right = T))

map$location[map$location == 'United States of America'] = 'USA'

map$location[map$location == 'Russian Federation'] = 'Russia'

map$location[map$location == 'United Kingdom'] = 'UK'

map$location[map$location == 'Congo'] = 'Republic of Congo'

map$location[map$location == "Iran (Islamic Republic of)"] = 'Iran'

map$location[map$location == "Democratic People's Republic of Korea"] = 'North Korea'

map$location[map$location == "Taiwan (Province of China)"] = 'Taiwan'

map$location[map$location == "Republic of Korea"] = 'South Korea'

map$location[map$location == "United Republic of Tanzania"] = 'Tanzania'

map$location[map$location == "Bolivia (Plurinational State of)"] = 'Bolivia'

map$location[map$location == "Venezuela (Bolivarian Republic of)"] = 'Venezuela'

map$location[map$location == "Czechia"] = 'Czech Republic'

map$location[map$location == "Republic of Moldova"] = 'Moldova'

map$location[map$location == "Viet Nam"] = 'Vietnam'

map$location[map$location == "Lao People's Democratic Republic"] = 'Laos'

map$location[map$location == "Syrian Arab Republic"] = 'Syria'

map$location[map$location == "North Macedonia"] = 'Macedonia'

map$location[map$location == "Micronesia (Federated States of)"] = 'Micronesia'

map$location[map$location == "Macedonia"] = 'North Macedonia'

map$location[map$location == "Trinidad and Tobago"] = 'Trinidad'

a <- map[map$location == "Trinidad",]

a$location <- 'Tobago'

map <- rbind(map,a)

map$location[map$location == "Cabo Verde"] = 'Cape Verde'

map$location[map$location == "United States Virgin Islands"] = 'Virgin Islands'

map$location[map$location == "Antigua and Barbuda"] = 'Antigu'

a <- map[map$location == "Antigu",]

a$location <- 'Barbuda'

map <- rbind(map,a)

map$location[map$location == "Saint Kitts and Nevis"] = 'Saint Kitts'

a <- map[map$location == "Saint Kitts",]

a$location <- 'Nevis'

map <- rbind(map,a)

map$location[map$location == "Côte d'Ivoire"] = 'Ivory Coast'

map$location[map$location == "Saint Vincent and the Grenadines"] = 'Saint Vincent'

a <- map[map$location == "Saint Vincent",]

a$location <- 'Grenadines'

map <- rbind(map,a)

map$location[map$location == "Eswatini"] = 'Swaziland'

map$location[map$location == "Brunei Darussalam"] = 'Brunei'

map <- full_join(world,map,by = c('region'='location')) %>%

filter(val != "NA")

mycolor2<-brewer.pal(5, "Blues")

fig <- map %>%

ggplot()+

geom_polygon(aes(x = long, y = lat,group = group,fill=val2),colour="white",

size=0.2) +

theme_void()+

scale_fill_manual(values=mycolor2) +

theme(legend.position = c(0.1,0.2),

legend.title = element_blank(),

legend.text = element_text(color="black",

size = 12,

),

plot.title = element_blank(),

panel.grid=element_blank(),

axis.title.x = element_blank(),

axis.text.x = element_blank(),

axis.ticks.x = element_blank(),

axis.title.y = element_blank(),

axis.text.y = element_blank(),

axis.ticks.y = element_blank(),

)

fig

IS1 <- IS %>%

filter(measure == "Prevalence") %>%

filter(year == 2021) %>%

filter(sex == "Both") %>%

filter(age == "All ages") %>%

filter(metric == "Number")

summary(IS1$val)

quantile(IS1$val,seq(0.1,1,0.1))

map <- IS1%>% mutate(val2 = cut(val, breaks = c(0,1,2,3,4,10000000),

labels = c("<1","1-2","2-3","3-4",">4"),

include.lowest = T,right = T))

map$location[map$location == 'United States of America'] = 'USA'

map$location[map$location == 'Russian Federation'] = 'Russia'

map$location[map$location == 'United Kingdom'] = 'UK'

map$location[map$location == 'Congo'] = 'Republic of Congo'

map$location[map$location == "Iran (Islamic Republic of)"] = 'Iran'

map$location[map$location == "Democratic People's Republic of Korea"] = 'North Korea'

map$location[map$location == "Taiwan (Province of China)"] = 'Taiwan'

map$location[map$location == "Republic of Korea"] = 'South Korea'

map$location[map$location == "United Republic of Tanzania"] = 'Tanzania'

map$location[map$location == "Bolivia (Plurinational State of)"] = 'Bolivia'

map$location[map$location == "Venezuela (Bolivarian Republic of)"] = 'Venezuela'

map$location[map$location == "Czechia"] = 'Czech Republic'

map$location[map$location == "Republic of Moldova"] = 'Moldova'

map$location[map$location == "Viet Nam"] = 'Vietnam'

map$location[map$location == "Lao People's Democratic Republic"] = 'Laos'

map$location[map$location == "Syrian Arab Republic"] = 'Syria'

map$location[map$location == "North Macedonia"] = 'Macedonia'

map$location[map$location == "Micronesia (Federated States of)"] = 'Micronesia'

map$location[map$location == "Macedonia"] = 'North Macedonia'

map$location[map$location == "Trinidad and Tobago"] = 'Trinidad'

a <- map[map$location == "Trinidad",]

a$location <- 'Tobago'

map <- rbind(map,a)

map$location[map$location == "Cabo Verde"] = 'Cape Verde'

map$location[map$location == "United States Virgin Islands"] = 'Virgin Islands'

map$location[map$location == "Antigua and Barbuda"] = 'Antigu'

a <- map[map$location == "Antigu",]

a$location <- 'Barbuda'

map <- rbind(map,a)

map$location[map$location == "Saint Kitts and Nevis"] = 'Saint Kitts'

a <- map[map$location == "Saint Kitts",]

a$location <- 'Nevis'

map <- rbind(map,a)

map$location[map$location == "Côte d'Ivoire"] = 'Ivory Coast'

map$location[map$location == "Saint Vincent and the Grenadines"] = 'Saint Vincent'

a <- map[map$location == "Saint Vincent",]

a$location <- 'Grenadines'

map <- rbind(map,a)

map$location[map$location == "Eswatini"] = 'Swaziland'

map$location[map$location == "Brunei Darussalam"] = 'Brunei'

map <- full_join(world,map,by = c('region'='location')) %>%

filter(val != "NA")

mycolor2<-brewer.pal(5, "Blues")

fig <- map %>%

ggplot()+

geom_polygon(aes(x = long, y = lat,group = group,fill=val2),colour="white",

size=0.2) +

theme_void()+

scale_fill_manual(values=mycolor2) +

theme(legend.position = c(0.1,0.2),

legend.title = element_blank(),

legend.text = element_text(color="black",

size = 12,

),

plot.title = element_blank(),

panel.grid=element_blank(),

axis.title.x = element_blank(),

axis.text.x = element_blank(),

axis.ticks.x = element_blank(),

axis.title.y = element_blank(),

axis.text.y = element_blank(),

axis.ticks.y = element_blank(),

)

fig

IS1 <- IS %>%

filter(measure == "Deaths") %>%

filter(year == 2021) %>%

filter(sex == "Both") %>%

filter(age == "All ages") %>%

filter(metric == "Number")

summary(IS1$val)

quantile(IS1$val,seq(0.1,1,0.1))

map <- IS1%>% mutate(val2 = cut(val, breaks = c(0,1,2,3,4,10000000),

labels = c("<1","1-2","2-3","3-4",">4"),

include.lowest = T,right = T))

map$location[map$location == 'United States of America'] = 'USA'

map$location[map$location == 'Russian Federation'] = 'Russia'

map$location[map$location == 'United Kingdom'] = 'UK'

map$location[map$location == 'Congo'] = 'Republic of Congo'

map$location[map$location == "Iran (Islamic Republic of)"] = 'Iran'

map$location[map$location == "Democratic People's Republic of Korea"] = 'North Korea'

map$location[map$location == "Taiwan (Province of China)"] = 'Taiwan'

map$location[map$location == "Republic of Korea"] = 'South Korea'

map$location[map$location == "United Republic of Tanzania"] = 'Tanzania'

map$location[map$location == "Bolivia (Plurinational State of)"] = 'Bolivia'

map$location[map$location == "Venezuela (Bolivarian Republic of)"] = 'Venezuela'

map$location[map$location == "Czechia"] = 'Czech Republic'

map$location[map$location == "Republic of Moldova"] = 'Moldova'

map$location[map$location == "Viet Nam"] = 'Vietnam'

map$location[map$location == "Lao People's Democratic Republic"] = 'Laos'

map$location[map$location == "Syrian Arab Republic"] = 'Syria'

map$location[map$location == "North Macedonia"] = 'Macedonia'

map$location[map$location == "Micronesia (Federated States of)"] = 'Micronesia'

map$location[map$location == "Macedonia"] = 'North Macedonia'

map$location[map$location == "Trinidad and Tobago"] = 'Trinidad'

a <- map[map$location == "Trinidad",]

a$location <- 'Tobago'

map <- rbind(map,a)

map$location[map$location == "Cabo Verde"] = 'Cape Verde'

map$location[map$location == "United States Virgin Islands"] = 'Virgin Islands'

map$location[map$location == "Antigua and Barbuda"] = 'Antigu'

a <- map[map$location == "Antigu",]

a$location <- 'Barbuda'

map <- rbind(map,a)

map$location[map$location == "Saint Kitts and Nevis"] = 'Saint Kitts'

a <- map[map$location == "Saint Kitts",]

a$location <- 'Nevis'

map <- rbind(map,a)

map$location[map$location == "Côte d'Ivoire"] = 'Ivory Coast'

map$location[map$location == "Saint Vincent and the Grenadines"] = 'Saint Vincent'

a <- map[map$location == "Saint Vincent",]

a$location <- 'Grenadines'

map <- rbind(map,a)

map$location[map$location == "Eswatini"] = 'Swaziland'

map$location[map$location == "Brunei Darussalam"] = 'Brunei'

map <- full_join(world,map,by = c('region'='location')) %>%

filter(val != "NA")

mycolor2<-brewer.pal(5, "Blues")

fig <- map %>%

ggplot()+

geom_polygon(aes(x = long, y = lat,group = group,fill=val2),colour="white",

size=0.2) +

theme_void()+

scale_fill_manual(values=mycolor2) +

theme(legend.position = c(0.1,0.2),

legend.title = element_blank(),

legend.text = element_text(color="black",

size = 12,

),

plot.title = element_blank(),

panel.grid=element_blank(),

axis.title.x = element_blank(),

axis.text.x = element_blank(),

axis.ticks.x = element_blank(),

axis.title.y = element_blank(),

axis.text.y = element_blank(),

axis.ticks.y = element_blank(),

)

fig

IS1 <- IS %>%

filter(measure == "DALYs") %>%

filter(year == 2021) %>%

filter(sex == "Both") %>%

filter(age == "All ages") %>%

filter(metric == "Number")

summary(IS1$val)

quantile(IS1$val,seq(0.1,1,0.1))

map <- IS1%>% mutate(val2 = cut(val, breaks = c(0,1,2,3,4,10000000),

labels = c("<1","1-2","2-3","3-4",">4"),

include.lowest = T,right = T))

map$location[map$location == 'United States of America'] = 'USA'

map$location[map$location == 'Russian Federation'] = 'Russia'

map$location[map$location == 'United Kingdom'] = 'UK'

map$location[map$location == 'Congo'] = 'Republic of Congo'

map$location[map$location == "Iran (Islamic Republic of)"] = 'Iran'

map$location[map$location == "Democratic People's Republic of Korea"] = 'North Korea'

map$location[map$location == "Taiwan (Province of China)"] = 'Taiwan'

map$location[map$location == "Republic of Korea"] = 'South Korea'

map$location[map$location == "United Republic of Tanzania"] = 'Tanzania'

map$location[map$location == "Bolivia (Plurinational State of)"] = 'Bolivia'

map$location[map$location == "Venezuela (Bolivarian Republic of)"] = 'Venezuela'

map$location[map$location == "Czechia"] = 'Czech Republic'

map$location[map$location == "Republic of Moldova"] = 'Moldova'

map$location[map$location == "Viet Nam"] = 'Vietnam'

map$location[map$location == "Lao People's Democratic Republic"] = 'Laos'

map$location[map$location == "Syrian Arab Republic"] = 'Syria'

map$location[map$location == "North Macedonia"] = 'Macedonia'

map$location[map$location == "Micronesia (Federated States of)"] = 'Micronesia'

map$location[map$location == "Macedonia"] = 'North Macedonia'

map$location[map$location == "Trinidad and Tobago"] = 'Trinidad'

a <- map[map$location == "Trinidad",]

a$location <- 'Tobago'

map <- rbind(map,a)

map$location[map$location == "Cabo Verde"] = 'Cape Verde'

map$location[map$location == "United States Virgin Islands"] = 'Virgin Islands'

map$location[map$location == "Antigua and Barbuda"] = 'Antigu'

a <- map[map$location == "Antigu",]

a$location <- 'Barbuda'

map <- rbind(map,a)

map$location[map$location == "Saint Kitts and Nevis"] = 'Saint Kitts'

a <- map[map$location == "Saint Kitts",]

a$location <- 'Nevis'

map <- rbind(map,a)

map$location[map$location == "Côte d'Ivoire"] = 'Ivory Coast'

map$location[map$location == "Saint Vincent and the Grenadines"] = 'Saint Vincent'

a <- map[map$location == "Saint Vincent",]

a$location <- 'Grenadines'

map <- rbind(map,a)

map$location[map$location == "Eswatini"] = 'Swaziland'

map$location[map$location == "Brunei Darussalam"] = 'Brunei'

map <- full_join(world,map,by = c('region'='location')) %>%

filter(val != "NA")

mycolor2<-brewer.pal(5, "Blues")

fig <- map %>%

ggplot()+

geom_polygon(aes(x = long, y = lat,group = group,fill=val2),colour="white",

size=0.2) +

theme_void()+

scale_fill_manual(values=mycolor2) +

theme(legend.position = c(0.1,0.2),

legend.title = element_blank(),

legend.text = element_text(color="black",

size = 12,

),

plot.title = element_blank(),

panel.grid=element_blank(),

axis.title.x = element_blank(),

axis.text.x = element_blank(),

axis.ticks.x = element_blank(),

axis.title.y = element_blank(),

axis.text.y = element_blank(),

axis.ticks.y = element_blank(),

)

fig

world <- map_data('world')

CML_country<- vroom::vroom("CML_country.csv")

IS <- CML_country %>%

dplyr::select(measure_name,location_name,metric_name,

sex_name,age_name,year,val,lower,upper) %>%

rename(measure=measure_name,

location=location_name,

metric=metric_name,

sex=sex_name,

age=age_name)

IS1 <- IS %>%

filter(measure == "Incidence") %>%

filter(year == 2021) %>%

filter(sex == "Both") %>%

filter(age == "Age-standardized") %>%

filter(metric == "Rate")

summary(IS1$val)

quantile(IS1$val,seq(0.1,1,0.1))

map <- IS1%>% mutate(val2 = cut(val, breaks = c(0,1,2,3,4,10000000),

labels = c("<1","1-2","2-3","3-4",">4"),

include.lowest = T,right = T))

map$location[map$location == 'United States of America'] = 'USA'

map$location[map$location == 'Russian Federation'] = 'Russia'

map$location[map$location == 'United Kingdom'] = 'UK'

map$location[map$location == 'Congo'] = 'Republic of Congo'

map$location[map$location == "Iran (Islamic Republic of)"] = 'Iran'

map$location[map$location == "Democratic People's Republic of Korea"] = 'North Korea'

map$location[map$location == "Taiwan (Province of China)"] = 'Taiwan'

map$location[map$location == "Republic of Korea"] = 'South Korea'

map$location[map$location == "United Republic of Tanzania"] = 'Tanzania'

map$location[map$location == "Bolivia (Plurinational State of)"] = 'Bolivia'

map$location[map$location == "Venezuela (Bolivarian Republic of)"] = 'Venezuela'

map$location[map$location == "Czechia"] = 'Czech Republic'

map$location[map$location == "Republic of Moldova"] = 'Moldova'

map$location[map$location == "Viet Nam"] = 'Vietnam'

map$location[map$location == "Lao People's Democratic Republic"] = 'Laos'

map$location[map$location == "Syrian Arab Republic"] = 'Syria'

map$location[map$location == "North Macedonia"] = 'Macedonia'

map$location[map$location == "Micronesia (Federated States of)"] = 'Micronesia'

map$location[map$location == "Macedonia"] = 'North Macedonia'

map$location[map$location == "Trinidad and Tobago"] = 'Trinidad'

a <- map[map$location == "Trinidad",]

a$location <- 'Tobago'

map <- rbind(map,a)

map$location[map$location == "Cabo Verde"] = 'Cape Verde'

map$location[map$location == "United States Virgin Islands"] = 'Virgin Islands'

map$location[map$location == "Antigua and Barbuda"] = 'Antigu'

a <- map[map$location == "Antigu",]

a$location <- 'Barbuda'

map <- rbind(map,a)

map$location[map$location == "Saint Kitts and Nevis"] = 'Saint Kitts'

a <- map[map$location == "Saint Kitts",]

a$location <- 'Nevis'

map <- rbind(map,a)

map$location[map$location == "Côte d'Ivoire"] = 'Ivory Coast'

map$location[map$location == "Saint Vincent and the Grenadines"] = 'Saint Vincent'

a <- map[map$location == "Saint Vincent",]

a$location <- 'Grenadines'

map <- rbind(map,a)

map$location[map$location == "Eswatini"] = 'Swaziland'

map$location[map$location == "Brunei Darussalam"] = 'Brunei'

map <- full_join(world,map,by = c('region'='location')) %>%

filter(val != "NA")

mycolor2<-brewer.pal(5, "Blues")

fig <- map %>%

ggplot()+

geom_polygon(aes(x = long, y = lat,group = group,fill=val2),colour="white",

size=0.2) +

theme_void()+

scale_fill_manual(values=mycolor2) +

theme(legend.position = c(0.1,0.2),

legend.title = element_blank(),

legend.text = element_text(color="black",

size = 12,

),

plot.title = element_blank(),

panel.grid=element_blank(),

axis.title.x = element_blank(),

axis.text.x = element_blank(),

axis.ticks.x = element_blank(),

axis.title.y = element_blank(),

axis.text.y = element_blank(),

axis.ticks.y = element_blank(),

)

fig

IS1 <- IS %>%

filter(measure == "Prevalence") %>%

filter(year == 2021) %>%

filter(sex == "Both") %>%

filter(age == "Age-standardized") %>%

filter(metric == "Rate")

summary(IS1$val)

quantile(IS1$val,seq(0.1,1,0.1))

map <- IS1%>% mutate(val2 = cut(val, breaks = c(0,1,2,3,4,10000000),

labels = c("<1","1-2","2-3","3-4",">4"),

include.lowest = T,right = T))

map$location[map$location == 'United States of America'] = 'USA'

map$location[map$location == 'Russian Federation'] = 'Russia'

map$location[map$location == 'United Kingdom'] = 'UK'

map$location[map$location == 'Congo'] = 'Republic of Congo'

map$location[map$location == "Iran (Islamic Republic of)"] = 'Iran'

map$location[map$location == "Democratic People's Republic of Korea"] = 'North Korea'

map$location[map$location == "Taiwan (Province of China)"] = 'Taiwan'

map$location[map$location == "Republic of Korea"] = 'South Korea'

map$location[map$location == "United Republic of Tanzania"] = 'Tanzania'

map$location[map$location == "Bolivia (Plurinational State of)"] = 'Bolivia'

map$location[map$location == "Venezuela (Bolivarian Republic of)"] = 'Venezuela'

map$location[map$location == "Czechia"] = 'Czech Republic'

map$location[map$location == "Republic of Moldova"] = 'Moldova'

map$location[map$location == "Viet Nam"] = 'Vietnam'

map$location[map$location == "Lao People's Democratic Republic"] = 'Laos'

map$location[map$location == "Syrian Arab Republic"] = 'Syria'

map$location[map$location == "North Macedonia"] = 'Macedonia'

map$location[map$location == "Micronesia (Federated States of)"] = 'Micronesia'

map$location[map$location == "Macedonia"] = 'North Macedonia'

map$location[map$location == "Trinidad and Tobago"] = 'Trinidad'

a <- map[map$location == "Trinidad",]

a$location <- 'Tobago'

map <- rbind(map,a)

map$location[map$location == "Cabo Verde"] = 'Cape Verde'

map$location[map$location == "United States Virgin Islands"] = 'Virgin Islands'

map$location[map$location == "Antigua and Barbuda"] = 'Antigu'

a <- map[map$location == "Antigu",]

a$location <- 'Barbuda'

map <- rbind(map,a)

map$location[map$location == "Saint Kitts and Nevis"] = 'Saint Kitts'

a <- map[map$location == "Saint Kitts",]

a$location <- 'Nevis'

map <- rbind(map,a)

map$location[map$location == "Côte d'Ivoire"] = 'Ivory Coast'

map$location[map$location == "Saint Vincent and the Grenadines"] = 'Saint Vincent'

a <- map[map$location == "Saint Vincent",]

a$location <- 'Grenadines'

map <- rbind(map,a)

map$location[map$location == "Eswatini"] = 'Swaziland'

map$location[map$location == "Brunei Darussalam"] = 'Brunei'

map <- full_join(world,map,by = c('region'='location')) %>%

filter(val != "NA")

mycolor2<-brewer.pal(5, "Blues")

fig <- map %>%

ggplot()+

geom_polygon(aes(x = long, y = lat,group = group,fill=val2),colour="white",

size=0.2) +

theme_void()+

scale_fill_manual(values=mycolor2) +

theme(legend.position = c(0.1,0.2),

legend.title = element_blank(),

legend.text = element_text(color="black",

size = 12,

),

plot.title = element_blank(),

panel.grid=element_blank(),

axis.title.x = element_blank(),

axis.text.x = element_blank(),

axis.ticks.x = element_blank(),

axis.title.y = element_blank(),

axis.text.y = element_blank(),

axis.ticks.y = element_blank(),

)

fig

IS1 <- IS %>%

filter(measure == "Deaths") %>%

filter(year == 2021) %>%

filter(sex == "Both") %>%

filter(age == "Age-standardized") %>%

filter(metric == "Rate")

summary(IS1$val)

quantile(IS1$val,seq(0.1,1,0.1))

map <- IS1%>% mutate(val2 = cut(val, breaks = c(0,1,2,3,4,10000000),

labels = c("<1","1-2","2-3","3-4",">4"),

include.lowest = T,right = T))

map$location[map$location == 'United States of America'] = 'USA'

map$location[map$location == 'Russian Federation'] = 'Russia'

map$location[map$location == 'United Kingdom'] = 'UK'

map$location[map$location == 'Congo'] = 'Republic of Congo'

map$location[map$location == "Iran (Islamic Republic of)"] = 'Iran'

map$location[map$location == "Democratic People's Republic of Korea"] = 'North Korea'

map$location[map$location == "Taiwan (Province of China)"] = 'Taiwan'

map$location[map$location == "Republic of Korea"] = 'South Korea'

map$location[map$location == "United Republic of Tanzania"] = 'Tanzania'

map$location[map$location == "Bolivia (Plurinational State of)"] = 'Bolivia'

map$location[map$location == "Venezuela (Bolivarian Republic of)"] = 'Venezuela'

map$location[map$location == "Czechia"] = 'Czech Republic'

map$location[map$location == "Republic of Moldova"] = 'Moldova'

map$location[map$location == "Viet Nam"] = 'Vietnam'

map$location[map$location == "Lao People's Democratic Republic"] = 'Laos'

map$location[map$location == "Syrian Arab Republic"] = 'Syria'

map$location[map$location == "North Macedonia"] = 'Macedonia'

map$location[map$location == "Micronesia (Federated States of)"] = 'Micronesia'

map$location[map$location == "Macedonia"] = 'North Macedonia'

map$location[map$location == "Trinidad and Tobago"] = 'Trinidad'

a <- map[map$location == "Trinidad",]

a$location <- 'Tobago'

map <- rbind(map,a)

map$location[map$location == "Cabo Verde"] = 'Cape Verde'

map$location[map$location == "United States Virgin Islands"] = 'Virgin Islands'

map$location[map$location == "Antigua and Barbuda"] = 'Antigu'

a <- map[map$location == "Antigu",]

a$location <- 'Barbuda'

map <- rbind(map,a)

map$location[map$location == "Saint Kitts and Nevis"] = 'Saint Kitts'

a <- map[map$location == "Saint Kitts",]

a$location <- 'Nevis'

map <- rbind(map,a)

map$location[map$location == "Côte d'Ivoire"] = 'Ivory Coast'

map$location[map$location == "Saint Vincent and the Grenadines"] = 'Saint Vincent'

a <- map[map$location == "Saint Vincent",]

a$location <- 'Grenadines'

map <- rbind(map,a)

map$location[map$location == "Eswatini"] = 'Swaziland'

map$location[map$location == "Brunei Darussalam"] = 'Brunei'

map <- full_join(world,map,by = c('region'='location')) %>%

filter(val != "NA")

mycolor2<-brewer.pal(5, "Blues")

fig <- map %>%

ggplot()+

geom_polygon(aes(x = long, y = lat,group = group,fill=val2),colour="white",

size=0.2) +

theme_void()+

scale_fill_manual(values=mycolor2) +

theme(legend.position = c(0.1,0.2),

legend.title = element_blank(),

legend.text = element_text(color="black",

size = 12,

),

plot.title = element_blank(),

panel.grid=element_blank(),

axis.title.x = element_blank(),

axis.text.x = element_blank(),

axis.ticks.x = element_blank(),

axis.title.y = element_blank(),

axis.text.y = element_blank(),

axis.ticks.y = element_blank(),

)

fig

IS1 <- IS %>%

filter(measure == "DALYs") %>%

filter(year == 2021) %>%

filter(sex == "Both") %>%

filter(age == "Age-standardized") %>%

filter(metric == "Rate")

summary(IS1$val)

quantile(IS1$val,seq(0.1,1,0.1))

map <- IS1%>% mutate(val2 = cut(val, breaks = c(0,1,2,3,4,10000000),

labels = c("<1","1-2","2-3","3-4",">4"),

include.lowest = T,right = T))

map$location[map$location == 'United States of America'] = 'USA'

map$location[map$location == 'Russian Federation'] = 'Russia'

map$location[map$location == 'United Kingdom'] = 'UK'

map$location[map$location == 'Congo'] = 'Republic of Congo'

map$location[map$location == "Iran (Islamic Republic of)"] = 'Iran'

map$location[map$location == "Democratic People's Republic of Korea"] = 'North Korea'

map$location[map$location == "Taiwan (Province of China)"] = 'Taiwan'

map$location[map$location == "Republic of Korea"] = 'South Korea'

map$location[map$location == "United Republic of Tanzania"] = 'Tanzania'

map$location[map$location == "Bolivia (Plurinational State of)"] = 'Bolivia'

map$location[map$location == "Venezuela (Bolivarian Republic of)"] = 'Venezuela'

map$location[map$location == "Czechia"] = 'Czech Republic'

map$location[map$location == "Republic of Moldova"] = 'Moldova'

map$location[map$location == "Viet Nam"] = 'Vietnam'

map$location[map$location == "Lao People's Democratic Republic"] = 'Laos'

map$location[map$location == "Syrian Arab Republic"] = 'Syria'

map$location[map$location == "North Macedonia"] = 'Macedonia'

map$location[map$location == "Micronesia (Federated States of)"] = 'Micronesia'

map$location[map$location == "Macedonia"] = 'North Macedonia'

map$location[map$location == "Trinidad and Tobago"] = 'Trinidad'

a <- map[map$location == "Trinidad",]

a$location <- 'Tobago'

map <- rbind(map,a)

map$location[map$location == "Cabo Verde"] = 'Cape Verde'

map$location[map$location == "United States Virgin Islands"] = 'Virgin Islands'

map$location[map$location == "Antigua and Barbuda"] = 'Antigu'

a <- map[map$location == "Antigu",]

a$location <- 'Barbuda'

map <- rbind(map,a)

map$location[map$location == "Saint Kitts and Nevis"] = 'Saint Kitts'

a <- map[map$location == "Saint Kitts",]

a$location <- 'Nevis'

map <- rbind(map,a)

map$location[map$location == "Côte d'Ivoire"] = 'Ivory Coast'

map$location[map$location == "Saint Vincent and the Grenadines"] = 'Saint Vincent'

a <- map[map$location == "Saint Vincent",]

a$location <- 'Grenadines'

map <- rbind(map,a)

map$location[map$location == "Eswatini"] = 'Swaziland'

map$location[map$location == "Brunei Darussalam"] = 'Brunei'

map <- full_join(world,map,by = c('region'='location')) %>%

filter(val != "NA")

mycolor2<-brewer.pal(5, "Blues")

fig <- map %>%

ggplot()+

geom_polygon(aes(x = long, y = lat,group = group,fill=val2),colour="white",

size=0.2) +

theme_void()+

scale_fill_manual(values=mycolor2) +

theme(legend.position = c(0.1,0.2),

legend.title = element_blank(),

legend.text = element_text(color="black",

size = 12,

),

plot.title = element_blank(),

panel.grid=element_blank(),

axis.title.x = element_blank(),

axis.text.x = element_blank(),

axis.ticks.x = element_blank(),

axis.title.y = element_blank(),

axis.text.y = element_blank(),

axis.ticks.y = element_blank(),

)

fig

IS1 <- IS %>%

filter(measure == "Incidence") %>%

filter(year == 2021) %>%

filter(sex == "Both") %>%

filter(age == "All ages") %>%

filter(metric == "Number")

summary(IS1$val)

quantile(IS1$val,seq(0.1,1,0.1))

map <- IS1%>% mutate(val2 = cut(val, breaks = c(0,1,2,3,4,10000000),

labels = c("<1","1-2","2-3","3-4",">4"),

include.lowest = T,right = T))

map$location[map$location == 'United States of America'] = 'USA'

map$location[map$location == 'Russian Federation'] = 'Russia'

map$location[map$location == 'United Kingdom'] = 'UK'

map$location[map$location == 'Congo'] = 'Republic of Congo'

map$location[map$location == "Iran (Islamic Republic of)"] = 'Iran'

map$location[map$location == "Democratic People's Republic of Korea"] = 'North Korea'

map$location[map$location == "Taiwan (Province of China)"] = 'Taiwan'

map$location[map$location == "Republic of Korea"] = 'South Korea'

map$location[map$location == "United Republic of Tanzania"] = 'Tanzania'

map$location[map$location == "Bolivia (Plurinational State of)"] = 'Bolivia'

map$location[map$location == "Venezuela (Bolivarian Republic of)"] = 'Venezuela'

map$location[map$location == "Czechia"] = 'Czech Republic'

map$location[map$location == "Republic of Moldova"] = 'Moldova'

map$location[map$location == "Viet Nam"] = 'Vietnam'

map$location[map$location == "Lao People's Democratic Republic"] = 'Laos'

map$location[map$location == "Syrian Arab Republic"] = 'Syria'

map$location[map$location == "North Macedonia"] = 'Macedonia'

map$location[map$location == "Micronesia (Federated States of)"] = 'Micronesia'

map$location[map$location == "Macedonia"] = 'North Macedonia'

map$location[map$location == "Trinidad and Tobago"] = 'Trinidad'

a <- map[map$location == "Trinidad",]

a$location <- 'Tobago'

map <- rbind(map,a)

map$location[map$location == "Cabo Verde"] = 'Cape Verde'

map$location[map$location == "United States Virgin Islands"] = 'Virgin Islands'

map$location[map$location == "Antigua and Barbuda"] = 'Antigu'

a <- map[map$location == "Antigu",]

a$location <- 'Barbuda'

map <- rbind(map,a)

map$location[map$location == "Saint Kitts and Nevis"] = 'Saint Kitts'

a <- map[map$location == "Saint Kitts",]

a$location <- 'Nevis'

map <- rbind(map,a)

map$location[map$location == "Côte d'Ivoire"] = 'Ivory Coast'

map$location[map$location == "Saint Vincent and the Grenadines"] = 'Saint Vincent'

a <- map[map$location == "Saint Vincent",]

a$location <- 'Grenadines'

map <- rbind(map,a)

map$location[map$location == "Eswatini"] = 'Swaziland'

map$location[map$location == "Brunei Darussalam"] = 'Brunei'

map <- full_join(world,map,by = c('region'='location')) %>%

filter(val != "NA")

mycolor2<-brewer.pal(5, "Blues")

fig <- map %>%

ggplot()+

geom_polygon(aes(x = long, y = lat,group = group,fill=val2),colour="white",

size=0.2) +

theme_void()+

scale_fill_manual(values=mycolor2) +

theme(legend.position = c(0.1,0.2),

legend.title = element_blank(),

legend.text = element_text(color="black",

size = 12,

),

plot.title = element_blank(),

panel.grid=element_blank(),

axis.title.x = element_blank(),

axis.text.x = element_blank(),

axis.ticks.x = element_blank(),

axis.title.y = element_blank(),

axis.text.y = element_blank(),

axis.ticks.y = element_blank(),

)

fig

IS1 <- IS %>%

filter(measure == "Prevalence") %>%

filter(year == 2021) %>%

filter(sex == "Both") %>%

filter(age == "All ages") %>%

filter(metric == "Number")

summary(IS1$val)

quantile(IS1$val,seq(0.1,1,0.1))

map <- IS1%>% mutate(val2 = cut(val, breaks = c(0,1,2,3,4,10000000),

labels = c("<1","1-2","2-3","3-4",">4"),

include.lowest = T,right = T))

map$location[map$location == 'United States of America'] = 'USA'

map$location[map$location == 'Russian Federation'] = 'Russia'

map$location[map$location == 'United Kingdom'] = 'UK'

map$location[map$location == 'Congo'] = 'Republic of Congo'

map$location[map$location == "Iran (Islamic Republic of)"] = 'Iran'

map$location[map$location == "Democratic People's Republic of Korea"] = 'North Korea'

map$location[map$location == "Taiwan (Province of China)"] = 'Taiwan'

map$location[map$location == "Republic of Korea"] = 'South Korea'

map$location[map$location == "United Republic of Tanzania"] = 'Tanzania'

map$location[map$location == "Bolivia (Plurinational State of)"] = 'Bolivia'

map$location[map$location == "Venezuela (Bolivarian Republic of)"] = 'Venezuela'

map$location[map$location == "Czechia"] = 'Czech Republic'

map$location[map$location == "Republic of Moldova"] = 'Moldova'

map$location[map$location == "Viet Nam"] = 'Vietnam'

map$location[map$location == "Lao People's Democratic Republic"] = 'Laos'

map$location[map$location == "Syrian Arab Republic"] = 'Syria'

map$location[map$location == "North Macedonia"] = 'Macedonia'

map$location[map$location == "Micronesia (Federated States of)"] = 'Micronesia'

map$location[map$location == "Macedonia"] = 'North Macedonia'

map$location[map$location == "Trinidad and Tobago"] = 'Trinidad'

a <- map[map$location == "Trinidad",]

a$location <- 'Tobago'

map <- rbind(map,a)

map$location[map$location == "Cabo Verde"] = 'Cape Verde'

map$location[map$location == "United States Virgin Islands"] = 'Virgin Islands'

map$location[map$location == "Antigua and Barbuda"] = 'Antigu'

a <- map[map$location == "Antigu",]

a$location <- 'Barbuda'

map <- rbind(map,a)

map$location[map$location == "Saint Kitts and Nevis"] = 'Saint Kitts'

a <- map[map$location == "Saint Kitts",]

a$location <- 'Nevis'

map <- rbind(map,a)

map$location[map$location == "Côte d'Ivoire"] = 'Ivory Coast'

map$location[map$location == "Saint Vincent and the Grenadines"] = 'Saint Vincent'

a <- map[map$location == "Saint Vincent",]

a$location <- 'Grenadines'

map <- rbind(map,a)

map$location[map$location == "Eswatini"] = 'Swaziland'

map$location[map$location == "Brunei Darussalam"] = 'Brunei'

map <- full_join(world,map,by = c('region'='location')) %>%

filter(val != "NA")

mycolor2<-brewer.pal(5, "Blues")

fig <- map %>%

ggplot()+

geom_polygon(aes(x = long, y = lat,group = group,fill=val2),colour="white",

size=0.2) +

theme_void()+

scale_fill_manual(values=mycolor2) +

theme(legend.position = c(0.1,0.2),

legend.title = element_blank(),

legend.text = element_text(color="black",

size = 12,

),

plot.title = element_blank(),

panel.grid=element_blank(),

axis.title.x = element_blank(),

axis.text.x = element_blank(),

axis.ticks.x = element_blank(),

axis.title.y = element_blank(),

axis.text.y = element_blank(),

axis.ticks.y = element_blank(),

)

fig

IS1 <- IS %>%

filter(measure == "Deaths") %>%

filter(year == 2021) %>%

filter(sex == "Both") %>%

filter(age == "All ages") %>%

filter(metric == "Number")

summary(IS1$val)

quantile(IS1$val,seq(0.1,1,0.1))

map <- IS1%>% mutate(val2 = cut(val, breaks = c(0,1,2,3,4,10000000),

labels = c("<1","1-2","2-3","3-4",">4"),

include.lowest = T,right = T))

map$location[map$location == 'United States of America'] = 'USA'

map$location[map$location == 'Russian Federation'] = 'Russia'

map$location[map$location == 'United Kingdom'] = 'UK'

map$location[map$location == 'Congo'] = 'Republic of Congo'

map$location[map$location == "Iran (Islamic Republic of)"] = 'Iran'

map$location[map$location == "Democratic People's Republic of Korea"] = 'North Korea'

map$location[map$location == "Taiwan (Province of China)"] = 'Taiwan'

map$location[map$location == "Republic of Korea"] = 'South Korea'

map$location[map$location == "United Republic of Tanzania"] = 'Tanzania'

map$location[map$location == "Bolivia (Plurinational State of)"] = 'Bolivia'

map$location[map$location == "Venezuela (Bolivarian Republic of)"] = 'Venezuela'

map$location[map$location == "Czechia"] = 'Czech Republic'

map$location[map$location == "Republic of Moldova"] = 'Moldova'

map$location[map$location == "Viet Nam"] = 'Vietnam'

map$location[map$location == "Lao People's Democratic Republic"] = 'Laos'

map$location[map$location == "Syrian Arab Republic"] = 'Syria'

map$location[map$location == "North Macedonia"] = 'Macedonia'

map$location[map$location == "Micronesia (Federated States of)"] = 'Micronesia'

map$location[map$location == "Macedonia"] = 'North Macedonia'

map$location[map$location == "Trinidad and Tobago"] = 'Trinidad'

a <- map[map$location == "Trinidad",]

a$location <- 'Tobago'

map <- rbind(map,a)

map$location[map$location == "Cabo Verde"] = 'Cape Verde'

map$location[map$location == "United States Virgin Islands"] = 'Virgin Islands'

map$location[map$location == "Antigua and Barbuda"] = 'Antigu'

a <- map[map$location == "Antigu",]

a$location <- 'Barbuda'

map <- rbind(map,a)

map$location[map$location == "Saint Kitts and Nevis"] = 'Saint Kitts'

a <- map[map$location == "Saint Kitts",]

a$location <- 'Nevis'

map <- rbind(map,a)

map$location[map$location == "Côte d'Ivoire"] = 'Ivory Coast'

map$location[map$location == "Saint Vincent and the Grenadines"] = 'Saint Vincent'

a <- map[map$location == "Saint Vincent",]

a$location <- 'Grenadines'

map <- rbind(map,a)

map$location[map$location == "Eswatini"] = 'Swaziland'

map$location[map$location == "Brunei Darussalam"] = 'Brunei'

map <- full_join(world,map,by = c('region'='location')) %>%

filter(val != "NA")

mycolor2<-brewer.pal(5, "Blues")

fig <- map %>%

ggplot()+

geom_polygon(aes(x = long, y = lat,group = group,fill=val2),colour="white",

size=0.2) +

theme_void()+

scale_fill_manual(values=mycolor2) +

theme(legend.position = c(0.1,0.2),

legend.title = element_blank(),

legend.text = element_text(color="black",

size = 12,

),

plot.title = element_blank(),

panel.grid=element_blank(),

axis.title.x = element_blank(),

axis.text.x = element_blank(),

axis.ticks.x = element_blank(),

axis.title.y = element_blank(),

axis.text.y = element_blank(),

axis.ticks.y = element_blank(),

)

fig

IS1 <- IS %>%

filter(measure == "DALYs") %>%

filter(year == 2021) %>%

filter(sex == "Both") %>%

filter(age == "All ages") %>%

filter(metric == "Number")

summary(IS1$val)

quantile(IS1$val,seq(0.1,1,0.1))

map <- IS1%>% mutate(val2 = cut(val, breaks = c(0,1,2,3,4,10000000),

labels = c("<1","1-2","2-3","3-4",">4"),

include.lowest = T,right = T))

map$location[map$location == 'United States of America'] = 'USA'

map$location[map$location == 'Russian Federation'] = 'Russia'

map$location[map$location == 'United Kingdom'] = 'UK'

map$location[map$location == 'Congo'] = 'Republic of Congo'

map$location[map$location == "Iran (Islamic Republic of)"] = 'Iran'

map$location[map$location == "Democratic People's Republic of Korea"] = 'North Korea'

map$location[map$location == "Taiwan (Province of China)"] = 'Taiwan'

map$location[map$location == "Republic of Korea"] = 'South Korea'

map$location[map$location == "United Republic of Tanzania"] = 'Tanzania'

map$location[map$location == "Bolivia (Plurinational State of)"] = 'Bolivia'

map$location[map$location == "Venezuela (Bolivarian Republic of)"] = 'Venezuela'

map$location[map$location == "Czechia"] = 'Czech Republic'

map$location[map$location == "Republic of Moldova"] = 'Moldova'

map$location[map$location == "Viet Nam"] = 'Vietnam'

map$location[map$location == "Lao People's Democratic Republic"] = 'Laos'

map$location[map$location == "Syrian Arab Republic"] = 'Syria'

map$location[map$location == "North Macedonia"] = 'Macedonia'

map$location[map$location == "Micronesia (Federated States of)"] = 'Micronesia'

map$location[map$location == "Macedonia"] = 'North Macedonia'

map$location[map$location == "Trinidad and Tobago"] = 'Trinidad'

a <- map[map$location == "Trinidad",]

a$location <- 'Tobago'

map <- rbind(map,a)

map$location[map$location == "Cabo Verde"] = 'Cape Verde'

map$location[map$location == "United States Virgin Islands"] = 'Virgin Islands'

map$location[map$location == "Antigua and Barbuda"] = 'Antigu'

a <- map[map$location == "Antigu",]

a$location <- 'Barbuda'

map <- rbind(map,a)

map$location[map$location == "Saint Kitts and Nevis"] = 'Saint Kitts'

a <- map[map$location == "Saint Kitts",]

a$location <- 'Nevis'

map <- rbind(map,a)

map$location[map$location == "Côte d'Ivoire"] = 'Ivory Coast'

map$location[map$location == "Saint Vincent and the Grenadines"] = 'Saint Vincent'

a <- map[map$location == "Saint Vincent",]

a$location <- 'Grenadines'

map <- rbind(map,a)

map$location[map$location == "Eswatini"] = 'Swaziland'

map$location[map$location == "Brunei Darussalam"] = 'Brunei'

map <- full_join(world,map,by = c('region'='location')) %>%

filter(val != "NA")

mycolor2<-brewer.pal(5, "Blues")

fig <- map %>%

ggplot()+

geom_polygon(aes(x = long, y = lat,group = group,fill=val2),colour="white",

size=0.2) +

theme_void()+

scale_fill_manual(values=mycolor2) +

theme(legend.position = c(0.1,0.2),

legend.title = element_blank(),

legend.text = element_text(color="black",

size = 12,

),

plot.title = element_blank(),

panel.grid=element_blank(),

axis.title.x = element_blank(),

axis.text.x = element_blank(),

axis.ticks.x = element_blank(),

axis.title.y = element_blank(),

axis.text.y = element_blank(),

axis.ticks.y = element_blank(),

)

fig

world <- map_data('world')

ALL_country<- vroom::vroom("ALL_country.csv")

IS <- ALL_country %>%

dplyr::select(measure_name,location_name,metric_name,

sex_name,age_name,year,val,lower,upper) %>%

rename(measure=measure_name,

location=location_name,

metric=metric_name,

sex=sex_name,

age=age_name)

IS1 <- IS %>%

filter(measure == "Incidence") %>%

filter(year == 2021) %>%

filter(sex == "Both") %>%

filter(age == "Age-standardized") %>%

filter(metric == "Rate")

summary(IS1$val)

quantile(IS1$val,seq(0.1,1,0.1))

map <- IS1%>% mutate(val2 = cut(val, breaks = c(0,1,2,3,4,10000000),

labels = c("<1","1-2","2-3","3-4",">4"),

include.lowest = T,right = T))

map$location[map$location == 'United States of America'] = 'USA'

map$location[map$location == 'Russian Federation'] = 'Russia'

map$location[map$location == 'United Kingdom'] = 'UK'

map$location[map$location == 'Congo'] = 'Republic of Congo'

map$location[map$location == "Iran (Islamic Republic of)"] = 'Iran'

map$location[map$location == "Democratic People's Republic of Korea"] = 'North Korea'

map$location[map$location == "Taiwan (Province of China)"] = 'Taiwan'

map$location[map$location == "Republic of Korea"] = 'South Korea'

map$location[map$location == "United Republic of Tanzania"] = 'Tanzania'

map$location[map$location == "Bolivia (Plurinational State of)"] = 'Bolivia'

map$location[map$location == "Venezuela (Bolivarian Republic of)"] = 'Venezuela'

map$location[map$location == "Czechia"] = 'Czech Republic'

map$location[map$location == "Republic of Moldova"] = 'Moldova'

map$location[map$location == "Viet Nam"] = 'Vietnam'

map$location[map$location == "Lao People's Democratic Republic"] = 'Laos'

map$location[map$location == "Syrian Arab Republic"] = 'Syria'

map$location[map$location == "North Macedonia"] = 'Macedonia'

map$location[map$location == "Micronesia (Federated States of)"] = 'Micronesia'

map$location[map$location == "Macedonia"] = 'North Macedonia'

map$location[map$location == "Trinidad and Tobago"] = 'Trinidad'

a <- map[map$location == "Trinidad",]

a$location <- 'Tobago'

map <- rbind(map,a)

map$location[map$location == "Cabo Verde"] = 'Cape Verde'

map$location[map$location == "United States Virgin Islands"] = 'Virgin Islands'

map$location[map$location == "Antigua and Barbuda"] = 'Antigu'

a <- map[map$location == "Antigu",]

a$location <- 'Barbuda'

map <- rbind(map,a)

map$location[map$location == "Saint Kitts and Nevis"] = 'Saint Kitts'

a <- map[map$location == "Saint Kitts",]

a$location <- 'Nevis'

map <- rbind(map,a)

map$location[map$location == "Côte d'Ivoire"] = 'Ivory Coast'

map$location[map$location == "Saint Vincent and the Grenadines"] = 'Saint Vincent'

a <- map[map$location == "Saint Vincent",]

a$location <- 'Grenadines'

map <- rbind(map,a)

map$location[map$location == "Eswatini"] = 'Swaziland'

map$location[map$location == "Brunei Darussalam"] = 'Brunei'

map <- full_join(world,map,by = c('region'='location')) %>%

filter(val != "NA")

mycolor2<-brewer.pal(5, "Blues")

fig <- map %>%

ggplot()+

geom_polygon(aes(x = long, y = lat,group = group,fill=val2),colour="white",

size=0.2) +

theme_void()+

scale_fill_manual(values=mycolor2) +

theme(legend.position = c(0.1,0.2),

legend.title = element_blank(),

legend.text = element_text(color="black",

size = 12,

),

plot.title = element_blank(),

panel.grid=element_blank(),

axis.title.x = element_blank(),

axis.text.x = element_blank(),

axis.ticks.x = element_blank(),

axis.title.y = element_blank(),

axis.text.y = element_blank(),

axis.ticks.y = element_blank(),

)

fig

IS1 <- IS %>%

filter(measure == "Prevalence") %>%

filter(year == 2021) %>%

filter(sex == "Both") %>%

filter(age == "Age-standardized") %>%

filter(metric == "Rate")

summary(IS1$val)

quantile(IS1$val,seq(0.1,1,0.1))

map <- IS1%>% mutate(val2 = cut(val, breaks = c(0,1,2,3,4,10000000),

labels = c("<1","1-2","2-3","3-4",">4"),

include.lowest = T,right = T))

map$location[map$location == 'United States of America'] = 'USA'

map$location[map$location == 'Russian Federation'] = 'Russia'

map$location[map$location == 'United Kingdom'] = 'UK'

map$location[map$location == 'Congo'] = 'Republic of Congo'

map$location[map$location == "Iran (Islamic Republic of)"] = 'Iran'

map$location[map$location == "Democratic People's Republic of Korea"] = 'North Korea'

map$location[map$location == "Taiwan (Province of China)"] = 'Taiwan'

map$location[map$location == "Republic of Korea"] = 'South Korea'

map$location[map$location == "United Republic of Tanzania"] = 'Tanzania'

map$location[map$location == "Bolivia (Plurinational State of)"] = 'Bolivia'

map$location[map$location == "Venezuela (Bolivarian Republic of)"] = 'Venezuela'

map$location[map$location == "Czechia"] = 'Czech Republic'

map$location[map$location == "Republic of Moldova"] = 'Moldova'

map$location[map$location == "Viet Nam"] = 'Vietnam'

map$location[map$location == "Lao People's Democratic Republic"] = 'Laos'

map$location[map$location == "Syrian Arab Republic"] = 'Syria'

map$location[map$location == "North Macedonia"] = 'Macedonia'

map$location[map$location == "Micronesia (Federated States of)"] = 'Micronesia'

map$location[map$location == "Macedonia"] = 'North Macedonia'

map$location[map$location == "Trinidad and Tobago"] = 'Trinidad'

a <- map[map$location == "Trinidad",]

a$location <- 'Tobago'

map <- rbind(map,a)

map$location[map$location == "Cabo Verde"] = 'Cape Verde'

map$location[map$location == "United States Virgin Islands"] = 'Virgin Islands'

map$location[map$location == "Antigua and Barbuda"] = 'Antigu'

a <- map[map$location == "Antigu",]

a$location <- 'Barbuda'

map <- rbind(map,a)

map$location[map$location == "Saint Kitts and Nevis"] = 'Saint Kitts'

a <- map[map$location == "Saint Kitts",]

a$location <- 'Nevis'

map <- rbind(map,a)

map$location[map$location == "Côte d'Ivoire"] = 'Ivory Coast'

map$location[map$location == "Saint Vincent and the Grenadines"] = 'Saint Vincent'

a <- map[map$location == "Saint Vincent",]

a$location <- 'Grenadines'

map <- rbind(map,a)

map$location[map$location == "Eswatini"] = 'Swaziland'

map$location[map$location == "Brunei Darussalam"] = 'Brunei'

map <- full_join(world,map,by = c('region'='location')) %>%

filter(val != "NA")

mycolor2<-brewer.pal(5, "Blues")

fig <- map %>%

ggplot()+

geom_polygon(aes(x = long, y = lat,group = group,fill=val2),colour="white",

size=0.2) +

theme_void()+

scale_fill_manual(values=mycolor2) +

theme(legend.position = c(0.1,0.2),

legend.title = element_blank(),

legend.text = element_text(color="black",

size = 12,

),

plot.title = element_blank(),

panel.grid=element_blank(),

axis.title.x = element_blank(),

axis.text.x = element_blank(),

axis.ticks.x = element_blank(),

axis.title.y = element_blank(),

axis.text.y = element_blank(),

axis.ticks.y = element_blank(),

)

fig

IS1 <- IS %>%

filter(measure == "Deaths") %>%

filter(year == 2021) %>%

filter(sex == "Both") %>%

filter(age == "Age-standardized") %>%

filter(metric == "Rate")

summary(IS1$val)

quantile(IS1$val,seq(0.1,1,0.1))

map <- IS1%>% mutate(val2 = cut(val, breaks = c(0,1,2,3,4,10000000),

labels = c("<1","1-2","2-3","3-4",">4"),

include.lowest = T,right = T))

map$location[map$location == 'United States of America'] = 'USA'

map$location[map$location == 'Russian Federation'] = 'Russia'

map$location[map$location == 'United Kingdom'] = 'UK'

map$location[map$location == 'Congo'] = 'Republic of Congo'

map$location[map$location == "Iran (Islamic Republic of)"] = 'Iran'

map$location[map$location == "Democratic People's Republic of Korea"] = 'North Korea'

map$location[map$location == "Taiwan (Province of China)"] = 'Taiwan'

map$location[map$location == "Republic of Korea"] = 'South Korea'

map$location[map$location == "United Republic of Tanzania"] = 'Tanzania'

map$location[map$location == "Bolivia (Plurinational State of)"] = 'Bolivia'

map$location[map$location == "Venezuela (Bolivarian Republic of)"] = 'Venezuela'

map$location[map$location == "Czechia"] = 'Czech Republic'

map$location[map$location == "Republic of Moldova"] = 'Moldova'

map$location[map$location == "Viet Nam"] = 'Vietnam'

map$location[map$location == "Lao People's Democratic Republic"] = 'Laos'

map$location[map$location == "Syrian Arab Republic"] = 'Syria'

map$location[map$location == "North Macedonia"] = 'Macedonia'

map$location[map$location == "Micronesia (Federated States of)"] = 'Micronesia'

map$location[map$location == "Macedonia"] = 'North Macedonia'

map$location[map$location == "Trinidad and Tobago"] = 'Trinidad'

a <- map[map$location == "Trinidad",]

a$location <- 'Tobago'

map <- rbind(map,a)

map$location[map$location == "Cabo Verde"] = 'Cape Verde'

map$location[map$location == "United States Virgin Islands"] = 'Virgin Islands'

map$location[map$location == "Antigua and Barbuda"] = 'Antigu'

a <- map[map$location == "Antigu",]

a$location <- 'Barbuda'

map <- rbind(map,a)

map$location[map$location == "Saint Kitts and Nevis"] = 'Saint Kitts'

a <- map[map$location == "Saint Kitts",]

a$location <- 'Nevis'

map <- rbind(map,a)

map$location[map$location == "Côte d'Ivoire"] = 'Ivory Coast'

map$location[map$location == "Saint Vincent and the Grenadines"] = 'Saint Vincent'

a <- map[map$location == "Saint Vincent",]

a$location <- 'Grenadines'

map <- rbind(map,a)

map$location[map$location == "Eswatini"] = 'Swaziland'

map$location[map$location == "Brunei Darussalam"] = 'Brunei'

map <- full_join(world,map,by = c('region'='location')) %>%

filter(val != "NA")

mycolor2<-brewer.pal(5, "Blues")

fig <- map %>%

ggplot()+

geom_polygon(aes(x = long, y = lat,group = group,fill=val2),colour="white",

size=0.2) +

theme_void()+

scale_fill_manual(values=mycolor2) +

theme(legend.position = c(0.1,0.2),

legend.title = element_blank(),

legend.text = element_text(color="black",

size = 12,

),

plot.title = element_blank(),

panel.grid=element_blank(),

axis.title.x = element_blank(),

axis.text.x = element_blank(),

axis.ticks.x = element_blank(),

axis.title.y = element_blank(),

axis.text.y = element_blank(),

axis.ticks.y = element_blank(),

)

fig

IS1 <- IS %>%

filter(measure == "DALYs") %>%

filter(year == 2021) %>%

filter(sex == "Both") %>%

filter(age == "Age-standardized") %>%

filter(metric == "Rate")

summary(IS1$val)

quantile(IS1$val,seq(0.1,1,0.1))

map <- IS1%>% mutate(val2 = cut(val, breaks = c(0,1,2,3,4,10000000),

labels = c("<1","1-2","2-3","3-4",">4"),

include.lowest = T,right = T))

map$location[map$location == 'United States of America'] = 'USA'

map$location[map$location == 'Russian Federation'] = 'Russia'

map$location[map$location == 'United Kingdom'] = 'UK'

map$location[map$location == 'Congo'] = 'Republic of Congo'

map$location[map$location == "Iran (Islamic Republic of)"] = 'Iran'

map$location[map$location == "Democratic People's Republic of Korea"] = 'North Korea'

map$location[map$location == "Taiwan (Province of China)"] = 'Taiwan'

map$location[map$location == "Republic of Korea"] = 'South Korea'

map$location[map$location == "United Republic of Tanzania"] = 'Tanzania'

map$location[map$location == "Bolivia (Plurinational State of)"] = 'Bolivia'

map$location[map$location == "Venezuela (Bolivarian Republic of)"] = 'Venezuela'

map$location[map$location == "Czechia"] = 'Czech Republic'

map$location[map$location == "Republic of Moldova"] = 'Moldova'

map$location[map$location == "Viet Nam"] = 'Vietnam'

map$location[map$location == "Lao People's Democratic Republic"] = 'Laos'

map$location[map$location == "Syrian Arab Republic"] = 'Syria'

map$location[map$location == "North Macedonia"] = 'Macedonia'

map$location[map$location == "Micronesia (Federated States of)"] = 'Micronesia'

map$location[map$location == "Macedonia"] = 'North Macedonia'

map$location[map$location == "Trinidad and Tobago"] = 'Trinidad'

a <- map[map$location == "Trinidad",]

a$location <- 'Tobago'

map <- rbind(map,a)

map$location[map$location == "Cabo Verde"] = 'Cape Verde'

map$location[map$location == "United States Virgin Islands"] = 'Virgin Islands'

map$location[map$location == "Antigua and Barbuda"] = 'Antigu'

a <- map[map$location == "Antigu",]

a$location <- 'Barbuda'

map <- rbind(map,a)

map$location[map$location == "Saint Kitts and Nevis"] = 'Saint Kitts'

a <- map[map$location == "Saint Kitts",]

a$location <- 'Nevis'

map <- rbind(map,a)

map$location[map$location == "Côte d'Ivoire"] = 'Ivory Coast'

map$location[map$location == "Saint Vincent and the Grenadines"] = 'Saint Vincent'

a <- map[map$location == "Saint Vincent",]

a$location <- 'Grenadines'

map <- rbind(map,a)

map$location[map$location == "Eswatini"] = 'Swaziland'

map$location[map$location == "Brunei Darussalam"] = 'Brunei'

map <- full_join(world,map,by = c('region'='location')) %>%

filter(val != "NA")

mycolor2<-brewer.pal(5, "Blues")

fig <- map %>%

ggplot()+

geom_polygon(aes(x = long, y = lat,group = group,fill=val2),colour="white",

size=0.2) +

theme_void()+

scale_fill_manual(values=mycolor2) +

theme(legend.position = c(0.1,0.2),

legend.title = element_blank(),

legend.text = element_text(color="black",

size = 12,

),

plot.title = element_blank(),

panel.grid=element_blank(),

axis.title.x = element_blank(),

axis.text.x = element_blank(),

axis.ticks.x = element_blank(),

axis.title.y = element_blank(),

axis.text.y = element_blank(),

axis.ticks.y = element_blank(),

)

fig

IS1 <- IS %>%

filter(measure == "Incidence") %>%

filter(year == 2021) %>%

filter(sex == "Both") %>%

filter(age == "All ages") %>%

filter(metric == "Number")

summary(IS1$val)

quantile(IS1$val,seq(0.1,1,0.1))

map <- IS1%>% mutate(val2 = cut(val, breaks = c(0,1,2,3,4,10000000),

labels = c("<1","1-2","2-3","3-4",">4"),

include.lowest = T,right = T))

map$location[map$location == 'United States of America'] = 'USA'

map$location[map$location == 'Russian Federation'] = 'Russia'

map$location[map$location == 'United Kingdom'] = 'UK'

map$location[map$location == 'Congo'] = 'Republic of Congo'

map$location[map$location == "Iran (Islamic Republic of)"] = 'Iran'

map$location[map$location == "Democratic People's Republic of Korea"] = 'North Korea'

map$location[map$location == "Taiwan (Province of China)"] = 'Taiwan'

map$location[map$location == "Republic of Korea"] = 'South Korea'

map$location[map$location == "United Republic of Tanzania"] = 'Tanzania'

map$location[map$location == "Bolivia (Plurinational State of)"] = 'Bolivia'

map$location[map$location == "Venezuela (Bolivarian Republic of)"] = 'Venezuela'

map$location[map$location == "Czechia"] = 'Czech Republic'

map$location[map$location == "Republic of Moldova"] = 'Moldova'

map$location[map$location == "Viet Nam"] = 'Vietnam'

map$location[map$location == "Lao People's Democratic Republic"] = 'Laos'

map$location[map$location == "Syrian Arab Republic"] = 'Syria'

map$location[map$location == "North Macedonia"] = 'Macedonia'

map$location[map$location == "Micronesia (Federated States of)"] = 'Micronesia'

map$location[map$location == "Macedonia"] = 'North Macedonia'

map$location[map$location == "Trinidad and Tobago"] = 'Trinidad'

a <- map[map$location == "Trinidad",]

a$location <- 'Tobago'

map <- rbind(map,a)

map$location[map$location == "Cabo Verde"] = 'Cape Verde'

map$location[map$location == "United States Virgin Islands"] = 'Virgin Islands'

map$location[map$location == "Antigua and Barbuda"] = 'Antigu'

a <- map[map$location == "Antigu",]

a$location <- 'Barbuda'

map <- rbind(map,a)

map$location[map$location == "Saint Kitts and Nevis"] = 'Saint Kitts'

a <- map[map$location == "Saint Kitts",]

a$location <- 'Nevis'

map <- rbind(map,a)

map$location[map$location == "Côte d'Ivoire"] = 'Ivory Coast'

map$location[map$location == "Saint Vincent and the Grenadines"] = 'Saint Vincent'

a <- map[map$location == "Saint Vincent",]

a$location <- 'Grenadines'

map <- rbind(map,a)

map$location[map$location == "Eswatini"] = 'Swaziland'

map$location[map$location == "Brunei Darussalam"] = 'Brunei'

map <- full_join(world,map,by = c('region'='location')) %>%

filter(val != "NA")

mycolor2<-brewer.pal(5, "Blues")

fig <- map %>%

ggplot()+

geom_polygon(aes(x = long, y = lat,group = group,fill=val2),colour="white",

size=0.2) +

theme_void()+

scale_fill_manual(values=mycolor2) +

theme(legend.position = c(0.1,0.2),

legend.title = element_blank(),

legend.text = element_text(color="black",

size = 12,

),

plot.title = element_blank(),

panel.grid=element_blank(),

axis.title.x = element_blank(),

axis.text.x = element_blank(),

axis.ticks.x = element_blank(),

axis.title.y = element_blank(),

axis.text.y = element_blank(),

axis.ticks.y = element_blank(),

)

fig

IS1 <- IS %>%

filter(measure == "Prevalence") %>%

filter(year == 2021) %>%

filter(sex == "Both") %>%

filter(age == "All ages") %>%

filter(metric == "Number")

summary(IS1$val)

quantile(IS1$val,seq(0.1,1,0.1))

map <- IS1%>% mutate(val2 = cut(val, breaks = c(0,1,2,3,4,10000000),

labels = c("<1","1-2","2-3","3-4",">4"),

include.lowest = T,right = T))

map$location[map$location == 'United States of America'] = 'USA'

map$location[map$location == 'Russian Federation'] = 'Russia'

map$location[map$location == 'United Kingdom'] = 'UK'

map$location[map$location == 'Congo'] = 'Republic of Congo'

map$location[map$location == "Iran (Islamic Republic of)"] = 'Iran'

map$location[map$location == "Democratic People's Republic of Korea"] = 'North Korea'

map$location[map$location == "Taiwan (Province of China)"] = 'Taiwan'

map$location[map$location == "Republic of Korea"] = 'South Korea'

map$location[map$location == "United Republic of Tanzania"] = 'Tanzania'

map$location[map$location == "Bolivia (Plurinational State of)"] = 'Bolivia'

map$location[map$location == "Venezuela (Bolivarian Republic of)"] = 'Venezuela'

map$location[map$location == "Czechia"] = 'Czech Republic'

map$location[map$location == "Republic of Moldova"] = 'Moldova'

map$location[map$location == "Viet Nam"] = 'Vietnam'

map$location[map$location == "Lao People's Democratic Republic"] = 'Laos'

map$location[map$location == "Syrian Arab Republic"] = 'Syria'

map$location[map$location == "North Macedonia"] = 'Macedonia'

map$location[map$location == "Micronesia (Federated States of)"] = 'Micronesia'

map$location[map$location == "Macedonia"] = 'North Macedonia'

map$location[map$location == "Trinidad and Tobago"] = 'Trinidad'

a <- map[map$location == "Trinidad",]

a$location <- 'Tobago'

map <- rbind(map,a)

map$location[map$location == "Cabo Verde"] = 'Cape Verde'

map$location[map$location == "United States Virgin Islands"] = 'Virgin Islands'

map$location[map$location == "Antigua and Barbuda"] = 'Antigu'

a <- map[map$location == "Antigu",]

a$location <- 'Barbuda'

map <- rbind(map,a)

map$location[map$location == "Saint Kitts and Nevis"] = 'Saint Kitts'

a <- map[map$location == "Saint Kitts",]

a$location <- 'Nevis'

map <- rbind(map,a)

map$location[map$location == "Côte d'Ivoire"] = 'Ivory Coast'

map$location[map$location == "Saint Vincent and the Grenadines"] = 'Saint Vincent'

a <- map[map$location == "Saint Vincent",]

a$location <- 'Grenadines'

map <- rbind(map,a)

map$location[map$location == "Eswatini"] = 'Swaziland'

map$location[map$location == "Brunei Darussalam"] = 'Brunei'

map <- full_join(world,map,by = c('region'='location')) %>%

filter(val != "NA")

mycolor2<-brewer.pal(5, "Blues")

fig <- map %>%

ggplot()+

geom_polygon(aes(x = long, y = lat,group = group,fill=val2),colour="white",

size=0.2) +

theme_void()+

scale_fill_manual(values=mycolor2) +

theme(legend.position = c(0.1,0.2),

legend.title = element_blank(),

legend.text = element_text(color="black",

size = 12,

),

plot.title = element_blank(),

panel.grid=element_blank(),

axis.title.x = element_blank(),

axis.text.x = element_blank(),

axis.ticks.x = element_blank(),

axis.title.y = element_blank(),

axis.text.y = element_blank(),

axis.ticks.y = element_blank(),

)

fig

IS1 <- IS %>%

filter(measure == "Deaths") %>%

filter(year == 2021) %>%

filter(sex == "Both") %>%

filter(age == "All ages") %>%

filter(metric == "Number")

summary(IS1$val)

quantile(IS1$val,seq(0.1,1,0.1))

map <- IS1%>% mutate(val2 = cut(val, breaks = c(0,1,2,3,4,10000000),

labels = c("<1","1-2","2-3","3-4",">4"),

include.lowest = T,right = T))

map$location[map$location == 'United States of America'] = 'USA'

map$location[map$location == 'Russian Federation'] = 'Russia'

map$location[map$location == 'United Kingdom'] = 'UK'

map$location[map$location == 'Congo'] = 'Republic of Congo'

map$location[map$location == "Iran (Islamic Republic of)"] = 'Iran'

map$location[map$location == "Democratic People's Republic of Korea"] = 'North Korea'

map$location[map$location == "Taiwan (Province of China)"] = 'Taiwan'

map$location[map$location == "Republic of Korea"] = 'South Korea'

map$location[map$location == "United Republic of Tanzania"] = 'Tanzania'

map$location[map$location == "Bolivia (Plurinational State of)"] = 'Bolivia'

map$location[map$location == "Venezuela (Bolivarian Republic of)"] = 'Venezuela'

map$location[map$location == "Czechia"] = 'Czech Republic'

map$location[map$location == "Republic of Moldova"] = 'Moldova'

map$location[map$location == "Viet Nam"] = 'Vietnam'

map$location[map$location == "Lao People's Democratic Republic"] = 'Laos'

map$location[map$location == "Syrian Arab Republic"] = 'Syria'

map$location[map$location == "North Macedonia"] = 'Macedonia'

map$location[map$location == "Micronesia (Federated States of)"] = 'Micronesia'

map$location[map$location == "Macedonia"] = 'North Macedonia'

map$location[map$location == "Trinidad and Tobago"] = 'Trinidad'

a <- map[map$location == "Trinidad",]

a$location <- 'Tobago'

map <- rbind(map,a)

map$location[map$location == "Cabo Verde"] = 'Cape Verde'

map$location[map$location == "United States Virgin Islands"] = 'Virgin Islands'

map$location[map$location == "Antigua and Barbuda"] = 'Antigu'

a <- map[map$location == "Antigu",]

a$location <- 'Barbuda'

map <- rbind(map,a)

map$location[map$location == "Saint Kitts and Nevis"] = 'Saint Kitts'

a <- map[map$location == "Saint Kitts",]

a$location <- 'Nevis'

map <- rbind(map,a)

map$location[map$location == "Côte d'Ivoire"] = 'Ivory Coast'

map$location[map$location == "Saint Vincent and the Grenadines"] = 'Saint Vincent'

a <- map[map$location == "Saint Vincent",]

a$location <- 'Grenadines'

map <- rbind(map,a)

map$location[map$location == "Eswatini"] = 'Swaziland'

map$location[map$location == "Brunei Darussalam"] = 'Brunei'

map <- full_join(world,map,by = c('region'='location')) %>%

filter(val != "NA")

mycolor2<-brewer.pal(5, "Blues")

fig <- map %>%

ggplot()+

geom_polygon(aes(x = long, y = lat,group = group,fill=val2),colour="white",

size=0.2) +

theme_void()+

scale_fill_manual(values=mycolor2) +

theme(legend.position = c(0.1,0.2),

legend.title = element_blank(),

legend.text = element_text(color="black",

size = 12,

),

plot.title = element_blank(),

panel.grid=element_blank(),

axis.title.x = element_blank(),

axis.text.x = element_blank(),

axis.ticks.x = element_blank(),

axis.title.y = element_blank(),

axis.text.y = element_blank(),

axis.ticks.y = element_blank(),

)

fig

IS1 <- IS %>%

filter(measure == "DALYs") %>%

filter(year == 2021) %>%

filter(sex == "Both") %>%

filter(age == "All ages") %>%

filter(metric == "Number")

summary(IS1$val)

quantile(IS1$val,seq(0.1,1,0.1))

map <- IS1%>% mutate(val2 = cut(val, breaks = c(0,1,2,3,4,10000000),

labels = c("<1","1-2","2-3","3-4",">4"),

include.lowest = T,right = T))

map$location[map$location == 'United States of America'] = 'USA'

map$location[map$location == 'Russian Federation'] = 'Russia'

map$location[map$location == 'United Kingdom'] = 'UK'

map$location[map$location == 'Congo'] = 'Republic of Congo'

map$location[map$location == "Iran (Islamic Republic of)"] = 'Iran'

map$location[map$location == "Democratic People's Republic of Korea"] = 'North Korea'

map$location[map$location == "Taiwan (Province of China)"] = 'Taiwan'

map$location[map$location == "Republic of Korea"] = 'South Korea'

map$location[map$location == "United Republic of Tanzania"] = 'Tanzania'

map$location[map$location == "Bolivia (Plurinational State of)"] = 'Bolivia'

map$location[map$location == "Venezuela (Bolivarian Republic of)"] = 'Venezuela'

map$location[map$location == "Czechia"] = 'Czech Republic'

map$location[map$location == "Republic of Moldova"] = 'Moldova'

map$location[map$location == "Viet Nam"] = 'Vietnam'

map$location[map$location == "Lao People's Democratic Republic"] = 'Laos'

map$location[map$location == "Syrian Arab Republic"] = 'Syria'

map$location[map$location == "North Macedonia"] = 'Macedonia'

map$location[map$location == "Micronesia (Federated States of)"] = 'Micronesia'

map$location[map$location == "Macedonia"] = 'North Macedonia'

map$location[map$location == "Trinidad and Tobago"] = 'Trinidad'

a <- map[map$location == "Trinidad",]

a$location <- 'Tobago'

map <- rbind(map,a)

map$location[map$location == "Cabo Verde"] = 'Cape Verde'

map$location[map$location == "United States Virgin Islands"] = 'Virgin Islands'

map$location[map$location == "Antigua and Barbuda"] = 'Antigu'

a <- map[map$location == "Antigu",]

a$location <- 'Barbuda'

map <- rbind(map,a)

map$location[map$location == "Saint Kitts and Nevis"] = 'Saint Kitts'

a <- map[map$location == "Saint Kitts",]

a$location <- 'Nevis'

map <- rbind(map,a)

map$location[map$location == "Côte d'Ivoire"] = 'Ivory Coast'

map$location[map$location == "Saint Vincent and the Grenadines"] = 'Saint Vincent'

a <- map[map$location == "Saint Vincent",]

a$location <- 'Grenadines'

map <- rbind(map,a)

map$location[map$location == "Eswatini"] = 'Swaziland'

map$location[map$location == "Brunei Darussalam"] = 'Brunei'

map <- full_join(world,map,by = c('region'='location')) %>%

filter(val != "NA")

mycolor2<-brewer.pal(5, "Blues")

fig <- map %>%

ggplot()+

geom_polygon(aes(x = long, y = lat,group = group,fill=val2),colour="white",

size=0.2) +

theme_void()+

scale_fill_manual(values=mycolor2) +

theme(legend.position = c(0.1,0.2),

legend.title = element_blank(),

legend.text = element_text(color="black",

size = 12,

),

plot.title = element_blank(),

panel.grid=element_blank(),

axis.title.x = element_blank(),

axis.text.x = element_blank(),

axis.ticks.x = element_blank(),

axis.title.y = element_blank(),

axis.text.y = element_blank(),

axis.ticks.y = element_blank(),

)

fig

world <- map_data('world')

CLL_country<- vroom::vroom("CLL_country.csv")

IS <- ALL_country %>%

dplyr::select(measure_name,location_name,metric_name,

sex_name,age_name,year,val,lower,upper) %>%

rename(measure=measure_name,

location=location_name,

metric=metric_name,

sex=sex_name,

age=age_name)

IS1 <- IS %>%

filter(measure == "Incidence") %>%

filter(year == 2021) %>%

filter(sex == "Both") %>%

filter(age == "Age-standardized") %>%

filter(metric == "Rate")

summary(IS1$val)

quantile(IS1$val,seq(0.1,1,0.1))

map <- IS1%>% mutate(val2 = cut(val, breaks = c(0,1,2,3,4,10000000),

labels = c("<1","1-2","2-3","3-4",">4"),

include.lowest = T,right = T))

map$location[map$location == 'United States of America'] = 'USA'

map$location[map$location == 'Russian Federation'] = 'Russia'

map$location[map$location == 'United Kingdom'] = 'UK'

map$location[map$location == 'Congo'] = 'Republic of Congo'

map$location[map$location == "Iran (Islamic Republic of)"] = 'Iran'

map$location[map$location == "Democratic People's Republic of Korea"] = 'North Korea'

map$location[map$location == "Taiwan (Province of China)"] = 'Taiwan'

map$location[map$location == "Republic of Korea"] = 'South Korea'

map$location[map$location == "United Republic of Tanzania"] = 'Tanzania'

map$location[map$location == "Bolivia (Plurinational State of)"] = 'Bolivia'

map$location[map$location == "Venezuela (Bolivarian Republic of)"] = 'Venezuela'

map$location[map$location == "Czechia"] = 'Czech Republic'

map$location[map$location == "Republic of Moldova"] = 'Moldova'

map$location[map$location == "Viet Nam"] = 'Vietnam'

map$location[map$location == "Lao People's Democratic Republic"] = 'Laos'

map$location[map$location == "Syrian Arab Republic"] = 'Syria'

map$location[map$location == "North Macedonia"] = 'Macedonia'

map$location[map$location == "Micronesia (Federated States of)"] = 'Micronesia'

map$location[map$location == "Macedonia"] = 'North Macedonia'

map$location[map$location == "Trinidad and Tobago"] = 'Trinidad'

a <- map[map$location == "Trinidad",]

a$location <- 'Tobago'

map <- rbind(map,a)

map$location[map$location == "Cabo Verde"] = 'Cape Verde'

map$location[map$location == "United States Virgin Islands"] = 'Virgin Islands'

map$location[map$location == "Antigua and Barbuda"] = 'Antigu'

a <- map[map$location == "Antigu",]

a$location <- 'Barbuda'

map <- rbind(map,a)

map$location[map$location == "Saint Kitts and Nevis"] = 'Saint Kitts'

a <- map[map$location == "Saint Kitts",]

a$location <- 'Nevis'

map <- rbind(map,a)

map$location[map$location == "Côte d'Ivoire"] = 'Ivory Coast'

map$location[map$location == "Saint Vincent and the Grenadines"] = 'Saint Vincent'

a <- map[map$location == "Saint Vincent",]

a$location <- 'Grenadines'

map <- rbind(map,a)

map$location[map$location == "Eswatini"] = 'Swaziland'

map$location[map$location == "Brunei Darussalam"] = 'Brunei'

map <- full_join(world,map,by = c('region'='location')) %>%

filter(val != "NA")

mycolor2<-brewer.pal(5, "Blues")

fig <- map %>%

ggplot()+

geom_polygon(aes(x = long, y = lat,group = group,fill=val2),colour="white",

size=0.2) +

theme_void()+

scale_fill_manual(values=mycolor2) +

theme(legend.position = c(0.1,0.2),

legend.title = element_blank(),

legend.text = element_text(color="black",

size = 12,

),

plot.title = element_blank(),

panel.grid=element_blank(),

axis.title.x = element_blank(),

axis.text.x = element_blank(),

axis.ticks.x = element_blank(),

axis.title.y = element_blank(),

axis.text.y = element_blank(),

axis.ticks.y = element_blank(),

)

fig

IS1 <- IS %>%

filter(measure == "Prevalence") %>%

filter(year == 2021) %>%

filter(sex == "Both") %>%

filter(age == "Age-standardized") %>%

filter(metric == "Rate")

summary(IS1$val)

quantile(IS1$val,seq(0.1,1,0.1))

map <- IS1%>% mutate(val2 = cut(val, breaks = c(0,1,2,3,4,10000000),

labels = c("<1","1-2","2-3","3-4",">4"),

include.lowest = T,right = T))

map$location[map$location == 'United States of America'] = 'USA'

map$location[map$location == 'Russian Federation'] = 'Russia'

map$location[map$location == 'United Kingdom'] = 'UK'

map$location[map$location == 'Congo'] = 'Republic of Congo'

map$location[map$location == "Iran (Islamic Republic of)"] = 'Iran'

map$location[map$location == "Democratic People's Republic of Korea"] = 'North Korea'

map$location[map$location == "Taiwan (Province of China)"] = 'Taiwan'

map$location[map$location == "Republic of Korea"] = 'South Korea'

map$location[map$location == "United Republic of Tanzania"] = 'Tanzania'

map$location[map$location == "Bolivia (Plurinational State of)"] = 'Bolivia'

map$location[map$location == "Venezuela (Bolivarian Republic of)"] = 'Venezuela'

map$location[map$location == "Czechia"] = 'Czech Republic'

map$location[map$location == "Republic of Moldova"] = 'Moldova'

map$location[map$location == "Viet Nam"] = 'Vietnam'

map$location[map$location == "Lao People's Democratic Republic"] = 'Laos'

map$location[map$location == "Syrian Arab Republic"] = 'Syria'

map$location[map$location == "North Macedonia"] = 'Macedonia'

map$location[map$location == "Micronesia (Federated States of)"] = 'Micronesia'

map$location[map$location == "Macedonia"] = 'North Macedonia'

map$location[map$location == "Trinidad and Tobago"] = 'Trinidad'

a <- map[map$location == "Trinidad",]

a$location <- 'Tobago'

map <- rbind(map,a)

map$location[map$location == "Cabo Verde"] = 'Cape Verde'

map$location[map$location == "United States Virgin Islands"] = 'Virgin Islands'

map$location[map$location == "Antigua and Barbuda"] = 'Antigu'

a <- map[map$location == "Antigu",]

a$location <- 'Barbuda'

map <- rbind(map,a)

map$location[map$location == "Saint Kitts and Nevis"] = 'Saint Kitts'

a <- map[map$location == "Saint Kitts",]

a$location <- 'Nevis'

map <- rbind(map,a)

map$location[map$location == "Côte d'Ivoire"] = 'Ivory Coast'

map$location[map$location == "Saint Vincent and the Grenadines"] = 'Saint Vincent'

a <- map[map$location == "Saint Vincent",]

a$location <- 'Grenadines'

map <- rbind(map,a)

map$location[map$location == "Eswatini"] = 'Swaziland'

map$location[map$location == "Brunei Darussalam"] = 'Brunei'

map <- full_join(world,map,by = c('region'='location')) %>%

filter(val != "NA")

mycolor2<-brewer.pal(5, "Blues")

fig <- map %>%

ggplot()+

geom_polygon(aes(x = long, y = lat,group = group,fill=val2),colour="white",

size=0.2) +

theme_void()+

scale_fill_manual(values=mycolor2) +

theme(legend.position = c(0.1,0.2),

legend.title = element_blank(),

legend.text = element_text(color="black",

size = 12,

),

plot.title = element_blank(),

panel.grid=element_blank(),

axis.title.x = element_blank(),

axis.text.x = element_blank(),

axis.ticks.x = element_blank(),

axis.title.y = element_blank(),

axis.text.y = element_blank(),

axis.ticks.y = element_blank(),

)

fig

IS1 <- IS %>%

filter(measure == "Deaths") %>%

filter(year == 2021) %>%

filter(sex == "Both") %>%

filter(age == "Age-standardized") %>%

filter(metric == "Rate")

summary(IS1$val)

quantile(IS1$val,seq(0.1,1,0.1))

map <- IS1%>% mutate(val2 = cut(val, breaks = c(0,1,2,3,4,10000000),

labels = c("<1","1-2","2-3","3-4",">4"),

include.lowest = T,right = T))

map$location[map$location == 'United States of America'] = 'USA'

map$location[map$location == 'Russian Federation'] = 'Russia'

map$location[map$location == 'United Kingdom'] = 'UK'

map$location[map$location == 'Congo'] = 'Republic of Congo'

map$location[map$location == "Iran (Islamic Republic of)"] = 'Iran'

map$location[map$location == "Democratic People's Republic of Korea"] = 'North Korea'

map$location[map$location == "Taiwan (Province of China)"] = 'Taiwan'

map$location[map$location == "Republic of Korea"] = 'South Korea'

map$location[map$location == "United Republic of Tanzania"] = 'Tanzania'

map$location[map$location == "Bolivia (Plurinational State of)"] = 'Bolivia'

map$location[map$location == "Venezuela (Bolivarian Republic of)"] = 'Venezuela'

map$location[map$location == "Czechia"] = 'Czech Republic'

map$location[map$location == "Republic of Moldova"] = 'Moldova'

map$location[map$location == "Viet Nam"] = 'Vietnam'

map$location[map$location == "Lao People's Democratic Republic"] = 'Laos'

map$location[map$location == "Syrian Arab Republic"] = 'Syria'

map$location[map$location == "North Macedonia"] = 'Macedonia'

map$location[map$location == "Micronesia (Federated States of)"] = 'Micronesia'

map$location[map$location == "Macedonia"] = 'North Macedonia'

map$location[map$location == "Trinidad and Tobago"] = 'Trinidad'

a <- map[map$location == "Trinidad",]

a$location <- 'Tobago'

map <- rbind(map,a)

map$location[map$location == "Cabo Verde"] = 'Cape Verde'

map$location[map$location == "United States Virgin Islands"] = 'Virgin Islands'

map$location[map$location == "Antigua and Barbuda"] = 'Antigu'

a <- map[map$location == "Antigu",]

a$location <- 'Barbuda'

map <- rbind(map,a)

map$location[map$location == "Saint Kitts and Nevis"] = 'Saint Kitts'

a <- map[map$location == "Saint Kitts",]

a$location <- 'Nevis'

map <- rbind(map,a)

map$location[map$location == "Côte d'Ivoire"] = 'Ivory Coast'

map$location[map$location == "Saint Vincent and the Grenadines"] = 'Saint Vincent'

a <- map[map$location == "Saint Vincent",]

a$location <- 'Grenadines'

map <- rbind(map,a)

map$location[map$location == "Eswatini"] = 'Swaziland'

map$location[map$location == "Brunei Darussalam"] = 'Brunei'

map <- full_join(world,map,by = c('region'='location')) %>%

filter(val != "NA")

mycolor2<-brewer.pal(5, "Blues")

fig <- map %>%

ggplot()+

geom_polygon(aes(x = long, y = lat,group = group,fill=val2),colour="white",

size=0.2) +

theme_void()+

scale_fill_manual(values=mycolor2) +

theme(legend.position = c(0.1,0.2),

legend.title = element_blank(),

legend.text = element_text(color="black",

size = 12,

),

plot.title = element_blank(),

panel.grid=element_blank(),

axis.title.x = element_blank(),

axis.text.x = element_blank(),

axis.ticks.x = element_blank(),

axis.title.y = element_blank(),

axis.text.y = element_blank(),

axis.ticks.y = element_blank(),

)

fig

IS1 <- IS %>%

filter(measure == "DALYs") %>%

filter(year == 2021) %>%

filter(sex == "Both") %>%

filter(age == "Age-standardized") %>%

filter(metric == "Rate")

summary(IS1$val)

quantile(IS1$val,seq(0.1,1,0.1))

map <- IS1%>% mutate(val2 = cut(val, breaks = c(0,1,2,3,4,10000000),

labels = c("<1","1-2","2-3","3-4",">4"),

include.lowest = T,right = T))

map$location[map$location == 'United States of America'] = 'USA'

map$location[map$location == 'Russian Federation'] = 'Russia'

map$location[map$location == 'United Kingdom'] = 'UK'

map$location[map$location == 'Congo'] = 'Republic of Congo'

map$location[map$location == "Iran (Islamic Republic of)"] = 'Iran'

map$location[map$location == "Democratic People's Republic of Korea"] = 'North Korea'

map$location[map$location == "Taiwan (Province of China)"] = 'Taiwan'

map$location[map$location == "Republic of Korea"] = 'South Korea'

map$location[map$location == "United Republic of Tanzania"] = 'Tanzania'

map$location[map$location == "Bolivia (Plurinational State of)"] = 'Bolivia'

map$location[map$location == "Venezuela (Bolivarian Republic of)"] = 'Venezuela'

map$location[map$location == "Czechia"] = 'Czech Republic'

map$location[map$location == "Republic of Moldova"] = 'Moldova'

map$location[map$location == "Viet Nam"] = 'Vietnam'

map$location[map$location == "Lao People's Democratic Republic"] = 'Laos'

map$location[map$location == "Syrian Arab Republic"] = 'Syria'

map$location[map$location == "North Macedonia"] = 'Macedonia'

map$location[map$location == "Micronesia (Federated States of)"] = 'Micronesia'

map$location[map$location == "Macedonia"] = 'North Macedonia'

map$location[map$location == "Trinidad and Tobago"] = 'Trinidad'

a <- map[map$location == "Trinidad",]

a$location <- 'Tobago'

map <- rbind(map,a)

map$location[map$location == "Cabo Verde"] = 'Cape Verde'

map$location[map$location == "United States Virgin Islands"] = 'Virgin Islands'

map$location[map$location == "Antigua and Barbuda"] = 'Antigu'

a <- map[map$location == "Antigu",]

a$location <- 'Barbuda'

map <- rbind(map,a)

map$location[map$location == "Saint Kitts and Nevis"] = 'Saint Kitts'

a <- map[map$location == "Saint Kitts",]

a$location <- 'Nevis'

map <- rbind(map,a)

map$location[map$location == "Côte d'Ivoire"] = 'Ivory Coast'

map$location[map$location == "Saint Vincent and the Grenadines"] = 'Saint Vincent'

a <- map[map$location == "Saint Vincent",]

a$location <- 'Grenadines'

map <- rbind(map,a)

map$location[map$location == "Eswatini"] = 'Swaziland'

map$location[map$location == "Brunei Darussalam"] = 'Brunei'

map <- full_join(world,map,by = c('region'='location')) %>%

filter(val != "NA")

mycolor2<-brewer.pal(5, "Blues")

fig <- map %>%

ggplot()+

geom_polygon(aes(x = long, y = lat,group = group,fill=val2),colour="white",

size=0.2) +

theme_void()+

scale_fill_manual(values=mycolor2) +

theme(legend.position = c(0.1,0.2),

legend.title = element_blank(),

legend.text = element_text(color="black",

size = 12,

),

plot.title = element_blank(),

panel.grid=element_blank(),

axis.title.x = element_blank(),

axis.text.x = element_blank(),

axis.ticks.x = element_blank(),

axis.title.y = element_blank(),

axis.text.y = element_blank(),

axis.ticks.y = element_blank(),

)

fig

IS1 <- IS %>%

filter(measure == "Incidence") %>%

filter(year == 2021) %>%

filter(sex == "Both") %>%

filter(age == "All ages") %>%

filter(metric == "Number")

summary(IS1$val)

quantile(IS1$val,seq(0.1,1,0.1))

map <- IS1%>% mutate(val2 = cut(val, breaks = c(0,1,2,3,4,10000000),

labels = c("<1","1-2","2-3","3-4",">4"),

include.lowest = T,right = T))

map$location[map$location == 'United States of America'] = 'USA'

map$location[map$location == 'Russian Federation'] = 'Russia'

map$location[map$location == 'United Kingdom'] = 'UK'

map$location[map$location == 'Congo'] = 'Republic of Congo'

map$location[map$location == "Iran (Islamic Republic of)"] = 'Iran'

map$location[map$location == "Democratic People's Republic of Korea"] = 'North Korea'

map$location[map$location == "Taiwan (Province of China)"] = 'Taiwan'

map$location[map$location == "Republic of Korea"] = 'South Korea'

map$location[map$location == "United Republic of Tanzania"] = 'Tanzania'

map$location[map$location == "Bolivia (Plurinational State of)"] = 'Bolivia'

map$location[map$location == "Venezuela (Bolivarian Republic of)"] = 'Venezuela'

map$location[map$location == "Czechia"] = 'Czech Republic'

map$location[map$location == "Republic of Moldova"] = 'Moldova'

map$location[map$location == "Viet Nam"] = 'Vietnam'

map$location[map$location == "Lao People's Democratic Republic"] = 'Laos'

map$location[map$location == "Syrian Arab Republic"] = 'Syria'

map$location[map$location == "North Macedonia"] = 'Macedonia'

map$location[map$location == "Micronesia (Federated States of)"] = 'Micronesia'

map$location[map$location == "Macedonia"] = 'North Macedonia'

map$location[map$location == "Trinidad and Tobago"] = 'Trinidad'

a <- map[map$location == "Trinidad",]

a$location <- 'Tobago'

map <- rbind(map,a)

map$location[map$location == "Cabo Verde"] = 'Cape Verde'

map$location[map$location == "United States Virgin Islands"] = 'Virgin Islands'

map$location[map$location == "Antigua and Barbuda"] = 'Antigu'

a <- map[map$location == "Antigu",]

a$location <- 'Barbuda'

map <- rbind(map,a)

map$location[map$location == "Saint Kitts and Nevis"] = 'Saint Kitts'

a <- map[map$location == "Saint Kitts",]

a$location <- 'Nevis'

map <- rbind(map,a)

map$location[map$location == "Côte d'Ivoire"] = 'Ivory Coast'

map$location[map$location == "Saint Vincent and the Grenadines"] = 'Saint Vincent'

a <- map[map$location == "Saint Vincent",]

a$location <- 'Grenadines'

map <- rbind(map,a)

map$location[map$location == "Eswatini"] = 'Swaziland'

map$location[map$location == "Brunei Darussalam"] = 'Brunei'

map <- full_join(world,map,by = c('region'='location')) %>%

filter(val != "NA")

mycolor2<-brewer.pal(5, "Blues")

fig <- map %>%

ggplot()+

geom_polygon(aes(x = long, y = lat,group = group,fill=val2),colour="white",

size=0.2) +

theme_void()+

scale_fill_manual(values=mycolor2) +

theme(legend.position = c(0.1,0.2),

legend.title = element_blank(),

legend.text = element_text(color="black",

size = 12,

),

plot.title = element_blank(),

panel.grid=element_blank(),

axis.title.x = element_blank(),

axis.text.x = element_blank(),

axis.ticks.x = element_blank(),

axis.title.y = element_blank(),

axis.text.y = element_blank(),

axis.ticks.y = element_blank(),

)

fig

IS1 <- IS %>%

filter(measure == "Prevalence") %>%

filter(year == 2021) %>%

filter(sex == "Both") %>%

filter(age == "All ages") %>%

filter(metric == "Number")

summary(IS1$val)

quantile(IS1$val,seq(0.1,1,0.1))

map <- IS1%>% mutate(val2 = cut(val, breaks = c(0,1,2,3,4,10000000),

labels = c("<1","1-2","2-3","3-4",">4"),

include.lowest = T,right = T))

map$location[map$location == 'United States of America'] = 'USA'

map$location[map$location == 'Russian Federation'] = 'Russia'

map$location[map$location == 'United Kingdom'] = 'UK'

map$location[map$location == 'Congo'] = 'Republic of Congo'

map$location[map$location == "Iran (Islamic Republic of)"] = 'Iran'

map$location[map$location == "Democratic People's Republic of Korea"] = 'North Korea'

map$location[map$location == "Taiwan (Province of China)"] = 'Taiwan'

map$location[map$location == "Republic of Korea"] = 'South Korea'

map$location[map$location == "United Republic of Tanzania"] = 'Tanzania'

map$location[map$location == "Bolivia (Plurinational State of)"] = 'Bolivia'

map$location[map$location == "Venezuela (Bolivarian Republic of)"] = 'Venezuela'

map$location[map$location == "Czechia"] = 'Czech Republic'

map$location[map$location == "Republic of Moldova"] = 'Moldova'

map$location[map$location == "Viet Nam"] = 'Vietnam'

map$location[map$location == "Lao People's Democratic Republic"] = 'Laos'

map$location[map$location == "Syrian Arab Republic"] = 'Syria'

map$location[map$location == "North Macedonia"] = 'Macedonia'

map$location[map$location == "Micronesia (Federated States of)"] = 'Micronesia'

map$location[map$location == "Macedonia"] = 'North Macedonia'

map$location[map$location == "Trinidad and Tobago"] = 'Trinidad'

a <- map[map$location == "Trinidad",]

a$location <- 'Tobago'

map <- rbind(map,a)

map$location[map$location == "Cabo Verde"] = 'Cape Verde'

map$location[map$location == "United States Virgin Islands"] = 'Virgin Islands'

map$location[map$location == "Antigua and Barbuda"] = 'Antigu'

a <- map[map$location == "Antigu",]

a$location <- 'Barbuda'

map <- rbind(map,a)

map$location[map$location == "Saint Kitts and Nevis"] = 'Saint Kitts'

a <- map[map$location == "Saint Kitts",]

a$location <- 'Nevis'

map <- rbind(map,a)

map$location[map$location == "Côte d'Ivoire"] = 'Ivory Coast'

map$location[map$location == "Saint Vincent and the Grenadines"] = 'Saint Vincent'

a <- map[map$location == "Saint Vincent",]

a$location <- 'Grenadines'

map <- rbind(map,a)

map$location[map$location == "Eswatini"] = 'Swaziland'

map$location[map$location == "Brunei Darussalam"] = 'Brunei'

map <- full_join(world,map,by = c('region'='location')) %>%

filter(val != "NA")

mycolor2<-brewer.pal(5, "Blues")

fig <- map %>%

ggplot()+

geom_polygon(aes(x = long, y = lat,group = group,fill=val2),colour="white",

size=0.2) +

theme_void()+

scale_fill_manual(values=mycolor2) +

theme(legend.position = c(0.1,0.2),

legend.title = element_blank(),

legend.text = element_text(color="black",

size = 12,

),

plot.title = element_blank(),

panel.grid=element_blank(),

axis.title.x = element_blank(),

axis.text.x = element_blank(),

axis.ticks.x = element_blank(),

axis.title.y = element_blank(),

axis.text.y = element_blank(),

axis.ticks.y = element_blank(),

)

fig

IS1 <- IS %>%

filter(measure == "Deaths") %>%

filter(year == 2021) %>%

filter(sex == "Both") %>%

filter(age == "All ages") %>%

filter(metric == "Number")

summary(IS1$val)

quantile(IS1$val,seq(0.1,1,0.1))

map <- IS1%>% mutate(val2 = cut(val, breaks = c(0,1,2,3,4,10000000),

labels = c("<1","1-2","2-3","3-4",">4"),

include.lowest = T,right = T))

map$location[map$location == 'United States of America'] = 'USA'

map$location[map$location == 'Russian Federation'] = 'Russia'

map$location[map$location == 'United Kingdom'] = 'UK'

map$location[map$location == 'Congo'] = 'Republic of Congo'

map$location[map$location == "Iran (Islamic Republic of)"] = 'Iran'

map$location[map$location == "Democratic People's Republic of Korea"] = 'North Korea'

map$location[map$location == "Taiwan (Province of China)"] = 'Taiwan'

map$location[map$location == "Republic of Korea"] = 'South Korea'

map$location[map$location == "United Republic of Tanzania"] = 'Tanzania'

map$location[map$location == "Bolivia (Plurinational State of)"] = 'Bolivia'

map$location[map$location == "Venezuela (Bolivarian Republic of)"] = 'Venezuela'

map$location[map$location == "Czechia"] = 'Czech Republic'

map$location[map$location == "Republic of Moldova"] = 'Moldova'

map$location[map$location == "Viet Nam"] = 'Vietnam'

map$location[map$location == "Lao People's Democratic Republic"] = 'Laos'

map$location[map$location == "Syrian Arab Republic"] = 'Syria'

map$location[map$location == "North Macedonia"] = 'Macedonia'

map$location[map$location == "Micronesia (Federated States of)"] = 'Micronesia'

map$location[map$location == "Macedonia"] = 'North Macedonia'

map$location[map$location == "Trinidad and Tobago"] = 'Trinidad'

a <- map[map$location == "Trinidad",]

a$location <- 'Tobago'

map <- rbind(map,a)

map$location[map$location == "Cabo Verde"] = 'Cape Verde'

map$location[map$location == "United States Virgin Islands"] = 'Virgin Islands'

map$location[map$location == "Antigua and Barbuda"] = 'Antigu'

a <- map[map$location == "Antigu",]

a$location <- 'Barbuda'

map <- rbind(map,a)

map$location[map$location == "Saint Kitts and Nevis"] = 'Saint Kitts'

a <- map[map$location == "Saint Kitts",]

a$location <- 'Nevis'

map <- rbind(map,a)

map$location[map$location == "Côte d'Ivoire"] = 'Ivory Coast'

map$location[map$location == "Saint Vincent and the Grenadines"] = 'Saint Vincent'

a <- map[map$location == "Saint Vincent",]

a$location <- 'Grenadines'

map <- rbind(map,a)

map$location[map$location == "Eswatini"] = 'Swaziland'

map$location[map$location == "Brunei Darussalam"] = 'Brunei'

map <- full_join(world,map,by = c('region'='location')) %>%

filter(val != "NA")

mycolor2<-brewer.pal(5, "Blues")

fig <- map %>%

ggplot()+

geom_polygon(aes(x = long, y = lat,group = group,fill=val2),colour="white",

size=0.2) +

theme_void()+

scale_fill_manual(values=mycolor2) +

theme(legend.position = c(0.1,0.2),

legend.title = element_blank(),

legend.text = element_text(color="black",

size = 12,

),

plot.title = element_blank(),

panel.grid=element_blank(),

axis.title.x = element_blank(),

axis.text.x = element_blank(),

axis.ticks.x = element_blank(),

axis.title.y = element_blank(),

axis.text.y = element_blank(),

axis.ticks.y = element_blank(),

)

fig

IS1 <- IS %>%

filter(measure == "DALYs") %>%

filter(year == 2021) %>%

filter(sex == "Both") %>%

filter(age == "All ages") %>%

filter(metric == "Number")

summary(IS1$val)

quantile(IS1$val,seq(0.1,1,0.1))

map <- IS1%>% mutate(val2 = cut(val, breaks = c(0,1,2,3,4,10000000),

labels = c("<1","1-2","2-3","3-4",">4"),

include.lowest = T,right = T))

map$location[map$location == 'United States of America'] = 'USA'

map$location[map$location == 'Russian Federation'] = 'Russia'

map$location[map$location == 'United Kingdom'] = 'UK'

map$location[map$location == 'Congo'] = 'Republic of Congo'

map$location[map$location == "Iran (Islamic Republic of)"] = 'Iran'

map$location[map$location == "Democratic People's Republic of Korea"] = 'North Korea'

map$location[map$location == "Taiwan (Province of China)"] = 'Taiwan'

map$location[map$location == "Republic of Korea"] = 'South Korea'

map$location[map$location == "United Republic of Tanzania"] = 'Tanzania'

map$location[map$location == "Bolivia (Plurinational State of)"] = 'Bolivia'

map$location[map$location == "Venezuela (Bolivarian Republic of)"] = 'Venezuela'

map$location[map$location == "Czechia"] = 'Czech Republic'

map$location[map$location == "Republic of Moldova"] = 'Moldova'

map$location[map$location == "Viet Nam"] = 'Vietnam'

map$location[map$location == "Lao People's Democratic Republic"] = 'Laos'

map$location[map$location == "Syrian Arab Republic"] = 'Syria'

map$location[map$location == "North Macedonia"] = 'Macedonia'

map$location[map$location == "Micronesia (Federated States of)"] = 'Micronesia'

map$location[map$location == "Macedonia"] = 'North Macedonia'

map$location[map$location == "Trinidad and Tobago"] = 'Trinidad'

a <- map[map$location == "Trinidad",]

a$location <- 'Tobago'

map <- rbind(map,a)

map$location[map$location == "Cabo Verde"] = 'Cape Verde'

map$location[map$location == "United States Virgin Islands"] = 'Virgin Islands'

map$location[map$location == "Antigua and Barbuda"] = 'Antigu'

a <- map[map$location == "Antigu",]

a$location <- 'Barbuda'

map <- rbind(map,a)

map$location[map$location == "Saint Kitts and Nevis"] = 'Saint Kitts'

a <- map[map$location == "Saint Kitts",]

a$location <- 'Nevis'

map <- rbind(map,a)

map$location[map$location == "Côte d'Ivoire"] = 'Ivory Coast'

map$location[map$location == "Saint Vincent and the Grenadines"] = 'Saint Vincent'

a <- map[map$location == "Saint Vincent",]

a$location <- 'Grenadines'

map <- rbind(map,a)

map$location[map$location == "Eswatini"] = 'Swaziland'

map$location[map$location == "Brunei Darussalam"] = 'Brunei'

map <- full_join(world,map,by = c('region'='location')) %>%

filter(val != "NA")

mycolor2<-brewer.pal(5, "Blues")

fig <- map %>%

ggplot()+

geom_polygon(aes(x = long, y = lat,group = group,fill=val2),colour="white",

size=0.2) +

theme_void()+

scale_fill_manual(values=mycolor2) +

theme(legend.position = c(0.1,0.2),

legend.title = element_blank(),

legend.text = element_text(color="black",

size = 12,

),

plot.title = element_blank(),

panel.grid=element_blank(),

axis.title.x = element_blank(),

axis.text.x = element_blank(),

axis.ticks.x = element_blank(),

axis.title.y = element_blank(),

axis.text.y = element_blank(),

axis.ticks.y = element_blank(),

)

fig

world <- map_data('world')

MM_country<- vroom::vroom("MM_country.csv")

IS <- MM_country %>%

dplyr::select(measure_name,location_name,metric_name,

sex_name,age_name,year,val,lower,upper) %>%

rename(measure=measure_name,

location=location_name,

metric=metric_name,

sex=sex_name,

age=age_name)

IS1 <- IS %>%

filter(measure == "Incidence") %>%

filter(year == 2021) %>%

filter(sex == "Both") %>%

filter(age == "Age-standardized") %>%

filter(metric == "Rate")

summary(IS1$val)

quantile(IS1$val,seq(0.1,1,0.1))

map <- IS1%>% mutate(val2 = cut(val, breaks = c(0,1,2,3,4,10000000),

labels = c("<1","1-2","2-3","3-4",">4"),

include.lowest = T,right = T))

map$location[map$location == 'United States of America'] = 'USA'

map$location[map$location == 'Russian Federation'] = 'Russia'

map$location[map$location == 'United Kingdom'] = 'UK'

map$location[map$location == 'Congo'] = 'Republic of Congo'

map$location[map$location == "Iran (Islamic Republic of)"] = 'Iran'

map$location[map$location == "Democratic People's Republic of Korea"] = 'North Korea'

map$location[map$location == "Taiwan (Province of China)"] = 'Taiwan'

map$location[map$location == "Republic of Korea"] = 'South Korea'

map$location[map$location == "United Republic of Tanzania"] = 'Tanzania'

map$location[map$location == "Bolivia (Plurinational State of)"] = 'Bolivia'

map$location[map$location == "Venezuela (Bolivarian Republic of)"] = 'Venezuela'

map$location[map$location == "Czechia"] = 'Czech Republic'

map$location[map$location == "Republic of Moldova"] = 'Moldova'

map$location[map$location == "Viet Nam"] = 'Vietnam'

map$location[map$location == "Lao People's Democratic Republic"] = 'Laos'

map$location[map$location == "Syrian Arab Republic"] = 'Syria'

map$location[map$location == "North Macedonia"] = 'Macedonia'

map$location[map$location == "Micronesia (Federated States of)"] = 'Micronesia'

map$location[map$location == "Macedonia"] = 'North Macedonia'

map$location[map$location == "Trinidad and Tobago"] = 'Trinidad'

a <- map[map$location == "Trinidad",]

a$location <- 'Tobago'

map <- rbind(map,a)

map$location[map$location == "Cabo Verde"] = 'Cape Verde'

map$location[map$location == "United States Virgin Islands"] = 'Virgin Islands'

map$location[map$location == "Antigua and Barbuda"] = 'Antigu'

a <- map[map$location == "Antigu",]

a$location <- 'Barbuda'

map <- rbind(map,a)

map$location[map$location == "Saint Kitts and Nevis"] = 'Saint Kitts'

a <- map[map$location == "Saint Kitts",]

a$location <- 'Nevis'

map <- rbind(map,a)

map$location[map$location == "Côte d'Ivoire"] = 'Ivory Coast'

map$location[map$location == "Saint Vincent and the Grenadines"] = 'Saint Vincent'

a <- map[map$location == "Saint Vincent",]

a$location <- 'Grenadines'

map <- rbind(map,a)

map$location[map$location == "Eswatini"] = 'Swaziland'

map$location[map$location == "Brunei Darussalam"] = 'Brunei'

map <- full_join(world,map,by = c('region'='location')) %>%

filter(val != "NA")

mycolor2<-brewer.pal(5, "Blues")

fig <- map %>%

ggplot()+

geom_polygon(aes(x = long, y = lat,group = group,fill=val2),colour="white",

size=0.2) +

theme_void()+

scale_fill_manual(values=mycolor2) +

theme(legend.position = c(0.1,0.2),

legend.title = element_blank(),

legend.text = element_text(color="black",

size = 12,

),

plot.title = element_blank(),

panel.grid=element_blank(),

axis.title.x = element_blank(),

axis.text.x = element_blank(),

axis.ticks.x = element_blank(),

axis.title.y = element_blank(),

axis.text.y = element_blank(),

axis.ticks.y = element_blank(),

)

fig

IS1 <- IS %>%

filter(measure == "Prevalence") %>%

filter(year == 2021) %>%

filter(sex == "Both") %>%

filter(age == "Age-standardized") %>%

filter(metric == "Rate")

summary(IS1$val)

quantile(IS1$val,seq(0.1,1,0.1))

map <- IS1%>% mutate(val2 = cut(val, breaks = c(0,1,2,3,4,10000000),

labels = c("<1","1-2","2-3","3-4",">4"),

include.lowest = T,right = T))

map$location[map$location == 'United States of America'] = 'USA'

map$location[map$location == 'Russian Federation'] = 'Russia'

map$location[map$location == 'United Kingdom'] = 'UK'

map$location[map$location == 'Congo'] = 'Republic of Congo'

map$location[map$location == "Iran (Islamic Republic of)"] = 'Iran'

map$location[map$location == "Democratic People's Republic of Korea"] = 'North Korea'

map$location[map$location == "Taiwan (Province of China)"] = 'Taiwan'

map$location[map$location == "Republic of Korea"] = 'South Korea'

map$location[map$location == "United Republic of Tanzania"] = 'Tanzania'

map$location[map$location == "Bolivia (Plurinational State of)"] = 'Bolivia'

map$location[map$location == "Venezuela (Bolivarian Republic of)"] = 'Venezuela'

map$location[map$location == "Czechia"] = 'Czech Republic'

map$location[map$location == "Republic of Moldova"] = 'Moldova'

map$location[map$location == "Viet Nam"] = 'Vietnam'

map$location[map$location == "Lao People's Democratic Republic"] = 'Laos'

map$location[map$location == "Syrian Arab Republic"] = 'Syria'

map$location[map$location == "North Macedonia"] = 'Macedonia'

map$location[map$location == "Micronesia (Federated States of)"] = 'Micronesia'

map$location[map$location == "Macedonia"] = 'North Macedonia'

map$location[map$location == "Trinidad and Tobago"] = 'Trinidad'

a <- map[map$location == "Trinidad",]

a$location <- 'Tobago'

map <- rbind(map,a)

map$location[map$location == "Cabo Verde"] = 'Cape Verde'

map$location[map$location == "United States Virgin Islands"] = 'Virgin Islands'

map$location[map$location == "Antigua and Barbuda"] = 'Antigu'

a <- map[map$location == "Antigu",]

a$location <- 'Barbuda'

map <- rbind(map,a)

map$location[map$location == "Saint Kitts and Nevis"] = 'Saint Kitts'

a <- map[map$location == "Saint Kitts",]

a$location <- 'Nevis'

map <- rbind(map,a)

map$location[map$location == "Côte d'Ivoire"] = 'Ivory Coast'

map$location[map$location == "Saint Vincent and the Grenadines"] = 'Saint Vincent'

a <- map[map$location == "Saint Vincent",]

a$location <- 'Grenadines'

map <- rbind(map,a)

map$location[map$location == "Eswatini"] = 'Swaziland'

map$location[map$location == "Brunei Darussalam"] = 'Brunei'

map <- full_join(world,map,by = c('region'='location')) %>%

filter(val != "NA")

mycolor2<-brewer.pal(5, "Blues")

fig <- map %>%

ggplot()+

geom_polygon(aes(x = long, y = lat,group = group,fill=val2),colour="white",

size=0.2) +

theme_void()+

scale_fill_manual(values=mycolor2) +

theme(legend.position = c(0.1,0.2),

legend.title = element_blank(),

legend.text = element_text(color="black",

size = 12,

),

plot.title = element_blank(),

panel.grid=element_blank(),

axis.title.x = element_blank(),

axis.text.x = element_blank(),

axis.ticks.x = element_blank(),

axis.title.y = element_blank(),

axis.text.y = element_blank(),

axis.ticks.y = element_blank(),

)

fig

IS1 <- IS %>%

filter(measure == "Deaths") %>%

filter(year == 2021) %>%

filter(sex == "Both") %>%

filter(age == "Age-standardized") %>%

filter(metric == "Rate")

summary(IS1$val)

quantile(IS1$val,seq(0.1,1,0.1))

map <- IS1%>% mutate(val2 = cut(val, breaks = c(0,1,2,3,4,10000000),

labels = c("<1","1-2","2-3","3-4",">4"),

include.lowest = T,right = T))

map$location[map$location == 'United States of America'] = 'USA'

map$location[map$location == 'Russian Federation'] = 'Russia'

map$location[map$location == 'United Kingdom'] = 'UK'

map$location[map$location == 'Congo'] = 'Republic of Congo'

map$location[map$location == "Iran (Islamic Republic of)"] = 'Iran'

map$location[map$location == "Democratic People's Republic of Korea"] = 'North Korea'

map$location[map$location == "Taiwan (Province of China)"] = 'Taiwan'

map$location[map$location == "Republic of Korea"] = 'South Korea'

map$location[map$location == "United Republic of Tanzania"] = 'Tanzania'

map$location[map$location == "Bolivia (Plurinational State of)"] = 'Bolivia'

map$location[map$location == "Venezuela (Bolivarian Republic of)"] = 'Venezuela'

map$location[map$location == "Czechia"] = 'Czech Republic'

map$location[map$location == "Republic of Moldova"] = 'Moldova'

map$location[map$location == "Viet Nam"] = 'Vietnam'

map$location[map$location == "Lao People's Democratic Republic"] = 'Laos'

map$location[map$location == "Syrian Arab Republic"] = 'Syria'

map$location[map$location == "North Macedonia"] = 'Macedonia'

map$location[map$location == "Micronesia (Federated States of)"] = 'Micronesia'

map$location[map$location == "Macedonia"] = 'North Macedonia'

map$location[map$location == "Trinidad and Tobago"] = 'Trinidad'

a <- map[map$location == "Trinidad",]

a$location <- 'Tobago'

map <- rbind(map,a)

map$location[map$location == "Cabo Verde"] = 'Cape Verde'

map$location[map$location == "United States Virgin Islands"] = 'Virgin Islands'

map$location[map$location == "Antigua and Barbuda"] = 'Antigu'

a <- map[map$location == "Antigu",]

a$location <- 'Barbuda'

map <- rbind(map,a)

map$location[map$location == "Saint Kitts and Nevis"] = 'Saint Kitts'

a <- map[map$location == "Saint Kitts",]

a$location <- 'Nevis'

map <- rbind(map,a)

map$location[map$location == "Côte d'Ivoire"] = 'Ivory Coast'

map$location[map$location == "Saint Vincent and the Grenadines"] = 'Saint Vincent'

a <- map[map$location == "Saint Vincent",]

a$location <- 'Grenadines'

map <- rbind(map,a)

map$location[map$location == "Eswatini"] = 'Swaziland'

map$location[map$location == "Brunei Darussalam"] = 'Brunei'

map <- full_join(world,map,by = c('region'='location')) %>%

filter(val != "NA")

mycolor2<-brewer.pal(5, "Blues")

fig <- map %>%

ggplot()+

geom_polygon(aes(x = long, y = lat,group = group,fill=val2),colour="white",

size=0.2) +

theme_void()+

scale_fill_manual(values=mycolor2) +

theme(legend.position = c(0.1,0.2),

legend.title = element_blank(),

legend.text = element_text(color="black",

size = 12,

),

plot.title = element_blank(),

panel.grid=element_blank(),

axis.title.x = element_blank(),

axis.text.x = element_blank(),

axis.ticks.x = element_blank(),

axis.title.y = element_blank(),

axis.text.y = element_blank(),

axis.ticks.y = element_blank(),

)

fig

IS1 <- IS %>%

filter(measure == "DALYs") %>%

filter(year == 2021) %>%

filter(sex == "Both") %>%

filter(age == "Age-standardized") %>%

filter(metric == "Rate")

summary(IS1$val)

quantile(IS1$val,seq(0.1,1,0.1))

map <- IS1%>% mutate(val2 = cut(val, breaks = c(0,1,2,3,4,10000000),

labels = c("<1","1-2","2-3","3-4",">4"),

include.lowest = T,right = T))

map$location[map$location == 'United States of America'] = 'USA'

map$location[map$location == 'Russian Federation'] = 'Russia'

map$location[map$location == 'United Kingdom'] = 'UK'

map$location[map$location == 'Congo'] = 'Republic of Congo'

map$location[map$location == "Iran (Islamic Republic of)"] = 'Iran'

map$location[map$location == "Democratic People's Republic of Korea"] = 'North Korea'

map$location[map$location == "Taiwan (Province of China)"] = 'Taiwan'

map$location[map$location == "Republic of Korea"] = 'South Korea'

map$location[map$location == "United Republic of Tanzania"] = 'Tanzania'

map$location[map$location == "Bolivia (Plurinational State of)"] = 'Bolivia'

map$location[map$location == "Venezuela (Bolivarian Republic of)"] = 'Venezuela'

map$location[map$location == "Czechia"] = 'Czech Republic'

map$location[map$location == "Republic of Moldova"] = 'Moldova'

map$location[map$location == "Viet Nam"] = 'Vietnam'

map$location[map$location == "Lao People's Democratic Republic"] = 'Laos'

map$location[map$location == "Syrian Arab Republic"] = 'Syria'

map$location[map$location == "North Macedonia"] = 'Macedonia'

map$location[map$location == "Micronesia (Federated States of)"] = 'Micronesia'

map$location[map$location == "Macedonia"] = 'North Macedonia'

map$location[map$location == "Trinidad and Tobago"] = 'Trinidad'

a <- map[map$location == "Trinidad",]

a$location <- 'Tobago'

map <- rbind(map,a)

map$location[map$location == "Cabo Verde"] = 'Cape Verde'

map$location[map$location == "United States Virgin Islands"] = 'Virgin Islands'

map$location[map$location == "Antigua and Barbuda"] = 'Antigu'

a <- map[map$location == "Antigu",]

a$location <- 'Barbuda'

map <- rbind(map,a)

map$location[map$location == "Saint Kitts and Nevis"] = 'Saint Kitts'

a <- map[map$location == "Saint Kitts",]

a$location <- 'Nevis'

map <- rbind(map,a)

map$location[map$location == "Côte d'Ivoire"] = 'Ivory Coast'

map$location[map$location == "Saint Vincent and the Grenadines"] = 'Saint Vincent'

a <- map[map$location == "Saint Vincent",]

a$location <- 'Grenadines'

map <- rbind(map,a)

map$location[map$location == "Eswatini"] = 'Swaziland'

map$location[map$location == "Brunei Darussalam"] = 'Brunei'

map <- full_join(world,map,by = c('region'='location')) %>%

filter(val != "NA")

mycolor2<-brewer.pal(5, "Blues")

fig <- map %>%

ggplot()+

geom_polygon(aes(x = long, y = lat,group = group,fill=val2),colour="white",

size=0.2) +

theme_void()+

scale_fill_manual(values=mycolor2) +

theme(legend.position = c(0.1,0.2),

legend.title = element_blank(),

legend.text = element_text(color="black",

size = 12,

),

plot.title = element_blank(),

panel.grid=element_blank(),

axis.title.x = element_blank(),

axis.text.x = element_blank(),

axis.ticks.x = element_blank(),

axis.title.y = element_blank(),

axis.text.y = element_blank(),

axis.ticks.y = element_blank(),

)

fig

IS1 <- IS %>%

filter(measure == "Incidence") %>%

filter(year == 2021) %>%

filter(sex == "Both") %>%

filter(age == "All ages") %>%

filter(metric == "Number")

summary(IS1$val)

quantile(IS1$val,seq(0.1,1,0.1))

map <- IS1%>% mutate(val2 = cut(val, breaks = c(0,1,2,3,4,10000000),

labels = c("<1","1-2","2-3","3-4",">4"),

include.lowest = T,right = T))

map$location[map$location == 'United States of America'] = 'USA'

map$location[map$location == 'Russian Federation'] = 'Russia'

map$location[map$location == 'United Kingdom'] = 'UK'

map$location[map$location == 'Congo'] = 'Republic of Congo'

map$location[map$location == "Iran (Islamic Republic of)"] = 'Iran'

map$location[map$location == "Democratic People's Republic of Korea"] = 'North Korea'

map$location[map$location == "Taiwan (Province of China)"] = 'Taiwan'

map$location[map$location == "Republic of Korea"] = 'South Korea'

map$location[map$location == "United Republic of Tanzania"] = 'Tanzania'

map$location[map$location == "Bolivia (Plurinational State of)"] = 'Bolivia'

map$location[map$location == "Venezuela (Bolivarian Republic of)"] = 'Venezuela'

map$location[map$location == "Czechia"] = 'Czech Republic'

map$location[map$location == "Republic of Moldova"] = 'Moldova'

map$location[map$location == "Viet Nam"] = 'Vietnam'

map$location[map$location == "Lao People's Democratic Republic"] = 'Laos'

map$location[map$location == "Syrian Arab Republic"] = 'Syria'

map$location[map$location == "North Macedonia"] = 'Macedonia'

map$location[map$location == "Micronesia (Federated States of)"] = 'Micronesia'

map$location[map$location == "Macedonia"] = 'North Macedonia'

map$location[map$location == "Trinidad and Tobago"] = 'Trinidad'

a <- map[map$location == "Trinidad",]

a$location <- 'Tobago'

map <- rbind(map,a)

map$location[map$location == "Cabo Verde"] = 'Cape Verde'

map$location[map$location == "United States Virgin Islands"] = 'Virgin Islands'

map$location[map$location == "Antigua and Barbuda"] = 'Antigu'

a <- map[map$location == "Antigu",]

a$location <- 'Barbuda'

map <- rbind(map,a)

map$location[map$location == "Saint Kitts and Nevis"] = 'Saint Kitts'

a <- map[map$location == "Saint Kitts",]

a$location <- 'Nevis'

map <- rbind(map,a)

map$location[map$location == "Côte d'Ivoire"] = 'Ivory Coast'

map$location[map$location == "Saint Vincent and the Grenadines"] = 'Saint Vincent'

a <- map[map$location == "Saint Vincent",]

a$location <- 'Grenadines'

map <- rbind(map,a)

map$location[map$location == "Eswatini"] = 'Swaziland'

map$location[map$location == "Brunei Darussalam"] = 'Brunei'

map <- full_join(world,map,by = c('region'='location')) %>%

filter(val != "NA")

mycolor2<-brewer.pal(5, "Blues")

fig <- map %>%

ggplot()+

geom_polygon(aes(x = long, y = lat,group = group,fill=val2),colour="white",

size=0.2) +

theme_void()+

scale_fill_manual(values=mycolor2) +

theme(legend.position = c(0.1,0.2),

legend.title = element_blank(),

legend.text = element_text(color="black",

size = 12,

),

plot.title = element_blank(),

panel.grid=element_blank(),

axis.title.x = element_blank(),

axis.text.x = element_blank(),

axis.ticks.x = element_blank(),

axis.title.y = element_blank(),

axis.text.y = element_blank(),

axis.ticks.y = element_blank(),

)

fig

IS1 <- IS %>%

filter(measure == "Prevalence") %>%

filter(year == 2021) %>%

filter(sex == "Both") %>%

filter(age == "All ages") %>%

filter(metric == "Number")

summary(IS1$val)

quantile(IS1$val,seq(0.1,1,0.1))

map <- IS1%>% mutate(val2 = cut(val, breaks = c(0,1,2,3,4,10000000),

labels = c("<1","1-2","2-3","3-4",">4"),

include.lowest = T,right = T))

map$location[map$location == 'United States of America'] = 'USA'

map$location[map$location == 'Russian Federation'] = 'Russia'

map$location[map$location == 'United Kingdom'] = 'UK'

map$location[map$location == 'Congo'] = 'Republic of Congo'

map$location[map$location == "Iran (Islamic Republic of)"] = 'Iran'

map$location[map$location == "Democratic People's Republic of Korea"] = 'North Korea'

map$location[map$location == "Taiwan (Province of China)"] = 'Taiwan'

map$location[map$location == "Republic of Korea"] = 'South Korea'

map$location[map$location == "United Republic of Tanzania"] = 'Tanzania'

map$location[map$location == "Bolivia (Plurinational State of)"] = 'Bolivia'

map$location[map$location == "Venezuela (Bolivarian Republic of)"] = 'Venezuela'

map$location[map$location == "Czechia"] = 'Czech Republic'

map$location[map$location == "Republic of Moldova"] = 'Moldova'

map$location[map$location == "Viet Nam"] = 'Vietnam'

map$location[map$location == "Lao People's Democratic Republic"] = 'Laos'

map$location[map$location == "Syrian Arab Republic"] = 'Syria'

map$location[map$location == "North Macedonia"] = 'Macedonia'

map$location[map$location == "Micronesia (Federated States of)"] = 'Micronesia'

map$location[map$location == "Macedonia"] = 'North Macedonia'

map$location[map$location == "Trinidad and Tobago"] = 'Trinidad'

a <- map[map$location == "Trinidad",]

a$location <- 'Tobago'

map <- rbind(map,a)

map$location[map$location == "Cabo Verde"] = 'Cape Verde'

map$location[map$location == "United States Virgin Islands"] = 'Virgin Islands'

map$location[map$location == "Antigua and Barbuda"] = 'Antigu'

a <- map[map$location == "Antigu",]

a$location <- 'Barbuda'

map <- rbind(map,a)

map$location[map$location == "Saint Kitts and Nevis"] = 'Saint Kitts'

a <- map[map$location == "Saint Kitts",]

a$location <- 'Nevis'

map <- rbind(map,a)

map$location[map$location == "Côte d'Ivoire"] = 'Ivory Coast'

map$location[map$location == "Saint Vincent and the Grenadines"] = 'Saint Vincent'

a <- map[map$location == "Saint Vincent",]

a$location <- 'Grenadines'

map <- rbind(map,a)

map$location[map$location == "Eswatini"] = 'Swaziland'

map$location[map$location == "Brunei Darussalam"] = 'Brunei'

map <- full_join(world,map,by = c('region'='location')) %>%

filter(val != "NA")

mycolor2<-brewer.pal(5, "Blues")

fig <- map %>%

ggplot()+

geom_polygon(aes(x = long, y = lat,group = group,fill=val2),colour="white",

size=0.2) +

theme_void()+

scale_fill_manual(values=mycolor2) +

theme(legend.position = c(0.1,0.2),

legend.title = element_blank(),

legend.text = element_text(color="black",

size = 12,

),

plot.title = element_blank(),

panel.grid=element_blank(),

axis.title.x = element_blank(),

axis.text.x = element_blank(),

axis.ticks.x = element_blank(),

axis.title.y = element_blank(),

axis.text.y = element_blank(),

axis.ticks.y = element_blank(),

)

fig

IS1 <- IS %>%

filter(measure == "Deaths") %>%

filter(year == 2021) %>%

filter(sex == "Both") %>%

filter(age == "All ages") %>%

filter(metric == "Number")

summary(IS1$val)

quantile(IS1$val,seq(0.1,1,0.1))

map <- IS1%>% mutate(val2 = cut(val, breaks = c(0,1,2,3,4,10000000),

labels = c("<1","1-2","2-3","3-4",">4"),

include.lowest = T,right = T))

map$location[map$location == 'United States of America'] = 'USA'

map$location[map$location == 'Russian Federation'] = 'Russia'

map$location[map$location == 'United Kingdom'] = 'UK'

map$location[map$location == 'Congo'] = 'Republic of Congo'

map$location[map$location == "Iran (Islamic Republic of)"] = 'Iran'

map$location[map$location == "Democratic People's Republic of Korea"] = 'North Korea'

map$location[map$location == "Taiwan (Province of China)"] = 'Taiwan'

map$location[map$location == "Republic of Korea"] = 'South Korea'

map$location[map$location == "United Republic of Tanzania"] = 'Tanzania'

map$location[map$location == "Bolivia (Plurinational State of)"] = 'Bolivia'

map$location[map$location == "Venezuela (Bolivarian Republic of)"] = 'Venezuela'

map$location[map$location == "Czechia"] = 'Czech Republic'

map$location[map$location == "Republic of Moldova"] = 'Moldova'

map$location[map$location == "Viet Nam"] = 'Vietnam'

map$location[map$location == "Lao People's Democratic Republic"] = 'Laos'

map$location[map$location == "Syrian Arab Republic"] = 'Syria'

map$location[map$location == "North Macedonia"] = 'Macedonia'

map$location[map$location == "Micronesia (Federated States of)"] = 'Micronesia'

map$location[map$location == "Macedonia"] = 'North Macedonia'

map$location[map$location == "Trinidad and Tobago"] = 'Trinidad'

a <- map[map$location == "Trinidad",]

a$location <- 'Tobago'

map <- rbind(map,a)

map$location[map$location == "Cabo Verde"] = 'Cape Verde'

map$location[map$location == "United States Virgin Islands"] = 'Virgin Islands'

map$location[map$location == "Antigua and Barbuda"] = 'Antigu'

a <- map[map$location == "Antigu",]

a$location <- 'Barbuda'

map <- rbind(map,a)

map$location[map$location == "Saint Kitts and Nevis"] = 'Saint Kitts'

a <- map[map$location == "Saint Kitts",]

a$location <- 'Nevis'

map <- rbind(map,a)

map$location[map$location == "Côte d'Ivoire"] = 'Ivory Coast'

map$location[map$location == "Saint Vincent and the Grenadines"] = 'Saint Vincent'

a <- map[map$location == "Saint Vincent",]

a$location <- 'Grenadines'

map <- rbind(map,a)

map$location[map$location == "Eswatini"] = 'Swaziland'

map$location[map$location == "Brunei Darussalam"] = 'Brunei'

map <- full_join(world,map,by = c('region'='location')) %>%

filter(val != "NA")

mycolor2<-brewer.pal(5, "Blues")

fig <- map %>%

ggplot()+

geom_polygon(aes(x = long, y = lat,group = group,fill=val2),colour="white",

size=0.2) +

theme_void()+

scale_fill_manual(values=mycolor2) +

theme(legend.position = c(0.1,0.2),

legend.title = element_blank(),

legend.text = element_text(color="black",

size = 12,

),

plot.title = element_blank(),

panel.grid=element_blank(),

axis.title.x = element_blank(),

axis.text.x = element_blank(),

axis.ticks.x = element_blank(),

axis.title.y = element_blank(),

axis.text.y = element_blank(),

axis.ticks.y = element_blank(),

)

fig

IS1 <- IS %>%

filter(measure == "DALYs") %>%

filter(year == 2021) %>%

filter(sex == "Both") %>%

filter(age == "All ages") %>%

filter(metric == "Number")

summary(IS1$val)

quantile(IS1$val,seq(0.1,1,0.1))

map <- IS1%>% mutate(val2 = cut(val, breaks = c(0,1,2,3,4,10000000),

labels = c("<1","1-2","2-3","3-4",">4"),

include.lowest = T,right = T))

map$location[map$location == 'United States of America'] = 'USA'

map$location[map$location == 'Russian Federation'] = 'Russia'

map$location[map$location == 'United Kingdom'] = 'UK'

map$location[map$location == 'Congo'] = 'Republic of Congo'

map$location[map$location == "Iran (Islamic Republic of)"] = 'Iran'

map$location[map$location == "Democratic People's Republic of Korea"] = 'North Korea'

map$location[map$location == "Taiwan (Province of China)"] = 'Taiwan'

map$location[map$location == "Republic of Korea"] = 'South Korea'

map$location[map$location == "United Republic of Tanzania"] = 'Tanzania'

map$location[map$location == "Bolivia (Plurinational State of)"] = 'Bolivia'

map$location[map$location == "Venezuela (Bolivarian Republic of)"] = 'Venezuela'

map$location[map$location == "Czechia"] = 'Czech Republic'

map$location[map$location == "Republic of Moldova"] = 'Moldova'

map$location[map$location == "Viet Nam"] = 'Vietnam'

map$location[map$location == "Lao People's Democratic Republic"] = 'Laos'

map$location[map$location == "Syrian Arab Republic"] = 'Syria'

map$location[map$location == "North Macedonia"] = 'Macedonia'

map$location[map$location == "Micronesia (Federated States of)"] = 'Micronesia'

map$location[map$location == "Macedonia"] = 'North Macedonia'

map$location[map$location == "Trinidad and Tobago"] = 'Trinidad'

a <- map[map$location == "Trinidad",]

a$location <- 'Tobago'

map <- rbind(map,a)

map$location[map$location == "Cabo Verde"] = 'Cape Verde'

map$location[map$location == "United States Virgin Islands"] = 'Virgin Islands'

map$location[map$location == "Antigua and Barbuda"] = 'Antigu'

a <- map[map$location == "Antigu",]

a$location <- 'Barbuda'

map <- rbind(map,a)

map$location[map$location == "Saint Kitts and Nevis"] = 'Saint Kitts'

a <- map[map$location == "Saint Kitts",]

a$location <- 'Nevis'

map <- rbind(map,a)

map$location[map$location == "Côte d'Ivoire"] = 'Ivory Coast'

map$location[map$location == "Saint Vincent and the Grenadines"] = 'Saint Vincent'

a <- map[map$location == "Saint Vincent",]

a$location <- 'Grenadines'

map <- rbind(map,a)

map$location[map$location == "Eswatini"] = 'Swaziland'

map$location[map$location == "Brunei Darussalam"] = 'Brunei'

map <- full_join(world,map,by = c('region'='location')) %>%

filter(val != "NA")

mycolor2<-brewer.pal(5, "Blues")

fig <- map %>%

ggplot()+

geom_polygon(aes(x = long, y = lat,group = group,fill=val2),colour="white",

size=0.2) +

theme_void()+

scale_fill_manual(values=mycolor2) +

theme(legend.position = c(0.1,0.2),

legend.title = element_blank(),

legend.text = element_text(color="black",

size = 12,

),

plot.title = element_blank(),

panel.grid=element_blank(),

axis.title.x = element_blank(),

axis.text.x = element_blank(),

axis.ticks.x = element_blank(),

axis.title.y = element_blank(),

axis.text.y = element_blank(),

axis.ticks.y = element_blank(),

)

fig

#Figure 4-5; Figure S13

library(dplyr)

library(ggplot2)

IS <- HL_region %>%

dplyr::select(measure_name,location_name,metric_name,

sex_name,age_name,year,val,lower,upper) %>%

rename(measure=measure_name,

location=location_name,

metric=metric_name,

sex=sex_name,

age=age_name)

Incidence_age <- IS %>%

filter(measure == "Incidence" &

sex != "Both" &

age != "All ages" &

age != "Age-standardized" &

year == 2021) %>%

filter(location=="Global" |

location=="High SDI" |

location=="High-middle SDI" |

location=="Middle SDI" |

location=="Low-middle SDI" |

location=="Low SDI") %>%

mutate(val=ifelse(sex=="Male",val,-val),

lower=ifelse(sex=="Male",lower,-lower),

upper=ifelse(sex=="Male",upper,-upper)) %>%

mutate(group=ifelse(location=="High SDI"&sex=="Male","a",ifelse(

location=="High-middle SDI"&sex=="Male","b",ifelse(

location=="Middle SDI"&sex=="Male","c",ifelse(

location=="Low-middle SDI"&sex=="Male","d",ifelse(

location=="Low SDI"&sex=="Male","e",ifelse(

location=="High SDI"&sex=="Female","f",ifelse(

location=="High-middle SDI"&sex=="Female","g",ifelse(

location=="Middle SDI"&sex=="Female","h",ifelse(

location=="Low-middle SDI"&sex=="Female","i","j")))))))))) %>%

mutate(age_new=ifelse(age=="<5 years",1,ifelse(

age=="5-9 years",2,ifelse(

age=="10-14 years",3,ifelse(

age=="15-19 years",4,ifelse(

age=="20-24 years",5,ifelse(

age=="25-29 years",6,ifelse(

age=="30-34 years",7,ifelse(

age=="35-39 years",8,ifelse(

age=="40-44 years",9,ifelse(

age=="45-49 years",10,ifelse(

age=="50-54 years",11,ifelse(

age=="55-59 years",12,ifelse(

age=="60-64 years",13,ifelse(

age=="65-69 years",14,ifelse(

age=="70-74 years",15,ifelse(

age=="75-79 years",16,ifelse(

age=="80-84 years",17,ifelse(

age=="85-89 years",18,ifelse(

age=="90-94 years",19,20)))))))))))))))))))) %>%

dplyr::select(,c(2,3,4,5,7:11))

ad_factor <- max(Incidence_age$val[Incidence_age$metric=="Rate"&Incidence_age$location=="Global"])/

max(Incidence_age$val[Incidence_age$metric=="Number"&Incidence_age$location!="Global"])

p <- ggplot() +

geom_col(data = subset(Incidence_age,metric=='Number'&location!="Global"),

aes(x = age_new, y = val, fill = group)) +

geom_line(data = subset(Incidence_age,metric=='Rate'&location=="Global"),

aes(x = age_new, y = val/ad_factor, color = sex, group = sex), size = 1) +

geom_ribbon(data = subset(Incidence_age,metric=='Rate'&location=="Global"),

aes(x = age_new, ymin = lower/ad_factor, ymax = upper/ad_factor, fill = sex), alpha = 0.2) +

scale_fill_manual(name="Gender, SDI region (Number)",

values = c("a" = "#338a57",

"b" = "#5bb577",

"c" = "#90d191",

"d" = "#c9e9c2",

"e" = "#f1f9ec",

"f" = "#ca3352",

"g" = "#f3624d",

"h" = "#fda364",

"i" = "#fed67e",

"j" = "#ffffc1"),

labels=c(

"a"="Male,High SDI",

'b'='Male,High-middle SDI',

'c'='Male,Middle SDI',

'd'='Male,Low-middle SDI',

'e'='Male,Low SDI',

'f'='Female,High SDI',

'g'='Female,High-middle SDI',

'h'='Female,Middle SDI',

'i'='Female,Low-middle SDI',

'j'='Female,Low SDI'

)) +

scale_color_manual(name="Gender, Global Rate (per 100,000)",

values = c("Male" = "#006d2b", "Female" = "#31a255"),

labels = c(

'Male'='Male,Global',

'Female'='Female,Global'

)) +

scale_x_continuous(breaks = 1:20,

labels=c('<5 years','5-9 years','10-14 years','15-19 years','20-24 years',

'25-29 years','30-34 years','35-39 years','40-44 years','45-49 years',

'50-54 years','55-59 years','60-64 years','65-69 years','70-74 years',

'75-79 years','80-84 years','85-89 years','90-94 years','95+ years')) +

scale_y_continuous(sec.axis = sec_axis(~.*ad_factor, name = "Gender, Global Rate (per 100,000)",

labels = function(x) format(abs(x), scientific = FALSE)),

labels = function(x) format(abs(x), scientific = FALSE)) +

theme_bw() +

theme(legend.position = "right",

panel.grid.major = element_line(color = "gray90"),

panel.grid.minor = element_line(color = "gray95"),

axis.text.x = element_text(angle = 45, hjust = 1)) +

labs(title = "",

x = 'Age',

y = 'Number')

p

Prevalence_age <- IS %>%

filter(measure == "Prevalence" &

sex != "Both" &

age != "All ages" &

age != "Age-standardized" &

year == 2021) %>%

filter(location=="Global" |

location=="High SDI" |

location=="High-middle SDI" |

location=="Middle SDI" |

location=="Low-middle SDI" |

location=="Low SDI") %>%

mutate(val=ifelse(sex=="Male",val,-val),

lower=ifelse(sex=="Male",lower,-lower),

upper=ifelse(sex=="Male",upper,-upper)) %>%

mutate(group=ifelse(location=="High SDI"&sex=="Male","a",ifelse(

location=="High-middle SDI"&sex=="Male","b",ifelse(

location=="Middle SDI"&sex=="Male","c",ifelse(

location=="Low-middle SDI"&sex=="Male","d",ifelse(

location=="Low SDI"&sex=="Male","e",ifelse(

location=="High SDI"&sex=="Female","f",ifelse(

location=="High-middle SDI"&sex=="Female","g",ifelse(

location=="Middle SDI"&sex=="Female","h",ifelse(

location=="Low-middle SDI"&sex=="Female","i","j")))))))))) %>%

mutate(age_new=ifelse(age=="<5 years",1,ifelse(

age=="5-9 years",2,ifelse(

age=="10-14 years",3,ifelse(

age=="15-19 years",4,ifelse(

age=="20-24 years",5,ifelse(

age=="25-29 years",6,ifelse(

age=="30-34 years",7,ifelse(

age=="35-39 years",8,ifelse(

age=="40-44 years",9,ifelse(

age=="45-49 years",10,ifelse(

age=="50-54 years",11,ifelse(

age=="55-59 years",12,ifelse(

age=="60-64 years",13,ifelse(

age=="65-69 years",14,ifelse(

age=="70-74 years",15,ifelse(

age=="75-79 years",16,ifelse(

age=="80-84 years",17,ifelse(

age=="85-89 years",18,ifelse(

age=="90-94 years",19,20)))))))))))))))))))) %>%

dplyr::select(,c(2,3,4,5,7:11))

ad_factor <- max(Incidence_age$val[Incidence_age$metric=="Rate"&Incidence_age$location=="Global"])/

max(Incidence_age$val[Incidence_age$metric=="Number"&Incidence_age$location!="Global"])

p <- ggplot() +

geom_col(data = subset(Incidence_age,metric=='Number'&location!="Global"),

aes(x = age_new, y = val, fill = group)) +

geom_line(data = subset(Incidence_age,metric=='Rate'&location=="Global"),

aes(x = age_new, y = val/ad_factor, color = sex, group = sex), size = 1) +

geom_ribbon(data = subset(Incidence_age,metric=='Rate'&location=="Global"),

aes(x = age_new, ymin = lower/ad_factor, ymax = upper/ad_factor, fill = sex), alpha = 0.2) +

scale_fill_manual(name="Gender, SDI region (Number)",

values = c("a" = "#338a57",

"b" = "#5bb577",

"c" = "#90d191",

"d" = "#c9e9c2",

"e" = "#f1f9ec",

"f" = "#ca3352",

"g" = "#f3624d",

"h" = "#fda364",

"i" = "#fed67e",

"j" = "#ffffc1"),

labels=c(

"a"="Male,High SDI",

'b'='Male,High-middle SDI',

'c'='Male,Middle SDI',

'd'='Male,Low-middle SDI',

'e'='Male,Low SDI',

'f'='Female,High SDI',

'g'='Female,High-middle SDI',

'h'='Female,Middle SDI',

'i'='Female,Low-middle SDI',

'j'='Female,Low SDI'

)) +

scale_color_manual(name="Gender, Global Rate (per 100,000)",

values = c("Male" = "#006d2b", "Female" = "#31a255"),

labels = c(

'Male'='Male,Global',

'Female'='Female,Global'

)) +

scale_x_continuous(breaks = 1:20,

labels=c('<5 years','5-9 years','10-14 years','15-19 years','20-24 years',

'25-29 years','30-34 years','35-39 years','40-44 years','45-49 years',

'50-54 years','55-59 years','60-64 years','65-69 years','70-74 years',

'75-79 years','80-84 years','85-89 years','90-94 years','95+ years')) +

scale_y_continuous(sec.axis = sec_axis(~.*ad_factor, name = "Gender, Global Rate (per 100,000)",

labels = function(x) format(abs(x), scientific = FALSE)),

labels = function(x) format(abs(x), scientific = FALSE)) +

theme_bw() +

theme(legend.position = "right",

panel.grid.major = element_line(color = "gray90"),

panel.grid.minor = element_line(color = "gray95"),

axis.text.x = element_text(angle = 45, hjust = 1)) +

labs(title = "",

x = 'Age',

y = 'Number')

p

Deaths_age <- IS %>%

filter(measure == "Deaths" &

sex != "Both" &

age != "All ages" &

age != "Age-standardized" &

year == 2021) %>%

filter(location=="Global" |

location=="High SDI" |

location=="High-middle SDI" |

location=="Middle SDI" |

location=="Low-middle SDI" |

location=="Low SDI") %>%

mutate(val=ifelse(sex=="Male",val,-val),

lower=ifelse(sex=="Male",lower,-lower),

upper=ifelse(sex=="Male",upper,-upper)) %>%

mutate(group=ifelse(location=="High SDI"&sex=="Male","a",ifelse(

location=="High-middle SDI"&sex=="Male","b",ifelse(

location=="Middle SDI"&sex=="Male","c",ifelse(

location=="Low-middle SDI"&sex=="Male","d",ifelse(

location=="Low SDI"&sex=="Male","e",ifelse(

location=="High SDI"&sex=="Female","f",ifelse(

location=="High-middle SDI"&sex=="Female","g",ifelse(

location=="Middle SDI"&sex=="Female","h",ifelse(

location=="Low-middle SDI"&sex=="Female","i","j")))))))))) %>%

mutate(age_new=ifelse(age=="<5 years",1,ifelse(

age=="5-9 years",2,ifelse(

age=="10-14 years",3,ifelse(

age=="15-19 years",4,ifelse(

age=="20-24 years",5,ifelse(

age=="25-29 years",6,ifelse(

age=="30-34 years",7,ifelse(

age=="35-39 years",8,ifelse(

age=="40-44 years",9,ifelse(

age=="45-49 years",10,ifelse(

age=="50-54 years",11,ifelse(

age=="55-59 years",12,ifelse(

age=="60-64 years",13,ifelse(

age=="65-69 years",14,ifelse(

age=="70-74 years",15,ifelse(

age=="75-79 years",16,ifelse(

age=="80-84 years",17,ifelse(

age=="85-89 years",18,ifelse(

age=="90-94 years",19,20)))))))))))))))))))) %>%

dplyr::select(,c(2,3,4,5,7:11))

ad_factor <- max(Incidence_age$val[Incidence_age$metric=="Rate"&Incidence_age$location=="Global"])/

max(Incidence_age$val[Incidence_age$metric=="Number"&Incidence_age$location!="Global"])

p <- ggplot() +

geom_col(data = subset(Incidence_age,metric=='Number'&location!="Global"),

aes(x = age_new, y = val, fill = group)) +

geom_line(data = subset(Incidence_age,metric=='Rate'&location=="Global"),

aes(x = age_new, y = val/ad_factor, color = sex, group = sex), size = 1) +

geom_ribbon(data = subset(Incidence_age,metric=='Rate'&location=="Global"),

aes(x = age_new, ymin = lower/ad_factor, ymax = upper/ad_factor, fill = sex), alpha = 0.2) +

scale_fill_manual(name="Gender, SDI region (Number)",

values = c("a" = "#338a57",

"b" = "#5bb577",

"c" = "#90d191",

"d" = "#c9e9c2",

"e" = "#f1f9ec",

"f" = "#ca3352",

"g" = "#f3624d",

"h" = "#fda364",

"i" = "#fed67e",

"j" = "#ffffc1"),

labels=c(

"a"="Male,High SDI",

'b'='Male,High-middle SDI',

'c'='Male,Middle SDI',

'd'='Male,Low-middle SDI',

'e'='Male,Low SDI',

'f'='Female,High SDI',

'g'='Female,High-middle SDI',

'h'='Female,Middle SDI',

'i'='Female,Low-middle SDI',

'j'='Female,Low SDI'

)) +

scale_color_manual(name="Gender, Global Rate (per 100,000)",

values = c("Male" = "#006d2b", "Female" = "#31a255"),

labels = c(

'Male'='Male,Global',

'Female'='Female,Global'

)) +

scale_x_continuous(breaks = 1:20,

labels=c('<5 years','5-9 years','10-14 years','15-19 years','20-24 years',

'25-29 years','30-34 years','35-39 years','40-44 years','45-49 years',

'50-54 years','55-59 years','60-64 years','65-69 years','70-74 years',

'75-79 years','80-84 years','85-89 years','90-94 years','95+ years')) +

scale_y_continuous(sec.axis = sec_axis(~.*ad_factor, name = "Gender, Global Rate (per 100,000)",

labels = function(x) format(abs(x), scientific = FALSE)),

labels = function(x) format(abs(x), scientific = FALSE)) +

theme_bw() +

theme(legend.position = "right",

panel.grid.major = element_line(color = "gray90"),

panel.grid.minor = element_line(color = "gray95"),

axis.text.x = element_text(angle = 45, hjust = 1)) +

labs(title = "",

x = 'Age',

y = 'Number')

p

DALY_age <- IS %>%

filter(measure == "DALYs (Disability-Adjusted Life Years)" &

sex != "Both" &

age != "All ages" &

age != "Age-standardized" &

year == 2021) %>%

filter(location=="Global" |

location=="High SDI" |

location=="High-middle SDI" |

location=="Middle SDI" |

location=="Low-middle SDI" |

location=="Low SDI") %>%

mutate(val=ifelse(sex=="Male",val,-val),

lower=ifelse(sex=="Male",lower,-lower),

upper=ifelse(sex=="Male",upper,-upper)) %>%

mutate(group=ifelse(location=="High SDI"&sex=="Male","a",ifelse(

location=="High-middle SDI"&sex=="Male","b",ifelse(

location=="Middle SDI"&sex=="Male","c",ifelse(

location=="Low-middle SDI"&sex=="Male","d",ifelse(

location=="Low SDI"&sex=="Male","e",ifelse(

location=="High SDI"&sex=="Female","f",ifelse(

location=="High-middle SDI"&sex=="Female","g",ifelse(

location=="Middle SDI"&sex=="Female","h",ifelse(

location=="Low-middle SDI"&sex=="Female","i","j")))))))))) %>%

mutate(age_new=ifelse(age=="<5 years",1,ifelse(

age=="5-9 years",2,ifelse(

age=="10-14 years",3,ifelse(

age=="15-19 years",4,ifelse(

age=="20-24 years",5,ifelse(

age=="25-29 years",6,ifelse(

age=="30-34 years",7,ifelse(

age=="35-39 years",8,ifelse(

age=="40-44 years",9,ifelse(

age=="45-49 years",10,ifelse(

age=="50-54 years",11,ifelse(

age=="55-59 years",12,ifelse(

age=="60-64 years",13,ifelse(

age=="65-69 years",14,ifelse(

age=="70-74 years",15,ifelse(

age=="75-79 years",16,ifelse(

age=="80-84 years",17,ifelse(

age=="85-89 years",18,ifelse(

age=="90-94 years",19,20)))))))))))))))))))) %>%

dplyr::select(,c(2,3,4,5,7:11))

ad_factor <- max(Incidence_age$val[Incidence_age$metric=="Rate"&Incidence_age$location=="Global"])/

max(Incidence_age$val[Incidence_age$metric=="Number"&Incidence_age$location!="Global"])

p <- ggplot() +

geom_col(data = subset(Incidence_age,metric=='Number'&location!="Global"),

aes(x = age_new, y = val, fill = group)) +

geom_line(data = subset(Incidence_age,metric=='Rate'&location=="Global"),

aes(x = age_new, y = val/ad_factor, color = sex, group = sex), size = 1) +

geom_ribbon(data = subset(Incidence_age,metric=='Rate'&location=="Global"),

aes(x = age_new, ymin = lower/ad_factor, ymax = upper/ad_factor, fill = sex), alpha = 0.2) +

scale_fill_manual(name="Gender, SDI region (Number)",

values = c("a" = "#338a57",

"b" = "#5bb577",

"c" = "#90d191",

"d" = "#c9e9c2",

"e" = "#f1f9ec",

"f" = "#ca3352",

"g" = "#f3624d",

"h" = "#fda364",

"i" = "#fed67e",

"j" = "#ffffc1"),

labels=c(

"a"="Male,High SDI",

'b'='Male,High-middle SDI',

'c'='Male,Middle SDI',

'd'='Male,Low-middle SDI',

'e'='Male,Low SDI',

'f'='Female,High SDI',

'g'='Female,High-middle SDI',

'h'='Female,Middle SDI',

'i'='Female,Low-middle SDI',

'j'='Female,Low SDI'

)) +

scale_color_manual(name="Gender, Global Rate (per 100,000)",

values = c("Male" = "#006d2b", "Female" = "#31a255"),

labels = c(

'Male'='Male,Global',

'Female'='Female,Global'

)) +

scale_x_continuous(breaks = 1:20,

labels=c('<5 years','5-9 years','10-14 years','15-19 years','20-24 years',

'25-29 years','30-34 years','35-39 years','40-44 years','45-49 years',

'50-54 years','55-59 years','60-64 years','65-69 years','70-74 years',

'75-79 years','80-84 years','85-89 years','90-94 years','95+ years')) +

scale_y_continuous(sec.axis = sec_axis(~.*ad_factor, name = "Gender, Global Rate (per 100,000)",

labels = function(x) format(abs(x), scientific = FALSE)),

labels = function(x) format(abs(x), scientific = FALSE)) +

theme_bw() +

theme(legend.position = "right",

panel.grid.major = element_line(color = "gray90"),

panel.grid.minor = element_line(color = "gray95"),

axis.text.x = element_text(angle = 45, hjust = 1)) +

labs(title = "",

x = 'Age',

y = 'Number')

p

IS <- NHL_region %>%

dplyr::select(measure_name,location_name,metric_name,

sex_name,age_name,year,val,lower,upper) %>%

rename(measure=measure_name,

location=location_name,

metric=metric_name,

sex=sex_name,

age=age_name)

Incidence_age <- IS %>%

filter(measure == "Incidence" &

sex != "Both" &

age != "All ages" &

age != "Age-standardized" &

year == 2021) %>%

filter(location=="Global" |

location=="High SDI" |

location=="High-middle SDI" |

location=="Middle SDI" |

location=="Low-middle SDI" |

location=="Low SDI") %>%

mutate(val=ifelse(sex=="Male",val,-val),

lower=ifelse(sex=="Male",lower,-lower),

upper=ifelse(sex=="Male",upper,-upper)) %>%

mutate(group=ifelse(location=="High SDI"&sex=="Male","a",ifelse(

location=="High-middle SDI"&sex=="Male","b",ifelse(

location=="Middle SDI"&sex=="Male","c",ifelse(

location=="Low-middle SDI"&sex=="Male","d",ifelse(

location=="Low SDI"&sex=="Male","e",ifelse(

location=="High SDI"&sex=="Female","f",ifelse(

location=="High-middle SDI"&sex=="Female","g",ifelse(

location=="Middle SDI"&sex=="Female","h",ifelse(

location=="Low-middle SDI"&sex=="Female","i","j")))))))))) %>%

mutate(age_new=ifelse(age=="<5 years",1,ifelse(

age=="5-9 years",2,ifelse(

age=="10-14 years",3,ifelse(

age=="15-19 years",4,ifelse(

age=="20-24 years",5,ifelse(

age=="25-29 years",6,ifelse(

age=="30-34 years",7,ifelse(

age=="35-39 years",8,ifelse(

age=="40-44 years",9,ifelse(

age=="45-49 years",10,ifelse(

age=="50-54 years",11,ifelse(

age=="55-59 years",12,ifelse(

age=="60-64 years",13,ifelse(

age=="65-69 years",14,ifelse(

age=="70-74 years",15,ifelse(

age=="75-79 years",16,ifelse(

age=="80-84 years",17,ifelse(

age=="85-89 years",18,ifelse(

age=="90-94 years",19,20)))))))))))))))))))) %>%

dplyr::select(,c(2,3,4,5,7:11))

ad_factor <- max(Incidence_age$val[Incidence_age$metric=="Rate"&Incidence_age$location=="Global"])/

max(Incidence_age$val[Incidence_age$metric=="Number"&Incidence_age$location!="Global"])

p <- ggplot() +

geom_col(data = subset(Incidence_age,metric=='Number'&location!="Global"),

aes(x = age_new, y = val, fill = group)) +

geom_line(data = subset(Incidence_age,metric=='Rate'&location=="Global"),

aes(x = age_new, y = val/ad_factor, color = sex, group = sex), size = 1) +

geom_ribbon(data = subset(Incidence_age,metric=='Rate'&location=="Global"),

aes(x = age_new, ymin = lower/ad_factor, ymax = upper/ad_factor, fill = sex), alpha = 0.2) +

scale_fill_manual(name="Gender, SDI region (Number)",

values = c("a" = "#338a57",

"b" = "#5bb577",

"c" = "#90d191",

"d" = "#c9e9c2",

"e" = "#f1f9ec",

"f" = "#ca3352",

"g" = "#f3624d",

"h" = "#fda364",

"i" = "#fed67e",

"j" = "#ffffc1"),

labels=c(

"a"="Male,High SDI",

'b'='Male,High-middle SDI',

'c'='Male,Middle SDI',

'd'='Male,Low-middle SDI',

'e'='Male,Low SDI',

'f'='Female,High SDI',

'g'='Female,High-middle SDI',

'h'='Female,Middle SDI',

'i'='Female,Low-middle SDI',

'j'='Female,Low SDI'

)) +

scale_color_manual(name="Gender, Global Rate (per 100,000)",

values = c("Male" = "#006d2b", "Female" = "#31a255"),

labels = c(

'Male'='Male,Global',

'Female'='Female,Global'

)) +

scale_x_continuous(breaks = 1:20,

labels=c('<5 years','5-9 years','10-14 years','15-19 years','20-24 years',

'25-29 years','30-34 years','35-39 years','40-44 years','45-49 years',

'50-54 years','55-59 years','60-64 years','65-69 years','70-74 years',

'75-79 years','80-84 years','85-89 years','90-94 years','95+ years')) +

scale_y_continuous(sec.axis = sec_axis(~.*ad_factor, name = "Gender, Global Rate (per 100,000)",

labels = function(x) format(abs(x), scientific = FALSE)),

labels = function(x) format(abs(x), scientific = FALSE)) +

theme_bw() +

theme(legend.position = "right",

panel.grid.major = element_line(color = "gray90"),

panel.grid.minor = element_line(color = "gray95"),

axis.text.x = element_text(angle = 45, hjust = 1)) +

labs(title = "",

x = 'Age',

y = 'Number')

p

Prevalence_age <- IS %>%

filter(measure == "Prevalence" &

sex != "Both" &

age != "All ages" &

age != "Age-standardized" &

year == 2021) %>%

filter(location=="Global" |

location=="High SDI" |

location=="High-middle SDI" |

location=="Middle SDI" |

location=="Low-middle SDI" |

location=="Low SDI") %>%

mutate(val=ifelse(sex=="Male",val,-val),

lower=ifelse(sex=="Male",lower,-lower),

upper=ifelse(sex=="Male",upper,-upper)) %>%

mutate(group=ifelse(location=="High SDI"&sex=="Male","a",ifelse(

location=="High-middle SDI"&sex=="Male","b",ifelse(

location=="Middle SDI"&sex=="Male","c",ifelse(

location=="Low-middle SDI"&sex=="Male","d",ifelse(

location=="Low SDI"&sex=="Male","e",ifelse(

location=="High SDI"&sex=="Female","f",ifelse(

location=="High-middle SDI"&sex=="Female","g",ifelse(

location=="Middle SDI"&sex=="Female","h",ifelse(

location=="Low-middle SDI"&sex=="Female","i","j")))))))))) %>%

mutate(age_new=ifelse(age=="<5 years",1,ifelse(

age=="5-9 years",2,ifelse(

age=="10-14 years",3,ifelse(

age=="15-19 years",4,ifelse(

age=="20-24 years",5,ifelse(

age=="25-29 years",6,ifelse(

age=="30-34 years",7,ifelse(

age=="35-39 years",8,ifelse(

age=="40-44 years",9,ifelse(

age=="45-49 years",10,ifelse(

age=="50-54 years",11,ifelse(

age=="55-59 years",12,ifelse(

age=="60-64 years",13,ifelse(

age=="65-69 years",14,ifelse(

age=="70-74 years",15,ifelse(

age=="75-79 years",16,ifelse(

age=="80-84 years",17,ifelse(

age=="85-89 years",18,ifelse(

age=="90-94 years",19,20)))))))))))))))))))) %>%

dplyr::select(,c(2,3,4,5,7:11))

ad_factor <- max(Incidence_age$val[Incidence_age$metric=="Rate"&Incidence_age$location=="Global"])/

max(Incidence_age$val[Incidence_age$metric=="Number"&Incidence_age$location!="Global"])

p <- ggplot() +

geom_col(data = subset(Incidence_age,metric=='Number'&location!="Global"),

aes(x = age_new, y = val, fill = group)) +

geom_line(data = subset(Incidence_age,metric=='Rate'&location=="Global"),

aes(x = age_new, y = val/ad_factor, color = sex, group = sex), size = 1) +

geom_ribbon(data = subset(Incidence_age,metric=='Rate'&location=="Global"),

aes(x = age_new, ymin = lower/ad_factor, ymax = upper/ad_factor, fill = sex), alpha = 0.2) +

scale_fill_manual(name="Gender, SDI region (Number)",

values = c("a" = "#338a57",

"b" = "#5bb577",

"c" = "#90d191",

"d" = "#c9e9c2",

"e" = "#f1f9ec",

"f" = "#ca3352",

"g" = "#f3624d",

"h" = "#fda364",

"i" = "#fed67e",

"j" = "#ffffc1"),

labels=c(

"a"="Male,High SDI",

'b'='Male,High-middle SDI',

'c'='Male,Middle SDI',

'd'='Male,Low-middle SDI',

'e'='Male,Low SDI',

'f'='Female,High SDI',

'g'='Female,High-middle SDI',

'h'='Female,Middle SDI',

'i'='Female,Low-middle SDI',

'j'='Female,Low SDI'

)) +

scale_color_manual(name="Gender, Global Rate (per 100,000)",

values = c("Male" = "#006d2b", "Female" = "#31a255"),

labels = c(

'Male'='Male,Global',

'Female'='Female,Global'

)) +

scale_x_continuous(breaks = 1:20,

labels=c('<5 years','5-9 years','10-14 years','15-19 years','20-24 years',

'25-29 years','30-34 years','35-39 years','40-44 years','45-49 years',

'50-54 years','55-59 years','60-64 years','65-69 years','70-74 years',

'75-79 years','80-84 years','85-89 years','90-94 years','95+ years')) +

scale_y_continuous(sec.axis = sec_axis(~.*ad_factor, name = "Gender, Global Rate (per 100,000)",

labels = function(x) format(abs(x), scientific = FALSE)),

labels = function(x) format(abs(x), scientific = FALSE)) +

theme_bw() +

theme(legend.position = "right",

panel.grid.major = element_line(color = "gray90"),

panel.grid.minor = element_line(color = "gray95"),

axis.text.x = element_text(angle = 45, hjust = 1)) +

labs(title = "",

x = 'Age',

y = 'Number')

p

Deaths_age <- IS %>%

filter(measure == "Deaths" &

sex != "Both" &

age != "All ages" &

age != "Age-standardized" &

year == 2021) %>%

filter(location=="Global" |

location=="High SDI" |

location=="High-middle SDI" |

location=="Middle SDI" |

location=="Low-middle SDI" |

location=="Low SDI") %>%

mutate(val=ifelse(sex=="Male",val,-val),

lower=ifelse(sex=="Male",lower,-lower),

upper=ifelse(sex=="Male",upper,-upper)) %>%

mutate(group=ifelse(location=="High SDI"&sex=="Male","a",ifelse(

location=="High-middle SDI"&sex=="Male","b",ifelse(

location=="Middle SDI"&sex=="Male","c",ifelse(

location=="Low-middle SDI"&sex=="Male","d",ifelse(

location=="Low SDI"&sex=="Male","e",ifelse(

location=="High SDI"&sex=="Female","f",ifelse(

location=="High-middle SDI"&sex=="Female","g",ifelse(

location=="Middle SDI"&sex=="Female","h",ifelse(

location=="Low-middle SDI"&sex=="Female","i","j")))))))))) %>%

mutate(age_new=ifelse(age=="<5 years",1,ifelse(

age=="5-9 years",2,ifelse(

age=="10-14 years",3,ifelse(

age=="15-19 years",4,ifelse(

age=="20-24 years",5,ifelse(

age=="25-29 years",6,ifelse(

age=="30-34 years",7,ifelse(

age=="35-39 years",8,ifelse(

age=="40-44 years",9,ifelse(

age=="45-49 years",10,ifelse(

age=="50-54 years",11,ifelse(

age=="55-59 years",12,ifelse(

age=="60-64 years",13,ifelse(

age=="65-69 years",14,ifelse(

age=="70-74 years",15,ifelse(

age=="75-79 years",16,ifelse(

age=="80-84 years",17,ifelse(

age=="85-89 years",18,ifelse(

age=="90-94 years",19,20)))))))))))))))))))) %>%

dplyr::select(,c(2,3,4,5,7:11))

ad_factor <- max(Incidence_age$val[Incidence_age$metric=="Rate"&Incidence_age$location=="Global"])/

max(Incidence_age$val[Incidence_age$metric=="Number"&Incidence_age$location!="Global"])

p <- ggplot() +

geom_col(data = subset(Incidence_age,metric=='Number'&location!="Global"),

aes(x = age_new, y = val, fill = group)) +

geom_line(data = subset(Incidence_age,metric=='Rate'&location=="Global"),

aes(x = age_new, y = val/ad_factor, color = sex, group = sex), size = 1) +

geom_ribbon(data = subset(Incidence_age,metric=='Rate'&location=="Global"),

aes(x = age_new, ymin = lower/ad_factor, ymax = upper/ad_factor, fill = sex), alpha = 0.2) +

scale_fill_manual(name="Gender, SDI region (Number)",

values = c("a" = "#338a57",

"b" = "#5bb577",

"c" = "#90d191",

"d" = "#c9e9c2",

"e" = "#f1f9ec",

"f" = "#ca3352",

"g" = "#f3624d",

"h" = "#fda364",

"i" = "#fed67e",

"j" = "#ffffc1"),

labels=c(

"a"="Male,High SDI",

'b'='Male,High-middle SDI',

'c'='Male,Middle SDI',

'd'='Male,Low-middle SDI',

'e'='Male,Low SDI',

'f'='Female,High SDI',

'g'='Female,High-middle SDI',

'h'='Female,Middle SDI',

'i'='Female,Low-middle SDI',

'j'='Female,Low SDI'

)) +

scale_color_manual(name="Gender, Global Rate (per 100,000)",

values = c("Male" = "#006d2b", "Female" = "#31a255"),

labels = c(

'Male'='Male,Global',

'Female'='Female,Global'

)) +

scale_x_continuous(breaks = 1:20,

labels=c('<5 years','5-9 years','10-14 years','15-19 years','20-24 years',

'25-29 years','30-34 years','35-39 years','40-44 years','45-49 years',

'50-54 years','55-59 years','60-64 years','65-69 years','70-74 years',

'75-79 years','80-84 years','85-89 years','90-94 years','95+ years')) +

scale_y_continuous(sec.axis = sec_axis(~.*ad_factor, name = "Gender, Global Rate (per 100,000)",

labels = function(x) format(abs(x), scientific = FALSE)),

labels = function(x) format(abs(x), scientific = FALSE)) +

theme_bw() +

theme(legend.position = "right",

panel.grid.major = element_line(color = "gray90"),

panel.grid.minor = element_line(color = "gray95"),

axis.text.x = element_text(angle = 45, hjust = 1)) +

labs(title = "",

x = 'Age',

y = 'Number')

p

DALY_age <- IS %>%

filter(measure == "DALYs (Disability-Adjusted Life Years)" &

sex != "Both" &

age != "All ages" &

age != "Age-standardized" &

year == 2021) %>%

filter(location=="Global" |

location=="High SDI" |

location=="High-middle SDI" |

location=="Middle SDI" |

location=="Low-middle SDI" |

location=="Low SDI") %>%

mutate(val=ifelse(sex=="Male",val,-val),

lower=ifelse(sex=="Male",lower,-lower),

upper=ifelse(sex=="Male",upper,-upper)) %>%

mutate(group=ifelse(location=="High SDI"&sex=="Male","a",ifelse(

location=="High-middle SDI"&sex=="Male","b",ifelse(

location=="Middle SDI"&sex=="Male","c",ifelse(

location=="Low-middle SDI"&sex=="Male","d",ifelse(

location=="Low SDI"&sex=="Male","e",ifelse(

location=="High SDI"&sex=="Female","f",ifelse(

location=="High-middle SDI"&sex=="Female","g",ifelse(

location=="Middle SDI"&sex=="Female","h",ifelse(

location=="Low-middle SDI"&sex=="Female","i","j")))))))))) %>%

mutate(age_new=ifelse(age=="<5 years",1,ifelse(

age=="5-9 years",2,ifelse(

age=="10-14 years",3,ifelse(

age=="15-19 years",4,ifelse(

age=="20-24 years",5,ifelse(

age=="25-29 years",6,ifelse(

age=="30-34 years",7,ifelse(

age=="35-39 years",8,ifelse(

age=="40-44 years",9,ifelse(

age=="45-49 years",10,ifelse(

age=="50-54 years",11,ifelse(

age=="55-59 years",12,ifelse(

age=="60-64 years",13,ifelse(

age=="65-69 years",14,ifelse(

age=="70-74 years",15,ifelse(

age=="75-79 years",16,ifelse(

age=="80-84 years",17,ifelse(

age=="85-89 years",18,ifelse(

age=="90-94 years",19,20)))))))))))))))))))) %>%

dplyr::select(,c(2,3,4,5,7:11))

ad_factor <- max(Incidence_age$val[Incidence_age$metric=="Rate"&Incidence_age$location=="Global"])/

max(Incidence_age$val[Incidence_age$metric=="Number"&Incidence_age$location!="Global"])

p <- ggplot() +

geom_col(data = subset(Incidence_age,metric=='Number'&location!="Global"),

aes(x = age_new, y = val, fill = group)) +

geom_line(data = subset(Incidence_age,metric=='Rate'&location=="Global"),

aes(x = age_new, y = val/ad_factor, color = sex, group = sex), size = 1) +

geom_ribbon(data = subset(Incidence_age,metric=='Rate'&location=="Global"),

aes(x = age_new, ymin = lower/ad_factor, ymax = upper/ad_factor, fill = sex), alpha = 0.2) +

scale_fill_manual(name="Gender, SDI region (Number)",

values = c("a" = "#338a57",

"b" = "#5bb577",

"c" = "#90d191",

"d" = "#c9e9c2",

"e" = "#f1f9ec",

"f" = "#ca3352",

"g" = "#f3624d",

"h" = "#fda364",

"i" = "#fed67e",

"j" = "#ffffc1"),

labels=c(

"a"="Male,High SDI",

'b'='Male,High-middle SDI',

'c'='Male,Middle SDI',

'd'='Male,Low-middle SDI',

'e'='Male,Low SDI',

'f'='Female,High SDI',

'g'='Female,High-middle SDI',

'h'='Female,Middle SDI',

'i'='Female,Low-middle SDI',

'j'='Female,Low SDI'

)) +

scale_color_manual(name="Gender, Global Rate (per 100,000)",

values = c("Male" = "#006d2b", "Female" = "#31a255"),

labels = c(

'Male'='Male,Global',

'Female'='Female,Global'

)) +

scale_x_continuous(breaks = 1:20,

labels=c('<5 years','5-9 years','10-14 years','15-19 years','20-24 years',

'25-29 years','30-34 years','35-39 years','40-44 years','45-49 years',

'50-54 years','55-59 years','60-64 years','65-69 years','70-74 years',

'75-79 years','80-84 years','85-89 years','90-94 years','95+ years')) +

scale_y_continuous(sec.axis = sec_axis(~.*ad_factor, name = "Gender, Global Rate (per 100,000)",

labels = function(x) format(abs(x), scientific = FALSE)),

labels = function(x) format(abs(x), scientific = FALSE)) +

theme_bw() +

theme(legend.position = "right",

panel.grid.major = element_line(color = "gray90"),

panel.grid.minor = element_line(color = "gray95"),

axis.text.x = element_text(angle = 45, hjust = 1)) +

labs(title = "",

x = 'Age',

y = 'Number')

p

IS <- AML_region %>%

dplyr::select(measure_name,location_name,metric_name,

sex_name,age_name,year,val,lower,upper) %>%

rename(measure=measure_name,

location=location_name,

metric=metric_name,

sex=sex_name,

age=age_name)

Incidence_age <- IS %>%

filter(measure == "Incidence" &

sex != "Both" &

age != "All ages" &

age != "Age-standardized" &

year == 2021) %>%

filter(location=="Global" |

location=="High SDI" |

location=="High-middle SDI" |

location=="Middle SDI" |

location=="Low-middle SDI" |

location=="Low SDI") %>%

mutate(val=ifelse(sex=="Male",val,-val),

lower=ifelse(sex=="Male",lower,-lower),

upper=ifelse(sex=="Male",upper,-upper)) %>%

mutate(group=ifelse(location=="High SDI"&sex=="Male","a",ifelse(

location=="High-middle SDI"&sex=="Male","b",ifelse(

location=="Middle SDI"&sex=="Male","c",ifelse(

location=="Low-middle SDI"&sex=="Male","d",ifelse(

location=="Low SDI"&sex=="Male","e",ifelse(

location=="High SDI"&sex=="Female","f",ifelse(

location=="High-middle SDI"&sex=="Female","g",ifelse(

location=="Middle SDI"&sex=="Female","h",ifelse(

location=="Low-middle SDI"&sex=="Female","i","j")))))))))) %>%

mutate(age_new=ifelse(age=="<5 years",1,ifelse(

age=="5-9 years",2,ifelse(

age=="10-14 years",3,ifelse(

age=="15-19 years",4,ifelse(

age=="20-24 years",5,ifelse(

age=="25-29 years",6,ifelse(

age=="30-34 years",7,ifelse(

age=="35-39 years",8,ifelse(

age=="40-44 years",9,ifelse(

age=="45-49 years",10,ifelse(

age=="50-54 years",11,ifelse(

age=="55-59 years",12,ifelse(

age=="60-64 years",13,ifelse(

age=="65-69 years",14,ifelse(

age=="70-74 years",15,ifelse(

age=="75-79 years",16,ifelse(

age=="80-84 years",17,ifelse(

age=="85-89 years",18,ifelse(

age=="90-94 years",19,20)))))))))))))))))))) %>%

dplyr::select(,c(2,3,4,5,7:11))

ad_factor <- max(Incidence_age$val[Incidence_age$metric=="Rate"&Incidence_age$location=="Global"])/

max(Incidence_age$val[Incidence_age$metric=="Number"&Incidence_age$location!="Global"])

p <- ggplot() +

geom_col(data = subset(Incidence_age,metric=='Number'&location!="Global"),

aes(x = age_new, y = val, fill = group)) +

geom_line(data = subset(Incidence_age,metric=='Rate'&location=="Global"),

aes(x = age_new, y = val/ad_factor, color = sex, group = sex), size = 1) +

geom_ribbon(data = subset(Incidence_age,metric=='Rate'&location=="Global"),

aes(x = age_new, ymin = lower/ad_factor, ymax = upper/ad_factor, fill = sex), alpha = 0.2) +

scale_fill_manual(name="Gender, SDI region (Number)",

values = c("a" = "#338a57",

"b" = "#5bb577",

"c" = "#90d191",

"d" = "#c9e9c2",

"e" = "#f1f9ec",

"f" = "#ca3352",

"g" = "#f3624d",

"h" = "#fda364",

"i" = "#fed67e",

"j" = "#ffffc1"),

labels=c(

"a"="Male,High SDI",

'b'='Male,High-middle SDI',

'c'='Male,Middle SDI',

'd'='Male,Low-middle SDI',

'e'='Male,Low SDI',

'f'='Female,High SDI',

'g'='Female,High-middle SDI',

'h'='Female,Middle SDI',

'i'='Female,Low-middle SDI',

'j'='Female,Low SDI'

)) +

scale_color_manual(name="Gender, Global Rate (per 100,000)",

values = c("Male" = "#006d2b", "Female" = "#31a255"),

labels = c(

'Male'='Male,Global',

'Female'='Female,Global'

)) +

scale_x_continuous(breaks = 1:20,

labels=c('<5 years','5-9 years','10-14 years','15-19 years','20-24 years',

'25-29 years','30-34 years','35-39 years','40-44 years','45-49 years',

'50-54 years','55-59 years','60-64 years','65-69 years','70-74 years',

'75-79 years','80-84 years','85-89 years','90-94 years','95+ years')) +

scale_y_continuous(sec.axis = sec_axis(~.*ad_factor, name = "Gender, Global Rate (per 100,000)",

labels = function(x) format(abs(x), scientific = FALSE)),

labels = function(x) format(abs(x), scientific = FALSE)) +

theme_bw() +

theme(legend.position = "right",

panel.grid.major = element_line(color = "gray90"),

panel.grid.minor = element_line(color = "gray95"),

axis.text.x = element_text(angle = 45, hjust = 1)) +

labs(title = "",

x = 'Age',

y = 'Number')

p

Prevalence_age <- IS %>%

filter(measure == "Prevalence" &

sex != "Both" &

age != "All ages" &

age != "Age-standardized" &

year == 2021) %>%

filter(location=="Global" |

location=="High SDI" |

location=="High-middle SDI" |

location=="Middle SDI" |

location=="Low-middle SDI" |

location=="Low SDI") %>%

mutate(val=ifelse(sex=="Male",val,-val),

lower=ifelse(sex=="Male",lower,-lower),

upper=ifelse(sex=="Male",upper,-upper)) %>%

mutate(group=ifelse(location=="High SDI"&sex=="Male","a",ifelse(

location=="High-middle SDI"&sex=="Male","b",ifelse(

location=="Middle SDI"&sex=="Male","c",ifelse(

location=="Low-middle SDI"&sex=="Male","d",ifelse(

location=="Low SDI"&sex=="Male","e",ifelse(

location=="High SDI"&sex=="Female","f",ifelse(

location=="High-middle SDI"&sex=="Female","g",ifelse(

location=="Middle SDI"&sex=="Female","h",ifelse(

location=="Low-middle SDI"&sex=="Female","i","j")))))))))) %>%

mutate(age_new=ifelse(age=="<5 years",1,ifelse(

age=="5-9 years",2,ifelse(

age=="10-14 years",3,ifelse(

age=="15-19 years",4,ifelse(

age=="20-24 years",5,ifelse(

age=="25-29 years",6,ifelse(

age=="30-34 years",7,ifelse(

age=="35-39 years",8,ifelse(

age=="40-44 years",9,ifelse(

age=="45-49 years",10,ifelse(

age=="50-54 years",11,ifelse(

age=="55-59 years",12,ifelse(

age=="60-64 years",13,ifelse(

age=="65-69 years",14,ifelse(

age=="70-74 years",15,ifelse(

age=="75-79 years",16,ifelse(

age=="80-84 years",17,ifelse(

age=="85-89 years",18,ifelse(

age=="90-94 years",19,20)))))))))))))))))))) %>%

dplyr::select(,c(2,3,4,5,7:11))

ad_factor <- max(Incidence_age$val[Incidence_age$metric=="Rate"&Incidence_age$location=="Global"])/

max(Incidence_age$val[Incidence_age$metric=="Number"&Incidence_age$location!="Global"])

p <- ggplot() +

geom_col(data = subset(Incidence_age,metric=='Number'&location!="Global"),

aes(x = age_new, y = val, fill = group)) +

geom_line(data = subset(Incidence_age,metric=='Rate'&location=="Global"),

aes(x = age_new, y = val/ad_factor, color = sex, group = sex), size = 1) +

geom_ribbon(data = subset(Incidence_age,metric=='Rate'&location=="Global"),

aes(x = age_new, ymin = lower/ad_factor, ymax = upper/ad_factor, fill = sex), alpha = 0.2) +

scale_fill_manual(name="Gender, SDI region (Number)",

values = c("a" = "#338a57",

"b" = "#5bb577",

"c" = "#90d191",

"d" = "#c9e9c2",

"e" = "#f1f9ec",

"f" = "#ca3352",

"g" = "#f3624d",

"h" = "#fda364",

"i" = "#fed67e",

"j" = "#ffffc1"),

labels=c(

"a"="Male,High SDI",

'b'='Male,High-middle SDI',

'c'='Male,Middle SDI',

'd'='Male,Low-middle SDI',

'e'='Male,Low SDI',

'f'='Female,High SDI',

'g'='Female,High-middle SDI',

'h'='Female,Middle SDI',

'i'='Female,Low-middle SDI',

'j'='Female,Low SDI'

)) +

scale_color_manual(name="Gender, Global Rate (per 100,000)",

values = c("Male" = "#006d2b", "Female" = "#31a255"),

labels = c(

'Male'='Male,Global',

'Female'='Female,Global'

)) +

scale_x_continuous(breaks = 1:20,

labels=c('<5 years','5-9 years','10-14 years','15-19 years','20-24 years',

'25-29 years','30-34 years','35-39 years','40-44 years','45-49 years',

'50-54 years','55-59 years','60-64 years','65-69 years','70-74 years',

'75-79 years','80-84 years','85-89 years','90-94 years','95+ years')) +

scale_y_continuous(sec.axis = sec_axis(~.*ad_factor, name = "Gender, Global Rate (per 100,000)",

labels = function(x) format(abs(x), scientific = FALSE)),

labels = function(x) format(abs(x), scientific = FALSE)) +

theme_bw() +

theme(legend.position = "right",

panel.grid.major = element_line(color = "gray90"),

panel.grid.minor = element_line(color = "gray95"),

axis.text.x = element_text(angle = 45, hjust = 1)) +

labs(title = "",

x = 'Age',

y = 'Number')

p

Deaths_age <- IS %>%

filter(measure == "Deaths" &

sex != "Both" &

age != "All ages" &

age != "Age-standardized" &

year == 2021) %>%

filter(location=="Global" |

location=="High SDI" |

location=="High-middle SDI" |

location=="Middle SDI" |

location=="Low-middle SDI" |

location=="Low SDI") %>%

mutate(val=ifelse(sex=="Male",val,-val),

lower=ifelse(sex=="Male",lower,-lower),

upper=ifelse(sex=="Male",upper,-upper)) %>%

mutate(group=ifelse(location=="High SDI"&sex=="Male","a",ifelse(

location=="High-middle SDI"&sex=="Male","b",ifelse(

location=="Middle SDI"&sex=="Male","c",ifelse(

location=="Low-middle SDI"&sex=="Male","d",ifelse(

location=="Low SDI"&sex=="Male","e",ifelse(

location=="High SDI"&sex=="Female","f",ifelse(

location=="High-middle SDI"&sex=="Female","g",ifelse(

location=="Middle SDI"&sex=="Female","h",ifelse(

location=="Low-middle SDI"&sex=="Female","i","j")))))))))) %>%

mutate(age_new=ifelse(age=="<5 years",1,ifelse(

age=="5-9 years",2,ifelse(

age=="10-14 years",3,ifelse(

age=="15-19 years",4,ifelse(

age=="20-24 years",5,ifelse(

age=="25-29 years",6,ifelse(

age=="30-34 years",7,ifelse(

age=="35-39 years",8,ifelse(

age=="40-44 years",9,ifelse(

age=="45-49 years",10,ifelse(

age=="50-54 years",11,ifelse(

age=="55-59 years",12,ifelse(

age=="60-64 years",13,ifelse(

age=="65-69 years",14,ifelse(

age=="70-74 years",15,ifelse(

age=="75-79 years",16,ifelse(

age=="80-84 years",17,ifelse(

age=="85-89 years",18,ifelse(

age=="90-94 years",19,20)))))))))))))))))))) %>%

dplyr::select(,c(2,3,4,5,7:11))

ad_factor <- max(Incidence_age$val[Incidence_age$metric=="Rate"&Incidence_age$location=="Global"])/

max(Incidence_age$val[Incidence_age$metric=="Number"&Incidence_age$location!="Global"])

p <- ggplot() +

geom_col(data = subset(Incidence_age,metric=='Number'&location!="Global"),

aes(x = age_new, y = val, fill = group)) +

geom_line(data = subset(Incidence_age,metric=='Rate'&location=="Global"),

aes(x = age_new, y = val/ad_factor, color = sex, group = sex), size = 1) +

geom_ribbon(data = subset(Incidence_age,metric=='Rate'&location=="Global"),

aes(x = age_new, ymin = lower/ad_factor, ymax = upper/ad_factor, fill = sex), alpha = 0.2) +

scale_fill_manual(name="Gender, SDI region (Number)",

values = c("a" = "#338a57",

"b" = "#5bb577",

"c" = "#90d191",

"d" = "#c9e9c2",

"e" = "#f1f9ec",

"f" = "#ca3352",

"g" = "#f3624d",

"h" = "#fda364",

"i" = "#fed67e",

"j" = "#ffffc1"),

labels=c(

"a"="Male,High SDI",

'b'='Male,High-middle SDI',

'c'='Male,Middle SDI',

'd'='Male,Low-middle SDI',

'e'='Male,Low SDI',

'f'='Female,High SDI',

'g'='Female,High-middle SDI',

'h'='Female,Middle SDI',

'i'='Female,Low-middle SDI',

'j'='Female,Low SDI'

)) +

scale_color_manual(name="Gender, Global Rate (per 100,000)",

values = c("Male" = "#006d2b", "Female" = "#31a255"),

labels = c(

'Male'='Male,Global',

'Female'='Female,Global'

)) +

scale_x_continuous(breaks = 1:20,

labels=c('<5 years','5-9 years','10-14 years','15-19 years','20-24 years',

'25-29 years','30-34 years','35-39 years','40-44 years','45-49 years',

'50-54 years','55-59 years','60-64 years','65-69 years','70-74 years',

'75-79 years','80-84 years','85-89 years','90-94 years','95+ years')) +

scale_y_continuous(sec.axis = sec_axis(~.*ad_factor, name = "Gender, Global Rate (per 100,000)",

labels = function(x) format(abs(x), scientific = FALSE)),

labels = function(x) format(abs(x), scientific = FALSE)) +

theme_bw() +

theme(legend.position = "right",

panel.grid.major = element_line(color = "gray90"),

panel.grid.minor = element_line(color = "gray95"),

axis.text.x = element_text(angle = 45, hjust = 1)) +

labs(title = "",

x = 'Age',

y = 'Number')

p

DALY_age <- IS %>%

filter(measure == "DALYs (Disability-Adjusted Life Years)" &

sex != "Both" &

age != "All ages" &

age != "Age-standardized" &

year == 2021) %>%

filter(location=="Global" |

location=="High SDI" |

location=="High-middle SDI" |

location=="Middle SDI" |

location=="Low-middle SDI" |

location=="Low SDI") %>%

mutate(val=ifelse(sex=="Male",val,-val),

lower=ifelse(sex=="Male",lower,-lower),

upper=ifelse(sex=="Male",upper,-upper)) %>%

mutate(group=ifelse(location=="High SDI"&sex=="Male","a",ifelse(

location=="High-middle SDI"&sex=="Male","b",ifelse(

location=="Middle SDI"&sex=="Male","c",ifelse(

location=="Low-middle SDI"&sex=="Male","d",ifelse(

location=="Low SDI"&sex=="Male","e",ifelse(

location=="High SDI"&sex=="Female","f",ifelse(

location=="High-middle SDI"&sex=="Female","g",ifelse(

location=="Middle SDI"&sex=="Female","h",ifelse(

location=="Low-middle SDI"&sex=="Female","i","j")))))))))) %>%

mutate(age_new=ifelse(age=="<5 years",1,ifelse(

age=="5-9 years",2,ifelse(

age=="10-14 years",3,ifelse(

age=="15-19 years",4,ifelse(

age=="20-24 years",5,ifelse(

age=="25-29 years",6,ifelse(

age=="30-34 years",7,ifelse(

age=="35-39 years",8,ifelse(

age=="40-44 years",9,ifelse(

age=="45-49 years",10,ifelse(

age=="50-54 years",11,ifelse(

age=="55-59 years",12,ifelse(

age=="60-64 years",13,ifelse(

age=="65-69 years",14,ifelse(

age=="70-74 years",15,ifelse(

age=="75-79 years",16,ifelse(

age=="80-84 years",17,ifelse(

age=="85-89 years",18,ifelse(

age=="90-94 years",19,20)))))))))))))))))))) %>%

dplyr::select(,c(2,3,4,5,7:11))

ad_factor <- max(Incidence_age$val[Incidence_age$metric=="Rate"&Incidence_age$location=="Global"])/

max(Incidence_age$val[Incidence_age$metric=="Number"&Incidence_age$location!="Global"])

p <- ggplot() +

geom_col(data = subset(Incidence_age,metric=='Number'&location!="Global"),

aes(x = age_new, y = val, fill = group)) +

geom_line(data = subset(Incidence_age,metric=='Rate'&location=="Global"),

aes(x = age_new, y = val/ad_factor, color = sex, group = sex), size = 1) +

geom_ribbon(data = subset(Incidence_age,metric=='Rate'&location=="Global"),

aes(x = age_new, ymin = lower/ad_factor, ymax = upper/ad_factor, fill = sex), alpha = 0.2) +

scale_fill_manual(name="Gender, SDI region (Number)",

values = c("a" = "#338a57",

"b" = "#5bb577",

"c" = "#90d191",

"d" = "#c9e9c2",

"e" = "#f1f9ec",

"f" = "#ca3352",

"g" = "#f3624d",

"h" = "#fda364",

"i" = "#fed67e",

"j" = "#ffffc1"),

labels=c(

"a"="Male,High SDI",

'b'='Male,High-middle SDI',

'c'='Male,Middle SDI',

'd'='Male,Low-middle SDI',

'e'='Male,Low SDI',

'f'='Female,High SDI',

'g'='Female,High-middle SDI',

'h'='Female,Middle SDI',

'i'='Female,Low-middle SDI',

'j'='Female,Low SDI'

)) +

scale_color_manual(name="Gender, Global Rate (per 100,000)",

values = c("Male" = "#006d2b", "Female" = "#31a255"),

labels = c(

'Male'='Male,Global',

'Female'='Female,Global'

)) +

scale_x_continuous(breaks = 1:20,

labels=c('<5 years','5-9 years','10-14 years','15-19 years','20-24 years',

'25-29 years','30-34 years','35-39 years','40-44 years','45-49 years',

'50-54 years','55-59 years','60-64 years','65-69 years','70-74 years',

'75-79 years','80-84 years','85-89 years','90-94 years','95+ years')) +

scale_y_continuous(sec.axis = sec_axis(~.*ad_factor, name = "Gender, Global Rate (per 100,000)",

labels = function(x) format(abs(x), scientific = FALSE)),

labels = function(x) format(abs(x), scientific = FALSE)) +

theme_bw() +

theme(legend.position = "right",

panel.grid.major = element_line(color = "gray90"),

panel.grid.minor = element_line(color = "gray95"),

axis.text.x = element_text(angle = 45, hjust = 1)) +

labs(title = "",

x = 'Age',

y = 'Number')

p

IS <- CML_region %>%

dplyr::select(measure_name,location_name,metric_name,

sex_name,age_name,year,val,lower,upper) %>%

rename(measure=measure_name,

location=location_name,

metric=metric_name,

sex=sex_name,

age=age_name)

Incidence_age <- IS %>%

filter(measure == "Incidence" &

sex != "Both" &

age != "All ages" &

age != "Age-standardized" &

year == 2021) %>%

filter(location=="Global" |

location=="High SDI" |

location=="High-middle SDI" |

location=="Middle SDI" |

location=="Low-middle SDI" |

location=="Low SDI") %>%

mutate(val=ifelse(sex=="Male",val,-val),

lower=ifelse(sex=="Male",lower,-lower),

upper=ifelse(sex=="Male",upper,-upper)) %>%

mutate(group=ifelse(location=="High SDI"&sex=="Male","a",ifelse(

location=="High-middle SDI"&sex=="Male","b",ifelse(

location=="Middle SDI"&sex=="Male","c",ifelse(

location=="Low-middle SDI"&sex=="Male","d",ifelse(

location=="Low SDI"&sex=="Male","e",ifelse(

location=="High SDI"&sex=="Female","f",ifelse(

location=="High-middle SDI"&sex=="Female","g",ifelse(

location=="Middle SDI"&sex=="Female","h",ifelse(

location=="Low-middle SDI"&sex=="Female","i","j")))))))))) %>%

mutate(age_new=ifelse(age=="<5 years",1,ifelse(

age=="5-9 years",2,ifelse(

age=="10-14 years",3,ifelse(

age=="15-19 years",4,ifelse(

age=="20-24 years",5,ifelse(

age=="25-29 years",6,ifelse(

age=="30-34 years",7,ifelse(

age=="35-39 years",8,ifelse(

age=="40-44 years",9,ifelse(

age=="45-49 years",10,ifelse(

age=="50-54 years",11,ifelse(

age=="55-59 years",12,ifelse(

age=="60-64 years",13,ifelse(

age=="65-69 years",14,ifelse(

age=="70-74 years",15,ifelse(

age=="75-79 years",16,ifelse(

age=="80-84 years",17,ifelse(

age=="85-89 years",18,ifelse(

age=="90-94 years",19,20)))))))))))))))))))) %>%

dplyr::select(,c(2,3,4,5,7:11))

ad_factor <- max(Incidence_age$val[Incidence_age$metric=="Rate"&Incidence_age$location=="Global"])/

max(Incidence_age$val[Incidence_age$metric=="Number"&Incidence_age$location!="Global"])

p <- ggplot() +

geom_col(data = subset(Incidence_age,metric=='Number'&location!="Global"),

aes(x = age_new, y = val, fill = group)) +

geom_line(data = subset(Incidence_age,metric=='Rate'&location=="Global"),

aes(x = age_new, y = val/ad_factor, color = sex, group = sex), size = 1) +

geom_ribbon(data = subset(Incidence_age,metric=='Rate'&location=="Global"),

aes(x = age_new, ymin = lower/ad_factor, ymax = upper/ad_factor, fill = sex), alpha = 0.2) +

scale_fill_manual(name="Gender, SDI region (Number)",

values = c("a" = "#338a57",

"b" = "#5bb577",

"c" = "#90d191",

"d" = "#c9e9c2",

"e" = "#f1f9ec",

"f" = "#ca3352",

"g" = "#f3624d",

"h" = "#fda364",

"i" = "#fed67e",

"j" = "#ffffc1"),

labels=c(

"a"="Male,High SDI",

'b'='Male,High-middle SDI',

'c'='Male,Middle SDI',

'd'='Male,Low-middle SDI',

'e'='Male,Low SDI',

'f'='Female,High SDI',

'g'='Female,High-middle SDI',

'h'='Female,Middle SDI',

'i'='Female,Low-middle SDI',

'j'='Female,Low SDI'

)) +

scale_color_manual(name="Gender, Global Rate (per 100,000)",

values = c("Male" = "#006d2b", "Female" = "#31a255"),

labels = c(

'Male'='Male,Global',

'Female'='Female,Global'

)) +

scale_x_continuous(breaks = 1:20,

labels=c('<5 years','5-9 years','10-14 years','15-19 years','20-24 years',

'25-29 years','30-34 years','35-39 years','40-44 years','45-49 years',

'50-54 years','55-59 years','60-64 years','65-69 years','70-74 years',

'75-79 years','80-84 years','85-89 years','90-94 years','95+ years')) +

scale_y_continuous(sec.axis = sec_axis(~.*ad_factor, name = "Gender, Global Rate (per 100,000)",

labels = function(x) format(abs(x), scientific = FALSE)),

labels = function(x) format(abs(x), scientific = FALSE)) +

theme_bw() +

theme(legend.position = "right",

panel.grid.major = element_line(color = "gray90"),

panel.grid.minor = element_line(color = "gray95"),

axis.text.x = element_text(angle = 45, hjust = 1)) +

labs(title = "",

x = 'Age',

y = 'Number')

p

Prevalence_age <- IS %>%

filter(measure == "Prevalence" &

sex != "Both" &

age != "All ages" &

age != "Age-standardized" &

year == 2021) %>%

filter(location=="Global" |

location=="High SDI" |

location=="High-middle SDI" |

location=="Middle SDI" |

location=="Low-middle SDI" |

location=="Low SDI") %>%

mutate(val=ifelse(sex=="Male",val,-val),

lower=ifelse(sex=="Male",lower,-lower),

upper=ifelse(sex=="Male",upper,-upper)) %>%

mutate(group=ifelse(location=="High SDI"&sex=="Male","a",ifelse(

location=="High-middle SDI"&sex=="Male","b",ifelse(

location=="Middle SDI"&sex=="Male","c",ifelse(

location=="Low-middle SDI"&sex=="Male","d",ifelse(

location=="Low SDI"&sex=="Male","e",ifelse(

location=="High SDI"&sex=="Female","f",ifelse(

location=="High-middle SDI"&sex=="Female","g",ifelse(

location=="Middle SDI"&sex=="Female","h",ifelse(

location=="Low-middle SDI"&sex=="Female","i","j")))))))))) %>%

mutate(age_new=ifelse(age=="<5 years",1,ifelse(

age=="5-9 years",2,ifelse(

age=="10-14 years",3,ifelse(

age=="15-19 years",4,ifelse(

age=="20-24 years",5,ifelse(

age=="25-29 years",6,ifelse(

age=="30-34 years",7,ifelse(

age=="35-39 years",8,ifelse(

age=="40-44 years",9,ifelse(

age=="45-49 years",10,ifelse(

age=="50-54 years",11,ifelse(

age=="55-59 years",12,ifelse(

age=="60-64 years",13,ifelse(

age=="65-69 years",14,ifelse(

age=="70-74 years",15,ifelse(

age=="75-79 years",16,ifelse(

age=="80-84 years",17,ifelse(

age=="85-89 years",18,ifelse(

age=="90-94 years",19,20)))))))))))))))))))) %>%

dplyr::select(,c(2,3,4,5,7:11))

ad_factor <- max(Incidence_age$val[Incidence_age$metric=="Rate"&Incidence_age$location=="Global"])/

max(Incidence_age$val[Incidence_age$metric=="Number"&Incidence_age$location!="Global"])

p <- ggplot() +

geom_col(data = subset(Incidence_age,metric=='Number'&location!="Global"),

aes(x = age_new, y = val, fill = group)) +

geom_line(data = subset(Incidence_age,metric=='Rate'&location=="Global"),

aes(x = age_new, y = val/ad_factor, color = sex, group = sex), size = 1) +

geom_ribbon(data = subset(Incidence_age,metric=='Rate'&location=="Global"),

aes(x = age_new, ymin = lower/ad_factor, ymax = upper/ad_factor, fill = sex), alpha = 0.2) +

scale_fill_manual(name="Gender, SDI region (Number)",

values = c("a" = "#338a57",

"b" = "#5bb577",

"c" = "#90d191",

"d" = "#c9e9c2",

"e" = "#f1f9ec",

"f" = "#ca3352",

"g" = "#f3624d",

"h" = "#fda364",

"i" = "#fed67e",

"j" = "#ffffc1"),

labels=c(

"a"="Male,High SDI",

'b'='Male,High-middle SDI',

'c'='Male,Middle SDI',

'd'='Male,Low-middle SDI',

'e'='Male,Low SDI',

'f'='Female,High SDI',

'g'='Female,High-middle SDI',

'h'='Female,Middle SDI',

'i'='Female,Low-middle SDI',

'j'='Female,Low SDI'

)) +

scale_color_manual(name="Gender, Global Rate (per 100,000)",

values = c("Male" = "#006d2b", "Female" = "#31a255"),

labels = c(

'Male'='Male,Global',

'Female'='Female,Global'

)) +

scale_x_continuous(breaks = 1:20,

labels=c('<5 years','5-9 years','10-14 years','15-19 years','20-24 years',

'25-29 years','30-34 years','35-39 years','40-44 years','45-49 years',

'50-54 years','55-59 years','60-64 years','65-69 years','70-74 years',

'75-79 years','80-84 years','85-89 years','90-94 years','95+ years')) +

scale_y_continuous(sec.axis = sec_axis(~.*ad_factor, name = "Gender, Global Rate (per 100,000)",

labels = function(x) format(abs(x), scientific = FALSE)),

labels = function(x) format(abs(x), scientific = FALSE)) +

theme_bw() +

theme(legend.position = "right",

panel.grid.major = element_line(color = "gray90"),

panel.grid.minor = element_line(color = "gray95"),

axis.text.x = element_text(angle = 45, hjust = 1)) +

labs(title = "",

x = 'Age',

y = 'Number')

p

Deaths_age <- IS %>%

filter(measure == "Deaths" &

sex != "Both" &

age != "All ages" &

age != "Age-standardized" &

year == 2021) %>%

filter(location=="Global" |

location=="High SDI" |

location=="High-middle SDI" |

location=="Middle SDI" |

location=="Low-middle SDI" |

location=="Low SDI") %>%

mutate(val=ifelse(sex=="Male",val,-val),

lower=ifelse(sex=="Male",lower,-lower),

upper=ifelse(sex=="Male",upper,-upper)) %>%

mutate(group=ifelse(location=="High SDI"&sex=="Male","a",ifelse(

location=="High-middle SDI"&sex=="Male","b",ifelse(

location=="Middle SDI"&sex=="Male","c",ifelse(

location=="Low-middle SDI"&sex=="Male","d",ifelse(

location=="Low SDI"&sex=="Male","e",ifelse(

location=="High SDI"&sex=="Female","f",ifelse(

location=="High-middle SDI"&sex=="Female","g",ifelse(

location=="Middle SDI"&sex=="Female","h",ifelse(

location=="Low-middle SDI"&sex=="Female","i","j")))))))))) %>%

mutate(age_new=ifelse(age=="<5 years",1,ifelse(

age=="5-9 years",2,ifelse(

age=="10-14 years",3,ifelse(

age=="15-19 years",4,ifelse(

age=="20-24 years",5,ifelse(

age=="25-29 years",6,ifelse(

age=="30-34 years",7,ifelse(

age=="35-39 years",8,ifelse(

age=="40-44 years",9,ifelse(

age=="45-49 years",10,ifelse(

age=="50-54 years",11,ifelse(

age=="55-59 years",12,ifelse(

age=="60-64 years",13,ifelse(

age=="65-69 years",14,ifelse(

age=="70-74 years",15,ifelse(

age=="75-79 years",16,ifelse(

age=="80-84 years",17,ifelse(

age=="85-89 years",18,ifelse(

age=="90-94 years",19,20)))))))))))))))))))) %>%

dplyr::select(,c(2,3,4,5,7:11))

ad_factor <- max(Incidence_age$val[Incidence_age$metric=="Rate"&Incidence_age$location=="Global"])/

max(Incidence_age$val[Incidence_age$metric=="Number"&Incidence_age$location!="Global"])

p <- ggplot() +

geom_col(data = subset(Incidence_age,metric=='Number'&location!="Global"),

aes(x = age_new, y = val, fill = group)) +

geom_line(data = subset(Incidence_age,metric=='Rate'&location=="Global"),

aes(x = age_new, y = val/ad_factor, color = sex, group = sex), size = 1) +

geom_ribbon(data = subset(Incidence_age,metric=='Rate'&location=="Global"),

aes(x = age_new, ymin = lower/ad_factor, ymax = upper/ad_factor, fill = sex), alpha = 0.2) +

scale_fill_manual(name="Gender, SDI region (Number)",

values = c("a" = "#338a57",

"b" = "#5bb577",

"c" = "#90d191",

"d" = "#c9e9c2",

"e" = "#f1f9ec",

"f" = "#ca3352",

"g" = "#f3624d",

"h" = "#fda364",

"i" = "#fed67e",

"j" = "#ffffc1"),

labels=c(

"a"="Male,High SDI",

'b'='Male,High-middle SDI',

'c'='Male,Middle SDI',

'd'='Male,Low-middle SDI',

'e'='Male,Low SDI',

'f'='Female,High SDI',

'g'='Female,High-middle SDI',

'h'='Female,Middle SDI',

'i'='Female,Low-middle SDI',

'j'='Female,Low SDI'

)) +

scale_color_manual(name="Gender, Global Rate (per 100,000)",

values = c("Male" = "#006d2b", "Female" = "#31a255"),

labels = c(

'Male'='Male,Global',

'Female'='Female,Global'

)) +

scale_x_continuous(breaks = 1:20,

labels=c('<5 years','5-9 years','10-14 years','15-19 years','20-24 years',

'25-29 years','30-34 years','35-39 years','40-44 years','45-49 years',

'50-54 years','55-59 years','60-64 years','65-69 years','70-74 years',

'75-79 years','80-84 years','85-89 years','90-94 years','95+ years')) +

scale_y_continuous(sec.axis = sec_axis(~.*ad_factor, name = "Gender, Global Rate (per 100,000)",

labels = function(x) format(abs(x), scientific = FALSE)),

labels = function(x) format(abs(x), scientific = FALSE)) +

theme_bw() +

theme(legend.position = "right",

panel.grid.major = element_line(color = "gray90"),

panel.grid.minor = element_line(color = "gray95"),

axis.text.x = element_text(angle = 45, hjust = 1)) +

labs(title = "",

x = 'Age',

y = 'Number')

p

DALY_age <- IS %>%

filter(measure == "DALYs (Disability-Adjusted Life Years)" &

sex != "Both" &

age != "All ages" &

age != "Age-standardized" &

year == 2021) %>%

filter(location=="Global" |

location=="High SDI" |

location=="High-middle SDI" |

location=="Middle SDI" |

location=="Low-middle SDI" |

location=="Low SDI") %>%

mutate(val=ifelse(sex=="Male",val,-val),

lower=ifelse(sex=="Male",lower,-lower),

upper=ifelse(sex=="Male",upper,-upper)) %>%

mutate(group=ifelse(location=="High SDI"&sex=="Male","a",ifelse(

location=="High-middle SDI"&sex=="Male","b",ifelse(

location=="Middle SDI"&sex=="Male","c",ifelse(

location=="Low-middle SDI"&sex=="Male","d",ifelse(

location=="Low SDI"&sex=="Male","e",ifelse(

location=="High SDI"&sex=="Female","f",ifelse(

location=="High-middle SDI"&sex=="Female","g",ifelse(

location=="Middle SDI"&sex=="Female","h",ifelse(

location=="Low-middle SDI"&sex=="Female","i","j")))))))))) %>%

mutate(age_new=ifelse(age=="<5 years",1,ifelse(

age=="5-9 years",2,ifelse(

age=="10-14 years",3,ifelse(

age=="15-19 years",4,ifelse(

age=="20-24 years",5,ifelse(

age=="25-29 years",6,ifelse(

age=="30-34 years",7,ifelse(

age=="35-39 years",8,ifelse(

age=="40-44 years",9,ifelse(

age=="45-49 years",10,ifelse(

age=="50-54 years",11,ifelse(

age=="55-59 years",12,ifelse(

age=="60-64 years",13,ifelse(

age=="65-69 years",14,ifelse(

age=="70-74 years",15,ifelse(

age=="75-79 years",16,ifelse(

age=="80-84 years",17,ifelse(

age=="85-89 years",18,ifelse(

age=="90-94 years",19,20)))))))))))))))))))) %>%

dplyr::select(,c(2,3,4,5,7:11))

ad_factor <- max(Incidence_age$val[Incidence_age$metric=="Rate"&Incidence_age$location=="Global"])/

max(Incidence_age$val[Incidence_age$metric=="Number"&Incidence_age$location!="Global"])

p <- ggplot() +

geom_col(data = subset(Incidence_age,metric=='Number'&location!="Global"),

aes(x = age_new, y = val, fill = group)) +

geom_line(data = subset(Incidence_age,metric=='Rate'&location=="Global"),

aes(x = age_new, y = val/ad_factor, color = sex, group = sex), size = 1) +

geom_ribbon(data = subset(Incidence_age,metric=='Rate'&location=="Global"),

aes(x = age_new, ymin = lower/ad_factor, ymax = upper/ad_factor, fill = sex), alpha = 0.2) +

scale_fill_manual(name="Gender, SDI region (Number)",

values = c("a" = "#338a57",

"b" = "#5bb577",

"c" = "#90d191",

"d" = "#c9e9c2",

"e" = "#f1f9ec",

"f" = "#ca3352",

"g" = "#f3624d",

"h" = "#fda364",

"i" = "#fed67e",

"j" = "#ffffc1"),

labels=c(

"a"="Male,High SDI",

'b'='Male,High-middle SDI',

'c'='Male,Middle SDI',

'd'='Male,Low-middle SDI',

'e'='Male,Low SDI',

'f'='Female,High SDI',

'g'='Female,High-middle SDI',

'h'='Female,Middle SDI',

'i'='Female,Low-middle SDI',

'j'='Female,Low SDI'

)) +

scale_color_manual(name="Gender, Global Rate (per 100,000)",

values = c("Male" = "#006d2b", "Female" = "#31a255"),

labels = c(

'Male'='Male,Global',

'Female'='Female,Global'

)) +

scale_x_continuous(breaks = 1:20,

labels=c('<5 years','5-9 years','10-14 years','15-19 years','20-24 years',

'25-29 years','30-34 years','35-39 years','40-44 years','45-49 years',

'50-54 years','55-59 years','60-64 years','65-69 years','70-74 years',

'75-79 years','80-84 years','85-89 years','90-94 years','95+ years')) +

scale_y_continuous(sec.axis = sec_axis(~.*ad_factor, name = "Gender, Global Rate (per 100,000)",

labels = function(x) format(abs(x), scientific = FALSE)),

labels = function(x) format(abs(x), scientific = FALSE)) +

theme_bw() +

theme(legend.position = "right",

panel.grid.major = element_line(color = "gray90"),

panel.grid.minor = element_line(color = "gray95"),

axis.text.x = element_text(angle = 45, hjust = 1)) +

labs(title = "",

x = 'Age',

y = 'Number')

p

IS <- ALL_region %>%

dplyr::select(measure_name,location_name,metric_name,

sex_name,age_name,year,val,lower,upper) %>%

rename(measure=measure_name,

location=location_name,

metric=metric_name,

sex=sex_name,

age=age_name)

Incidence_age <- IS %>%

filter(measure == "Incidence" &

sex != "Both" &

age != "All ages" &

age != "Age-standardized" &

year == 2021) %>%

filter(location=="Global" |

location=="High SDI" |

location=="High-middle SDI" |

location=="Middle SDI" |

location=="Low-middle SDI" |

location=="Low SDI") %>%

mutate(val=ifelse(sex=="Male",val,-val),

lower=ifelse(sex=="Male",lower,-lower),

upper=ifelse(sex=="Male",upper,-upper)) %>%

mutate(group=ifelse(location=="High SDI"&sex=="Male","a",ifelse(

location=="High-middle SDI"&sex=="Male","b",ifelse(

location=="Middle SDI"&sex=="Male","c",ifelse(

location=="Low-middle SDI"&sex=="Male","d",ifelse(

location=="Low SDI"&sex=="Male","e",ifelse(

location=="High SDI"&sex=="Female","f",ifelse(

location=="High-middle SDI"&sex=="Female","g",ifelse(

location=="Middle SDI"&sex=="Female","h",ifelse(

location=="Low-middle SDI"&sex=="Female","i","j")))))))))) %>%

mutate(age_new=ifelse(age=="<5 years",1,ifelse(

age=="5-9 years",2,ifelse(

age=="10-14 years",3,ifelse(

age=="15-19 years",4,ifelse(

age=="20-24 years",5,ifelse(

age=="25-29 years",6,ifelse(

age=="30-34 years",7,ifelse(

age=="35-39 years",8,ifelse(

age=="40-44 years",9,ifelse(

age=="45-49 years",10,ifelse(

age=="50-54 years",11,ifelse(

age=="55-59 years",12,ifelse(

age=="60-64 years",13,ifelse(

age=="65-69 years",14,ifelse(

age=="70-74 years",15,ifelse(

age=="75-79 years",16,ifelse(

age=="80-84 years",17,ifelse(

age=="85-89 years",18,ifelse(

age=="90-94 years",19,20)))))))))))))))))))) %>%

dplyr::select(,c(2,3,4,5,7:11))

ad_factor <- max(Incidence_age$val[Incidence_age$metric=="Rate"&Incidence_age$location=="Global"])/

max(Incidence_age$val[Incidence_age$metric=="Number"&Incidence_age$location!="Global"])

p <- ggplot() +

geom_col(data = subset(Incidence_age,metric=='Number'&location!="Global"),

aes(x = age_new, y = val, fill = group)) +

geom_line(data = subset(Incidence_age,metric=='Rate'&location=="Global"),

aes(x = age_new, y = val/ad_factor, color = sex, group = sex), size = 1) +

geom_ribbon(data = subset(Incidence_age,metric=='Rate'&location=="Global"),

aes(x = age_new, ymin = lower/ad_factor, ymax = upper/ad_factor, fill = sex), alpha = 0.2) +

scale_fill_manual(name="Gender, SDI region (Number)",

values = c("a" = "#338a57",

"b" = "#5bb577",

"c" = "#90d191",

"d" = "#c9e9c2",

"e" = "#f1f9ec",

"f" = "#ca3352",

"g" = "#f3624d",

"h" = "#fda364",

"i" = "#fed67e",

"j" = "#ffffc1"),

labels=c(

"a"="Male,High SDI",

'b'='Male,High-middle SDI',

'c'='Male,Middle SDI',

'd'='Male,Low-middle SDI',

'e'='Male,Low SDI',

'f'='Female,High SDI',

'g'='Female,High-middle SDI',

'h'='Female,Middle SDI',

'i'='Female,Low-middle SDI',

'j'='Female,Low SDI'

)) +

scale_color_manual(name="Gender, Global Rate (per 100,000)",

values = c("Male" = "#006d2b", "Female" = "#31a255"),

labels = c(

'Male'='Male,Global',

'Female'='Female,Global'

)) +

scale_x_continuous(breaks = 1:20,

labels=c('<5 years','5-9 years','10-14 years','15-19 years','20-24 years',

'25-29 years','30-34 years','35-39 years','40-44 years','45-49 years',

'50-54 years','55-59 years','60-64 years','65-69 years','70-74 years',

'75-79 years','80-84 years','85-89 years','90-94 years','95+ years')) +

scale_y_continuous(sec.axis = sec_axis(~.*ad_factor, name = "Gender, Global Rate (per 100,000)",

labels = function(x) format(abs(x), scientific = FALSE)),

labels = function(x) format(abs(x), scientific = FALSE)) +

theme_bw() +

theme(legend.position = "right",

panel.grid.major = element_line(color = "gray90"),

panel.grid.minor = element_line(color = "gray95"),

axis.text.x = element_text(angle = 45, hjust = 1)) +

labs(title = "",

x = 'Age',

y = 'Number')

p

Prevalence_age <- IS %>%

filter(measure == "Prevalence" &

sex != "Both" &

age != "All ages" &

age != "Age-standardized" &

year == 2021) %>%

filter(location=="Global" |

location=="High SDI" |

location=="High-middle SDI" |

location=="Middle SDI" |

location=="Low-middle SDI" |

location=="Low SDI") %>%

mutate(val=ifelse(sex=="Male",val,-val),

lower=ifelse(sex=="Male",lower,-lower),

upper=ifelse(sex=="Male",upper,-upper)) %>%

mutate(group=ifelse(location=="High SDI"&sex=="Male","a",ifelse(

location=="High-middle SDI"&sex=="Male","b",ifelse(

location=="Middle SDI"&sex=="Male","c",ifelse(

location=="Low-middle SDI"&sex=="Male","d",ifelse(

location=="Low SDI"&sex=="Male","e",ifelse(

location=="High SDI"&sex=="Female","f",ifelse(

location=="High-middle SDI"&sex=="Female","g",ifelse(

location=="Middle SDI"&sex=="Female","h",ifelse(

location=="Low-middle SDI"&sex=="Female","i","j")))))))))) %>%

mutate(age_new=ifelse(age=="<5 years",1,ifelse(

age=="5-9 years",2,ifelse(

age=="10-14 years",3,ifelse(

age=="15-19 years",4,ifelse(

age=="20-24 years",5,ifelse(

age=="25-29 years",6,ifelse(

age=="30-34 years",7,ifelse(

age=="35-39 years",8,ifelse(

age=="40-44 years",9,ifelse(

age=="45-49 years",10,ifelse(

age=="50-54 years",11,ifelse(

age=="55-59 years",12,ifelse(

age=="60-64 years",13,ifelse(

age=="65-69 years",14,ifelse(

age=="70-74 years",15,ifelse(

age=="75-79 years",16,ifelse(

age=="80-84 years",17,ifelse(

age=="85-89 years",18,ifelse(

age=="90-94 years",19,20)))))))))))))))))))) %>%

dplyr::select(,c(2,3,4,5,7:11))

ad_factor <- max(Incidence_age$val[Incidence_age$metric=="Rate"&Incidence_age$location=="Global"])/

max(Incidence_age$val[Incidence_age$metric=="Number"&Incidence_age$location!="Global"])

p <- ggplot() +

geom_col(data = subset(Incidence_age,metric=='Number'&location!="Global"),

aes(x = age_new, y = val, fill = group)) +

geom_line(data = subset(Incidence_age,metric=='Rate'&location=="Global"),

aes(x = age_new, y = val/ad_factor, color = sex, group = sex), size = 1) +

geom_ribbon(data = subset(Incidence_age,metric=='Rate'&location=="Global"),

aes(x = age_new, ymin = lower/ad_factor, ymax = upper/ad_factor, fill = sex), alpha = 0.2) +

scale_fill_manual(name="Gender, SDI region (Number)",

values = c("a" = "#338a57",

"b" = "#5bb577",

"c" = "#90d191",

"d" = "#c9e9c2",

"e" = "#f1f9ec",

"f" = "#ca3352",

"g" = "#f3624d",

"h" = "#fda364",

"i" = "#fed67e",

"j" = "#ffffc1"),

labels=c(

"a"="Male,High SDI",

'b'='Male,High-middle SDI',

'c'='Male,Middle SDI',

'd'='Male,Low-middle SDI',

'e'='Male,Low SDI',

'f'='Female,High SDI',

'g'='Female,High-middle SDI',

'h'='Female,Middle SDI',

'i'='Female,Low-middle SDI',

'j'='Female,Low SDI'

)) +

scale_color_manual(name="Gender, Global Rate (per 100,000)",

values = c("Male" = "#006d2b", "Female" = "#31a255"),

labels = c(

'Male'='Male,Global',

'Female'='Female,Global'

)) +

scale_x_continuous(breaks = 1:20,

labels=c('<5 years','5-9 years','10-14 years','15-19 years','20-24 years',

'25-29 years','30-34 years','35-39 years','40-44 years','45-49 years',

'50-54 years','55-59 years','60-64 years','65-69 years','70-74 years',

'75-79 years','80-84 years','85-89 years','90-94 years','95+ years')) +

scale_y_continuous(sec.axis = sec_axis(~.*ad_factor, name = "Gender, Global Rate (per 100,000)",

labels = function(x) format(abs(x), scientific = FALSE)),

labels = function(x) format(abs(x), scientific = FALSE)) +

theme_bw() +

theme(legend.position = "right",

panel.grid.major = element_line(color = "gray90"),

panel.grid.minor = element_line(color = "gray95"),

axis.text.x = element_text(angle = 45, hjust = 1)) +

labs(title = "",

x = 'Age',

y = 'Number')

p

Deaths_age <- IS %>%

filter(measure == "Deaths" &

sex != "Both" &

age != "All ages" &

age != "Age-standardized" &

year == 2021) %>%

filter(location=="Global" |

location=="High SDI" |

location=="High-middle SDI" |

location=="Middle SDI" |

location=="Low-middle SDI" |

location=="Low SDI") %>%

mutate(val=ifelse(sex=="Male",val,-val),

lower=ifelse(sex=="Male",lower,-lower),

upper=ifelse(sex=="Male",upper,-upper)) %>%

mutate(group=ifelse(location=="High SDI"&sex=="Male","a",ifelse(

location=="High-middle SDI"&sex=="Male","b",ifelse(

location=="Middle SDI"&sex=="Male","c",ifelse(

location=="Low-middle SDI"&sex=="Male","d",ifelse(

location=="Low SDI"&sex=="Male","e",ifelse(

location=="High SDI"&sex=="Female","f",ifelse(

location=="High-middle SDI"&sex=="Female","g",ifelse(

location=="Middle SDI"&sex=="Female","h",ifelse(

location=="Low-middle SDI"&sex=="Female","i","j")))))))))) %>%

mutate(age_new=ifelse(age=="<5 years",1,ifelse(

age=="5-9 years",2,ifelse(

age=="10-14 years",3,ifelse(

age=="15-19 years",4,ifelse(

age=="20-24 years",5,ifelse(

age=="25-29 years",6,ifelse(

age=="30-34 years",7,ifelse(

age=="35-39 years",8,ifelse(

age=="40-44 years",9,ifelse(

age=="45-49 years",10,ifelse(

age=="50-54 years",11,ifelse(

age=="55-59 years",12,ifelse(

age=="60-64 years",13,ifelse(

age=="65-69 years",14,ifelse(

age=="70-74 years",15,ifelse(

age=="75-79 years",16,ifelse(

age=="80-84 years",17,ifelse(

age=="85-89 years",18,ifelse(

age=="90-94 years",19,20)))))))))))))))))))) %>%

dplyr::select(,c(2,3,4,5,7:11))

ad_factor <- max(Incidence_age$val[Incidence_age$metric=="Rate"&Incidence_age$location=="Global"])/

max(Incidence_age$val[Incidence_age$metric=="Number"&Incidence_age$location!="Global"])

p <- ggplot() +

geom_col(data = subset(Incidence_age,metric=='Number'&location!="Global"),

aes(x = age_new, y = val, fill = group)) +

geom_line(data = subset(Incidence_age,metric=='Rate'&location=="Global"),

aes(x = age_new, y = val/ad_factor, color = sex, group = sex), size = 1) +

geom_ribbon(data = subset(Incidence_age,metric=='Rate'&location=="Global"),

aes(x = age_new, ymin = lower/ad_factor, ymax = upper/ad_factor, fill = sex), alpha = 0.2) +

scale_fill_manual(name="Gender, SDI region (Number)",

values = c("a" = "#338a57",

"b" = "#5bb577",

"c" = "#90d191",

"d" = "#c9e9c2",

"e" = "#f1f9ec",

"f" = "#ca3352",

"g" = "#f3624d",

"h" = "#fda364",

"i" = "#fed67e",

"j" = "#ffffc1"),

labels=c(

"a"="Male,High SDI",

'b'='Male,High-middle SDI',

'c'='Male,Middle SDI',

'd'='Male,Low-middle SDI',

'e'='Male,Low SDI',

'f'='Female,High SDI',

'g'='Female,High-middle SDI',

'h'='Female,Middle SDI',

'i'='Female,Low-middle SDI',

'j'='Female,Low SDI'

)) +

scale_color_manual(name="Gender, Global Rate (per 100,000)",

values = c("Male" = "#006d2b", "Female" = "#31a255"),

labels = c(

'Male'='Male,Global',

'Female'='Female,Global'

)) +

scale_x_continuous(breaks = 1:20,

labels=c('<5 years','5-9 years','10-14 years','15-19 years','20-24 years',

'25-29 years','30-34 years','35-39 years','40-44 years','45-49 years',

'50-54 years','55-59 years','60-64 years','65-69 years','70-74 years',

'75-79 years','80-84 years','85-89 years','90-94 years','95+ years')) +

scale_y_continuous(sec.axis = sec_axis(~.*ad_factor, name = "Gender, Global Rate (per 100,000)",

labels = function(x) format(abs(x), scientific = FALSE)),

labels = function(x) format(abs(x), scientific = FALSE)) +

theme_bw() +

theme(legend.position = "right",

panel.grid.major = element_line(color = "gray90"),

panel.grid.minor = element_line(color = "gray95"),

axis.text.x = element_text(angle = 45, hjust = 1)) +

labs(title = "",

x = 'Age',

y = 'Number')

p

DALY_age <- IS %>%

filter(measure == "DALYs (Disability-Adjusted Life Years)" &

sex != "Both" &

age != "All ages" &

age != "Age-standardized" &

year == 2021) %>%

filter(location=="Global" |

location=="High SDI" |

location=="High-middle SDI" |

location=="Middle SDI" |

location=="Low-middle SDI" |

location=="Low SDI") %>%

mutate(val=ifelse(sex=="Male",val,-val),

lower=ifelse(sex=="Male",lower,-lower),

upper=ifelse(sex=="Male",upper,-upper)) %>%

mutate(group=ifelse(location=="High SDI"&sex=="Male","a",ifelse(

location=="High-middle SDI"&sex=="Male","b",ifelse(

location=="Middle SDI"&sex=="Male","c",ifelse(

location=="Low-middle SDI"&sex=="Male","d",ifelse(

location=="Low SDI"&sex=="Male","e",ifelse(

location=="High SDI"&sex=="Female","f",ifelse(

location=="High-middle SDI"&sex=="Female","g",ifelse(

location=="Middle SDI"&sex=="Female","h",ifelse(

location=="Low-middle SDI"&sex=="Female","i","j")))))))))) %>%

mutate(age_new=ifelse(age=="<5 years",1,ifelse(

age=="5-9 years",2,ifelse(

age=="10-14 years",3,ifelse(

age=="15-19 years",4,ifelse(

age=="20-24 years",5,ifelse(

age=="25-29 years",6,ifelse(

age=="30-34 years",7,ifelse(

age=="35-39 years",8,ifelse(

age=="40-44 years",9,ifelse(

age=="45-49 years",10,ifelse(

age=="50-54 years",11,ifelse(

age=="55-59 years",12,ifelse(

age=="60-64 years",13,ifelse(

age=="65-69 years",14,ifelse(

age=="70-74 years",15,ifelse(

age=="75-79 years",16,ifelse(

age=="80-84 years",17,ifelse(

age=="85-89 years",18,ifelse(

age=="90-94 years",19,20)))))))))))))))))))) %>%

dplyr::select(,c(2,3,4,5,7:11))

ad_factor <- max(Incidence_age$val[Incidence_age$metric=="Rate"&Incidence_age$location=="Global"])/

max(Incidence_age$val[Incidence_age$metric=="Number"&Incidence_age$location!="Global"])

p <- ggplot() +

geom_col(data = subset(Incidence_age,metric=='Number'&location!="Global"),

aes(x = age_new, y = val, fill = group)) +

geom_line(data = subset(Incidence_age,metric=='Rate'&location=="Global"),

aes(x = age_new, y = val/ad_factor, color = sex, group = sex), size = 1) +

geom_ribbon(data = subset(Incidence_age,metric=='Rate'&location=="Global"),

aes(x = age_new, ymin = lower/ad_factor, ymax = upper/ad_factor, fill = sex), alpha = 0.2) +

scale_fill_manual(name="Gender, SDI region (Number)",

values = c("a" = "#338a57",

"b" = "#5bb577",

"c" = "#90d191",

"d" = "#c9e9c2",

"e" = "#f1f9ec",

"f" = "#ca3352",

"g" = "#f3624d",

"h" = "#fda364",

"i" = "#fed67e",

"j" = "#ffffc1"),

labels=c(

"a"="Male,High SDI",

'b'='Male,High-middle SDI',

'c'='Male,Middle SDI',

'd'='Male,Low-middle SDI',

'e'='Male,Low SDI',

'f'='Female,High SDI',

'g'='Female,High-middle SDI',

'h'='Female,Middle SDI',

'i'='Female,Low-middle SDI',

'j'='Female,Low SDI'

)) +

scale_color_manual(name="Gender, Global Rate (per 100,000)",

values = c("Male" = "#006d2b", "Female" = "#31a255"),

labels = c(

'Male'='Male,Global',

'Female'='Female,Global'

)) +

scale_x_continuous(breaks = 1:20,

labels=c('<5 years','5-9 years','10-14 years','15-19 years','20-24 years',

'25-29 years','30-34 years','35-39 years','40-44 years','45-49 years',

'50-54 years','55-59 years','60-64 years','65-69 years','70-74 years',

'75-79 years','80-84 years','85-89 years','90-94 years','95+ years')) +

scale_y_continuous(sec.axis = sec_axis(~.*ad_factor, name = "Gender, Global Rate (per 100,000)",

labels = function(x) format(abs(x), scientific = FALSE)),

labels = function(x) format(abs(x), scientific = FALSE)) +

theme_bw() +

theme(legend.position = "right",

panel.grid.major = element_line(color = "gray90"),

panel.grid.minor = element_line(color = "gray95"),

axis.text.x = element_text(angle = 45, hjust = 1)) +

labs(title = "",

x = 'Age',

y = 'Number')

p

IS <- CLL_region %>%

dplyr::select(measure_name,location_name,metric_name,

sex_name,age_name,year,val,lower,upper) %>%

rename(measure=measure_name,

location=location_name,

metric=metric_name,

sex=sex_name,

age=age_name)

Incidence_age <- IS %>%

filter(measure == "Incidence" &

sex != "Both" &

age != "All ages" &

age != "Age-standardized" &

year == 2021) %>%

filter(location=="Global" |

location=="High SDI" |

location=="High-middle SDI" |

location=="Middle SDI" |

location=="Low-middle SDI" |

location=="Low SDI") %>%

mutate(val=ifelse(sex=="Male",val,-val),

lower=ifelse(sex=="Male",lower,-lower),

upper=ifelse(sex=="Male",upper,-upper)) %>%

mutate(group=ifelse(location=="High SDI"&sex=="Male","a",ifelse(

location=="High-middle SDI"&sex=="Male","b",ifelse(

location=="Middle SDI"&sex=="Male","c",ifelse(

location=="Low-middle SDI"&sex=="Male","d",ifelse(

location=="Low SDI"&sex=="Male","e",ifelse(

location=="High SDI"&sex=="Female","f",ifelse(

location=="High-middle SDI"&sex=="Female","g",ifelse(

location=="Middle SDI"&sex=="Female","h",ifelse(

location=="Low-middle SDI"&sex=="Female","i","j")))))))))) %>%

mutate(age_new=ifelse(age=="<5 years",1,ifelse(

age=="5-9 years",2,ifelse(

age=="10-14 years",3,ifelse(

age=="15-19 years",4,ifelse(

age=="20-24 years",5,ifelse(

age=="25-29 years",6,ifelse(

age=="30-34 years",7,ifelse(

age=="35-39 years",8,ifelse(

age=="40-44 years",9,ifelse(

age=="45-49 years",10,ifelse(

age=="50-54 years",11,ifelse(

age=="55-59 years",12,ifelse(

age=="60-64 years",13,ifelse(

age=="65-69 years",14,ifelse(

age=="70-74 years",15,ifelse(

age=="75-79 years",16,ifelse(

age=="80-84 years",17,ifelse(

age=="85-89 years",18,ifelse(

age=="90-94 years",19,20)))))))))))))))))))) %>%

dplyr::select(,c(2,3,4,5,7:11))

ad_factor <- max(Incidence_age$val[Incidence_age$metric=="Rate"&Incidence_age$location=="Global"])/

max(Incidence_age$val[Incidence_age$metric=="Number"&Incidence_age$location!="Global"])

p <- ggplot() +

geom_col(data = subset(Incidence_age,metric=='Number'&location!="Global"),

aes(x = age_new, y = val, fill = group)) +

geom_line(data = subset(Incidence_age,metric=='Rate'&location=="Global"),

aes(x = age_new, y = val/ad_factor, color = sex, group = sex), size = 1) +

geom_ribbon(data = subset(Incidence_age,metric=='Rate'&location=="Global"),

aes(x = age_new, ymin = lower/ad_factor, ymax = upper/ad_factor, fill = sex), alpha = 0.2) +

scale_fill_manual(name="Gender, SDI region (Number)",

values = c("a" = "#338a57",

"b" = "#5bb577",

"c" = "#90d191",

"d" = "#c9e9c2",

"e" = "#f1f9ec",

"f" = "#ca3352",

"g" = "#f3624d",

"h" = "#fda364",

"i" = "#fed67e",

"j" = "#ffffc1"),

labels=c(

"a"="Male,High SDI",

'b'='Male,High-middle SDI',

'c'='Male,Middle SDI',

'd'='Male,Low-middle SDI',

'e'='Male,Low SDI',

'f'='Female,High SDI',

'g'='Female,High-middle SDI',

'h'='Female,Middle SDI',

'i'='Female,Low-middle SDI',

'j'='Female,Low SDI'

)) +

scale_color_manual(name="Gender, Global Rate (per 100,000)",

values = c("Male" = "#006d2b", "Female" = "#31a255"),

labels = c(

'Male'='Male,Global',

'Female'='Female,Global'

)) +

scale_x_continuous(breaks = 1:20,

labels=c('<5 years','5-9 years','10-14 years','15-19 years','20-24 years',

'25-29 years','30-34 years','35-39 years','40-44 years','45-49 years',

'50-54 years','55-59 years','60-64 years','65-69 years','70-74 years',

'75-79 years','80-84 years','85-89 years','90-94 years','95+ years')) +

scale_y_continuous(sec.axis = sec_axis(~.*ad_factor, name = "Gender, Global Rate (per 100,000)",

labels = function(x) format(abs(x), scientific = FALSE)),

labels = function(x) format(abs(x), scientific = FALSE)) +

theme_bw() +

theme(legend.position = "right",

panel.grid.major = element_line(color = "gray90"),

panel.grid.minor = element_line(color = "gray95"),

axis.text.x = element_text(angle = 45, hjust = 1)) +

labs(title = "",

x = 'Age',

y = 'Number')

p

Prevalence_age <- IS %>%

filter(measure == "Prevalence" &

sex != "Both" &

age != "All ages" &

age != "Age-standardized" &

year == 2021) %>%

filter(location=="Global" |

location=="High SDI" |

location=="High-middle SDI" |

location=="Middle SDI" |

location=="Low-middle SDI" |

location=="Low SDI") %>%

mutate(val=ifelse(sex=="Male",val,-val),

lower=ifelse(sex=="Male",lower,-lower),

upper=ifelse(sex=="Male",upper,-upper)) %>%

mutate(group=ifelse(location=="High SDI"&sex=="Male","a",ifelse(

location=="High-middle SDI"&sex=="Male","b",ifelse(

location=="Middle SDI"&sex=="Male","c",ifelse(

location=="Low-middle SDI"&sex=="Male","d",ifelse(

location=="Low SDI"&sex=="Male","e",ifelse(

location=="High SDI"&sex=="Female","f",ifelse(

location=="High-middle SDI"&sex=="Female","g",ifelse(

location=="Middle SDI"&sex=="Female","h",ifelse(

location=="Low-middle SDI"&sex=="Female","i","j")))))))))) %>%

mutate(age_new=ifelse(age=="<5 years",1,ifelse(

age=="5-9 years",2,ifelse(

age=="10-14 years",3,ifelse(

age=="15-19 years",4,ifelse(

age=="20-24 years",5,ifelse(

age=="25-29 years",6,ifelse(

age=="30-34 years",7,ifelse(

age=="35-39 years",8,ifelse(

age=="40-44 years",9,ifelse(

age=="45-49 years",10,ifelse(

age=="50-54 years",11,ifelse(

age=="55-59 years",12,ifelse(

age=="60-64 years",13,ifelse(

age=="65-69 years",14,ifelse(

age=="70-74 years",15,ifelse(

age=="75-79 years",16,ifelse(

age=="80-84 years",17,ifelse(

age=="85-89 years",18,ifelse(

age=="90-94 years",19,20)))))))))))))))))))) %>%

dplyr::select(,c(2,3,4,5,7:11))

ad_factor <- max(Incidence_age$val[Incidence_age$metric=="Rate"&Incidence_age$location=="Global"])/

max(Incidence_age$val[Incidence_age$metric=="Number"&Incidence_age$location!="Global"])

p <- ggplot() +

geom_col(data = subset(Incidence_age,metric=='Number'&location!="Global"),

aes(x = age_new, y = val, fill = group)) +

geom_line(data = subset(Incidence_age,metric=='Rate'&location=="Global"),

aes(x = age_new, y = val/ad_factor, color = sex, group = sex), size = 1) +

geom_ribbon(data = subset(Incidence_age,metric=='Rate'&location=="Global"),

aes(x = age_new, ymin = lower/ad_factor, ymax = upper/ad_factor, fill = sex), alpha = 0.2) +

scale_fill_manual(name="Gender, SDI region (Number)",

values = c("a" = "#338a57",

"b" = "#5bb577",

"c" = "#90d191",

"d" = "#c9e9c2",

"e" = "#f1f9ec",

"f" = "#ca3352",

"g" = "#f3624d",

"h" = "#fda364",

"i" = "#fed67e",

"j" = "#ffffc1"),

labels=c(

"a"="Male,High SDI",

'b'='Male,High-middle SDI',

'c'='Male,Middle SDI',

'd'='Male,Low-middle SDI',

'e'='Male,Low SDI',

'f'='Female,High SDI',

'g'='Female,High-middle SDI',

'h'='Female,Middle SDI',

'i'='Female,Low-middle SDI',

'j'='Female,Low SDI'

)) +

scale_color_manual(name="Gender, Global Rate (per 100,000)",

values = c("Male" = "#006d2b", "Female" = "#31a255"),

labels = c(

'Male'='Male,Global',

'Female'='Female,Global'

)) +

scale_x_continuous(breaks = 1:20,

labels=c('<5 years','5-9 years','10-14 years','15-19 years','20-24 years',

'25-29 years','30-34 years','35-39 years','40-44 years','45-49 years',

'50-54 years','55-59 years','60-64 years','65-69 years','70-74 years',

'75-79 years','80-84 years','85-89 years','90-94 years','95+ years')) +

scale_y_continuous(sec.axis = sec_axis(~.*ad_factor, name = "Gender, Global Rate (per 100,000)",

labels = function(x) format(abs(x), scientific = FALSE)),

labels = function(x) format(abs(x), scientific = FALSE)) +

theme_bw() +

theme(legend.position = "right",

panel.grid.major = element_line(color = "gray90"),

panel.grid.minor = element_line(color = "gray95"),

axis.text.x = element_text(angle = 45, hjust = 1)) +

labs(title = "",

x = 'Age',

y = 'Number')

p

Deaths_age <- IS %>%

filter(measure == "Deaths" &

sex != "Both" &

age != "All ages" &

age != "Age-standardized" &

year == 2021) %>%

filter(location=="Global" |

location=="High SDI" |

location=="High-middle SDI" |

location=="Middle SDI" |

location=="Low-middle SDI" |

location=="Low SDI") %>%

mutate(val=ifelse(sex=="Male",val,-val),

lower=ifelse(sex=="Male",lower,-lower),

upper=ifelse(sex=="Male",upper,-upper)) %>%

mutate(group=ifelse(location=="High SDI"&sex=="Male","a",ifelse(

location=="High-middle SDI"&sex=="Male","b",ifelse(

location=="Middle SDI"&sex=="Male","c",ifelse(

location=="Low-middle SDI"&sex=="Male","d",ifelse(

location=="Low SDI"&sex=="Male","e",ifelse(

location=="High SDI"&sex=="Female","f",ifelse(

location=="High-middle SDI"&sex=="Female","g",ifelse(

location=="Middle SDI"&sex=="Female","h",ifelse(

location=="Low-middle SDI"&sex=="Female","i","j")))))))))) %>%

mutate(age_new=ifelse(age=="<5 years",1,ifelse(

age=="5-9 years",2,ifelse(

age=="10-14 years",3,ifelse(

age=="15-19 years",4,ifelse(

age=="20-24 years",5,ifelse(

age=="25-29 years",6,ifelse(

age=="30-34 years",7,ifelse(

age=="35-39 years",8,ifelse(

age=="40-44 years",9,ifelse(

age=="45-49 years",10,ifelse(

age=="50-54 years",11,ifelse(

age=="55-59 years",12,ifelse(

age=="60-64 years",13,ifelse(

age=="65-69 years",14,ifelse(

age=="70-74 years",15,ifelse(

age=="75-79 years",16,ifelse(

age=="80-84 years",17,ifelse(

age=="85-89 years",18,ifelse(

age=="90-94 years",19,20)))))))))))))))))))) %>%

dplyr::select(,c(2,3,4,5,7:11))

ad_factor <- max(Incidence_age$val[Incidence_age$metric=="Rate"&Incidence_age$location=="Global"])/

max(Incidence_age$val[Incidence_age$metric=="Number"&Incidence_age$location!="Global"])

p <- ggplot() +

geom_col(data = subset(Incidence_age,metric=='Number'&location!="Global"),

aes(x = age_new, y = val, fill = group)) +

geom_line(data = subset(Incidence_age,metric=='Rate'&location=="Global"),

aes(x = age_new, y = val/ad_factor, color = sex, group = sex), size = 1) +

geom_ribbon(data = subset(Incidence_age,metric=='Rate'&location=="Global"),

aes(x = age_new, ymin = lower/ad_factor, ymax = upper/ad_factor, fill = sex), alpha = 0.2) +

scale_fill_manual(name="Gender, SDI region (Number)",

values = c("a" = "#338a57",

"b" = "#5bb577",

"c" = "#90d191",

"d" = "#c9e9c2",

"e" = "#f1f9ec",

"f" = "#ca3352",

"g" = "#f3624d",

"h" = "#fda364",

"i" = "#fed67e",

"j" = "#ffffc1"),

labels=c(

"a"="Male,High SDI",

'b'='Male,High-middle SDI',

'c'='Male,Middle SDI',

'd'='Male,Low-middle SDI',

'e'='Male,Low SDI',

'f'='Female,High SDI',

'g'='Female,High-middle SDI',

'h'='Female,Middle SDI',

'i'='Female,Low-middle SDI',

'j'='Female,Low SDI'

)) +

scale_color_manual(name="Gender, Global Rate (per 100,000)",

values = c("Male" = "#006d2b", "Female" = "#31a255"),

labels = c(

'Male'='Male,Global',

'Female'='Female,Global'

)) +

scale_x_continuous(breaks = 1:20,

labels=c('<5 years','5-9 years','10-14 years','15-19 years','20-24 years',

'25-29 years','30-34 years','35-39 years','40-44 years','45-49 years',

'50-54 years','55-59 years','60-64 years','65-69 years','70-74 years',

'75-79 years','80-84 years','85-89 years','90-94 years','95+ years')) +

scale_y_continuous(sec.axis = sec_axis(~.*ad_factor, name = "Gender, Global Rate (per 100,000)",

labels = function(x) format(abs(x), scientific = FALSE)),

labels = function(x) format(abs(x), scientific = FALSE)) +

theme_bw() +

theme(legend.position = "right",

panel.grid.major = element_line(color = "gray90"),

panel.grid.minor = element_line(color = "gray95"),

axis.text.x = element_text(angle = 45, hjust = 1)) +

labs(title = "",

x = 'Age',

y = 'Number')

p

DALY_age <- IS %>%

filter(measure == "DALYs (Disability-Adjusted Life Years)" &

sex != "Both" &

age != "All ages" &

age != "Age-standardized" &

year == 2021) %>%

filter(location=="Global" |

location=="High SDI" |

location=="High-middle SDI" |

location=="Middle SDI" |

location=="Low-middle SDI" |

location=="Low SDI") %>%

mutate(val=ifelse(sex=="Male",val,-val),

lower=ifelse(sex=="Male",lower,-lower),

upper=ifelse(sex=="Male",upper,-upper)) %>%

mutate(group=ifelse(location=="High SDI"&sex=="Male","a",ifelse(

location=="High-middle SDI"&sex=="Male","b",ifelse(

location=="Middle SDI"&sex=="Male","c",ifelse(

location=="Low-middle SDI"&sex=="Male","d",ifelse(

location=="Low SDI"&sex=="Male","e",ifelse(

location=="High SDI"&sex=="Female","f",ifelse(

location=="High-middle SDI"&sex=="Female","g",ifelse(

location=="Middle SDI"&sex=="Female","h",ifelse(

location=="Low-middle SDI"&sex=="Female","i","j")))))))))) %>%

mutate(age_new=ifelse(age=="<5 years",1,ifelse(

age=="5-9 years",2,ifelse(

age=="10-14 years",3,ifelse(

age=="15-19 years",4,ifelse(

age=="20-24 years",5,ifelse(

age=="25-29 years",6,ifelse(

age=="30-34 years",7,ifelse(

age=="35-39 years",8,ifelse(

age=="40-44 years",9,ifelse(

age=="45-49 years",10,ifelse(

age=="50-54 years",11,ifelse(

age=="55-59 years",12,ifelse(

age=="60-64 years",13,ifelse(

age=="65-69 years",14,ifelse(

age=="70-74 years",15,ifelse(

age=="75-79 years",16,ifelse(

age=="80-84 years",17,ifelse(

age=="85-89 years",18,ifelse(

age=="90-94 years",19,20)))))))))))))))))))) %>%

dplyr::select(,c(2,3,4,5,7:11))

ad_factor <- max(Incidence_age$val[Incidence_age$metric=="Rate"&Incidence_age$location=="Global"])/

max(Incidence_age$val[Incidence_age$metric=="Number"&Incidence_age$location!="Global"])

p <- ggplot() +

geom_col(data = subset(Incidence_age,metric=='Number'&location!="Global"),

aes(x = age_new, y = val, fill = group)) +

geom_line(data = subset(Incidence_age,metric=='Rate'&location=="Global"),

aes(x = age_new, y = val/ad_factor, color = sex, group = sex), size = 1) +

geom_ribbon(data = subset(Incidence_age,metric=='Rate'&location=="Global"),

aes(x = age_new, ymin = lower/ad_factor, ymax = upper/ad_factor, fill = sex), alpha = 0.2) +

scale_fill_manual(name="Gender, SDI region (Number)",

values = c("a" = "#338a57",

"b" = "#5bb577",

"c" = "#90d191",

"d" = "#c9e9c2",

"e" = "#f1f9ec",

"f" = "#ca3352",

"g" = "#f3624d",

"h" = "#fda364",

"i" = "#fed67e",

"j" = "#ffffc1"),

labels=c(

"a"="Male,High SDI",

'b'='Male,High-middle SDI',

'c'='Male,Middle SDI',

'd'='Male,Low-middle SDI',

'e'='Male,Low SDI',

'f'='Female,High SDI',

'g'='Female,High-middle SDI',

'h'='Female,Middle SDI',

'i'='Female,Low-middle SDI',

'j'='Female,Low SDI'

)) +

scale_color_manual(name="Gender, Global Rate (per 100,000)",

values = c("Male" = "#006d2b", "Female" = "#31a255"),

labels = c(

'Male'='Male,Global',

'Female'='Female,Global'

)) +

scale_x_continuous(breaks = 1:20,

labels=c('<5 years','5-9 years','10-14 years','15-19 years','20-24 years',

'25-29 years','30-34 years','35-39 years','40-44 years','45-49 years',

'50-54 years','55-59 years','60-64 years','65-69 years','70-74 years',

'75-79 years','80-84 years','85-89 years','90-94 years','95+ years')) +

scale_y_continuous(sec.axis = sec_axis(~.*ad_factor, name = "Gender, Global Rate (per 100,000)",

labels = function(x) format(abs(x), scientific = FALSE)),

labels = function(x) format(abs(x), scientific = FALSE)) +

theme_bw() +

theme(legend.position = "right",

panel.grid.major = element_line(color = "gray90"),

panel.grid.minor = element_line(color = "gray95"),

axis.text.x = element_text(angle = 45, hjust = 1)) +

labs(title = "",

x = 'Age',

y = 'Number')

p

IS <- MM_region %>%

dplyr::select(measure_name,location_name,metric_name,

sex_name,age_name,year,val,lower,upper) %>%

rename(measure=measure_name,

location=location_name,

metric=metric_name,

sex=sex_name,

age=age_name)

Incidence_age <- IS %>%

filter(measure == "Incidence" &

sex != "Both" &

age != "All ages" &

age != "Age-standardized" &

year == 2021) %>%

filter(location=="Global" |

location=="High SDI" |

location=="High-middle SDI" |

location=="Middle SDI" |

location=="Low-middle SDI" |

location=="Low SDI") %>%

mutate(val=ifelse(sex=="Male",val,-val),

lower=ifelse(sex=="Male",lower,-lower),

upper=ifelse(sex=="Male",upper,-upper)) %>%

mutate(group=ifelse(location=="High SDI"&sex=="Male","a",ifelse(

location=="High-middle SDI"&sex=="Male","b",ifelse(

location=="Middle SDI"&sex=="Male","c",ifelse(

location=="Low-middle SDI"&sex=="Male","d",ifelse(

location=="Low SDI"&sex=="Male","e",ifelse(

location=="High SDI"&sex=="Female","f",ifelse(

location=="High-middle SDI"&sex=="Female","g",ifelse(

location=="Middle SDI"&sex=="Female","h",ifelse(

location=="Low-middle SDI"&sex=="Female","i","j")))))))))) %>%

mutate(age_new=ifelse(age=="<5 years",1,ifelse(

age=="5-9 years",2,ifelse(

age=="10-14 years",3,ifelse(

age=="15-19 years",4,ifelse(

age=="20-24 years",5,ifelse(

age=="25-29 years",6,ifelse(

age=="30-34 years",7,ifelse(

age=="35-39 years",8,ifelse(

age=="40-44 years",9,ifelse(

age=="45-49 years",10,ifelse(

age=="50-54 years",11,ifelse(

age=="55-59 years",12,ifelse(

age=="60-64 years",13,ifelse(

age=="65-69 years",14,ifelse(

age=="70-74 years",15,ifelse(

age=="75-79 years",16,ifelse(

age=="80-84 years",17,ifelse(

age=="85-89 years",18,ifelse(

age=="90-94 years",19,20)))))))))))))))))))) %>%

dplyr::select(,c(2,3,4,5,7:11))

ad_factor <- max(Incidence_age$val[Incidence_age$metric=="Rate"&Incidence_age$location=="Global"])/

max(Incidence_age$val[Incidence_age$metric=="Number"&Incidence_age$location!="Global"])

p <- ggplot() +

geom_col(data = subset(Incidence_age,metric=='Number'&location!="Global"),

aes(x = age_new, y = val, fill = group)) +

geom_line(data = subset(Incidence_age,metric=='Rate'&location=="Global"),

aes(x = age_new, y = val/ad_factor, color = sex, group = sex), size = 1) +

geom_ribbon(data = subset(Incidence_age,metric=='Rate'&location=="Global"),

aes(x = age_new, ymin = lower/ad_factor, ymax = upper/ad_factor, fill = sex), alpha = 0.2) +

scale_fill_manual(name="Gender, SDI region (Number)",

values = c("a" = "#338a57",

"b" = "#5bb577",

"c" = "#90d191",

"d" = "#c9e9c2",

"e" = "#f1f9ec",

"f" = "#ca3352",

"g" = "#f3624d",

"h" = "#fda364",

"i" = "#fed67e",

"j" = "#ffffc1"),

labels=c(

"a"="Male,High SDI",

'b'='Male,High-middle SDI',

'c'='Male,Middle SDI',

'd'='Male,Low-middle SDI',

'e'='Male,Low SDI',

'f'='Female,High SDI',

'g'='Female,High-middle SDI',

'h'='Female,Middle SDI',

'i'='Female,Low-middle SDI',

'j'='Female,Low SDI'

)) +

scale_color_manual(name="Gender, Global Rate (per 100,000)",

values = c("Male" = "#006d2b", "Female" = "#31a255"),

labels = c(

'Male'='Male,Global',

'Female'='Female,Global'

)) +

scale_x_continuous(breaks = 1:20,

labels=c('<5 years','5-9 years','10-14 years','15-19 years','20-24 years',

'25-29 years','30-34 years','35-39 years','40-44 years','45-49 years',

'50-54 years','55-59 years','60-64 years','65-69 years','70-74 years',

'75-79 years','80-84 years','85-89 years','90-94 years','95+ years')) +

scale_y_continuous(sec.axis = sec_axis(~.*ad_factor, name = "Gender, Global Rate (per 100,000)",

labels = function(x) format(abs(x), scientific = FALSE)),

labels = function(x) format(abs(x), scientific = FALSE)) +

theme_bw() +

theme(legend.position = "right",

panel.grid.major = element_line(color = "gray90"),

panel.grid.minor = element_line(color = "gray95"),

axis.text.x = element_text(angle = 45, hjust = 1)) +

labs(title = "",

x = 'Age',

y = 'Number')

p

Prevalence_age <- IS %>%

filter(measure == "Prevalence" &

sex != "Both" &

age != "All ages" &

age != "Age-standardized" &

year == 2021) %>%

filter(location=="Global" |

location=="High SDI" |

location=="High-middle SDI" |

location=="Middle SDI" |

location=="Low-middle SDI" |

location=="Low SDI") %>%

mutate(val=ifelse(sex=="Male",val,-val),

lower=ifelse(sex=="Male",lower,-lower),

upper=ifelse(sex=="Male",upper,-upper)) %>%

mutate(group=ifelse(location=="High SDI"&sex=="Male","a",ifelse(

location=="High-middle SDI"&sex=="Male","b",ifelse(

location=="Middle SDI"&sex=="Male","c",ifelse(

location=="Low-middle SDI"&sex=="Male","d",ifelse(

location=="Low SDI"&sex=="Male","e",ifelse(

location=="High SDI"&sex=="Female","f",ifelse(

location=="High-middle SDI"&sex=="Female","g",ifelse(

location=="Middle SDI"&sex=="Female","h",ifelse(

location=="Low-middle SDI"&sex=="Female","i","j")))))))))) %>%

mutate(age_new=ifelse(age=="<5 years",1,ifelse(

age=="5-9 years",2,ifelse(

age=="10-14 years",3,ifelse(

age=="15-19 years",4,ifelse(

age=="20-24 years",5,ifelse(

age=="25-29 years",6,ifelse(

age=="30-34 years",7,ifelse(

age=="35-39 years",8,ifelse(

age=="40-44 years",9,ifelse(

age=="45-49 years",10,ifelse(

age=="50-54 years",11,ifelse(

age=="55-59 years",12,ifelse(

age=="60-64 years",13,ifelse(

age=="65-69 years",14,ifelse(

age=="70-74 years",15,ifelse(

age=="75-79 years",16,ifelse(

age=="80-84 years",17,ifelse(

age=="85-89 years",18,ifelse(

age=="90-94 years",19,20)))))))))))))))))))) %>%

dplyr::select(,c(2,3,4,5,7:11))

ad_factor <- max(Incidence_age$val[Incidence_age$metric=="Rate"&Incidence_age$location=="Global"])/

max(Incidence_age$val[Incidence_age$metric=="Number"&Incidence_age$location!="Global"])

p <- ggplot() +

geom_col(data = subset(Incidence_age,metric=='Number'&location!="Global"),

aes(x = age_new, y = val, fill = group)) +

geom_line(data = subset(Incidence_age,metric=='Rate'&location=="Global"),

aes(x = age_new, y = val/ad_factor, color = sex, group = sex), size = 1) +

geom_ribbon(data = subset(Incidence_age,metric=='Rate'&location=="Global"),

aes(x = age_new, ymin = lower/ad_factor, ymax = upper/ad_factor, fill = sex), alpha = 0.2) +

scale_fill_manual(name="Gender, SDI region (Number)",

values = c("a" = "#338a57",

"b" = "#5bb577",

"c" = "#90d191",

"d" = "#c9e9c2",

"e" = "#f1f9ec",

"f" = "#ca3352",

"g" = "#f3624d",

"h" = "#fda364",

"i" = "#fed67e",

"j" = "#ffffc1"),

labels=c(

"a"="Male,High SDI",

'b'='Male,High-middle SDI',

'c'='Male,Middle SDI',

'd'='Male,Low-middle SDI',

'e'='Male,Low SDI',

'f'='Female,High SDI',

'g'='Female,High-middle SDI',

'h'='Female,Middle SDI',

'i'='Female,Low-middle SDI',

'j'='Female,Low SDI'

)) +

scale_color_manual(name="Gender, Global Rate (per 100,000)",

values = c("Male" = "#006d2b", "Female" = "#31a255"),

labels = c(

'Male'='Male,Global',

'Female'='Female,Global'

)) +

scale_x_continuous(breaks = 1:20,

labels=c('<5 years','5-9 years','10-14 years','15-19 years','20-24 years',

'25-29 years','30-34 years','35-39 years','40-44 years','45-49 years',

'50-54 years','55-59 years','60-64 years','65-69 years','70-74 years',

'75-79 years','80-84 years','85-89 years','90-94 years','95+ years')) +

scale_y_continuous(sec.axis = sec_axis(~.*ad_factor, name = "Gender, Global Rate (per 100,000)",

labels = function(x) format(abs(x), scientific = FALSE)),

labels = function(x) format(abs(x), scientific = FALSE)) +

theme_bw() +

theme(legend.position = "right",

panel.grid.major = element_line(color = "gray90"),

panel.grid.minor = element_line(color = "gray95"),

axis.text.x = element_text(angle = 45, hjust = 1)) +

labs(title = "",

x = 'Age',

y = 'Number')

p

Deaths_age <- IS %>%

filter(measure == "Deaths" &

sex != "Both" &

age != "All ages" &

age != "Age-standardized" &

year == 2021) %>%

filter(location=="Global" |

location=="High SDI" |

location=="High-middle SDI" |

location=="Middle SDI" |

location=="Low-middle SDI" |

location=="Low SDI") %>%

mutate(val=ifelse(sex=="Male",val,-val),

lower=ifelse(sex=="Male",lower,-lower),

upper=ifelse(sex=="Male",upper,-upper)) %>%

mutate(group=ifelse(location=="High SDI"&sex=="Male","a",ifelse(

location=="High-middle SDI"&sex=="Male","b",ifelse(

location=="Middle SDI"&sex=="Male","c",ifelse(

location=="Low-middle SDI"&sex=="Male","d",ifelse(

location=="Low SDI"&sex=="Male","e",ifelse(

location=="High SDI"&sex=="Female","f",ifelse(

location=="High-middle SDI"&sex=="Female","g",ifelse(

location=="Middle SDI"&sex=="Female","h",ifelse(

location=="Low-middle SDI"&sex=="Female","i","j")))))))))) %>%

mutate(age_new=ifelse(age=="<5 years",1,ifelse(

age=="5-9 years",2,ifelse(

age=="10-14 years",3,ifelse(

age=="15-19 years",4,ifelse(

age=="20-24 years",5,ifelse(

age=="25-29 years",6,ifelse(

age=="30-34 years",7,ifelse(

age=="35-39 years",8,ifelse(

age=="40-44 years",9,ifelse(

age=="45-49 years",10,ifelse(

age=="50-54 years",11,ifelse(

age=="55-59 years",12,ifelse(

age=="60-64 years",13,ifelse(

age=="65-69 years",14,ifelse(

age=="70-74 years",15,ifelse(

age=="75-79 years",16,ifelse(

age=="80-84 years",17,ifelse(

age=="85-89 years",18,ifelse(

age=="90-94 years",19,20)))))))))))))))))))) %>%

dplyr::select(,c(2,3,4,5,7:11))

ad_factor <- max(Incidence_age$val[Incidence_age$metric=="Rate"&Incidence_age$location=="Global"])/

max(Incidence_age$val[Incidence_age$metric=="Number"&Incidence_age$location!="Global"])

p <- ggplot() +

geom_col(data = subset(Incidence_age,metric=='Number'&location!="Global"),

aes(x = age_new, y = val, fill = group)) +

geom_line(data = subset(Incidence_age,metric=='Rate'&location=="Global"),

aes(x = age_new, y = val/ad_factor, color = sex, group = sex), size = 1) +

geom_ribbon(data = subset(Incidence_age,metric=='Rate'&location=="Global"),

aes(x = age_new, ymin = lower/ad_factor, ymax = upper/ad_factor, fill = sex), alpha = 0.2) +

scale_fill_manual(name="Gender, SDI region (Number)",

values = c("a" = "#338a57",

"b" = "#5bb577",

"c" = "#90d191",

"d" = "#c9e9c2",

"e" = "#f1f9ec",

"f" = "#ca3352",

"g" = "#f3624d",

"h" = "#fda364",

"i" = "#fed67e",

"j" = "#ffffc1"),

labels=c(

"a"="Male,High SDI",

'b'='Male,High-middle SDI',

'c'='Male,Middle SDI',

'd'='Male,Low-middle SDI',

'e'='Male,Low SDI',

'f'='Female,High SDI',

'g'='Female,High-middle SDI',

'h'='Female,Middle SDI',

'i'='Female,Low-middle SDI',

'j'='Female,Low SDI'

)) +

scale_color_manual(name="Gender, Global Rate (per 100,000)",

values = c("Male" = "#006d2b", "Female" = "#31a255"),

labels = c(

'Male'='Male,Global',

'Female'='Female,Global'

)) +

scale_x_continuous(breaks = 1:20,

labels=c('<5 years','5-9 years','10-14 years','15-19 years','20-24 years',

'25-29 years','30-34 years','35-39 years','40-44 years','45-49 years',

'50-54 years','55-59 years','60-64 years','65-69 years','70-74 years',

'75-79 years','80-84 years','85-89 years','90-94 years','95+ years')) +

scale_y_continuous(sec.axis = sec_axis(~.*ad_factor, name = "Gender, Global Rate (per 100,000)",

labels = function(x) format(abs(x), scientific = FALSE)),

labels = function(x) format(abs(x), scientific = FALSE)) +

theme_bw() +

theme(legend.position = "right",

panel.grid.major = element_line(color = "gray90"),

panel.grid.minor = element_line(color = "gray95"),

axis.text.x = element_text(angle = 45, hjust = 1)) +

labs(title = "",

x = 'Age',

y = 'Number')

p

DALY_age <- IS %>%

filter(measure == "DALYs (Disability-Adjusted Life Years)" &

sex != "Both" &

age != "All ages" &

age != "Age-standardized" &

year == 2021) %>%

filter(location=="Global" |

location=="High SDI" |

location=="High-middle SDI" |

location=="Middle SDI" |

location=="Low-middle SDI" |

location=="Low SDI") %>%

mutate(val=ifelse(sex=="Male",val,-val),

lower=ifelse(sex=="Male",lower,-lower),

upper=ifelse(sex=="Male",upper,-upper)) %>%

mutate(group=ifelse(location=="High SDI"&sex=="Male","a",ifelse(

location=="High-middle SDI"&sex=="Male","b",ifelse(

location=="Middle SDI"&sex=="Male","c",ifelse(

location=="Low-middle SDI"&sex=="Male","d",ifelse(

location=="Low SDI"&sex=="Male","e",ifelse(

location=="High SDI"&sex=="Female","f",ifelse(

location=="High-middle SDI"&sex=="Female","g",ifelse(

location=="Middle SDI"&sex=="Female","h",ifelse(

location=="Low-middle SDI"&sex=="Female","i","j")))))))))) %>%

mutate(age_new=ifelse(age=="<5 years",1,ifelse(

age=="5-9 years",2,ifelse(

age=="10-14 years",3,ifelse(

age=="15-19 years",4,ifelse(

age=="20-24 years",5,ifelse(

age=="25-29 years",6,ifelse(

age=="30-34 years",7,ifelse(

age=="35-39 years",8,ifelse(

age=="40-44 years",9,ifelse(

age=="45-49 years",10,ifelse(

age=="50-54 years",11,ifelse(

age=="55-59 years",12,ifelse(

age=="60-64 years",13,ifelse(

age=="65-69 years",14,ifelse(

age=="70-74 years",15,ifelse(

age=="75-79 years",16,ifelse(

age=="80-84 years",17,ifelse(

age=="85-89 years",18,ifelse(

age=="90-94 years",19,20)))))))))))))))))))) %>%

dplyr::select(,c(2,3,4,5,7:11))

ad_factor <- max(Incidence_age$val[Incidence_age$metric=="Rate"&Incidence_age$location=="Global"])/

max(Incidence_age$val[Incidence_age$metric=="Number"&Incidence_age$location!="Global"])

p <- ggplot() +

geom_col(data = subset(Incidence_age,metric=='Number'&location!="Global"),

aes(x = age_new, y = val, fill = group)) +

geom_line(data = subset(Incidence_age,metric=='Rate'&location=="Global"),

aes(x = age_new, y = val/ad_factor, color = sex, group = sex), size = 1) +

geom_ribbon(data = subset(Incidence_age,metric=='Rate'&location=="Global"),

aes(x = age_new, ymin = lower/ad_factor, ymax = upper/ad_factor, fill = sex), alpha = 0.2) +

scale_fill_manual(name="Gender, SDI region (Number)",

values = c("a" = "#338a57",

"b" = "#5bb577",

"c" = "#90d191",

"d" = "#c9e9c2",

"e" = "#f1f9ec",

"f" = "#ca3352",

"g" = "#f3624d",

"h" = "#fda364",

"i" = "#fed67e",

"j" = "#ffffc1"),

labels=c(

"a"="Male,High SDI",

'b'='Male,High-middle SDI',

'c'='Male,Middle SDI',

'd'='Male,Low-middle SDI',

'e'='Male,Low SDI',

'f'='Female,High SDI',

'g'='Female,High-middle SDI',

'h'='Female,Middle SDI',

'i'='Female,Low-middle SDI',

'j'='Female,Low SDI'

)) +

scale_color_manual(name="Gender, Global Rate (per 100,000)",

values = c("Male" = "#006d2b", "Female" = "#31a255"),

labels = c(

'Male'='Male,Global',

'Female'='Female,Global'

)) +

scale_x_continuous(breaks = 1:20,

labels=c('<5 years','5-9 years','10-14 years','15-19 years','20-24 years',

'25-29 years','30-34 years','35-39 years','40-44 years','45-49 years',

'50-54 years','55-59 years','60-64 years','65-69 years','70-74 years',

'75-79 years','80-84 years','85-89 years','90-94 years','95+ years')) +

scale_y_continuous(sec.axis = sec_axis(~.*ad_factor, name = "Gender, Global Rate (per 100,000)",

labels = function(x) format(abs(x), scientific = FALSE)),

labels = function(x) format(abs(x), scientific = FALSE)) +

theme_bw() +

theme(legend.position = "right",

panel.grid.major = element_line(color = "gray90"),

panel.grid.minor = element_line(color = "gray95"),

axis.text.x = element_text(angle = 45, hjust = 1)) +

labs(title = "",

x = 'Age',

y = 'Number')

p

#Figure 6; Figure 14

library(reshape)

library(ggplot2)

library(ggrepel)

library(readxl)

IBD_region22 <- read.csv('HL_region.csv',header = T)

order_SDI <- read.csv('order_SDI.csv',header = F)

SDI_2021<-read.csv("SDI_2021.csv",header = T)

SDI_2021<-SDI_2021[,-1]

SDI_2021 <- melt(SDI_2021,id.vars ='Location')

SDI_2021$variable <- as.numeric(gsub('\\X',replacement = '', SDI_2021$variable))

names(SDI_2021) <- c('location','year','SDI')

SDI_2021$location[which(SDI_2021$location =='Central sub-Saharan Africa')] <-'Central Sub-Saharan Africa'

SDI_2021$location[which(SDI_2021$location =='Eastern sub-Saharan Africa')] <-'Eastern Sub-Saharan Africa'

SDI_2021$location[which(SDI_2021$location =='Southern sub-Saharan Africa')] <-'Southern Sub-Saharan Africa'

SDI_2021$location[which(SDI_2021$location =='Western sub-Saharan Africa')] <-'Western Sub-Saharan Africa'

### ASDR#####

IBD_ASMR <- subset(IBD_region22, IBD_region22$age_name=='Age-standardized' &

IBD_region22$metric_name== 'Rate' &

IBD_region22$measure_name=='Deaths'&

IBD_region22$sex_name=="Both")

IBD_ASMR <- IBD_ASMR[,c(5,14,15)]

names(IBD_ASMR)[3] <- 'ASMR'

names(IBD_ASMR)[1] <- 'location'

IBD_ASMR_SDI <- merge(IBD_ASMR,SDI_2021,by=c('location','year'))

IBD_ASMR_SDI$location <- factor(IBD_ASMR_SDI$location,

levels=order_SDI$V1,

ordered=TRUE)

write.csv(IBD_ASMR_SDI,"22_ASDR_SDI.csv")

size_breaks <- seq(min(IBD_ASMR_SDI$year), max(IBD_ASMR_SDI$year), by = 5)

size_labels <- size_breaks

ggplot(IBD_ASMR_SDI, aes(SDI,ASMR)) + geom_point(aes(color = location, shape= location,size=year))+

scale_shape_manual(values = 1:22) +

labs(x = "Socio-Demographic Index",

y = "ASMR(per 100000)") +

geom_smooth(colour='black',stat = "smooth",method='loess',se=F,span=0.5)+

scale_size_continuous(breaks = size_breaks, labels = size_labels,range = c(1, 3))

complete_data <- na.omit(IBD_ASMR_SDI)

cor.test(complete_data$ASMR, complete_data$SDI)

### ASIR#####

IBD_ASMR <- subset(IBD_region22, IBD_region22$age_name=='Age-standardized' &

IBD_region22$metric_name== 'Rate' &

IBD_region22$measure_name=='Incidence'&

IBD_region22$sex_name=="Both")

IBD_ASMR <- IBD_ASMR[,c(5,14,15)]

names(IBD_ASMR)[3] <- 'ASMR'

names(IBD_ASMR)[1] <- 'location'

IBD_ASMR_SDI <- merge(IBD_ASMR,SDI_2021,by=c('location','year'))

IBD_ASMR_SDI$location <- factor(IBD_ASMR_SDI$location,

levels=order_SDI$V1,

ordered=TRUE)

write.csv(IBD_ASMR_SDI,"22_ASIR_SDI.csv")

size_breaks <- seq(min(IBD_ASMR_SDI$year), max(IBD_ASMR_SDI$year), by = 5)

size_labels <- size_breaks

ggplot(IBD_ASMR_SDI, aes(SDI,ASMR)) + geom_point(aes(color = location, shape= location,size=year))+

scale_shape_manual(values = 1:22) +

labs(x = "Socio-Demographic Index",

y = "ASMR(per 100000") +

geom_smooth(colour='black',stat = "smooth",method='loess',se=F,span=0.5)+

scale_size_continuous(breaks = size_breaks, labels = size_labels,range = c(1, 3))

complete_data <- na.omit(IBD_ASMR_SDI)

cor.test(complete_data$ASMR, complete_data$SDI)

### ASPR#####

IBD_ASMR <- subset(IBD_region22, IBD_region22$age_name=='Age-standardized' &

IBD_region22$metric_name== 'Rate' &

IBD_region22$measure_name=='Prevalence'&

IBD_region22$sex_name=="Both")

IBD_ASMR <- IBD_ASMR[,c(5,14,15)]

names(IBD_ASMR)[3] <- 'ASMR'

names(IBD_ASMR)[1] <- 'location'

IBD_ASMR_SDI <- merge(IBD_ASMR,SDI_2021,by=c('location','year'))

IBD_ASMR_SDI$location <- factor(IBD_ASMR_SDI$location,

levels=order_SDI$V1,

ordered=TRUE)

write.csv(IBD_ASMR_SDI,"22_ASPR_SDI.csv")

size_breaks <- seq(min(IBD_ASMR_SDI$year), max(IBD_ASMR_SDI$year), by = 5)

size_labels <- size_breaks

ggplot(IBD_ASMR_SDI, aes(SDI,ASMR)) + geom_point(aes(color = location, shape= location,size=year))+

scale_shape_manual(values = 1:22) +

labs(x = "Socio-Demographic Index",

y = "ASMR(per 100000") +

geom_smooth(colour='black',stat = "smooth",method='loess',se=F,span=0.5)+

scale_size_continuous(breaks = size_breaks, labels = size_labels,range = c(1, 3))

complete_data <- na.omit(IBD_ASMR_SDI)

cor.test(complete_data$ASMR, complete_data$SDI)

### ASDALYsR#####

IBD_ASMR <- subset(IBD_region22, IBD_region22$age_name=='Age-standardized' &

IBD_region22$metric_name== 'Rate' &

IBD_region22$measure_name=='DALYs (Disability-Adjusted Life Years)'&

IBD_region22$sex_name=="Both")

IBD_ASMR <- IBD_ASMR[,c(5,14,15)]

names(IBD_ASMR)[3] <- 'ASMR'

names(IBD_ASMR)[1] <- 'location'

IBD_ASMR_SDI <- merge(IBD_ASMR,SDI_2021,by=c('location','year'))

IBD_ASMR_SDI$location <- factor(IBD_ASMR_SDI$location,

levels=order_SDI$V1,

ordered=TRUE)

write.csv(IBD_ASMR_SDI,"22_ASDALYsR_SDI.csv")

size_breaks <- seq(min(IBD_ASMR_SDI$year), max(IBD_ASMR_SDI$year), by = 5)

size_labels <- size_breaks

ggplot(IBD_ASMR_SDI, aes(SDI,ASMR)) + geom_point(aes(color = location, shape= location,size=year))+

scale_shape_manual(values = 1:22) +

labs(x = "Socio-Demographic Index",

y = "ASMR(per 100000") +

geom_smooth(colour='black',stat = "smooth",method='loess',se=F,span=0.5)+

scale_size_continuous(breaks = size_breaks, labels = size_labels,range = c(1, 3))

complete_data <- na.omit(IBD_ASMR_SDI)

cor.test(complete_data$ASMR, complete_data$SDI)

IBD_region22 <- read.csv('NHL_region.csv',header = T)

order_SDI <- read.csv('order_SDI.csv',header = F)

SDI_2021<-read.csv("SDI_2021.csv",header = T)

SDI_2021<-SDI_2021[,-1]

SDI_2021 <- melt(SDI_2021,id.vars ='Location')

SDI_2021$variable <- as.numeric(gsub('\\X',replacement = '', SDI_2021$variable))

names(SDI_2021) <- c('location','year','SDI')

SDI_2021$location[which(SDI_2021$location =='Central sub-Saharan Africa')] <-'Central Sub-Saharan Africa'

SDI_2021$location[which(SDI_2021$location =='Eastern sub-Saharan Africa')] <-'Eastern Sub-Saharan Africa'

SDI_2021$location[which(SDI_2021$location =='Southern sub-Saharan Africa')] <-'Southern Sub-Saharan Africa'

SDI_2021$location[which(SDI_2021$location =='Western sub-Saharan Africa')] <-'Western Sub-Saharan Africa'

### ASDR#####

IBD_ASMR <- subset(IBD_region22, IBD_region22$age_name=='Age-standardized' &

IBD_region22$metric_name== 'Rate' &

IBD_region22$measure_name=='Deaths'&

IBD_region22$sex_name=="Both")

IBD_ASMR <- IBD_ASMR[,c(5,14,15)]

names(IBD_ASMR)[3] <- 'ASMR'

names(IBD_ASMR)[1] <- 'location'

IBD_ASMR_SDI <- merge(IBD_ASMR,SDI_2021,by=c('location','year'))

IBD_ASMR_SDI$location <- factor(IBD_ASMR_SDI$location,

levels=order_SDI$V1,

ordered=TRUE)

write.csv(IBD_ASMR_SDI,"22_ASDR_SDI.csv")

size_breaks <- seq(min(IBD_ASMR_SDI$year), max(IBD_ASMR_SDI$year), by = 5)

size_labels <- size_breaks

ggplot(IBD_ASMR_SDI, aes(SDI,ASMR)) + geom_point(aes(color = location, shape= location,size=year))+

scale_shape_manual(values = 1:22) +

labs(x = "Socio-Demographic Index",

y = "ASMR(per 100000)") +

geom_smooth(colour='black',stat = "smooth",method='loess',se=F,span=0.5)+

scale_size_continuous(breaks = size_breaks, labels = size_labels,range = c(1, 3))

complete_data <- na.omit(IBD_ASMR_SDI)

cor.test(complete_data$ASMR, complete_data$SDI)

### ASIR#####

IBD_ASMR <- subset(IBD_region22, IBD_region22$age_name=='Age-standardized' &

IBD_region22$metric_name== 'Rate' &

IBD_region22$measure_name=='Incidence'&

IBD_region22$sex_name=="Both")

IBD_ASMR <- IBD_ASMR[,c(5,14,15)]

names(IBD_ASMR)[3] <- 'ASMR'

names(IBD_ASMR)[1] <- 'location'

IBD_ASMR_SDI <- merge(IBD_ASMR,SDI_2021,by=c('location','year'))

IBD_ASMR_SDI$location <- factor(IBD_ASMR_SDI$location,

levels=order_SDI$V1,

ordered=TRUE)

write.csv(IBD_ASMR_SDI,"22_ASIR_SDI.csv")

size_breaks <- seq(min(IBD_ASMR_SDI$year), max(IBD_ASMR_SDI$year), by = 5)

size_labels <- size_breaks

ggplot(IBD_ASMR_SDI, aes(SDI,ASMR)) + geom_point(aes(color = location, shape= location,size=year))+

scale_shape_manual(values = 1:22) +

labs(x = "Socio-Demographic Index",

y = "ASMR(per 100000") +

geom_smooth(colour='black',stat = "smooth",method='loess',se=F,span=0.5)+

scale_size_continuous(breaks = size_breaks, labels = size_labels,range = c(1, 3))

complete_data <- na.omit(IBD_ASMR_SDI)

cor.test(complete_data$ASMR, complete_data$SDI)

### ASPR#####

IBD_ASMR <- subset(IBD_region22, IBD_region22$age_name=='Age-standardized' &

IBD_region22$metric_name== 'Rate' &

IBD_region22$measure_name=='Prevalence'&

IBD_region22$sex_name=="Both")

IBD_ASMR <- IBD_ASMR[,c(5,14,15)]

names(IBD_ASMR)[3] <- 'ASMR'

names(IBD_ASMR)[1] <- 'location'

IBD_ASMR_SDI <- merge(IBD_ASMR,SDI_2021,by=c('location','year'))

IBD_ASMR_SDI$location <- factor(IBD_ASMR_SDI$location,

levels=order_SDI$V1,

ordered=TRUE)

write.csv(IBD_ASMR_SDI,"22_ASPR_SDI.csv")

size_breaks <- seq(min(IBD_ASMR_SDI$year), max(IBD_ASMR_SDI$year), by = 5)

size_labels <- size_breaks

ggplot(IBD_ASMR_SDI, aes(SDI,ASMR)) + geom_point(aes(color = location, shape= location,size=year))+

scale_shape_manual(values = 1:22) +

labs(x = "Socio-Demographic Index",

y = "ASMR(per 100000") +

geom_smooth(colour='black',stat = "smooth",method='loess',se=F,span=0.5)+

scale_size_continuous(breaks = size_breaks, labels = size_labels,range = c(1, 3))

complete_data <- na.omit(IBD_ASMR_SDI)

cor.test(complete_data$ASMR, complete_data$SDI)

### ASDALYsR#####

IBD_ASMR <- subset(IBD_region22, IBD_region22$age_name=='Age-standardized' &

IBD_region22$metric_name== 'Rate' &

IBD_region22$measure_name=='DALYs (Disability-Adjusted Life Years)'&

IBD_region22$sex_name=="Both")

IBD_ASMR <- IBD_ASMR[,c(5,14,15)]

names(IBD_ASMR)[3] <- 'ASMR'

names(IBD_ASMR)[1] <- 'location'

IBD_ASMR_SDI <- merge(IBD_ASMR,SDI_2021,by=c('location','year'))

IBD_ASMR_SDI$location <- factor(IBD_ASMR_SDI$location,

levels=order_SDI$V1,

ordered=TRUE)

write.csv(IBD_ASMR_SDI,"22_ASDALYsR_SDI.csv")

size_breaks <- seq(min(IBD_ASMR_SDI$year), max(IBD_ASMR_SDI$year), by = 5)

size_labels <- size_breaks

ggplot(IBD_ASMR_SDI, aes(SDI,ASMR)) + geom_point(aes(color = location, shape= location,size=year))+

scale_shape_manual(values = 1:22) +

labs(x = "Socio-Demographic Index",

y = "ASMR(per 100000") +

geom_smooth(colour='black',stat = "smooth",method='loess',se=F,span=0.5)+

scale_size_continuous(breaks = size_breaks, labels = size_labels,range = c(1, 3))

complete_data <- na.omit(IBD_ASMR_SDI)

cor.test(complete_data$ASMR, complete_data$SDI)

IBD_region22 <- read.csv('AML_region.csv',header = T)

order_SDI <- read.csv('order_SDI.csv',header = F)

SDI_2021<-read.csv("SDI_2021.csv",header = T)

SDI_2021<-SDI_2021[,-1]

SDI_2021 <- melt(SDI_2021,id.vars ='Location')

SDI_2021$variable <- as.numeric(gsub('\\X',replacement = '', SDI_2021$variable))

names(SDI_2021) <- c('location','year','SDI')

SDI_2021$location[which(SDI_2021$location =='Central sub-Saharan Africa')] <-'Central Sub-Saharan Africa'

SDI_2021$location[which(SDI_2021$location =='Eastern sub-Saharan Africa')] <-'Eastern Sub-Saharan Africa'

SDI_2021$location[which(SDI_2021$location =='Southern sub-Saharan Africa')] <-'Southern Sub-Saharan Africa'

SDI_2021$location[which(SDI_2021$location =='Western sub-Saharan Africa')] <-'Western Sub-Saharan Africa'

### ASDR#####

IBD_ASMR <- subset(IBD_region22, IBD_region22$age_name=='Age-standardized' &

IBD_region22$metric_name== 'Rate' &

IBD_region22$measure_name=='Deaths'&

IBD_region22$sex_name=="Both")

IBD_ASMR <- IBD_ASMR[,c(5,14,15)]

names(IBD_ASMR)[3] <- 'ASMR'

names(IBD_ASMR)[1] <- 'location'

IBD_ASMR_SDI <- merge(IBD_ASMR,SDI_2021,by=c('location','year'))

IBD_ASMR_SDI$location <- factor(IBD_ASMR_SDI$location,

levels=order_SDI$V1,

ordered=TRUE)

write.csv(IBD_ASMR_SDI,"22_ASDR_SDI.csv")

size_breaks <- seq(min(IBD_ASMR_SDI$year), max(IBD_ASMR_SDI$year), by = 5)

size_labels <- size_breaks

ggplot(IBD_ASMR_SDI, aes(SDI,ASMR)) + geom_point(aes(color = location, shape= location,size=year))+

scale_shape_manual(values = 1:22) +

labs(x = "Socio-Demographic Index",

y = "ASMR(per 100000)") +

geom_smooth(colour='black',stat = "smooth",method='loess',se=F,span=0.5)+

scale_size_continuous(breaks = size_breaks, labels = size_labels,range = c(1, 3))

complete_data <- na.omit(IBD_ASMR_SDI)

cor.test(complete_data$ASMR, complete_data$SDI)

### ASIR#####

IBD_ASMR <- subset(IBD_region22, IBD_region22$age_name=='Age-standardized' &

IBD_region22$metric_name== 'Rate' &

IBD_region22$measure_name=='Incidence'&

IBD_region22$sex_name=="Both")

IBD_ASMR <- IBD_ASMR[,c(5,14,15)]

names(IBD_ASMR)[3] <- 'ASMR'

names(IBD_ASMR)[1] <- 'location'

IBD_ASMR_SDI <- merge(IBD_ASMR,SDI_2021,by=c('location','year'))

IBD_ASMR_SDI$location <- factor(IBD_ASMR_SDI$location,

levels=order_SDI$V1,

ordered=TRUE)

write.csv(IBD_ASMR_SDI,"22_ASIR_SDI.csv")

size_breaks <- seq(min(IBD_ASMR_SDI$year), max(IBD_ASMR_SDI$year), by = 5)

size_labels <- size_breaks

ggplot(IBD_ASMR_SDI, aes(SDI,ASMR)) + geom_point(aes(color = location, shape= location,size=year))+

scale_shape_manual(values = 1:22) +

labs(x = "Socio-Demographic Index",

y = "ASMR(per 100000") +

geom_smooth(colour='black',stat = "smooth",method='loess',se=F,span=0.5)+

scale_size_continuous(breaks = size_breaks, labels = size_labels,range = c(1, 3))

complete_data <- na.omit(IBD_ASMR_SDI)

cor.test(complete_data$ASMR, complete_data$SDI)

### ASPR#####

IBD_ASMR <- subset(IBD_region22, IBD_region22$age_name=='Age-standardized' &

IBD_region22$metric_name== 'Rate' &

IBD_region22$measure_name=='Prevalence'&

IBD_region22$sex_name=="Both")

IBD_ASMR <- IBD_ASMR[,c(5,14,15)]

names(IBD_ASMR)[3] <- 'ASMR'

names(IBD_ASMR)[1] <- 'location'

IBD_ASMR_SDI <- merge(IBD_ASMR,SDI_2021,by=c('location','year'))

IBD_ASMR_SDI$location <- factor(IBD_ASMR_SDI$location,

levels=order_SDI$V1,

ordered=TRUE)

write.csv(IBD_ASMR_SDI,"22_ASPR_SDI.csv")

size_breaks <- seq(min(IBD_ASMR_SDI$year), max(IBD_ASMR_SDI$year), by = 5)

size_labels <- size_breaks

ggplot(IBD_ASMR_SDI, aes(SDI,ASMR)) + geom_point(aes(color = location, shape= location,size=year))+

scale_shape_manual(values = 1:22) +

labs(x = "Socio-Demographic Index",

y = "ASMR(per 100000") +

geom_smooth(colour='black',stat = "smooth",method='loess',se=F,span=0.5)+

scale_size_continuous(breaks = size_breaks, labels = size_labels,range = c(1, 3))

complete_data <- na.omit(IBD_ASMR_SDI)

cor.test(complete_data$ASMR, complete_data$SDI)

### ASDALYsR#####

IBD_ASMR <- subset(IBD_region22, IBD_region22$age_name=='Age-standardized' &

IBD_region22$metric_name== 'Rate' &

IBD_region22$measure_name=='DALYs (Disability-Adjusted Life Years)'&

IBD_region22$sex_name=="Both")

IBD_ASMR <- IBD_ASMR[,c(5,14,15)]

names(IBD_ASMR)[3] <- 'ASMR'

names(IBD_ASMR)[1] <- 'location'

IBD_ASMR_SDI <- merge(IBD_ASMR,SDI_2021,by=c('location','year'))

IBD_ASMR_SDI$location <- factor(IBD_ASMR_SDI$location,

levels=order_SDI$V1,

ordered=TRUE)

write.csv(IBD_ASMR_SDI,"22_ASDALYsR_SDI.csv")

size_breaks <- seq(min(IBD_ASMR_SDI$year), max(IBD_ASMR_SDI$year), by = 5)

size_labels <- size_breaks

ggplot(IBD_ASMR_SDI, aes(SDI,ASMR)) + geom_point(aes(color = location, shape= location,size=year))+

scale_shape_manual(values = 1:22) +

labs(x = "Socio-Demographic Index",

y = "ASMR(per 100000") +

geom_smooth(colour='black',stat = "smooth",method='loess',se=F,span=0.5)+

scale_size_continuous(breaks = size_breaks, labels = size_labels,range = c(1, 3))

complete_data <- na.omit(IBD_ASMR_SDI)

cor.test(complete_data$ASMR, complete_data$SDI)

IBD_region22 <- read.csv('CML_region.csv',header = T)

order_SDI <- read.csv('order_SDI.csv',header = F)

SDI_2021<-read.csv("SDI_2021.csv",header = T)

SDI_2021<-SDI_2021[,-1]

SDI_2021 <- melt(SDI_2021,id.vars ='Location')

SDI_2021$variable <- as.numeric(gsub('\\X',replacement = '', SDI_2021$variable))

names(SDI_2021) <- c('location','year','SDI')

SDI_2021$location[which(SDI_2021$location =='Central sub-Saharan Africa')] <-'Central Sub-Saharan Africa'

SDI_2021$location[which(SDI_2021$location =='Eastern sub-Saharan Africa')] <-'Eastern Sub-Saharan Africa'

SDI_2021$location[which(SDI_2021$location =='Southern sub-Saharan Africa')] <-'Southern Sub-Saharan Africa'

SDI_2021$location[which(SDI_2021$location =='Western sub-Saharan Africa')] <-'Western Sub-Saharan Africa'

### ASDR#####

IBD_ASMR <- subset(IBD_region22, IBD_region22$age_name=='Age-standardized' &

IBD_region22$metric_name== 'Rate' &

IBD_region22$measure_name=='Deaths'&

IBD_region22$sex_name=="Both")

IBD_ASMR <- IBD_ASMR[,c(5,14,15)]

names(IBD_ASMR)[3] <- 'ASMR'

names(IBD_ASMR)[1] <- 'location'

IBD_ASMR_SDI <- merge(IBD_ASMR,SDI_2021,by=c('location','year'))

IBD_ASMR_SDI$location <- factor(IBD_ASMR_SDI$location,

levels=order_SDI$V1,

ordered=TRUE)

write.csv(IBD_ASMR_SDI,"22_ASDR_SDI.csv")

size_breaks <- seq(min(IBD_ASMR_SDI$year), max(IBD_ASMR_SDI$year), by = 5)

size_labels <- size_breaks

ggplot(IBD_ASMR_SDI, aes(SDI,ASMR)) + geom_point(aes(color = location, shape= location,size=year))+

scale_shape_manual(values = 1:22) +

labs(x = "Socio-Demographic Index",

y = "ASMR(per 100000)") +

geom_smooth(colour='black',stat = "smooth",method='loess',se=F,span=0.5)+

scale_size_continuous(breaks = size_breaks, labels = size_labels,range = c(1, 3))

complete_data <- na.omit(IBD_ASMR_SDI)

cor.test(complete_data$ASMR, complete_data$SDI)

### ASIR#####

IBD_ASMR <- subset(IBD_region22, IBD_region22$age_name=='Age-standardized' &

IBD_region22$metric_name== 'Rate' &

IBD_region22$measure_name=='Incidence'&

IBD_region22$sex_name=="Both")

IBD_ASMR <- IBD_ASMR[,c(5,14,15)]

names(IBD_ASMR)[3] <- 'ASMR'

names(IBD_ASMR)[1] <- 'location'

IBD_ASMR_SDI <- merge(IBD_ASMR,SDI_2021,by=c('location','year'))

IBD_ASMR_SDI$location <- factor(IBD_ASMR_SDI$location,

levels=order_SDI$V1,

ordered=TRUE)

write.csv(IBD_ASMR_SDI,"22_ASIR_SDI.csv")

size_breaks <- seq(min(IBD_ASMR_SDI$year), max(IBD_ASMR_SDI$year), by = 5)

size_labels <- size_breaks

ggplot(IBD_ASMR_SDI, aes(SDI,ASMR)) + geom_point(aes(color = location, shape= location,size=year))+

scale_shape_manual(values = 1:22) +

labs(x = "Socio-Demographic Index",

y = "ASMR(per 100000") +

geom_smooth(colour='black',stat = "smooth",method='loess',se=F,span=0.5)+

scale_size_continuous(breaks = size_breaks, labels = size_labels,range = c(1, 3))

complete_data <- na.omit(IBD_ASMR_SDI)

cor.test(complete_data$ASMR, complete_data$SDI)

### ASPR#####

IBD_ASMR <- subset(IBD_region22, IBD_region22$age_name=='Age-standardized' &

IBD_region22$metric_name== 'Rate' &

IBD_region22$measure_name=='Prevalence'&

IBD_region22$sex_name=="Both")

IBD_ASMR <- IBD_ASMR[,c(5,14,15)]

names(IBD_ASMR)[3] <- 'ASMR'

names(IBD_ASMR)[1] <- 'location'

IBD_ASMR_SDI <- merge(IBD_ASMR,SDI_2021,by=c('location','year'))

IBD_ASMR_SDI$location <- factor(IBD_ASMR_SDI$location,

levels=order_SDI$V1,

ordered=TRUE)

write.csv(IBD_ASMR_SDI,"22_ASPR_SDI.csv")

size_breaks <- seq(min(IBD_ASMR_SDI$year), max(IBD_ASMR_SDI$year), by = 5)

size_labels <- size_breaks

ggplot(IBD_ASMR_SDI, aes(SDI,ASMR)) + geom_point(aes(color = location, shape= location,size=year))+

scale_shape_manual(values = 1:22) +

labs(x = "Socio-Demographic Index",

y = "ASMR(per 100000") +

geom_smooth(colour='black',stat = "smooth",method='loess',se=F,span=0.5)+

scale_size_continuous(breaks = size_breaks, labels = size_labels,range = c(1, 3))

complete_data <- na.omit(IBD_ASMR_SDI)

cor.test(complete_data$ASMR, complete_data$SDI)

### ASDALYsR#####

IBD_ASMR <- subset(IBD_region22, IBD_region22$age_name=='Age-standardized' &

IBD_region22$metric_name== 'Rate' &

IBD_region22$measure_name=='DALYs (Disability-Adjusted Life Years)'&

IBD_region22$sex_name=="Both")

IBD_ASMR <- IBD_ASMR[,c(5,14,15)]

names(IBD_ASMR)[3] <- 'ASMR'

names(IBD_ASMR)[1] <- 'location'

IBD_ASMR_SDI <- merge(IBD_ASMR,SDI_2021,by=c('location','year'))

IBD_ASMR_SDI$location <- factor(IBD_ASMR_SDI$location,

levels=order_SDI$V1,

ordered=TRUE)

write.csv(IBD_ASMR_SDI,"22_ASDALYsR_SDI.csv")

size_breaks <- seq(min(IBD_ASMR_SDI$year), max(IBD_ASMR_SDI$year), by = 5)

size_labels <- size_breaks

ggplot(IBD_ASMR_SDI, aes(SDI,ASMR)) + geom_point(aes(color = location, shape= location,size=year))+

scale_shape_manual(values = 1:22) +

labs(x = "Socio-Demographic Index",

y = "ASMR(per 100000") +

geom_smooth(colour='black',stat = "smooth",method='loess',se=F,span=0.5)+

scale_size_continuous(breaks = size_breaks, labels = size_labels,range = c(1, 3))

complete_data <- na.omit(IBD_ASMR_SDI)

cor.test(complete_data$ASMR, complete_data$SDI)

IBD_region22 <- read.csv('ALL_region.csv',header = T)

order_SDI <- read.csv('order_SDI.csv',header = F)

SDI_2021<-read.csv("SDI_2021.csv",header = T)

SDI_2021<-SDI_2021[,-1]

SDI_2021 <- melt(SDI_2021,id.vars ='Location')

SDI_2021$variable <- as.numeric(gsub('\\X',replacement = '', SDI_2021$variable))

names(SDI_2021) <- c('location','year','SDI')

SDI_2021$location[which(SDI_2021$location =='Central sub-Saharan Africa')] <-'Central Sub-Saharan Africa'

SDI_2021$location[which(SDI_2021$location =='Eastern sub-Saharan Africa')] <-'Eastern Sub-Saharan Africa'

SDI_2021$location[which(SDI_2021$location =='Southern sub-Saharan Africa')] <-'Southern Sub-Saharan Africa'

SDI_2021$location[which(SDI_2021$location =='Western sub-Saharan Africa')] <-'Western Sub-Saharan Africa'

### ASDR#####

IBD_ASMR <- subset(IBD_region22, IBD_region22$age_name=='Age-standardized' &

IBD_region22$metric_name== 'Rate' &

IBD_region22$measure_name=='Deaths'&

IBD_region22$sex_name=="Both")

IBD_ASMR <- IBD_ASMR[,c(5,14,15)]

names(IBD_ASMR)[3] <- 'ASMR'

names(IBD_ASMR)[1] <- 'location'

IBD_ASMR_SDI <- merge(IBD_ASMR,SDI_2021,by=c('location','year'))

IBD_ASMR_SDI$location <- factor(IBD_ASMR_SDI$location,

levels=order_SDI$V1,

ordered=TRUE)

write.csv(IBD_ASMR_SDI,"22_ASDR_SDI.csv")

size_breaks <- seq(min(IBD_ASMR_SDI$year), max(IBD_ASMR_SDI$year), by = 5)

size_labels <- size_breaks

ggplot(IBD_ASMR_SDI, aes(SDI,ASMR)) + geom_point(aes(color = location, shape= location,size=year))+

scale_shape_manual(values = 1:22) +

labs(x = "Socio-Demographic Index",

y = "ASMR(per 100000)") +

geom_smooth(colour='black',stat = "smooth",method='loess',se=F,span=0.5)+

scale_size_continuous(breaks = size_breaks, labels = size_labels,range = c(1, 3))

complete_data <- na.omit(IBD_ASMR_SDI)

cor.test(complete_data$ASMR, complete_data$SDI)

### ASIR#####

IBD_ASMR <- subset(IBD_region22, IBD_region22$age_name=='Age-standardized' &

IBD_region22$metric_name== 'Rate' &

IBD_region22$measure_name=='Incidence'&

IBD_region22$sex_name=="Both")

IBD_ASMR <- IBD_ASMR[,c(5,14,15)]

names(IBD_ASMR)[3] <- 'ASMR'

names(IBD_ASMR)[1] <- 'location'

IBD_ASMR_SDI <- merge(IBD_ASMR,SDI_2021,by=c('location','year'))

IBD_ASMR_SDI$location <- factor(IBD_ASMR_SDI$location,

levels=order_SDI$V1,

ordered=TRUE)

write.csv(IBD_ASMR_SDI,"22_ASIR_SDI.csv")

size_breaks <- seq(min(IBD_ASMR_SDI$year), max(IBD_ASMR_SDI$year), by = 5)

size_labels <- size_breaks

ggplot(IBD_ASMR_SDI, aes(SDI,ASMR)) + geom_point(aes(color = location, shape= location,size=year))+

scale_shape_manual(values = 1:22) +

labs(x = "Socio-Demographic Index",

y = "ASMR(per 100000") +

geom_smooth(colour='black',stat = "smooth",method='loess',se=F,span=0.5)+

scale_size_continuous(breaks = size_breaks, labels = size_labels,range = c(1, 3))

complete_data <- na.omit(IBD_ASMR_SDI)

cor.test(complete_data$ASMR, complete_data$SDI)

### ASPR#####

IBD_ASMR <- subset(IBD_region22, IBD_region22$age_name=='Age-standardized' &

IBD_region22$metric_name== 'Rate' &

IBD_region22$measure_name=='Prevalence'&

IBD_region22$sex_name=="Both")

IBD_ASMR <- IBD_ASMR[,c(5,14,15)]

names(IBD_ASMR)[3] <- 'ASMR'

names(IBD_ASMR)[1] <- 'location'

IBD_ASMR_SDI <- merge(IBD_ASMR,SDI_2021,by=c('location','year'))

IBD_ASMR_SDI$location <- factor(IBD_ASMR_SDI$location,

levels=order_SDI$V1,

ordered=TRUE)

write.csv(IBD_ASMR_SDI,"22_ASPR_SDI.csv")

size_breaks <- seq(min(IBD_ASMR_SDI$year), max(IBD_ASMR_SDI$year), by = 5)

size_labels <- size_breaks

ggplot(IBD_ASMR_SDI, aes(SDI,ASMR)) + geom_point(aes(color = location, shape= location,size=year))+

scale_shape_manual(values = 1:22) +

labs(x = "Socio-Demographic Index",

y = "ASMR(per 100000") +

geom_smooth(colour='black',stat = "smooth",method='loess',se=F,span=0.5)+

scale_size_continuous(breaks = size_breaks, labels = size_labels,range = c(1, 3))

complete_data <- na.omit(IBD_ASMR_SDI)

cor.test(complete_data$ASMR, complete_data$SDI)

### ASDALYsR#####

IBD_ASMR <- subset(IBD_region22, IBD_region22$age_name=='Age-standardized' &

IBD_region22$metric_name== 'Rate' &

IBD_region22$measure_name=='DALYs (Disability-Adjusted Life Years)'&

IBD_region22$sex_name=="Both")

IBD_ASMR <- IBD_ASMR[,c(5,14,15)]

names(IBD_ASMR)[3] <- 'ASMR'

names(IBD_ASMR)[1] <- 'location'

IBD_ASMR_SDI <- merge(IBD_ASMR,SDI_2021,by=c('location','year'))

IBD_ASMR_SDI$location <- factor(IBD_ASMR_SDI$location,

levels=order_SDI$V1,

ordered=TRUE)

write.csv(IBD_ASMR_SDI,"22_ASDALYsR_SDI.csv")

size_breaks <- seq(min(IBD_ASMR_SDI$year), max(IBD_ASMR_SDI$year), by = 5)

size_labels <- size_breaks

ggplot(IBD_ASMR_SDI, aes(SDI,ASMR)) + geom_point(aes(color = location, shape= location,size=year))+

scale_shape_manual(values = 1:22) +

labs(x = "Socio-Demographic Index",

y = "ASMR(per 100000") +

geom_smooth(colour='black',stat = "smooth",method='loess',se=F,span=0.5)+

scale_size_continuous(breaks = size_breaks, labels = size_labels,range = c(1, 3))

complete_data <- na.omit(IBD_ASMR_SDI)

cor.test(complete_data$ASMR, complete_data$SDI)

IBD_region22 <- read.csv('CLL_region.csv',header = T)

order_SDI <- read.csv('order_SDI.csv',header = F)

SDI_2021<-read.csv("SDI_2021.csv",header = T)

SDI_2021<-SDI_2021[,-1]

SDI_2021 <- melt(SDI_2021,id.vars ='Location')

SDI_2021$variable <- as.numeric(gsub('\\X',replacement = '', SDI_2021$variable))

names(SDI_2021) <- c('location','year','SDI')

SDI_2021$location[which(SDI_2021$location =='Central sub-Saharan Africa')] <-'Central Sub-Saharan Africa'

SDI_2021$location[which(SDI_2021$location =='Eastern sub-Saharan Africa')] <-'Eastern Sub-Saharan Africa'

SDI_2021$location[which(SDI_2021$location =='Southern sub-Saharan Africa')] <-'Southern Sub-Saharan Africa'

SDI_2021$location[which(SDI_2021$location =='Western sub-Saharan Africa')] <-'Western Sub-Saharan Africa'

### ASDR#####

IBD_ASMR <- subset(IBD_region22, IBD_region22$age_name=='Age-standardized' &

IBD_region22$metric_name== 'Rate' &

IBD_region22$measure_name=='Deaths'&

IBD_region22$sex_name=="Both")

IBD_ASMR <- IBD_ASMR[,c(5,14,15)]

names(IBD_ASMR)[3] <- 'ASMR'

names(IBD_ASMR)[1] <- 'location'

IBD_ASMR_SDI <- merge(IBD_ASMR,SDI_2021,by=c('location','year'))

IBD_ASMR_SDI$location <- factor(IBD_ASMR_SDI$location,

levels=order_SDI$V1,

ordered=TRUE)

write.csv(IBD_ASMR_SDI,"22_ASDR_SDI.csv")

size_breaks <- seq(min(IBD_ASMR_SDI$year), max(IBD_ASMR_SDI$year), by = 5)

size_labels <- size_breaks

ggplot(IBD_ASMR_SDI, aes(SDI,ASMR)) + geom_point(aes(color = location, shape= location,size=year))+

scale_shape_manual(values = 1:22) +

labs(x = "Socio-Demographic Index",

y = "ASMR(per 100000)") +

geom_smooth(colour='black',stat = "smooth",method='loess',se=F,span=0.5)+

scale_size_continuous(breaks = size_breaks, labels = size_labels,range = c(1, 3))

complete_data <- na.omit(IBD_ASMR_SDI)

cor.test(complete_data$ASMR, complete_data$SDI)

### ASIR#####

IBD_ASMR <- subset(IBD_region22, IBD_region22$age_name=='Age-standardized' &

IBD_region22$metric_name== 'Rate' &

IBD_region22$measure_name=='Incidence'&

IBD_region22$sex_name=="Both")

IBD_ASMR <- IBD_ASMR[,c(5,14,15)]

names(IBD_ASMR)[3] <- 'ASMR'

names(IBD_ASMR)[1] <- 'location'

IBD_ASMR_SDI <- merge(IBD_ASMR,SDI_2021,by=c('location','year'))

IBD_ASMR_SDI$location <- factor(IBD_ASMR_SDI$location,

levels=order_SDI$V1,

ordered=TRUE)

write.csv(IBD_ASMR_SDI,"22_ASIR_SDI.csv")

size_breaks <- seq(min(IBD_ASMR_SDI$year), max(IBD_ASMR_SDI$year), by = 5)

size_labels <- size_breaks

ggplot(IBD_ASMR_SDI, aes(SDI,ASMR)) + geom_point(aes(color = location, shape= location,size=year))+

scale_shape_manual(values = 1:22) +

labs(x = "Socio-Demographic Index",

y = "ASMR(per 100000") +

geom_smooth(colour='black',stat = "smooth",method='loess',se=F,span=0.5)+

scale_size_continuous(breaks = size_breaks, labels = size_labels,range = c(1, 3))

complete_data <- na.omit(IBD_ASMR_SDI)

cor.test(complete_data$ASMR, complete_data$SDI)

### ASPR#####

IBD_ASMR <- subset(IBD_region22, IBD_region22$age_name=='Age-standardized' &

IBD_region22$metric_name== 'Rate' &

IBD_region22$measure_name=='Prevalence'&

IBD_region22$sex_name=="Both")

IBD_ASMR <- IBD_ASMR[,c(5,14,15)]

names(IBD_ASMR)[3] <- 'ASMR'

names(IBD_ASMR)[1] <- 'location'

IBD_ASMR_SDI <- merge(IBD_ASMR,SDI_2021,by=c('location','year'))

IBD_ASMR_SDI$location <- factor(IBD_ASMR_SDI$location,

levels=order_SDI$V1,

ordered=TRUE)

write.csv(IBD_ASMR_SDI,"22_ASPR_SDI.csv")

size_breaks <- seq(min(IBD_ASMR_SDI$year), max(IBD_ASMR_SDI$year), by = 5)

size_labels <- size_breaks

ggplot(IBD_ASMR_SDI, aes(SDI,ASMR)) + geom_point(aes(color = location, shape= location,size=year))+

scale_shape_manual(values = 1:22) +

labs(x = "Socio-Demographic Index",

y = "ASMR(per 100000") +

geom_smooth(colour='black',stat = "smooth",method='loess',se=F,span=0.5)+

scale_size_continuous(breaks = size_breaks, labels = size_labels,range = c(1, 3))

complete_data <- na.omit(IBD_ASMR_SDI)

cor.test(complete_data$ASMR, complete_data$SDI)

### ASDALYsR#####

IBD_ASMR <- subset(IBD_region22, IBD_region22$age_name=='Age-standardized' &

IBD_region22$metric_name== 'Rate' &

IBD_region22$measure_name=='DALYs (Disability-Adjusted Life Years)'&

IBD_region22$sex_name=="Both")

IBD_ASMR <- IBD_ASMR[,c(5,14,15)]

names(IBD_ASMR)[3] <- 'ASMR'

names(IBD_ASMR)[1] <- 'location'

IBD_ASMR_SDI <- merge(IBD_ASMR,SDI_2021,by=c('location','year'))

IBD_ASMR_SDI$location <- factor(IBD_ASMR_SDI$location,

levels=order_SDI$V1,

ordered=TRUE)

write.csv(IBD_ASMR_SDI,"22_ASDALYsR_SDI.csv")

size_breaks <- seq(min(IBD_ASMR_SDI$year), max(IBD_ASMR_SDI$year), by = 5)

size_labels <- size_breaks

ggplot(IBD_ASMR_SDI, aes(SDI,ASMR)) + geom_point(aes(color = location, shape= location,size=year))+

scale_shape_manual(values = 1:22) +

labs(x = "Socio-Demographic Index",

y = "ASMR(per 100000") +

geom_smooth(colour='black',stat = "smooth",method='loess',se=F,span=0.5)+

scale_size_continuous(breaks = size_breaks, labels = size_labels,range = c(1, 3))

complete_data <- na.omit(IBD_ASMR_SDI)

cor.test(complete_data$ASMR, complete_data$SDI)

IBD_region22 <- read.csv('MM_region.csv',header = T)

order_SDI <- read.csv('order_SDI.csv',header = F)

SDI_2021<-read.csv("SDI_2021.csv",header = T)

SDI_2021<-SDI_2021[,-1]

SDI_2021 <- melt(SDI_2021,id.vars ='Location')

SDI_2021$variable <- as.numeric(gsub('\\X',replacement = '', SDI_2021$variable))

names(SDI_2021) <- c('location','year','SDI')

SDI_2021$location[which(SDI_2021$location =='Central sub-Saharan Africa')] <-'Central Sub-Saharan Africa'

SDI_2021$location[which(SDI_2021$location =='Eastern sub-Saharan Africa')] <-'Eastern Sub-Saharan Africa'

SDI_2021$location[which(SDI_2021$location =='Southern sub-Saharan Africa')] <-'Southern Sub-Saharan Africa'

SDI_2021$location[which(SDI_2021$location =='Western sub-Saharan Africa')] <-'Western Sub-Saharan Africa'

### ASDR#####

IBD_ASMR <- subset(IBD_region22, IBD_region22$age_name=='Age-standardized' &

IBD_region22$metric_name== 'Rate' &

IBD_region22$measure_name=='Deaths'&

IBD_region22$sex_name=="Both")

IBD_ASMR <- IBD_ASMR[,c(5,14,15)]

names(IBD_ASMR)[3] <- 'ASMR'

names(IBD_ASMR)[1] <- 'location'

IBD_ASMR_SDI <- merge(IBD_ASMR,SDI_2021,by=c('location','year'))

IBD_ASMR_SDI$location <- factor(IBD_ASMR_SDI$location,

levels=order_SDI$V1,

ordered=TRUE)

write.csv(IBD_ASMR_SDI,"22_ASDR_SDI.csv")

size_breaks <- seq(min(IBD_ASMR_SDI$year), max(IBD_ASMR_SDI$year), by = 5)

size_labels <- size_breaks

ggplot(IBD_ASMR_SDI, aes(SDI,ASMR)) + geom_point(aes(color = location, shape= location,size=year))+

scale_shape_manual(values = 1:22) +

labs(x = "Socio-Demographic Index",

y = "ASMR(per 100000)") +

geom_smooth(colour='black',stat = "smooth",method='loess',se=F,span=0.5)+

scale_size_continuous(breaks = size_breaks, labels = size_labels,range = c(1, 3))

complete_data <- na.omit(IBD_ASMR_SDI)

cor.test(complete_data$ASMR, complete_data$SDI)

### ASIR#####

IBD_ASMR <- subset(IBD_region22, IBD_region22$age_name=='Age-standardized' &

IBD_region22$metric_name== 'Rate' &

IBD_region22$measure_name=='Incidence'&

IBD_region22$sex_name=="Both")

IBD_ASMR <- IBD_ASMR[,c(5,14,15)]

names(IBD_ASMR)[3] <- 'ASMR'

names(IBD_ASMR)[1] <- 'location'

IBD_ASMR_SDI <- merge(IBD_ASMR,SDI_2021,by=c('location','year'))

IBD_ASMR_SDI$location <- factor(IBD_ASMR_SDI$location,

levels=order_SDI$V1,

ordered=TRUE)

write.csv(IBD_ASMR_SDI,"22_ASIR_SDI.csv")

size_breaks <- seq(min(IBD_ASMR_SDI$year), max(IBD_ASMR_SDI$year), by = 5)

size_labels <- size_breaks

ggplot(IBD_ASMR_SDI, aes(SDI,ASMR)) + geom_point(aes(color = location, shape= location,size=year))+

scale_shape_manual(values = 1:22) +

labs(x = "Socio-Demographic Index",

y = "ASMR(per 100000") +

geom_smooth(colour='black',stat = "smooth",method='loess',se=F,span=0.5)+

scale_size_continuous(breaks = size_breaks, labels = size_labels,range = c(1, 3))

complete_data <- na.omit(IBD_ASMR_SDI)

cor.test(complete_data$ASMR, complete_data$SDI)

### ASPR#####

IBD_ASMR <- subset(IBD_region22, IBD_region22$age_name=='Age-standardized' &

IBD_region22$metric_name== 'Rate' &

IBD_region22$measure_name=='Prevalence'&

IBD_region22$sex_name=="Both")

IBD_ASMR <- IBD_ASMR[,c(5,14,15)]

names(IBD_ASMR)[3] <- 'ASMR'

names(IBD_ASMR)[1] <- 'location'

IBD_ASMR_SDI <- merge(IBD_ASMR,SDI_2021,by=c('location','year'))

IBD_ASMR_SDI$location <- factor(IBD_ASMR_SDI$location,

levels=order_SDI$V1,

ordered=TRUE)

write.csv(IBD_ASMR_SDI,"22_ASPR_SDI.csv")

size_breaks <- seq(min(IBD_ASMR_SDI$year), max(IBD_ASMR_SDI$year), by = 5)

size_labels <- size_breaks

ggplot(IBD_ASMR_SDI, aes(SDI,ASMR)) + geom_point(aes(color = location, shape= location,size=year))+

scale_shape_manual(values = 1:22) +

labs(x = "Socio-Demographic Index",

y = "ASMR(per 100000") +

geom_smooth(colour='black',stat = "smooth",method='loess',se=F,span=0.5)+

scale_size_continuous(breaks = size_breaks, labels = size_labels,range = c(1, 3))

complete_data <- na.omit(IBD_ASMR_SDI)

cor.test(complete_data$ASMR, complete_data$SDI)

### ASDALYsR#####

IBD_ASMR <- subset(IBD_region22, IBD_region22$age_name=='Age-standardized' &

IBD_region22$metric_name== 'Rate' &

IBD_region22$measure_name=='DALYs (Disability-Adjusted Life Years)'&

IBD_region22$sex_name=="Both")

IBD_ASMR <- IBD_ASMR[,c(5,14,15)]

names(IBD_ASMR)[3] <- 'ASMR'

names(IBD_ASMR)[1] <- 'location'

IBD_ASMR_SDI <- merge(IBD_ASMR,SDI_2021,by=c('location','year'))

IBD_ASMR_SDI$location <- factor(IBD_ASMR_SDI$location,

levels=order_SDI$V1,

ordered=TRUE)

write.csv(IBD_ASMR_SDI,"22_ASDALYsR_SDI.csv")

size_breaks <- seq(min(IBD_ASMR_SDI$year), max(IBD_ASMR_SDI$year), by = 5)

size_labels <- size_breaks

ggplot(IBD_ASMR_SDI, aes(SDI,ASMR)) + geom_point(aes(color = location, shape= location,size=year))+

scale_shape_manual(values = 1:22) +

labs(x = "Socio-Demographic Index",

y = "ASMR(per 100000") +

geom_smooth(colour='black',stat = "smooth",method='loess',se=F,span=0.5)+

scale_size_continuous(breaks = size_breaks, labels = size_labels,range = c(1, 3))

complete_data <- na.omit(IBD_ASMR_SDI)

cor.test(complete_data$ASMR, complete_data$SDI)

#Figure 7

library(ggplot2)

order <- c('Global','High SDI','High-middle SDI','Middle SDI','Low-middle SDI',

'Low SDI','Andean Latin America','Australasia','Caribbean','Central Asia',

'Central Europe','Central Latin America','Central Sub-Saharan Africa',

'East Asia','Eastern Europe','Eastern Sub-Saharan Africa','High-income Asia Pacific',

'High-income North America','North Africa and Middle East','Oceania',

'South Asia','Southeast Asia','Southern Latin America','Southern Sub-Saharan Africa',

'Tropical Latin America','Western Europe','Western Sub-Saharan Africa')

order <- rev(order)

order <- factor(1:length(order),labels = order)

locations <- c('High body-mass index','Tobacco')

locations <- factor(1:length(locations),labels = locations)

RF_ad <- read.csv('IHME-GBD_2021_DATA-9cfa8e21-1.csv',header = T)

RF_ad$location <- factor(RF_ad$location_name,levels = levels(order))

RF_ad$rei <- factor(RF_ad$rei_name,levels = levels(locations))

p <- ggplot(RF_ad,aes(x=location,y=val*100,group=measure_name)) +

geom_bar(aes(fill=measure_name),stat="identity",position='dodge')+

scale_y_continuous(limits=c(0,15))+

scale_fill_manual(values=c("#b54537",'#2070ad'),

labels=c(

'DALYs (Disability-Adjusted Life Years)'='DALYs'

))+

theme_bw() +

facet_wrap(~rei,scales="free_x",ncol=4)+

coord_flip()+

labs(x='',

y='')

p

#Figure 8; Figure S15

library(BAPC)

library(INLA)

library(data.table)

library(tidyverse)

library(dplyr)

library(ggplot2)

library(epitools)

library(reshape2)

library(openxlsx)

age_stand <- read.xlsx('Age standard.xlsx')

agestand <- c(age_stand$Percent_Population %>% as.numeric())/sum(age_stand$Percent_Population)

ages <- c('<5 years','5-9 years','10-14 years','15-19 years','20-24 years',

'25-29 years','30-34 years','35-39 years','40-44 years','45-49 years',

'50-54 years','55-59 years','60-64 years','65-69 years','70-74 years',

'75-79 years','80-84 years','85-89 years','90-94 years','95+ years')

IS_Male_Incidence <- IS %>%

filter(age %in% ages &

measure == 'Incidence' &

metric == 'Number' &

location == "Global" &

sex == "Male") %>%

mutate(age = factor(age,

levels = ages))

IS_Male_Incidence_n <- reshape2::dcast(data = IS_Male_Incidence, year~age, value.var = "val")

rownames(IS_Male_Incidence_n) <- IS_Male_Incidence_n$year

IS_Male_Incidence_n <- IS_Male_Incidence_n[,-1] %>%

apply(c(1,2), as.numeric) %>%

apply(c(1,2), round) %>%

as.data.frame()

IS_Female_Incidence <- IS %>%

filter(age %in% ages &

measure == 'Incidence' &

metric == 'Number' &

location == "Global" &

sex == "Female") %>%

mutate(age = factor(age,

levels = ages))

IS_Female_Incidence_n <- reshape2::dcast(data = IS_Female_Incidence, year~age, value.var = "val")

rownames(IS_Female_Incidence_n) <- IS_Female_Incidence_n$year

IS_Female_Incidence_n <- IS_Female_Incidence_n[,-1] %>%

apply(c(1,2), as.numeric) %>%

apply(c(1,2), round) %>%

as.data.frame()

IS_Both_Incidence_n <- IS_Male_Incidence_n +IS_Female_Incidence_n

GBD_population <- fread('IHME-GBD_2021_DATA-358594dc-1.csv') %>% as.data.frame() %>%

filter(age_name %in% ages) %>%

dplyr::select(6,8,11,12) %>%

rename(sex=sex_name,

age=age_name,

pop=val) %>%

mutate(age=factor(age,

levels=ages))

GBD_population_prediction <- fread('IHME_POP_2017_2100_POP_REFERENCE_Y2020M05D01.csv') %>% as.data.frame() %>%

mutate(age_group_name=sub(' to ',replacement = '-', age_group_name)) %>%

mutate(age_group_name=sub('95 plus',replacement = '95+', age_group_name)) %>%

mutate(age_group_name=str_c(age_group_name,' years')) %>%

filter(location_name == "Global") %>%

filter(age_group_name %in% ages) %>%

filter(year_id %in% 2022:2040) %>%

dplyr::select(4,6,7,14) %>%

rename(year=year_id,

age=age_group_name,

pop=val)

GBD_population_prediction_under5 <- fread('IHME_POP_2017_2100_POP_REFERENCE_Y2020M05D01.csv') %>% as.data.frame() %>%

filter(age_group_name %in% c(

"Early Neonatal", "Late Neonatal",

"Post Neonatal", "1 to 4"

)) %>%

filter(location_name == "Global") %>%

filter(year_id %in% 2022:2040) %>%

group_by(sex,year_id) %>%

reframe(sex,

age="<5 years",

pop=sum(val)) %>%

rename(year=year_id) %>%

distinct()

GBD_population_prediction <- GBD_population_prediction %>%

rbind(GBD_population_prediction_under5) %>%

mutate(age=factor(age,

levels=ages))

unique(GBD_population_prediction$age)

GBD <- rbind(GBD_population,GBD_population_prediction)

GBD_Global_Male <- GBD %>% filter(sex == 'Male')

GBD_Global_Female <- GBD %>% filter(sex == 'Female')

GBD_Global_Male_n <- reshape2::dcast(data = GBD_Global_Male,

year ~ age ,

value.var = c("pop")) %>%

select(-1)

GBD_Global_Female_n <- reshape2::dcast(data = GBD_Global_Female,

year ~ age,

value.var = c("pop")) %>%

select(-1)

GBD_Global_Both_n <- GBD_Global_Female_n + GBD_Global_Male_n

IS_pro <- matrix(data = NA, nrow = 2040-2021, ncol = ncol(GBD_Global_Male_n)) %>% as.data.frame()

rownames(IS_pro) <- seq(2022,2040,1)

colnames(IS_pro) <- names(IS_Male_Incidence_n)

IS_Male_Incidence_n <- rbind(IS_Male_Incidence_n , IS_pro)

IS_Female_Incidence_n <- rbind(IS_Female_Incidence_n , IS_pro)

IS_Both_Incidence_n <- rbind(IS_Both_Incidence_n , IS_pro)

Male_esoph <- APCList(IS_Male_Incidence_n, GBD_Global_Male_n, gf = 5)

Male_bapc_result <- BAPC(Male_esoph, predict = list(npredict = 19, retro = T),

secondDiff = FALSE, stdweight = agestand, verbose = F)

Female_esoph <- APCList(IS_Female_Incidence_n, GBD_Global_Female_n, gf = 5)

Female_bapc_result <- BAPC(Female_esoph, predict = list(npredict = 19, retro = T),

secondDiff = FALSE, stdweight = agestand, verbose = F)

Both_esoph <- APCList(IS_Both_Incidence_n, GBD_Global_Both_n, gf = 5)

Both_bapc_result <- BAPC(Both_esoph, predict = list(npredict = 19, retro = T),

secondDiff = FALSE, stdweight = agestand, verbose = F)

Male_ASR <- agestd.rate(x = Male_bapc_result) %>% as.data.frame()

Male_ASR$mean <- Male_ASR$mean*100000

Male_ASR$year <- rownames(Male_ASR)

Female_ASR <- agestd.rate(x =Female_bapc_result) %>% as.data.frame()

Female_ASR$mean <- Female_ASR$mean*100000

Female_ASR$year <- rownames(Female_ASR)

Both_ASR <- agestd.rate(x =Both_bapc_result) %>% as.data.frame()

Both_ASR$mean <- Both_ASR$mean*100000

Both_ASR$year <- rownames(Both_ASR)

Male_bapc_result <- qapc(Male_bapc_result,percentiles=c(0.025,0.975))

Female_bapc_result <- qapc(Female_bapc_result,percentiles=c(0.025,0.975))

Both_bapc_result <- qapc(Both_bapc_result,percentiles=c(0.025,0.975))

Male_ASR <- agestd.rate(x = Male_bapc_result) %>% as.data.frame()*10^5

Female_ASR <- agestd.rate(x = Female_bapc_result) %>% as.data.frame()*10^5

Both_ASR <- agestd.rate(x = Both_bapc_result) %>% as.data.frame()*10^5

Male_ASR$year <- 1990:2040

Female_ASR$year <- 1990:2040

Both_ASR$year <- 1990:2040

Male_ASR$sex <- 'Male'

Female_ASR$sex <- 'Female'

Both_ASR$sex <- 'Both'

ASR <- Male_ASR %>%

rbind(Female_ASR) %>%

rbind(Both_ASR) %>%

rename(ASR=mean,

lower='0.025Q',

upper='0.975Q') %>%

dplyr::select(6,5,1,3,4)

write.csv(ASR,"ASIR.csv")

p <- ggplot() +

geom_line(data=subset(ASR,year %in% 1990:2021),aes(year,ASR,color='Global',linetype='Observed')) +

geom_line(data=subset(ASR,year %in% 2021:2040),aes(year,ASR,linetype="Predicted"),color="#ce9494") +

geom_ribbon(data=subset(ASR,year %in% 2021:2040),aes(x=year,ymin=lower,ymax=upper),fill='#ce9494',alpha=.1) +

geom_vline(xintercept = 2021,linetype=2)+

scale_y_continuous(limits = c(0,NA))+

scale_linetype_manual(name="line",

values=c('Observed'=1,

"Predicted"=2

)) +

scale_color_manual(name="group",

values=c('Global'='#ce9494'

)) +

facet_wrap(~sex) +

labs(title = "Incidence",

y = 'Age-standardzied rate (per 100000 population)')

p

IS_Male_DALYs <- IS %>%

filter(age %in% ages &

measure == 'DALYs (Disability-Adjusted Life Years)' &

metric == 'Number' &

location == "Global" &

sex == "Male") %>%

mutate(age = factor(age,

levels = ages))

IS_Male_DALYs_n <- reshape2::dcast(data = IS_Male_DALYs, year~age, value.var = "val")

rownames(IS_Male_DALYs_n) <- IS_Male_DALYs_n$year

IS_Male_DALYs_n <- IS_Male_DALYs_n[,-1] %>%

apply(c(1,2), as.numeric) %>%

apply(c(1,2), round) %>%

as.data.frame()

IS_Female_DALYs <- IS %>%

filter(age %in% ages &

measure == 'DALYs (Disability-Adjusted Life Years)' &

metric == 'Number' &

location == "Global" &

sex == "Female") %>%

mutate(age = factor(age,

levels = ages))

IS_Female_DALYs_n <- reshape2::dcast(data = IS_Female_DALYs, year~age, value.var = "val")

rownames(IS_Female_DALYs_n) <- IS_Female_DALYs_n$year

IS_Female_DALYs_n <- IS_Female_DALYs_n[,-1] %>%

apply(c(1,2), as.numeric) %>%

apply(c(1,2), round) %>%

as.data.frame()

IS_Both_DALYs_n <- IS_Male_DALYs_n +IS_Female_DALYs_n

GBD_population <- fread('IHME-GBD_2021_DATA-358594dc-1.csv') %>% as.data.frame() %>%

filter(age_name %in% ages) %>%

dplyr::select(6,8,11,12) %>%

rename(sex=sex_name,

age=age_name,

pop=val) %>%

mutate(age=factor(age,

levels=ages))

head(GBD_population)

GBD_population_prediction <- fread('IHME_POP_2017_2100_POP_REFERENCE_Y2020M05D01.csv') %>% as.data.frame() %>%

mutate(age_group_name=sub(' to ',replacement = '-', age_group_name)) %>%

mutate(age_group_name=sub('95 plus',replacement = '95+', age_group_name)) %>%

mutate(age_group_name=str_c(age_group_name,' years')) %>%

filter(location_name == "Global") %>%

filter(age_group_name %in% ages) %>%

filter(year_id %in% 2022:2040) %>%

dplyr::select(4,6,7,14) %>%

rename(year=year_id,

age=age_group_name,

pop=val)

GBD_population_prediction_under5 <- fread('IHME_POP_2017_2100_POP_REFERENCE_Y2020M05D01.csv') %>% as.data.frame() %>%

filter(age_group_name %in% c(

"Early Neonatal", "Late Neonatal",

"Post Neonatal", "1 to 4"

)) %>%

filter(location_name == "Global") %>%

filter(year_id %in% 2022:2040) %>%

group_by(sex,year_id) %>%

reframe(sex,

age="<5 years",

pop=sum(val)) %>%

rename(year=year_id) %>%

distinct()

GBD_population_prediction <- GBD_population_prediction %>%

rbind(GBD_population_prediction_under5) %>%

mutate(age=factor(age,

levels=ages))

unique(GBD_population_prediction$age)

GBD <- rbind(GBD_population,GBD_population_prediction)

GBD_Global_Male <- GBD %>% filter(sex == 'Male')

GBD_Global_Female <- GBD %>% filter(sex == 'Female')

GBD_Global_Male_n <- reshape2::dcast(data = GBD_Global_Male,

year ~ age ,

value.var = c("pop")) %>%

select(-1)

GBD_Global_Female_n <- reshape2::dcast(data = GBD_Global_Female,

year ~ age,

value.var = c("pop")) %>%

select(-1)

GBD_Global_Both_n <- GBD_Global_Female_n + GBD_Global_Male_n

IS_pro <- matrix(data = NA, nrow = 2040-2021, ncol = ncol(GBD_Global_Male_n)) %>% as.data.frame()

rownames(IS_pro) <- seq(2022,2040,1)

colnames(IS_pro) <- names(IS_Male_DALYs_n)

IS_Male_DALYs_n <- rbind(IS_Male_DALYs_n , IS_pro)

IS_Female_DALYs_n <- rbind(IS_Female_DALYs_n , IS_pro)

IS_Both_DALYs_n <- rbind(IS_Both_DALYs_n , IS_pro)

Male_esoph <- APCList(IS_Male_DALYs_n, GBD_Global_Male_n, gf = 5)

Male_bapc_result <- BAPC(Male_esoph, predict = list(npredict = 19, retro = T),

secondDiff = FALSE, stdweight = agestand, verbose = F)

Female_esoph <- APCList(IS_Female_DALYs_n, GBD_Global_Female_n, gf = 5)

Female_bapc_result <- BAPC(Female_esoph, predict = list(npredict = 19, retro = T),

secondDiff = FALSE, stdweight = agestand, verbose = F)

Both_esoph <- APCList(IS_Both_DALYs_n, GBD_Global_Both_n, gf = 5)

Both_bapc_result <- BAPC(Both_esoph, predict = list(npredict = 19, retro = T),

secondDiff = FALSE, stdweight = agestand, verbose = F)

Male_ASR <- agestd.rate(x = Male_bapc_result) %>% as.data.frame()

Male_ASR$mean <- Male_ASR$mean*100000

Male_ASR$year <- rownames(Male_ASR)

Female_ASR <- agestd.rate(x =Female_bapc_result) %>% as.data.frame()

Female_ASR$mean <- Female_ASR$mean*100000

Female_ASR$year <- rownames(Female_ASR)

Both_ASR <- agestd.rate(x =Both_bapc_result) %>% as.data.frame()

Both_ASR$mean <- Both_ASR$mean*100000

Both_ASR$year <- rownames(Both_ASR)

Male_bapc_result <- qapc(Male_bapc_result,percentiles=c(0.025,0.975))

Female_bapc_result <- qapc(Female_bapc_result,percentiles=c(0.025,0.975))

Both_bapc_result <- qapc(Both_bapc_result,percentiles=c(0.025,0.975))

Male_ASR <- agestd.rate(x = Male_bapc_result) %>% as.data.frame()*10^5

Female_ASR <- agestd.rate(x = Female_bapc_result) %>% as.data.frame()*10^5

Both_ASR <- agestd.rate(x = Both_bapc_result) %>% as.data.frame()*10^5

Male_ASR$year <- 1990:2040

Female_ASR$year <- 1990:2040

Both_ASR$year <- 1990:2040

Male_ASR$sex <- 'Male'

Female_ASR$sex <- 'Female'

Both_ASR$sex <- 'Both'

ASR <- Male_ASR %>%

rbind(Female_ASR) %>%

rbind(Both_ASR) %>%

rename(ASR=mean,

lower='0.025Q',

upper='0.975Q') %>%

dplyr::select(6,5,1,3,4)

write.csv(ASR,"ASDALYR_.csv")

p <- ggplot() +

geom_line(data=subset(ASR,year %in% 1990:2021),aes(year,ASR,color='Global',linetype='Observed')) +

geom_line(data=subset(ASR,year %in% 2021:2040),aes(year,ASR,linetype="Predicted"),color="#b68664") +

geom_ribbon(data=subset(ASR,year %in% 2021:2040),aes(x=year,ymin=lower,ymax=upper),fill='#b68664',alpha=.1) +

geom_vline(xintercept = 2021,linetype=2)+

scale_y_continuous(limits = c(0,NA))+

scale_linetype_manual(name="line",

values=c('Observed'=1,

"Predicted"=2

)) +

scale_color_manual(name="group",

values=c('Global'='#b68664'

)) +

facet_wrap(~sex) +

labs(title = "DALYs",

y = 'Age-standardzied rate (per 100000 population)')

p

###Table 1

library(dplyr)

library(ggplot2)

setwd("D:/HL-data")

IS <- HL_region %>%

dplyr::select(measure_name,location_name,metric_name,

sex_name,age_name,year,val,lower,upper) %>%

rename(measure=measure_name,

location=location_name,

metric=metric_name,

sex=sex_name,

age=age_name)

###Incidence

###Number

###1990 Incidence

IS_1990 <- IS %>%

filter(year==1990 &

age =='All ages' &

metric == 'Number' &

measure =='Incidence' &

sex =="Both"

) %>%

#Global+5 SDI regions+21 regions

filter(location == 'Global' |

location == 'Low SDI' |

location == 'Low-middle SDI' |

location == 'Middle SDI' |

location == 'High-middle SDI' |

location == 'High SDI' |

location == 'Andean Latin America' |

location == 'Australasia' |

location == 'Caribbean' |

location == 'Central Asia' |

location == 'Central Europe' |

location == 'Central Latin America' |

location == 'Central Sub-Saharan Africa' |

location == 'East Asia' |

location == 'Eastern Europe' |

location == 'Eastern Sub-Saharan Africa' |

location == 'High-income Asia Pacific' |

location == 'High-income North America' |

location == 'North Africa and Middle East' |

location == 'Oceania' |

location == 'South Asia' |

location == 'Southeast Asia' |

location == 'Southern Latin America' |

location == 'Southern Sub-Saharan Africa' |

location == 'Tropical Latin America' |

location == 'Western Europe' |

location == 'Western Sub-Saharan Africa') %>%

dplyr::select(,c(2,7:9))

IS_1990$val <- round(IS_1990$val,0)

IS_1990$lower <- round(IS_1990$lower,0)

IS_1990$upper <- round(IS_1990$upper,0)

IS_1990$'Number 1990' <- paste(IS_1990$lower,IS_1990$upper,sep = '-')

IS_1990$'Number 1990' <- paste(IS_1990$'Number 1990',')',sep = '')

IS_1990$'Number 1990' <- paste('(',IS_1990$'Number 1990',sep = '')

IS_1990$'Number 1990' <- paste(IS_1990$val,IS_1990$'Number 1990',sep = ' ')

Incidence_Num_1990 <- IS_1990[,c(1,5)]

head(Incidence_Num_1990)

## 2021 Incidence

IS_2021 <- IS %>%

filter(year ==2021 &

age =='All ages' &

metric == 'Number' &

measure =='Incidence' &

sex =="Both"

) %>%

#Global+5 SDI regions+21 regions

filter(location == 'Global' |

location == 'Low SDI' |

location == 'Low-middle SDI' |

location == 'Middle SDI' |

location == 'High-middle SDI' |

location == 'High SDI' |

location == 'Andean Latin America' |

location == 'Australasia' |

location == 'Caribbean' |

location == 'Central Asia' |

location == 'Central Europe' |

location == 'Central Latin America' |

location == 'Central Sub-Saharan Africa' |

location == 'East Asia' |

location == 'Eastern Europe' |

location == 'Eastern Sub-Saharan Africa' |

location == 'High-income Asia Pacific' |

location == 'High-income North America' |

location == 'North Africa and Middle East' |

location == 'Oceania' |

location == 'South Asia' |

location == 'Southeast Asia' |

location == 'Southern Latin America' |

location == 'Southern Sub-Saharan Africa' |

location == 'Tropical Latin America' |

location == 'Western Europe' |

location == 'Western Sub-Saharan Africa') %>%

dplyr::select(,c(2,7:9))

IS_2021$val <- round(IS_2021$val,0)

IS_2021$lower <- round(IS_2021$lower,0)

IS_2021$upper <- round(IS_2021$upper,0)

IS_2021$'Number 2021' <- paste(IS_2021$lower,IS_2021$upper,sep = '-')

IS_2021$'Number 2021' <- paste(IS_2021$'Number 2021',')',sep = '')

IS_2021$'Number 2021' <- paste('(',IS_2021$'Number 2021',sep = '')

IS_2021$'Number 2021' <- paste(IS_2021$val,IS_2021$'Number 2021',sep = ' ')

Incidence_Num_2021 <- IS_2021[,c(1,5)]

head(Incidence_Num_2021)

##### Age-standardized rates

## 1990 Incidence

IS_1990 <- IS %>%

filter(year==1990 &

age =='Age-standardized' &

metric == 'Rate' &

measure =='Incidence' &

sex =="Both"

) %>%

#Global+5 SDI regions+21 regions

filter(location == 'Global' |

location == 'Low SDI' |

location == 'Low-middle SDI' |

location == 'Middle SDI' |

location == 'High-middle SDI' |

location == 'High SDI' |

location == 'Andean Latin America' |

location == 'Australasia' |

location == 'Caribbean' |

location == 'Central Asia' |

location == 'Central Europe' |

location == 'Central Latin America' |

location == 'Central Sub-Saharan Africa' |

location == 'East Asia' |

location == 'Eastern Europe' |

location == 'Eastern Sub-Saharan Africa' |

location == 'High-income Asia Pacific' |

location == 'High-income North America' |

location == 'North Africa and Middle East' |

location == 'Oceania' |

location == 'South Asia' |

location == 'Southeast Asia' |

location == 'Southern Latin America' |

location == 'Southern Sub-Saharan Africa' |

location == 'Tropical Latin America' |

location == 'Western Europe' |

location == 'Western Sub-Saharan Africa') %>%

dplyr::select(,c(2,7:9))

IS_1990$val <- round(IS_1990$val,2)

IS_1990$lower <- round(IS_1990$lower,2)

IS_1990$upper <- round(IS_1990$upper,2)

IS_1990$'ASR 1990' <- paste(IS_1990$lower,IS_1990$upper,sep = '-')

IS_1990$'ASR 1990' <- paste(IS_1990$'ASR 1990',')',sep = '')

IS_1990$'ASR 1990' <- paste('(',IS_1990$'ASR 1990',sep = '')

IS_1990$'ASR 1990' <- paste(IS_1990$val,IS_1990$'ASR 1990',sep = ' ')

Incidence_ASR_1990 <- IS_1990[,c(1,5)]

head(Incidence_ASR_1990)

## 2021 Incidence

IS_2021 <- IS %>%

filter(year ==2021 &

age =='Age-standardized' &

metric == 'Rate' &

measure =='Incidence' &

sex =="Both"

) %>%

#Global+5 SDI regions+21 regions

filter(location == 'Global' |

location == 'Low SDI' |

location == 'Low-middle SDI' |

location == 'Middle SDI' |

location == 'High-middle SDI' |

location == 'High SDI' |

location == 'Andean Latin America' |

location == 'Australasia' |

location == 'Caribbean' |

location == 'Central Asia' |

location == 'Central Europe' |

location == 'Central Latin America' |

location == 'Central Sub-Saharan Africa' |

location == 'East Asia' |

location == 'Eastern Europe' |

location == 'Eastern Sub-Saharan Africa' |

location == 'High-income Asia Pacific' |

location == 'High-income North America' |

location == 'North Africa and Middle East' |

location == 'Oceania' |

location == 'South Asia' |

location == 'Southeast Asia' |

location == 'Southern Latin America' |

location == 'Southern Sub-Saharan Africa' |

location == 'Tropical Latin America' |

location == 'Western Europe' |

location == 'Western Sub-Saharan Africa') %>%

dplyr::select(,c(2,7:9))

IS_2021$val <- round(IS_2021$val,2)

IS_2021$lower <- round(IS_2021$lower,2)

IS_2021$upper <- round(IS_2021$upper,2)

IS_2021$'ASR 2021' <- paste(IS_2021$lower,IS_2021$upper,sep = '-')

IS_2021$'ASR 2021' <- paste(IS_2021$'ASR 2021',')',sep = '')

IS_2021$'ASR 2021' <- paste('(',IS_2021$'ASR 2021',sep = '')

IS_2021$'ASR 2021' <- paste(IS_2021$val,IS_2021$'ASR 2021',sep = ' ')

Incidence_ASR_2021 <- IS_2021[,c(1,5)]

head(Incidence_ASR_2021)

##### EAPC

Incidence_EAPC <- IS %>%

filter(age =='Age-standardized' &

metric == 'Rate' &

measure =='Incidence'&

sex =="Both"

) %>%

#Global+5 SDI regions+21 regions

filter(location == 'Global' |

location == 'Low SDI' |

location == 'Low-middle SDI' |

location == 'Middle SDI' |

location == 'High-middle SDI' |

location == 'High SDI' |

location == 'Andean Latin America' |

location == 'Australasia' |

location == 'Caribbean' |

location == 'Central Asia' |

location == 'Central Europe' |

location == 'Central Latin America' |

location == 'Central Sub-Saharan Africa' |

location == 'East Asia' |

location == 'Eastern Europe' |

location == 'Eastern Sub-Saharan Africa' |

location == 'High-income Asia Pacific' |

location == 'High-income North America' |

location == 'North Africa and Middle East' |

location == 'Oceania' |

location == 'South Asia' |

location == 'Southeast Asia' |

location == 'Southern Latin America' |

location == 'Southern Sub-Saharan Africa' |

location == 'Tropical Latin America' |

location == 'Western Europe' |

location == 'Western Sub-Saharan Africa') %>%

dplyr::select(,c(2,6,7))

nations <- IS_1990$location

EAPC_Incidence <- data.frame(location=nations,EAPC=rep(0,times=27),UCI=rep(0,times=27),LCI=rep(0,times=27))

for (i in 1:nrow(EAPC_Incidence)){

nation <- as.character(EAPC_Incidence[i,1])

a <- subset(Incidence_EAPC, Incidence_EAPC$location==nation)

a$y <- log(a$val)

mod_simp_reg<-lm(y~year,data=a)

estimate <- (exp(summary(mod_simp_reg)[["coefficients"]][2,1])-1)*100

low <- (exp(summary(mod_simp_reg)[["coefficients"]][2,1]-1.96*summary(mod_simp_reg)[["coefficients"]][2,2])-1)*100

high <- (exp(summary(mod_simp_reg)[["coefficients"]][2,1]+1.96*summary(mod_simp_reg)[["coefficients"]][2,2])-1)*100

EAPC_Incidence[i,2] <- estimate

EAPC_Incidence[i,4] <- low

EAPC_Incidence[i,3] <- high

}

EAPC_Incidence$EAPC <- round(EAPC_Incidence$EAPC,2)

EAPC_Incidence$UCI <- round(EAPC_Incidence$UCI,2)

EAPC_Incidence$LCI <- round(EAPC_Incidence$LCI,2)

EAPC_Incidence$'EAPC_95%CI' <- paste(EAPC_Incidence$LCI,EAPC_Incidence$UCI,sep = ' to ')

EAPC_Incidence$'EAPC_95%CI' <- paste(EAPC_Incidence$'EAPC_95%CI',')',sep = '')

EAPC_Incidence$'EAPC_95%CI' <- paste('(',EAPC_Incidence$'EAPC_95%CI',sep = '')

EAPC_Incidence$'EAPC_95%CI' <- paste(EAPC_Incidence$EAPC,EAPC_Incidence$'EAPC_95%CI',sep = ' ')

EAPC_Incidence <- EAPC_Incidence[,c(1,5)]

Incidence <- Incidence_Num_1990 %>%

left_join(Incidence_ASR_1990,by='location') %>%

left_join(Incidence_Num_2021,by='location') %>%

left_join(Incidence_ASR_2021,by='location') %>%

left_join(EAPC_Incidence,by='location') %>%

arrange(location)

View(Incidence)

write.csv(Incidence,"HL_Incidence.csv")

###Table 2

library(dplyr)

library(ggplot2)

setwd("D:/NHL-data")

IS <- NHL_region %>%

dplyr::select(measure_name,location_name,metric_name,

sex_name,age_name,year,val,lower,upper) %>%

rename(measure=measure_name,

location=location_name,

metric=metric_name,

sex=sex_name,

age=age_name)

###Incidence

###Number

###1990 Incidence

IS_1990 <- IS %>%

filter(year==1990 &

age =='All ages' &

metric == 'Number' &

measure =='Incidence' &

sex =="Both"

) %>%

#Global+5 SDI regions+21 regions

filter(location == 'Global' |

location == 'Low SDI' |

location == 'Low-middle SDI' |

location == 'Middle SDI' |

location == 'High-middle SDI' |

location == 'High SDI' |

location == 'Andean Latin America' |

location == 'Australasia' |

location == 'Caribbean' |

location == 'Central Asia' |

location == 'Central Europe' |

location == 'Central Latin America' |

location == 'Central Sub-Saharan Africa' |

location == 'East Asia' |

location == 'Eastern Europe' |

location == 'Eastern Sub-Saharan Africa' |

location == 'High-income Asia Pacific' |

location == 'High-income North America' |

location == 'North Africa and Middle East' |

location == 'Oceania' |

location == 'South Asia' |

location == 'Southeast Asia' |

location == 'Southern Latin America' |

location == 'Southern Sub-Saharan Africa' |

location == 'Tropical Latin America' |

location == 'Western Europe' |

location == 'Western Sub-Saharan Africa') %>%

dplyr::select(,c(2,7:9))

IS_1990$val <- round(IS_1990$val,0)

IS_1990$lower <- round(IS_1990$lower,0)

IS_1990$upper <- round(IS_1990$upper,0)

IS_1990$'Number 1990' <- paste(IS_1990$lower,IS_1990$upper,sep = '-')

IS_1990$'Number 1990' <- paste(IS_1990$'Number 1990',')',sep = '')

IS_1990$'Number 1990' <- paste('(',IS_1990$'Number 1990',sep = '')

IS_1990$'Number 1990' <- paste(IS_1990$val,IS_1990$'Number 1990',sep = ' ')

Incidence_Num_1990 <- IS_1990[,c(1,5)]

head(Incidence_Num_1990)

## 2021 Incidence

IS_2021 <- IS %>%

filter(year ==2021 &

age =='All ages' &

metric == 'Number' &

measure =='Incidence' &

sex =="Both"

) %>%

#Global+5 SDI regions+21 regions

filter(location == 'Global' |

location == 'Low SDI' |

location == 'Low-middle SDI' |

location == 'Middle SDI' |

location == 'High-middle SDI' |

location == 'High SDI' |

location == 'Andean Latin America' |

location == 'Australasia' |

location == 'Caribbean' |

location == 'Central Asia' |

location == 'Central Europe' |

location == 'Central Latin America' |

location == 'Central Sub-Saharan Africa' |

location == 'East Asia' |

location == 'Eastern Europe' |

location == 'Eastern Sub-Saharan Africa' |

location == 'High-income Asia Pacific' |

location == 'High-income North America' |

location == 'North Africa and Middle East' |

location == 'Oceania' |

location == 'South Asia' |

location == 'Southeast Asia' |

location == 'Southern Latin America' |

location == 'Southern Sub-Saharan Africa' |

location == 'Tropical Latin America' |

location == 'Western Europe' |

location == 'Western Sub-Saharan Africa') %>%

dplyr::select(,c(2,7:9))

IS_2021$val <- round(IS_2021$val,0)

IS_2021$lower <- round(IS_2021$lower,0)

IS_2021$upper <- round(IS_2021$upper,0)

IS_2021$'Number 2021' <- paste(IS_2021$lower,IS_2021$upper,sep = '-')

IS_2021$'Number 2021' <- paste(IS_2021$'Number 2021',')',sep = '')

IS_2021$'Number 2021' <- paste('(',IS_2021$'Number 2021',sep = '')

IS_2021$'Number 2021' <- paste(IS_2021$val,IS_2021$'Number 2021',sep = ' ')

Incidence_Num_2021 <- IS_2021[,c(1,5)]

head(Incidence_Num_2021)

##### Age-standardized rates

## 1990 Incidence

IS_1990 <- IS %>%

filter(year==1990 &

age =='Age-standardized' &

metric == 'Rate' &

measure =='Incidence' &

sex =="Both"

) %>%

#Global+5 SDI regions+21 regions

filter(location == 'Global' |

location == 'Low SDI' |

location == 'Low-middle SDI' |

location == 'Middle SDI' |

location == 'High-middle SDI' |

location == 'High SDI' |

location == 'Andean Latin America' |

location == 'Australasia' |

location == 'Caribbean' |

location == 'Central Asia' |

location == 'Central Europe' |

location == 'Central Latin America' |

location == 'Central Sub-Saharan Africa' |

location == 'East Asia' |

location == 'Eastern Europe' |

location == 'Eastern Sub-Saharan Africa' |

location == 'High-income Asia Pacific' |

location == 'High-income North America' |

location == 'North Africa and Middle East' |

location == 'Oceania' |

location == 'South Asia' |

location == 'Southeast Asia' |

location == 'Southern Latin America' |

location == 'Southern Sub-Saharan Africa' |

location == 'Tropical Latin America' |

location == 'Western Europe' |

location == 'Western Sub-Saharan Africa') %>%

dplyr::select(,c(2,7:9))

IS_1990$val <- round(IS_1990$val,2)

IS_1990$lower <- round(IS_1990$lower,2)

IS_1990$upper <- round(IS_1990$upper,2)

IS_1990$'ASR 1990' <- paste(IS_1990$lower,IS_1990$upper,sep = '-')

IS_1990$'ASR 1990' <- paste(IS_1990$'ASR 1990',')',sep = '')

IS_1990$'ASR 1990' <- paste('(',IS_1990$'ASR 1990',sep = '')

IS_1990$'ASR 1990' <- paste(IS_1990$val,IS_1990$'ASR 1990',sep = ' ')

Incidence_ASR_1990 <- IS_1990[,c(1,5)]

head(Incidence_ASR_1990)

## 2021 Incidence

IS_2021 <- IS %>%

filter(year ==2021 &

age =='Age-standardized' &

metric == 'Rate' &

measure =='Incidence' &

sex =="Both"

) %>%

#Global+5 SDI regions+21 regions

filter(location == 'Global' |

location == 'Low SDI' |

location == 'Low-middle SDI' |

location == 'Middle SDI' |

location == 'High-middle SDI' |

location == 'High SDI' |

location == 'Andean Latin America' |

location == 'Australasia' |

location == 'Caribbean' |

location == 'Central Asia' |

location == 'Central Europe' |

location == 'Central Latin America' |

location == 'Central Sub-Saharan Africa' |

location == 'East Asia' |

location == 'Eastern Europe' |

location == 'Eastern Sub-Saharan Africa' |

location == 'High-income Asia Pacific' |

location == 'High-income North America' |

location == 'North Africa and Middle East' |

location == 'Oceania' |

location == 'South Asia' |

location == 'Southeast Asia' |

location == 'Southern Latin America' |

location == 'Southern Sub-Saharan Africa' |

location == 'Tropical Latin America' |

location == 'Western Europe' |

location == 'Western Sub-Saharan Africa') %>%

dplyr::select(,c(2,7:9))

IS_2021$val <- round(IS_2021$val,2)

IS_2021$lower <- round(IS_2021$lower,2)

IS_2021$upper <- round(IS_2021$upper,2)

IS_2021$'ASR 2021' <- paste(IS_2021$lower,IS_2021$upper,sep = '-')

IS_2021$'ASR 2021' <- paste(IS_2021$'ASR 2021',')',sep = '')

IS_2021$'ASR 2021' <- paste('(',IS_2021$'ASR 2021',sep = '')

IS_2021$'ASR 2021' <- paste(IS_2021$val,IS_2021$'ASR 2021',sep = ' ')

Incidence_ASR_2021 <- IS_2021[,c(1,5)]

head(Incidence_ASR_2021)

##### EAPC

Incidence_EAPC <- IS %>%

filter(age =='Age-standardized' &

metric == 'Rate' &

measure =='Incidence'&

sex =="Both"

) %>%

#Global+5 SDI regions+21 regions

filter(location == 'Global' |

location == 'Low SDI' |

location == 'Low-middle SDI' |

location == 'Middle SDI' |

location == 'High-middle SDI' |

location == 'High SDI' |

location == 'Andean Latin America' |

location == 'Australasia' |

location == 'Caribbean' |

location == 'Central Asia' |

location == 'Central Europe' |

location == 'Central Latin America' |

location == 'Central Sub-Saharan Africa' |

location == 'East Asia' |

location == 'Eastern Europe' |

location == 'Eastern Sub-Saharan Africa' |

location == 'High-income Asia Pacific' |

location == 'High-income North America' |

location == 'North Africa and Middle East' |

location == 'Oceania' |

location == 'South Asia' |

location == 'Southeast Asia' |

location == 'Southern Latin America' |

location == 'Southern Sub-Saharan Africa' |

location == 'Tropical Latin America' |

location == 'Western Europe' |

location == 'Western Sub-Saharan Africa') %>%

dplyr::select(,c(2,6,7))

nations <- IS_1990$location

EAPC_Incidence <- data.frame(location=nations,EAPC=rep(0,times=27),UCI=rep(0,times=27),LCI=rep(0,times=27))

for (i in 1:nrow(EAPC_Incidence)){

nation <- as.character(EAPC_Incidence[i,1])

a <- subset(Incidence_EAPC, Incidence_EAPC$location==nation)

a$y <- log(a$val)

mod_simp_reg<-lm(y~year,data=a)

estimate <- (exp(summary(mod_simp_reg)[["coefficients"]][2,1])-1)*100

low <- (exp(summary(mod_simp_reg)[["coefficients"]][2,1]-1.96*summary(mod_simp_reg)[["coefficients"]][2,2])-1)*100

high <- (exp(summary(mod_simp_reg)[["coefficients"]][2,1]+1.96*summary(mod_simp_reg)[["coefficients"]][2,2])-1)*100

EAPC_Incidence[i,2] <- estimate

EAPC_Incidence[i,4] <- low

EAPC_Incidence[i,3] <- high

}

EAPC_Incidence$EAPC <- round(EAPC_Incidence$EAPC,2)

EAPC_Incidence$UCI <- round(EAPC_Incidence$UCI,2)

EAPC_Incidence$LCI <- round(EAPC_Incidence$LCI,2)

EAPC_Incidence$'EAPC_95%CI' <- paste(EAPC_Incidence$LCI,EAPC_Incidence$UCI,sep = ' to ')

EAPC_Incidence$'EAPC_95%CI' <- paste(EAPC_Incidence$'EAPC_95%CI',')',sep = '')

EAPC_Incidence$'EAPC_95%CI' <- paste('(',EAPC_Incidence$'EAPC_95%CI',sep = '')

EAPC_Incidence$'EAPC_95%CI' <- paste(EAPC_Incidence$EAPC,EAPC_Incidence$'EAPC_95%CI',sep = ' ')

EAPC_Incidence <- EAPC_Incidence[,c(1,5)]

Incidence <- Incidence_Num_1990 %>%

left_join(Incidence_ASR_1990,by='location') %>%

left_join(Incidence_Num_2021,by='location') %>%

left_join(Incidence_ASR_2021,by='location') %>%

left_join(EAPC_Incidence,by='location') %>%

arrange(location)

View(Incidence)

write.csv(Incidence,"NHL_Incidence.csv")

###Table 3

library(dplyr)

library(ggplot2)

setwd("D:/AML-data")

IS <- AML_region %>%

dplyr::select(measure_name,location_name,metric_name,

sex_name,age_name,year,val,lower,upper) %>%

rename(measure=measure_name,

location=location_name,

metric=metric_name,

sex=sex_name,

age=age_name)

###Incidence

###Number

###1990 Incidence

IS_1990 <- IS %>%

filter(year==1990 &

age =='All ages' &

metric == 'Number' &

measure =='Incidence' &

sex =="Both"

) %>%

#Global+5 SDI regions+21 regions

filter(location == 'Global' |

location == 'Low SDI' |

location == 'Low-middle SDI' |

location == 'Middle SDI' |

location == 'High-middle SDI' |

location == 'High SDI' |

location == 'Andean Latin America' |

location == 'Australasia' |

location == 'Caribbean' |

location == 'Central Asia' |

location == 'Central Europe' |

location == 'Central Latin America' |

location == 'Central Sub-Saharan Africa' |

location == 'East Asia' |

location == 'Eastern Europe' |

location == 'Eastern Sub-Saharan Africa' |

location == 'High-income Asia Pacific' |

location == 'High-income North America' |

location == 'North Africa and Middle East' |

location == 'Oceania' |

location == 'South Asia' |

location == 'Southeast Asia' |

location == 'Southern Latin America' |

location == 'Southern Sub-Saharan Africa' |

location == 'Tropical Latin America' |

location == 'Western Europe' |

location == 'Western Sub-Saharan Africa') %>%

dplyr::select(,c(2,7:9))

IS_1990$val <- round(IS_1990$val,0)

IS_1990$lower <- round(IS_1990$lower,0)

IS_1990$upper <- round(IS_1990$upper,0)

IS_1990$'Number 1990' <- paste(IS_1990$lower,IS_1990$upper,sep = '-')

IS_1990$'Number 1990' <- paste(IS_1990$'Number 1990',')',sep = '')

IS_1990$'Number 1990' <- paste('(',IS_1990$'Number 1990',sep = '')

IS_1990$'Number 1990' <- paste(IS_1990$val,IS_1990$'Number 1990',sep = ' ')

Incidence_Num_1990 <- IS_1990[,c(1,5)]

head(Incidence_Num_1990)

## 2021 Incidence

IS_2021 <- IS %>%

filter(year ==2021 &

age =='All ages' &

metric == 'Number' &

measure =='Incidence' &

sex =="Both"

) %>%

#Global+5 SDI regions+21 regions

filter(location == 'Global' |

location == 'Low SDI' |

location == 'Low-middle SDI' |

location == 'Middle SDI' |

location == 'High-middle SDI' |

location == 'High SDI' |

location == 'Andean Latin America' |

location == 'Australasia' |

location == 'Caribbean' |

location == 'Central Asia' |

location == 'Central Europe' |

location == 'Central Latin America' |

location == 'Central Sub-Saharan Africa' |

location == 'East Asia' |

location == 'Eastern Europe' |

location == 'Eastern Sub-Saharan Africa' |

location == 'High-income Asia Pacific' |

location == 'High-income North America' |

location == 'North Africa and Middle East' |

location == 'Oceania' |

location == 'South Asia' |

location == 'Southeast Asia' |

location == 'Southern Latin America' |

location == 'Southern Sub-Saharan Africa' |

location == 'Tropical Latin America' |

location == 'Western Europe' |

location == 'Western Sub-Saharan Africa') %>%

dplyr::select(,c(2,7:9))

IS_2021$val <- round(IS_2021$val,0)

IS_2021$lower <- round(IS_2021$lower,0)

IS_2021$upper <- round(IS_2021$upper,0)

IS_2021$'Number 2021' <- paste(IS_2021$lower,IS_2021$upper,sep = '-')

IS_2021$'Number 2021' <- paste(IS_2021$'Number 2021',')',sep = '')

IS_2021$'Number 2021' <- paste('(',IS_2021$'Number 2021',sep = '')

IS_2021$'Number 2021' <- paste(IS_2021$val,IS_2021$'Number 2021',sep = ' ')

Incidence_Num_2021 <- IS_2021[,c(1,5)]

head(Incidence_Num_2021)

##### Age-standardized rates

## 1990 Incidence

IS_1990 <- IS %>%

filter(year==1990 &

age =='Age-standardized' &

metric == 'Rate' &

measure =='Incidence' &

sex =="Both"

) %>%

#Global+5 SDI regions+21 regions

filter(location == 'Global' |

location == 'Low SDI' |

location == 'Low-middle SDI' |

location == 'Middle SDI' |

location == 'High-middle SDI' |

location == 'High SDI' |

location == 'Andean Latin America' |

location == 'Australasia' |

location == 'Caribbean' |

location == 'Central Asia' |

location == 'Central Europe' |

location == 'Central Latin America' |

location == 'Central Sub-Saharan Africa' |

location == 'East Asia' |

location == 'Eastern Europe' |

location == 'Eastern Sub-Saharan Africa' |

location == 'High-income Asia Pacific' |

location == 'High-income North America' |

location == 'North Africa and Middle East' |

location == 'Oceania' |

location == 'South Asia' |

location == 'Southeast Asia' |

location == 'Southern Latin America' |

location == 'Southern Sub-Saharan Africa' |

location == 'Tropical Latin America' |

location == 'Western Europe' |

location == 'Western Sub-Saharan Africa') %>%

dplyr::select(,c(2,7:9))

IS_1990$val <- round(IS_1990$val,2)

IS_1990$lower <- round(IS_1990$lower,2)

IS_1990$upper <- round(IS_1990$upper,2)

IS_1990$'ASR 1990' <- paste(IS_1990$lower,IS_1990$upper,sep = '-')

IS_1990$'ASR 1990' <- paste(IS_1990$'ASR 1990',')',sep = '')

IS_1990$'ASR 1990' <- paste('(',IS_1990$'ASR 1990',sep = '')

IS_1990$'ASR 1990' <- paste(IS_1990$val,IS_1990$'ASR 1990',sep = ' ')

Incidence_ASR_1990 <- IS_1990[,c(1,5)]

head(Incidence_ASR_1990)

## 2021 Incidence

IS_2021 <- IS %>%

filter(year ==2021 &

age =='Age-standardized' &

metric == 'Rate' &

measure =='Incidence' &

sex =="Both"

) %>%

#Global+5 SDI regions+21 regions

filter(location == 'Global' |

location == 'Low SDI' |

location == 'Low-middle SDI' |

location == 'Middle SDI' |

location == 'High-middle SDI' |

location == 'High SDI' |

location == 'Andean Latin America' |

location == 'Australasia' |
[truncated: 238,175 more chars]
