# Supplementary material for: Global burden and trends of hematologic malignancies based on Global Cancer Observatory 2022 and Global Burden of Disease 2021
Source: Exp Hematol Oncol. 2025 Jul 17;14:98. doi: 10.1186/s40164-025-00684-x (PMC12273037; doi:10.1186/s40164-025-00684-x)
Supplement: Supplementary file 5 — Supplementary Material 5 [file 40164_2025_684_MOESM5_ESM.docx]

**Supplementary Tables:**

**Table S1**: Summary of the crucial disparities between GBD 2021 and GLOBOCAN 2022.

**Table S2**: The prevalence of Hodgkin lymphoma in 1990 and 2021.

**Table S3**: The death of HL in 1990 and 2021.

**Table S4**: The DALYs of Hodgkin lymphoma in 1990 and 2021.

**Table S5**: 204 countries of Hodgkin lymphoma prevalence, incidence, deaths, DALYs, ASPR, ASIR, ASDR, and ASDALYR in 2021.

**Table S6**: The prevalence of Non − Hodgkin lymphoma in 1990 and 2021.

**Table S7**: The death of NHL in 1990 and 2021.

**Table S8**: The DALYs of Non − Hodgkin lymphoma in 1990 and 2021.

**Table S9**: 204 countries of Non − Hodgkin lymphoma prevalence, incidence, deaths, DALYs, ASPR, ASIR, ASDR, and ASDALYR in 2021.

**Table S10**: The prevalence of Acute myeloid leukemia in 1990 and 2021.

**Table S11**: The death of AML in 1990 and 2021.

**Table S12**: The DALYs of Acute myeloid leukemia in 1990 and 2021.

**Table S13**: 204 countries of Acute myeloid leukemia prevalence, incidence, deaths, DALYs, ASPR, ASIR, ASDR, and ASDALYR in 2021.

**Table S14**: The prevalence of Chronic myeloid leukemia in 1990 and 2021.

**Table S15**: The death of CML in 1990 and 2021.

**Table S16**: The DALYs of Chronic myeloid leukemia in 1990 and 2021.

**Table S17**: 204 countries of Chronic myeloid leukemia prevalence, incidence, deaths, DALYs, ASPR, ASIR, ASDR, and ASDALYR in 2021.

**Table S18**: The prevalence of Acute lymphoid leukemia in 1990 and 2021.

**Table S19**: The death of ALL in 1990 and 2021.

**Table S20**: The DALYs of Acute lymphoid leukemia in 1990 and 2021.

**Table S21**: 204 countries of Acute lymphoid leukemia prevalence, incidence, deaths, DALYs, ASPR, ASIR, ASDR, and ASDALYR in 2021.

**Table S22**: The prevalence of Chronic lymphoid leukemia in 1990 and 2021.

**Table S23**: The death of CLL in 1990 and 2021.

**Table S24**: The DALYs of Chronic lymphoid leukemia in 1990 and 2021.

**Table S25**: 204 countries of Chronic lymphoid leukemia prevalence, incidence, deaths, DALYs, ASPR, ASIR, ASDR, and ASDALYR in 2021.

**Table S26**: The prevalence of Multiple myeloma in 1990 and 2021.

**Table S27**: The death of MM in 1990 and 2021.

**Table S28**: The DALYs of Multiple myeloma in 1990 and 2021.

**Table S29**: 204 countries of Multiple myeloma prevalence, incidence, deaths, DALYs, ASPR, ASIR, ASDR, and ASDALYR in 2021.

**Supplementary Methods:** Relevant analysis and visualization codes.
